# Identification of Hub Genes Related to Carcinogenesis and Prognosis in Colorectal Cancer Based on Integrated Bioinformatics

**DOI:** 10.1155/2020/5934821

**Published:** 2020-04-09

**Authors:** Benjiao Gong, Yanlei Kao, Chenglin Zhang, Fudong Sun, Zhaohua Gong, Jian Chen

**Affiliations:** ^1^The Central Laboratory, Affiliated Yantai Yuhuangding Hospital of Qingdao University, Yantai, Shandong, China; ^2^Department of Spleen and Stomach Diseases, Yantai Hospital of Traditional Chinese Medicine, Yantai, Shandong, China; ^3^Pharmacy Department, Affiliated Yantai Yuhuangding Hospital of Qingdao University, Yantai, Shandong, China; ^4^Department of Oncology, Affiliated Yantai Yuhuangding Hospital of Qingdao University, Yantai, Shandong, China

## Abstract

The high mortality of colorectal cancer (CRC) patients and the limitations of conventional tumor-node-metastasis (TNM) stage emphasized the necessity of exploring hub genes closely related to carcinogenesis and prognosis in CRC. The study is aimed at identifying hub genes associated with carcinogenesis and prognosis for CRC. We identified and validated 212 differentially expressed genes (DEGs) from six Gene Expression Omnibus (GEO) datasets and the Cancer Genome Atlas (TCGA) database. We investigated functional enrichment analysis for DEGs. The protein-protein interaction (PPI) network was constructed, and hub modules and genes in CRC carcinogenesis were extracted. A prognostic signature was developed and validated based on Cox proportional hazards regression analysis. The DEGs mainly regulated biological processes covering response to stimulus, metabolic process, and affected molecular functions containing protein binding and catalytic activity. The DEGs played important roles in CRC-related pathways involving in preneoplastic lesions, carcinogenesis, metastasis, and poor prognosis. Hub genes closely related to CRC carcinogenesis were extracted including six genes in model 1 (CXCL1, CXCL3, CXCL8, CXCL11, NMU, and PPBP) and two genes and Metallothioneins (MTs) in model 2 (SLC26A3 and SLC30A10). Among them, CXCL8 was also related to prognosis. An eight-gene signature was proposed comprising AMH, WBSCR28, SFTA2, MYH2, POU4F1, SIX4, PGPEP1L, and PAX5. The study identified hub genes in CRC carcinogenesis and proposed an eight-gene signature with good reproducibility and robustness at the molecular level for CRC, which might provide directive significance for treatment selection and survival prediction.

## 1. Introduction

Colorectal cancer (CRC) is diagnosed the second most cancer in females and the third most form in males, which has been a major global public health problem [1]. The number of cases diagnosed is forecast to rise from 1800 million now to 3093 million by 2040 through the World Health Organization [2]. Although modern medicine has made great advances, CRC is still the third leading cause for cancer-related mortality [3]. As we all know, early detection of CRC has some effect on reducing its mortality and the discovery of precursor lesion can even cut down the incidence [4]. Early diagnosis with better survival and later diagnosis with worse prognosis have no doubt. Tumor-node-metastasis (TNM) stage, identified by the American Joint Committee on Cancer according to pathologic and clinical factors, is not only the fundamental for treatment but also the gold standard for CRC prognosis [5, 6]. The 5-year survival rate at stage I is more than 90%, and the 5-year survival rate for stage IV is only 10% [7]. However, 20% of patients at stage II undergo cancer-specific death and some stage III patients confront better outcomes than some patients at stage II [8]. Hence, it is extremely necessary to identify novel prognostic biomarkers for early diagnostic detection and improving outcomes due to the limitation of TNM stage.

In recent decades, the research on the molecular and genetic mechanisms in CRC carcinogenesis and progression has accelerated the investigation of genetic prognostic markers for the TNM staging system supplement [9]. And the progress of microarray and high-throughput sequencing technology has also promoted to interpret epigenetic or critical genetic alternations in carcinogenesis and to decipher hopeful biomarkers for cancer diagnosis, treatment, and prognosis [10, 11]. Publicly available genome databases like the Cancer Genome Atlas (TCGA) and the Gene Expression Omnibus (GEO) have provided more facilitated genome exploration on different cancers containing CRC for clinicians and bioinformatics, which was generally impossible in the past [12–15]. Meanwhile, integrated bioinformatics methods have been applied to cancer research and large amounts of valuable information have been excavated, which were explored to overcome the restricted or discordant results because of the application of either a small sample size or different types of technological platforms [16–19].

In this study, we identified and integrated differentially expressed genes (DEGs) from gene expression profile and RNA sequencing data for human CRC. The DEGs were further preformed functional enrichment analysis to investigate biological processes, molecular functions, and reactome pathways regulated by the DEGs. The protein-protein interaction (PPI) network reflecting the interactions among DEGs was constructed, and hub network modules were captured and deciphered, which embodied representative genes in CRC carcinogenesis. Finally, patients with overall survival data were randomly divided into two groups, the train group and the test group. The train group was used to reveal genes associated with survival and build a CRC gene signature for prognosis. The test group was employed to assess the prognosis model comprehensively.

## 2. Materials and Methods

### 2.1. DEG Identification by GEO

The gene expression profile data (GSE21510, GSE24514, GSE32323, GSE89076, GSE110225, and GSE113513) for colorectal cancer were extracted from the GEO database [20–24]. All included datasets contained at least 10 samples. The normalization and log_2_ conversion were performed for the matrix data of each GEO dataset, and the DEGs between tumor and control tissues were filtered out via the Limma package in R [25]. Gene integration for the DEGs screened from the six datasets was executed using the RobustRankAggreg (RRA) package based on a robust rank aggregation method [26]. |log_2_FC | >1.5 and adjusted *P* value < 0.05 set the criteria to filter statistically significant DEGs.

### 2.2. DEG Validation by TCGA

The integrated significant DEGs from GEO datasets were validated by means of RNA sequencing data in TCGA COADREAD dataset. Raw RNA sequencing data including 647 COADREAD samples and 51 matched noncancerous samples were extracted from TCGA database, and the clinical information of patients was also downloaded. The Mann-Whitney test was employed to normalize and analyze the TCGA data. Genes with |log_2_FC | >2 and adjusted *P* value < 0.05 were considered to be significantly differentially expressed. Overlapping DEGs between GEO and TCGA database were reserved for following studies.

### 2.3. Functional Enrichment Analysis

The potential biological processes and molecular functions of the overlapping DEGs were evaluated using BINGO plug-in of Cytoscape 3.2.1 [27]. During this procedure, the significance level was set to 0.05, and organism was selected as Homo sapiens. The pathway enrichment analysis was performed utilizing Reactome FI plug-in of Cytoscape 3.2.1, and the threshold level was defined as FDR < 0.05 [28]. The top ten terms of the functional enrichment analysis were visualized using the Bubble package [29].

### 2.4. PPI Network and Module Analysis

The protein-protein interactions among overlapping DEGs were identified via STRING database, and genes with the combined score ≥ 0.4 were selected to construct the PPI network [30]. The PPI network was visualized and analyzed by Cytoscape 3.2.1. And the hub network modules were captured with the help of the Cytoscape plug-in Molecular Complex Detection (MCODE) with parameters degree cutoff = 2, Node Score Cutoff = 0.2, and K − Core = 2 [31]. Then, the topological parameters were also calculated, and survival analysis was performed using clinical information via the survival package for hub modules.

### 2.5. COX Model Construction and Verification

After eliminating patients without overall survival data, 617 patients' data were used for survival analysis. All patients were randomly divided into two groups with the help of the caret package, train group and test group [32]. The train group was used for constructing the COX prognostic signature, and the test group was used for validating the signature. The train group executed univariate Cox proportional hazards regression analysis to recognize candidate genes associated with survival. Then, the LASSO penalized regression model was employed to achieve shrinkage and variable selection simultaneously and to prevent the prognostic model overfitting. Subsequently, the multivariate Cox proportional hazards regression model was performed and corresponding coefficients were calculated in the train group. The predicted overall survival information with a risk score for each patient in two groups was assessed on the basis of the expression level of the prognostic gene and its corresponding coefficient in the train group. The patients in two groups were classified into low- or high-risk groups according to the median risk score of the train group. Survival curves were plotted utilizing the survival package to assess the differences in survival rate between high- and low-risk patients in two groups. Furthermore, the receiver operating characteristic (ROC) curve was constructed based on the survivalROC package and the area under the curve (AUC) was measured to evaluate the predictive ability of the prognostic signature for clinical outcomes. The risk score distribution, survival time, and gene expression patterns for patients in the train and test groups were visualized in R.

## 3. Results

### 3.1. DEG Identification and Validation

**Figure 1:**
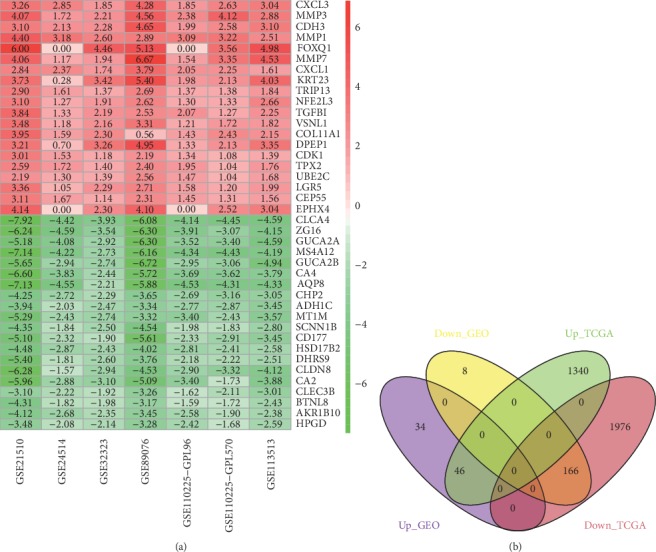
DEG identification from GEO and validation from TCGA. (a) The top 20 up- and downregulated genes in six GEO datasets based on a RRA package. (b) Overlapping DEGs between GEO and TCGA database.

The detailed information for the six GEO datasets in this study is shown in Table 1. 254 DEGs in total including 80 upregulated genes and 174 downregulated genes were obtained through screening of the Limma package and integration of the RRA package for the six datasets (Table S1). The top 20 up- and downregulated genes after the integrated analysis are displayed in Figure 1(a). The DEGs extracted from TCGA database comprised 1386 upregulated and 2142 downregulated genes (Table S2). Finally, 212 overlapping DEGs containing 46 upregulated and 166 downregulated genes were identified (Figure 1(b) and Table S3). In addition, the clinical information of patients was also organized for survival analysis (Table S4).

### 3.2. Functional Enrichment Analysis

**Figure 2:**
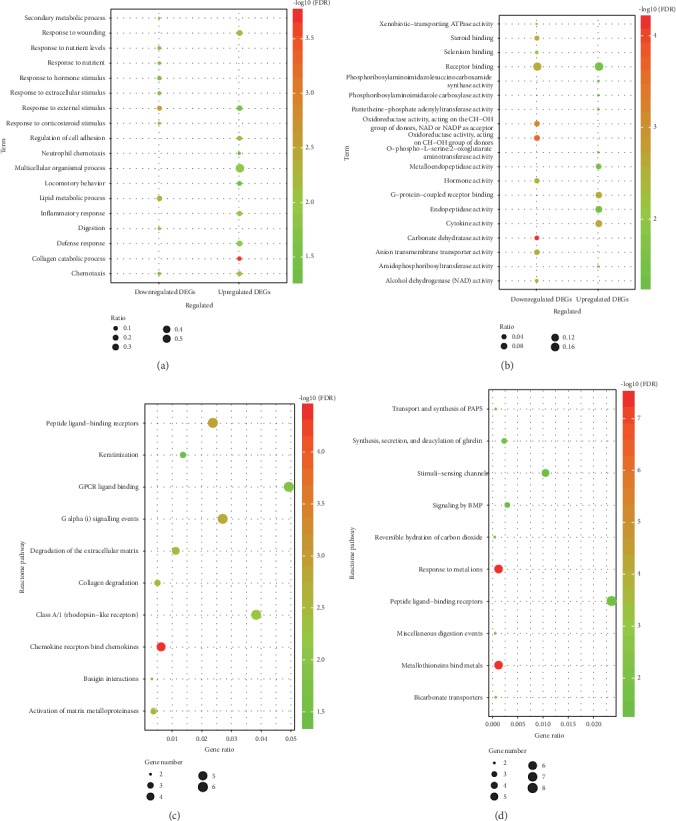
Functional enrichment analysis for DEGs. (a) The top 10 terms of biological process enrichment for up- and downregulated DEGs. (b) The top 10 terms of molecular function enrichment for up- and downregulated DEGs. (c) The top 10 terms of reactome pathway enrichment for upregulated DEGs. (d) The top 10 terms of reactome pathway enrichment for downregulated DEGs.

To explain the potential biological functions of the 212 overlapping DEGs, the biological process, molecular function, and reactome pathway enrichment analyses were executed. The biological processes were mainly involved in response to stimulus and metabolic process (Figure 2(a) and Table S5). The molecular functions were significantly enriched in protein binding and catalytic activity (Figure 2(b) and Table S6). According to the reactome pathway enrichment analysis, the upregulated genes were mainly associated with signaling by GPCR and extracellular matrix organization (Figure 2(c) and Table S7). And the downregulated genes participated in response to metal ions, metabolism, signal transduction, and transmembrane transport of small molecules (Figure 2(d) and Table S8).

### 3.3. PPI Network and Module Analysis

**Figure 3:**
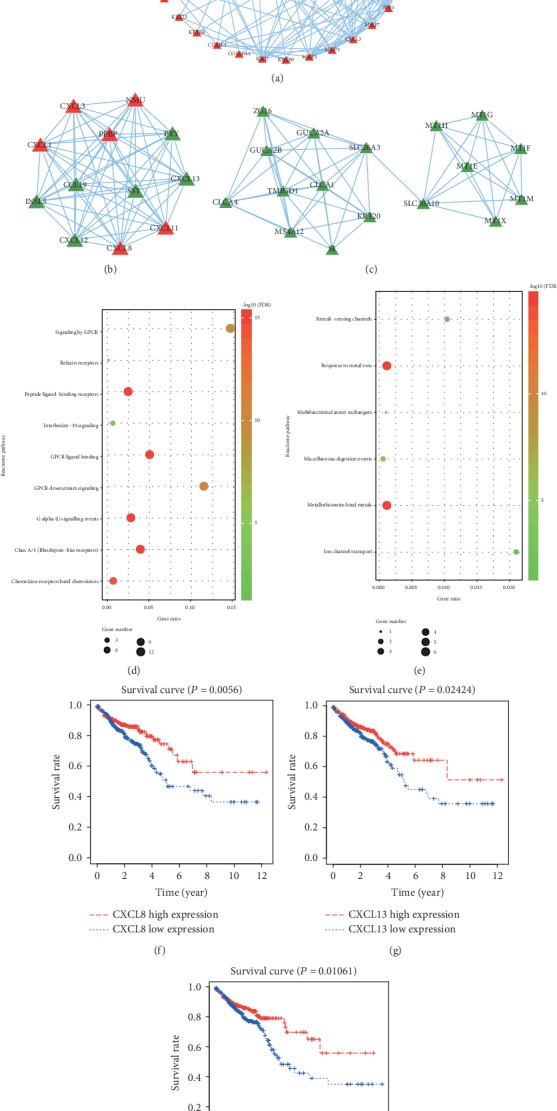
Construction of PPI network and module analysis. (a) The PPI network with red nodes for upregulated genes and green nodes for downregulated genes. (b) Module 1 of PPI network. (c) Module 2 of PPI network. (d) Reactome pathway enrichment for module 1. (e) Reactome pathway enrichment for module 2. (f) Survival curve of CXCL8. (g) Survival curve of CXCL13. (h) Survival curve of CLCA1.

The PPIs between 37 upregulated and 131 downregulated genes were excavated via STRING database with the combined score ≥ 0.4, and the PPI network was displayed containing 168 nodes and 417 interactions (Figure 3(a) and Table S9). To further investigate the hub network modules from the complex network, two hub modules with a score > 5 were extracted based on MCODE (Figures 3(b) and 3(c)). And three topological parameters covering degree, closeness centrality, and betweenness centrality were calculated to measure hub nodes in hub network modules (Tables S10 and S11). Hub genes with parameters greater than the mean of each group were considered to reflect key biological characteristics in the network module. However, all parameters were the same in model 1, but CXCL family genes accounted for a half. SLC26A3 and SLC30A10 were defined as hub genes in model 2. Then, the impact of the two modules on the pathways was also investigated. The genes in model 1 were significantly enriched in nine pathways, and the top five pathways coincided with the pathways that 46 upregulated genes mainly regulated, which might indicate that the upregulated genes in model 1 were dominant (Figure 3(d)). The genes in model 2 mainly gathered in six pathways, and the top five pathways were consistent with the pathways affected by 166 downregulated genes, which revealed that Metallothioneins (MTs) played an important role in model 2 (Figure 3(e)). Survival analysis of hub modules suggested CXCL8, CXCL13, and CLCA1 were associated with prognosis (*P* < 0.05), and the high expression group presented better prognosis (Figures 3(f)–3(h)).

### 3.4. COX Model Construction and Verification

**Figure 4:**
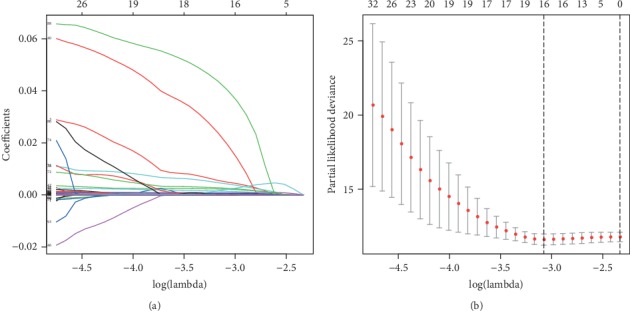
LASSO regression analysis for the train group. (a) LASSO coefficient profiles of prognostic genes with *P* < 0.001. (b) Selection of the optimal value of lambda via 10-fold cross-validations.

**Figure 5:**
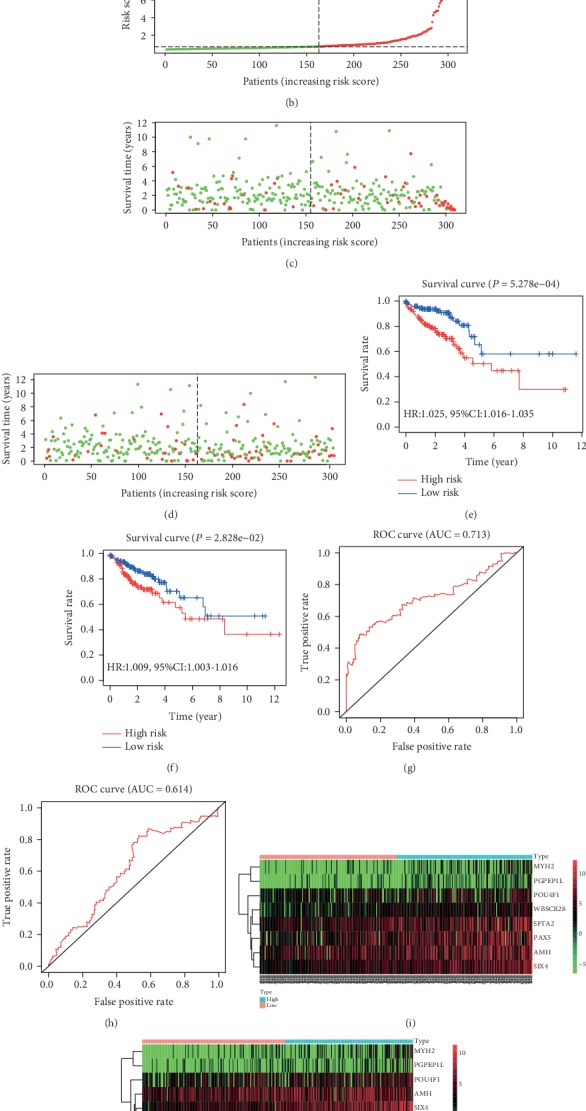
The evaluation and confirmation of the eight-gene signature. (a) The risk score distribution for the train group. (b) The risk score distribution for the test group. (c) The survival time statistic for the train group. (d) The survival time statistic for the test group. (e) Survival curve for the train group. (f) Survival curve for the test group. (g) ROC curve for the train group. (h) ROC curve for the test group. (i) Gene expression pattern for the train group. (j) Gene expression pattern for the test group.

The 617 patients' data were randomly divided into two groups, the train group (309) and the test group (308). In all, 102 genes were captured through the univariate Cox proportional hazards regression model in the train group, which were significantly associated with survival time (*P* < 0.001) and all belonged to high-risk genes (HR > 1) (Table S12). Then, 16 representative genes were screened out through shrinkage and variable selection simultaneously of the LASSO penalized regression model in the train group (Figures 4(a) and 4(b) and Table S13). A prognostic gene signature involved in eight genes was developed using the multivariate Cox proportional hazards regression model, covering Muellerian-inhibiting factor (AMH), transmembrane protein 270 (WBSCR28), surfactant-associated protein 2 (SFTA2), myosin-2 (MYH2), POU domain, class 4, transcription factor 1 (POU4F1), homeobox protein SIX4 (SIX4), pyroglutamyl-peptidase 1-like protein (PGPEP1L), and paired box protein Pax-5 (PAX5) (Table 2). All the eight genes with HR > 1 were identified as risky prognostic genes, which implied that the patient's risk increased along with the rising of the gene expression. The risk scores were calculated based on the gene expression values and relevant coefficients, and all patients were divided to high- or low-risk groups based on the median risk score of the train group (Figures 5(a) and 5(b)). The survival time statistics in high- and low-risk groups are exhibited in Figures 5(c) and 5(d). Obviously, a significant difference in survival rate was represented between the high- and low-risk groups in the train group in Figure 5(e), and Figure 5(f) verifies the existence of the significant difference in the test group. The survival rates of the low-risk group were 94.3% (95% CI: 90.6%-98.2%), 88.6% (95% CI: 82.1%-95.6%), and 65.3% (95% CI: 49.3%-86.4%) for 1, 3, and 5 years, respectively, compared with 85.8% (95% CI: 80.2%-91.8%), 70.3% (95% CI: 62.0%-79.7%), and 50.4% (95% CI: 37.0%-68.5%) for the high-risk group in the train group. The accuracy of the prognostic gene signature in survival prediction was presented with AUC as 0.713 and 0.614, respectively, for the train group and the test group (Figures 5(g) and 5(h)). With the rising of the risk score, the distribution of the gene expression trend is revealed in Figures 5(i) and 5(j).

## 4. Discussion

At the moment, TNM stage is the principal guideline for treatment selection and prognosis prediction of CRC patients. In clinical practice, CRC patients with similar histopathological characteristics presented significantly different prognosis or diverse responses to treatment, which might be associated with the high molecular heterogeneity of CRC and could expose the TNM stage limitations towards precision medicine in CRC [33–35]. Moreover, although increasing studies concerning biomarkers have been accumulated focusing on tumor diagnosis, treatment, and prognosis, there are scarce biomarkers utilized for early diagnosis, treatment selection, and predicting outcome in clinical. Thus, reliable prognostic biomarkers capable of differentiating patients' prognosis are still desperately required in CRC.

In this research, 254 DEGs containing 80 upregulated genes and 174 downregulated genes were screened and integrated from six GEO datasets and were mapped into RNA sequencing data from TCGA to extract 212 overlapping DEGs containing 46 upregulated and 166 downregulated genes. The biological process analysis suggested that the upregulated genes were mainly implicated in multiple metabolic processes including collagen catabolic process, multicellular organismal catabolic process, collagen metabolic process, multicellular organismal macromolecule metabolic process, and multicellular organismal metabolic process. The downregulated genes were primarily involved in various responses to stimulus, response to chemical stimulus like chemotaxis and response to nutrient, response to external stimulus like taxis and response to extracellular stimulus, and response to endogenous stimulus like response to glucocorticoid stimulus, response to corticosteroid stimulus, response to steroid hormone stimulus, and response to hormone stimulus. The molecular function analysis showed that the upregulated genes chiefly affected protein binding containing chemokine activity, chemokine receptor binding, cytokine activity, G-protein-coupled receptor binding, receptor binding, etc. The downregulated genes had much effect on catalytic activity such as lyase activity, oxidoreductase activity, transferase activity, and hydrolase activity. For the reactome pathway enrichment analysis, the upregulated genes mostly focused on regulation of the immune system and inflammation and cancer cell invasion and metastasis [36, 37]. The downregulated genes played important roles in CRC-related pathways involving in preneoplastic lesions, carcinogenesis, metastasis, and poor prognosis [38–40].

Two hub modules were also identified, and topological parameters were calculated in the PPI network. Topological parameters of genes in module 1 were not significantly different, but the pathway enrichment results mainly accumulated in pathways regulated by 46 upregulated genes, which revealed the major status of CXCL1, CXCL3, CXCL8, CXCL11, NMU, and PPBP. Increased CXCL1 levels had positive relationships with tumor size, degree of invasion, advancing stage, metastasis, and poor prognosis [41, 42]. High expression of CXCL3 was detected in premalignant adenomas and CRC tissue, and CXCL3 significantly downregulated in liver metastasis compared with the primary tumor. And CXCL3 obviously presented high expression in patients with local relative to systemic disease [43]. On the contrary, overexpression of CXCL8 promoted proliferation, migration, and invasion of CRC cells, which was strongly correlated with CRC angiogenesis, metastasis, poor prognosis, and disease-free survival [44, 45]. However, high expression of CXCL8 could act as a protective barrier for liver metastasis of CRC and coincide with better prognosis [46, 47]. Objectively, the role of CXCL8 still remained in dispute. This study confirmed that CXCL8 was associated with prognosis and suggested that the high CXCL8 expression group had a better prognosis than the low expression group. Besides angiogenesis, CXCL11 was an important cytokine in the progression of inflammation to CRC and induced tumor-associated macrophages to infiltrate, which enhanced the proliferation and invasion of CRC cells and generated poor prognosis [48–50]. NMU was capable of facilitating the proliferation, migration, and invasion of CRC cells [51]. PPBP, also known as CXCL7, was overexpressed in CRC and associated with poor prognosis and disease-free survival [52]. SLC26A3 and SLC30A10 were uncovered as hub genes in model 2, and the top 2 significant pathways hit on MT1M, MT1X, MT1F, MT1G, MT1H, and MT1E, which occupied the one-sided subnetwork of model 2. SLC26A3 downexpressed in CRC played a tumor suppressor role and was expected to be a candidate epithelial marker in CRC [53, 54]. SLC30A10 was acceptable to classify methylation epigenotypes and correlated with molecular genesis in CRC [55]. MTs, a protein family of low molecular weight and full of cysteine, contained at least 11 functional isoforms and implicated in zinc and redox metabolism. MTs were epigenetically downregulated in CRC early progression (especially MT1G) and tended to induce a worse prognosis [56]. MT overexpression represented a crucial early step in the development of ulcerative colitis-associated CRC [57]. MT expression was also a potential reminder affecting lymph node metastases, particularly in patients with synchronous liver metastases [40]. MT1G uncovered the capability of tumor suppressor via promoting CRC differentiation through zinc signaling [58]. Also, MT1G overexpression sensitized CRC cells to oxaliplatin and 5-fluorouracil via activating p53 and repressing NF-*κ*B activity [59]. In addition, CXCL13 and CLCA1 in hub modules were downregulated, and high expression of that had a better prognosis. CXCL13 showed significantly lower expression in CRC, and patients with CXCL13 deletion had a significantly higher risk of relapse [60]. CLCA1 was also reported to be involved in the pathophysiology of CRC, and upregulation of CLCA1 was associated with a favorable prognosis [61, 62].

In the present study, we detected the association between gene expression and prognosis in CRC patients by recruiting RNA sequencing data for 3528 genes of 309 patients and identified 102 genes significantly associated with CRC patients' overall survival. After removing gene information highly correlated, an eight-gene signature was developed and risk scores were evaluated, which classified CRC patients into high- and low-risk groups with significantly different overall survival. The test group validated the prognostic value of the eight-gene signature capable of good reproducibility and robustness, which suggested that the eight-gene signature could improve prognostic prediction at the molecular level beyond the conventional TNM stage. The eight-gene signature also pushed the limitation of traditional TNM stage for prognostic prediction due to molecular heterogeneity in CRC. Currently, several gene signatures have been reported for prognostic prediction of CRC [63–66]. Compared to the reported signatures, the uniqueness of this study was that LASSO regression analysis could execute feature selection and shrinkage and screen highly correlated genes, which determined the optimal genes to participate in subsequent signature building [66]. LASSO regression could prevent the gene signature overfitting and increase the accuracy of bioinformatics analysis [67]. We explored both ROC curve and test verification to assess the prognostic performance of the signature. In the future, the value of the eight-gene signature still needs to be examined in clinical guidelines. The eight-gene signature could delaminate the risk of CRC patients' survival before surgery selection, which implied patients' benefit from therapy with a good prognosis and avoiding unnecessary treatment with a poor prognosis.

Finally, the genes of the signature were more or less researched in human tumors. A monoclonal antibody targeting anti-mullerian-hormone-receptor II (AMHRII) acted through tumor-associated macrophage engagement in advanced/metastatic CRC and had been performed phase 2 study [68]. WBSCR28 had not been well studied in human tumor, but it was repressed by androgen receptor in prostate cancer [69]. SFTA2 was identified as a potential disease-free survival prognostic gene in colon cancer and one of the potential biomarkers for distinguishing between lung adenocarcinoma and squamous cell carcinoma [70, 71]. MYH2 was confirmed significantly changed in hepatocellular carcinoma and highly expressed in the origin of squamous cell carcinoma in the lungs of patients with previous head and neck malignancies [72, 73]. POU4F1 was upregulated and induced neuroendocrine phenotype in small cell lung cancer [74]. SIX4 promoted tumor angiogenesis and metastasis via activating AKT pathway in CRC [75, 76]. PGPEP1L was confirmed downregulated in CRC via Expression Atlas database and firstly proposed as an independent prognostic factor (Table S14). PAX5 was identified to be relevant to CRC with peritoneal metastasis [77].

## 5. Conclusion

In conclusion, we identified hub genes involved in the pathogenesis of CRC with the help of integrated bioinformatics analysis. We also proposed an eight-gene signature comprising AMH, WBSCR28, SFTA2, MYH2, POU4F1, SIX4, PGPEP1L, and PAX5, which would provide directive significance for prognostic prediction and treatment selection in CRC. However, the application of the eight-gene signature still needed to be assessed and validated in clinical.

## Figures and Tables

**Table 1:** Information for six GEO datasets in the study.

Dataset	Platform	Number of samples (tumor/control)
GSE21510	[HG-U133_Plus_2] Affymetrix Human Genome U133 Plus 2.0 Array	148 (104/44)
GSE24514	[HG-U133A] Affymetrix Human Genome U133A Array	49 (34/15)
GSE32323	[HG-U133_Plus_2] Affymetrix Human Genome U133 Plus 2.0 Array	44 (22/22)
GSE89076	Agilent-039494 SurePrint G3 Human GE v2 8x60K Microarray 039381	80 (41/39)
GSE110225	[HG-U133A] Affymetrix Human Genome U133A Array; [HG-U133_Plus_2] Affymetrix Human Genome U133 Plus 2.0 Array	60 (30/30)
GSE113513	[PrimeView] Affymetrix Human Gene Expression Array	28 (14/14)

**Table 2:** Prognostic information for the eight genes in train group.

Gene symbol	Univariate analysis		Multivariate analysis		
	HR (95% CI)	*P* value	HR (95% CI)	*P* value	Coefficient
AMH	1.001 (1.000-1.02)	0.000297	1.001 (1.000-1.001)	0.011546	0.000842
WBSCR28	1.022 (1.010-1.033)	0.000139	1.012 (0.999-1.026)	0.080719	0.012188
SFTA2	1.001 (1.001-1.002)	1.61*E*-05	1.001 (1.001-1.002)	0.000137	0.001245
MYH2	1.061 (1.029-1.095)	0.000162	1.067 (1.027-1.108)	0.00076	0.064845
POU4F1	1.005 (1.003-1.008)	5.65*E*-05	1.004 (1.002-1.007)	0.002323	0.004278
SIX4	1.003 (1.002-1.004)	6.33*E*-07	1.003 (1.002-1.005)	1.79*E*-05	0.003124
PGPEP1L	1.061 (1.032-1.090)	2.46*E*-05	1.070 (1.038-1.103)	1.43*E*-05	0.067637
PAX5	1.001 (1.000-1.001)	1.53*E*-05	1.001 (1.000-1.001)	0.000106	0.000774

## Data Availability

The data used to support the findings of this study are available from the corresponding author upon request.

## Abbreviations

CRC: Colorectal cancer
TNM: Tumor-node-metastasis
DEGs: Differentially expressed genes
GEO: Gene Expression Omnibus
TCGA: The Cancer Genome Atlas
PPI: Protein-protein interaction
MTs: Metallothioneins
RRA: RobustRankAggreg
MCODE: Molecular Complex Detection
ROC: Receiver operating characteristic
AUC: Area under the curve
AMH: Muellerian-inhibiting factor; anti-mullerian hormone
WBSCR28: Transmembrane protein 270
SFTA2: Surfactant-associated protein 2
MYH2: Myosin-2
POU4F1: POU domain, class 4, transcription factor 1
SIX4: Homeobox protein SIX4
PGPEP1L: Pyroglutamyl-peptidase 1-like protein
PAX5: Paired box protein Pax-5.

## Supplementary Materials

Supplementary 1 Table S1: 254 DEGs screened by Limma package and integrated by RRA package from six GEO datasets.

Supplementary 2 Table S2: DEGs extracted from TCGA database.

Supplementary 3 Table S3: overlapping DEGs identified between six GEO datasets and TCGA database.

Supplementary 4 Table S4: the clinical information of patients organized for survival analysis.

Supplementary 5 Table S5: the biological processes analyzed for overlapping DEGs.

Supplementary 6 Table S6: the molecular functions analyzed for overlapping DEGs.

Supplementary 7 Table S7: the reactome pathway enriched for upregulated overlapping DEGs.

Supplementary 8 Table S8: the reactome pathway enriched for downregulated overlapping DEGs.

Supplementary 9 Table S9: the protein-protein interactions among overlapping DEGs identified with combined score ≥ 0.4.

Supplementary 10 Table S10: three topological parameters calculated for module 1.

Supplementary 11 Table S11: three topological parameters calculated for module 2.

Supplementary 12 Table S12: the univariate Cox proportional hazards regression analysis for train group.

Supplementary 13 Table S13: the LASSO penalized regression performed for train group.

Supplementary 14 Table S14: differential expression of PGPEP1L in human.
