# Supplementary material for: Identification of Hub Genes Related to Carcinogenesis and Prognosis in Colorectal Cancer Based on Integrated Bioinformatics
Source: Mediators Inflamm. 2020 Apr 9;2020:5934821. doi: 10.1155/2020/5934821 (PMC7171686; doi:10.1155/2020/5934821)
Supplement: Supplementary 1 — Table S1: 254 DEGs screened by Limma package and integrated by RRA package from six GEO datasets. [file 5934821.f1.docx]

| Name | Pvalue | adjPvalue | logFC |
| --- | --- | --- | --- |
| CXCL3 | 6.21E-21 | 2.34E-16 | 2.8212 |
| MMP3 | 1.07E-20 | 4.03E-16 | 3.134236 |
| CDH3 | 1.51E-20 | 5.68E-16 | 2.834913 |
| MMP1 | 8.73E-19 | 3.29E-14 | 3.127169 |
| FOXQ1 | 6.08E-18 | 2.29E-13 | 3.44834 |
| MMP7 | 6.67E-18 | 2.51E-13 | 3.323702 |
| CXCL1 | 1.12E-17 | 4.22E-13 | 2.378574 |
| KRT23 | 1.85E-17 | 6.96E-13 | 2.992509 |
| TRIP13 | 2.79E-17 | 1.05E-12 | 1.879165 |
| NFE2L3 | 3.49E-17 | 1.31E-12 | 2.027644 |
| TGFBI | 5.63E-17 | 2.12E-12 | 2.21245 |
| VSNL1 | 2.09E-16 | 7.86E-12 | 2.126879 |
| COL11A1 | 7.26E-16 | 2.73E-11 | 2.058855 |
| DPEP1 | 8.03E-16 | 3.02E-11 | 2.705048 |
| CDK1 | 1.10E-15 | 4.15E-11 | 1.675794 |
| TPX2 | 2.37E-15 | 8.91E-11 | 1.838171 |
| UBE2C | 2.44E-15 | 9.18E-11 | 1.662836 |
| LGR5 | 2.51E-15 | 9.46E-11 | 2.024853 |
| CEP55 | 2.67E-15 | 1.00E-10 | 1.79213 |
| EPHX4 | 4.81E-15 | 1.81E-10 | 2.299589 |
| SLCO1B3 | 8.83E-15 | 3.32E-10 | 1.684702 |
| PSAT1 | 9.28E-15 | 3.49E-10 | 1.509553 |
| REG1A | 1.00E-14 | 3.77E-10 | 2.612816 |
| MMP12 | 2.30E-14 | 8.67E-10 | 2.001818 |
| SOX9 | 3.16E-14 | 1.19E-09 | 1.660267 |
| INHBA | 3.23E-14 | 1.22E-09 | 2.205863 |
| CSE1L | 4.13E-14 | 1.55E-09 | 1.563229 |
| PUS7 | 4.29E-14 | 1.62E-09 | 1.608438 |
| SLC7A5 | 4.47E-14 | 1.68E-09 | 1.955768 |
| ATAD2 | 4.83E-14 | 1.82E-09 | 1.539424 |
| CDKN3 | 4.93E-14 | 1.86E-09 | 1.589809 |
| CKS2 | 5.33E-14 | 2.01E-09 | 1.84381 |
| NMU | 7.49E-14 | 2.82E-09 | 1.509498 |
| DLGAP5 | 8.05E-14 | 3.03E-09 | 1.586454 |
| PPAT | 9.14E-14 | 3.44E-09 | 1.61659 |
| RAD54B | 9.82E-14 | 3.69E-09 | 1.733486 |
| ASPM | 1.21E-13 | 4.56E-09 | 1.507396 |
| CLDN1 | 1.54E-13 | 5.79E-09 | 2.348221 |
| ENC1 | 1.69E-13 | 6.37E-09 | 1.51146 |
| PAICS | 1.78E-13 | 6.69E-09 | 1.668806 |
| CCNB1 | 2.37E-13 | 8.92E-09 | 1.657949 |
| TOP2A | 4.63E-13 | 1.74E-08 | 1.806409 |
| PMAIP1 | 4.76E-13 | 1.79E-08 | 1.638605 |
| CRNDE | 7.38E-13 | 2.78E-08 | 2.021502 |
| TTK | 7.89E-13 | 2.97E-08 | 1.630346 |
| CEMIP | 1.17E-12 | 4.40E-08 | 1.5272 |
| TCN1 | 1.71E-12 | 6.42E-08 | 2.083524 |
| IFITM1 | 1.87E-12 | 7.06E-08 | 1.602359 |
| CXCL11 | 4.08E-12 | 1.54E-07 | 1.993361 |
| RRM2 | 4.26E-12 | 1.60E-07 | 1.639702 |
| CXCL10 | 4.87E-12 | 1.83E-07 | 1.647233 |
| PPBP | 7.57E-12 | 2.85E-07 | 1.657354 |
| GTF2IRD1 | 7.58E-12 | 2.85E-07 | 1.590841 |
| EGFL6 | 8.10E-12 | 3.05E-07 | 1.779307 |
| C2CD4A | 1.08E-11 | 4.07E-07 | 1.956612 |
| CXCL8 | 1.28E-11 | 4.81E-07 | 1.538146 |
| SPP1 | 2.05E-11 | 7.73E-07 | 1.733379 |
| ECT2 | 2.06E-11 | 7.76E-07 | 1.515848 |
| REG3A | 2.46E-11 | 9.26E-07 | 1.766306 |
| ANLN | 2.72E-11 | 1.02E-06 | 1.682652 |
| REG1B | 2.74E-11 | 1.03E-06 | 1.884765 |
| S100P | 5.48E-11 | 2.06E-06 | 1.63241 |
| MYC | 5.67E-11 | 2.14E-06 | 1.700488 |
| SLC35D3 | 7.75E-11 | 2.92E-06 | 1.758192 |
| CLDN2 | 9.68E-11 | 3.64E-06 | 2.088925 |
| SERPINB5 | 9.68E-11 | 3.64E-06 | 1.663891 |
| LRP8 | 1.01E-10 | 3.79E-06 | 1.79189 |
| CYP4X1 | 1.16E-10 | 4.35E-06 | 1.541912 |
| CTHRC1 | 1.47E-10 | 5.53E-06 | 1.904423 |
| TDGF1 | 1.52E-10 | 5.71E-06 | 1.704227 |
| GZMB | 2.03E-10 | 7.65E-06 | 1.501432 |
| KLK6 | 2.37E-10 | 8.93E-06 | 1.535578 |
| CHI3L1 | 2.51E-10 | 9.44E-06 | 1.66572 |
| TMPRSS3 | 2.51E-10 | 9.46E-06 | 1.512547 |
| CKMT2 | 7.87E-10 | 2.96E-05 | 1.739169 |
| KRT6B | 2.46E-09 | 9.27E-05 | 1.515627 |
| TESC | 2.86E-09 | 0.000108 | 1.549023 |
| ASCL2 | 8.33E-09 | 0.000314 | 1.730121 |
| ETV4 | 2.31E-08 | 0.000869 | 1.521021 |
| KRT80 | 1.61E-07 | 0.006041 | 1.660053 |
| CLCA4 | 1.37E-25 | 5.16E-21 | -5.0738 |
| ZG16 | 1.27E-24 | 4.80E-20 | -4.54302 |
| GUCA2A | 2.34E-24 | 8.82E-20 | -4.28577 |
| MS4A12 | 1.12E-23 | 4.21E-19 | -4.74444 |
| GUCA2B | 1.18E-22 | 4.43E-18 | -4.14184 |
| CA4 | 8.83E-22 | 3.32E-17 | -4.24091 |
| AQP8 | 9.16E-21 | 3.45E-16 | -4.70607 |
| CHP2 | 5.11E-20 | 1.92E-15 | -3.11678 |
| ADH1C | 3.96E-19 | 1.49E-14 | -2.98039 |
| MT1M | 4.87E-19 | 1.83E-14 | -3.31285 |
| SCNN1B | 1.26E-18 | 4.73E-14 | -2.83496 |
| CD177 | 1.50E-18 | 5.63E-14 | -3.37246 |
| HSD17B2 | 1.50E-18 | 5.63E-14 | -3.0853 |
| DHRS9 | 2.28E-18 | 8.57E-14 | -2.92459 |
| CLDN8 | 3.29E-18 | 1.24E-13 | -3.66491 |
| CA2 | 3.29E-18 | 1.24E-13 | -3.72039 |
| CLEC3B | 3.38E-18 | 1.27E-13 | -2.463 |
| BTNL8 | 5.68E-18 | 2.14E-13 | -2.43258 |
| AKR1B10 | 8.61E-18 | 3.24E-13 | -2.77928 |
| HPGD | 1.12E-17 | 4.22E-13 | -2.52436 |
| LRRC19 | 1.27E-17 | 4.80E-13 | -2.50519 |
| PCK1 | 3.49E-17 | 1.31E-12 | -2.63891 |
| CA1 | 3.68E-17 | 1.39E-12 | -3.47503 |
| GCG | 8.00E-17 | 3.01E-12 | -3.08379 |
| CHGA | 9.70E-17 | 3.65E-12 | -3.0118 |
| ABCG2 | 1.63E-16 | 6.14E-12 | -3.28537 |
| CFD | 2.91E-16 | 1.10E-11 | -2.27917 |
| CLCA1 | 3.03E-16 | 1.14E-11 | -2.75768 |
| TSPAN7 | 3.85E-16 | 1.45E-11 | -2.16877 |
| FCGBP | 6.77E-16 | 2.55E-11 | -2.64916 |
| SLC26A3 | 7.53E-16 | 2.84E-11 | -2.94906 |
| SLC26A2 | 1.08E-15 | 4.05E-11 | -3.16751 |
| HMGCS2 | 1.14E-15 | 4.29E-11 | -2.25707 |
| NR3C2 | 1.22E-15 | 4.58E-11 | -2.21254 |
| SRPX | 1.58E-15 | 5.95E-11 | -2.07003 |
| LGALS2 | 1.63E-15 | 6.14E-11 | -2.40404 |
| ADH1B | 2.44E-15 | 9.18E-11 | -2.25235 |
| UGT1A1 | 3.59E-15 | 1.35E-10 | -1.98571 |
| SI | 5.85E-15 | 2.20E-10 | -2.50949 |
| CXCL12 | 7.59E-15 | 2.85E-10 | -2.15397 |
| BEST2 | 7.78E-15 | 2.93E-10 | -2.10586 |
| AHCYL2 | 8.19E-15 | 3.08E-10 | -1.73435 |
| GBA3 | 9.99E-15 | 3.76E-10 | -2.38943 |
| CKB | 1.13E-14 | 4.25E-10 | -2.0318 |
| HSD11B2 | 1.24E-14 | 4.67E-10 | -2.10316 |
| INSL5 | 1.43E-14 | 5.39E-10 | -2.41684 |
| CA7 | 1.50E-14 | 5.64E-10 | -2.35783 |
| SLC4A4 | 1.76E-14 | 6.62E-10 | -2.42446 |
| GDPD3 | 2.06E-14 | 7.75E-10 | -1.77529 |
| KRT20 | 2.40E-14 | 9.03E-10 | -2.05293 |
| BCAS1 | 2.45E-14 | 9.23E-10 | -1.67667 |
| CEACAM7 | 2.62E-14 | 9.84E-10 | -2.92133 |
| ENTPD5 | 3.10E-14 | 1.17E-09 | -1.8328 |
| CWH43 | 3.58E-14 | 1.35E-09 | -2.35765 |
| PLAC8 | 3.81E-14 | 1.43E-09 | -2.43154 |
| KLF4 | 4.04E-14 | 1.52E-09 | -2.10086 |
| MEP1A | 4.88E-14 | 1.84E-09 | -2.322 |
| DPT | 5.03E-14 | 1.89E-09 | -2.13831 |
| PPAP2A | 5.65E-14 | 2.13E-09 | -1.70594 |
| CDHR5 | 6.09E-14 | 2.29E-09 | -1.93442 |
| GPX3 | 6.82E-14 | 2.57E-09 | -1.54763 |
| SGK1 | 6.88E-14 | 2.59E-09 | -2.17869 |
| DHRS11 | 7.35E-14 | 2.77E-09 | -2.08068 |
| CLU | 7.49E-14 | 2.82E-09 | -1.9885 |
| EPB41L3 | 7.77E-14 | 2.92E-09 | -2.17284 |
| ADAMDEC1 | 8.29E-14 | 3.12E-09 | -2.02348 |
| SGK2 | 8.66E-14 | 3.26E-09 | -1.51596 |
| MALL | 9.47E-14 | 3.57E-09 | -2.0097 |
| STMN2 | 1.23E-13 | 4.63E-09 | -1.90261 |
| IGJ | 1.53E-13 | 5.77E-09 | -2.1692 |
| ARL14 | 1.72E-13 | 6.48E-09 | -2.01011 |
| CA12 | 2.06E-13 | 7.74E-09 | -2.10231 |
| CEACAM1 | 2.23E-13 | 8.38E-09 | -1.7561 |
| BTNL3 | 2.56E-13 | 9.64E-09 | -1.77203 |
| CDHR2 | 2.77E-13 | 1.04E-08 | -1.68494 |
| MFAP5 | 2.94E-13 | 1.11E-08 | -1.66551 |
| SPINK5 | 3.08E-13 | 1.16E-08 | -1.9861 |
| CES2 | 3.12E-13 | 1.18E-08 | -1.64035 |
| TUBAL3 | 3.95E-13 | 1.49E-08 | -2.0205 |
| BCHE | 3.95E-13 | 1.49E-08 | -1.5989 |
| GPA33 | 4.25E-13 | 1.60E-08 | -1.53208 |
| PKIB | 4.45E-13 | 1.68E-08 | -2.52851 |
| MAOA | 4.70E-13 | 1.77E-08 | -1.50944 |
| CNN1 | 5.11E-13 | 1.92E-08 | -1.94258 |
| ITM2C | 5.18E-13 | 1.95E-08 | -1.79619 |
| VIP | 5.71E-13 | 2.15E-08 | -2.23527 |
| RETSAT | 5.79E-13 | 2.18E-08 | -1.51621 |
| SST | 5.87E-13 | 2.21E-08 | -2.35349 |
| APPL2 | 6.73E-13 | 2.53E-08 | -1.52535 |
| PAPSS2 | 7.48E-13 | 2.82E-08 | -1.64781 |
| BEST4 | 9.77E-13 | 3.68E-08 | -2.64195 |
| PDE9A | 1.13E-12 | 4.25E-08 | -1.96969 |
| BMP2 | 1.25E-12 | 4.70E-08 | -1.66818 |
| C2orf88 | 1.27E-12 | 4.80E-08 | -2.25373 |
| GCNT3 | 1.69E-12 | 6.35E-08 | -1.81123 |
| PLP1 | 1.79E-12 | 6.73E-08 | -1.72511 |
| SELENBP1 | 2.13E-12 | 8.01E-08 | -1.90184 |
| MT1F | 2.49E-12 | 9.38E-08 | -1.94732 |
| FGL2 | 2.72E-12 | 1.03E-07 | -1.56796 |
| TSPAN1 | 2.74E-12 | 1.03E-07 | -1.93963 |
| FABP1 | 2.78E-12 | 1.05E-07 | -2.02289 |
| EDN3 | 3.01E-12 | 1.13E-07 | -1.73711 |
| UGT2A3 | 3.28E-12 | 1.23E-07 | -2.04679 |
| MEP1B | 3.46E-12 | 1.30E-07 | -1.69973 |
| SPIB | 4.08E-12 | 1.54E-07 | -1.73199 |
| DEFB1 | 5.02E-12 | 1.89E-07 | -1.55183 |
| SCGN | 5.28E-12 | 1.99E-07 | -1.81391 |
| CHRDL1 | 5.54E-12 | 2.09E-07 | -1.94723 |
| GHR | 8.49E-12 | 3.20E-07 | -1.70743 |
| ABCB1 | 8.81E-12 | 3.32E-07 | -1.72348 |
| HHLA2 | 8.90E-12 | 3.35E-07 | -1.57014 |
| SEPP1 | 9.32E-12 | 3.51E-07 | -1.75079 |
| PIGZ | 9.41E-12 | 3.54E-07 | -1.6203 |
| NR1H4 | 1.05E-11 | 3.95E-07 | -1.54408 |
| SLCO2A1 | 1.18E-11 | 4.44E-07 | -1.53374 |
| TRPM6 | 1.25E-11 | 4.70E-07 | -1.84273 |
| CHST5 | 1.45E-11 | 5.44E-07 | -1.72843 |
| SECTM1 | 1.51E-11 | 5.68E-07 | -1.67191 |
| SYNM | 1.51E-11 | 5.68E-07 | -2.05261 |
| SLC17A4 | 1.85E-11 | 6.97E-07 | -1.80102 |
| PTPRH | 2.99E-11 | 1.12E-06 | -1.64633 |
| MT1H | 3.08E-11 | 1.16E-06 | -2.0019 |
| C1orf115 | 3.30E-11 | 1.24E-06 | -1.60677 |
| RUNDC3B | 3.48E-11 | 1.31E-06 | -1.7793 |
| CLDN23 | 3.73E-11 | 1.40E-06 | -1.99997 |
| NXPE4 | 3.73E-11 | 1.40E-06 | -1.70318 |
| OGN | 4.24E-11 | 1.60E-06 | -2.25887 |
| KRT24 | 4.31E-11 | 1.62E-06 | -1.51353 |
| SLC30A10 | 4.40E-11 | 1.66E-06 | -2.60189 |
| CCL19 | 4.57E-11 | 1.72E-06 | -1.5291 |
| GREM2 | 4.67E-11 | 1.76E-06 | -1.70165 |
| VSIG2 | 6.39E-11 | 2.41E-06 | -1.80672 |
| MUC2 | 9.68E-11 | 3.64E-06 | -2.04052 |
| SMPDL3A | 9.84E-11 | 3.70E-06 | -1.75277 |
| MT1E | 1.27E-10 | 4.80E-06 | -1.64933 |
| HEPACAM2 | 1.47E-10 | 5.53E-06 | -1.89672 |
| CDKN2B | 1.62E-10 | 6.09E-06 | -1.66134 |
| UGT2B17 | 1.89E-10 | 7.10E-06 | -1.84805 |
| TEX11 | 1.98E-10 | 7.43E-06 | -1.7457 |
| CILP | 2.07E-10 | 7.80E-06 | -1.60214 |
| LDHD | 2.66E-10 | 1.00E-05 | -1.55212 |
| CCDC68 | 4.04E-10 | 1.52E-05 | -1.51171 |
| SCGB2A1 | 4.46E-10 | 1.68E-05 | -1.55299 |
| PYY | 5.44E-10 | 2.05E-05 | -1.58509 |
| TMEM100 | 5.57E-10 | 2.10E-05 | -1.87014 |
| IGH | 5.58E-10 | 2.10E-05 | -1.51909 |
| MT1G | 7.11E-10 | 2.67E-05 | -1.63027 |
| ITLN1 | 7.33E-10 | 2.76E-05 | -1.65362 |
| CCL23 | 9.07E-10 | 3.42E-05 | -1.55069 |
| C2orf40 | 9.93E-10 | 3.74E-05 | -1.91059 |
| ACTG2 | 1.03E-09 | 3.87E-05 | -1.64138 |
| SCG2 | 1.32E-09 | 4.97E-05 | -1.53336 |
| AGPAT9 | 1.35E-09 | 5.09E-05 | -1.76895 |
| PCOLCE2 | 1.40E-09 | 5.27E-05 | -1.74209 |
| TNFRSF17 | 1.47E-09 | 5.53E-05 | -1.87215 |
| MT1X | 1.52E-09 | 5.74E-05 | -1.60707 |
| PADI2 | 1.70E-09 | 6.41E-05 | -1.51621 |
| CXCL13 | 2.37E-09 | 8.94E-05 | -1.62348 |
| ANPEP | 3.27E-09 | 0.000123 | -1.78508 |
| IL1R2 | 5.41E-09 | 0.000204 | -1.59083 |
| PDE6A | 5.47E-09 | 0.000206 | -1.57247 |
| MYH11 | 6.05E-09 | 0.000228 | -1.84209 |
| MAMDC2 | 6.74E-09 | 0.000254 | -1.59915 |
| ANO5 | 8.58E-09 | 0.000323 | -1.50877 |
| SPON1 | 9.78E-09 | 0.000368 | -1.50128 |
| TP53INP2 | 1.46E-08 | 0.000549 | -1.56524 |
| CNTN3 | 1.70E-08 | 0.000641 | -1.64148 |
| SCARA5 | 1.71E-08 | 0.000644 | -1.61356 |
| IL6R | 1.79E-08 | 0.000673 | -1.5944 |
| SLC16A9 | 4.42E-08 | 0.001663 | -1.50206 |
| LAMA1 | 5.66E-08 | 0.002129 | -1.51354 |
| MUC4 | 9.22E-08 | 0.003469 | -1.54103 |
| C11orf86 | 3.37E-07 | 0.012671 | -1.55352 |
| TMIGD1 | 9.34E-07 | 0.035141 | -1.72807 |
