# Supplementary material for: Identification of Hub Genes Related to Carcinogenesis and Prognosis in Colorectal Cancer Based on Integrated Bioinformatics
Source: Mediators Inflamm. 2020 Apr 9;2020:5934821. doi: 10.1155/2020/5934821 (PMC7171686; doi:10.1155/2020/5934821)
Supplement: Supplementary 2 — Table S2: DEGs extracted from TCGA database. [file 5934821.f2.docx]

| gene | conMean | treatMean | logFC | pValue | fdr |
| --- | --- | --- | --- | --- | --- |
| OTOP2 | 4738.392 | 12.03864 | -8.62058 | 9.12E-35 | 2.63E-32 |
| MS4A10 | 513.1961 | 1.896445 | -8.08007 | 6.33E-32 | 8.26E-30 |
| SLC10A2 | 818.7255 | 3.335394 | -7.93938 | 3.30E-37 | 1.54E-34 |
| AQP8 | 53194.22 | 361.0742 | -7.20283 | 2.37E-30 | 1.38E-28 |
| CA1 | 55595.35 | 506.3849 | -6.77859 | 2.45E-31 | 2.28E-29 |
| GUCA2B | 5471.549 | 51.18238 | -6.74016 | 2.43E-32 | 4.06E-30 |
| CPO | 406.1569 | 4.27357 | -6.57045 | 1.42E-10 | 4.00E-10 |
| OTOP3 | 142.3529 | 1.642968 | -6.43702 | 6.31E-47 | 1.27E-43 |
| SLC13A1 | 128.2745 | 1.506955 | -6.41145 | 6.71E-33 | 1.40E-30 |
| TMIGD1 | 6199.059 | 73.46832 | -6.39878 | 1.06E-32 | 2.03E-30 |
| PYY | 4892.255 | 58.84235 | -6.3775 | 1.96E-32 | 3.34E-30 |
| BEST4 | 4215.706 | 51.20711 | -6.36329 | 1.29E-32 | 2.38E-30 |
| INSL5 | 2450.725 | 32.34312 | -6.24361 | 1.75E-25 | 2.80E-24 |
| CD177P1 | 281.451 | 3.763524 | -6.22466 | 1.70E-33 | 3.81E-31 |
| CA7 | 5448.784 | 78.58578 | -6.11552 | 1.90E-32 | 3.31E-30 |
| MEP1B | 2055.02 | 30.34776 | -6.08142 | 5.67E-27 | 1.22E-25 |
| HSD3B2 | 1068.765 | 15.98918 | -6.0627 | 4.74E-27 | 1.04E-25 |
| SLC6A19 | 4462.176 | 70.0541 | -5.99313 | 1.74E-28 | 5.43E-27 |
| RP11-579E24.2 | 9.784314 | 0.160742 | -5.92765 | 1.03E-29 | 4.60E-28 |
| KRT24 | 218.549 | 3.636785 | -5.90915 | 3.24E-51 | 1.57E-47 |
| SST | 1053.471 | 17.56105 | -5.90663 | 3.89E-32 | 5.66E-30 |
| XXyac-YM21GA2.7 | 63.23529 | 1.132921 | -5.80261 | 9.59E-39 | 6.11E-36 |
| PLP1 | 1566.118 | 28.39413 | -5.78546 | 2.55E-32 | 4.12E-30 |
| SLC30A10 | 1015 | 18.59505 | -5.77042 | 4.60E-32 | 6.33E-30 |
| DHRS7C | 28.09804 | 0.51932 | -5.7577 | 1.14E-45 | 1.53E-42 |
| MS4A12 | 20060.59 | 376.2519 | -5.73652 | 1.76E-30 | 1.11E-28 |
| SLC17A8 | 115.8039 | 2.20711 | -5.71338 | 9.11E-37 | 3.62E-34 |
| RP11-807H22.5 | 74.58824 | 1.423493 | -5.71144 | 1.39E-38 | 8.63E-36 |
| CLCA4 | 55243.71 | 1118.354 | -5.62636 | 3.72E-31 | 3.19E-29 |
| KRTAP13-2 | 197.9412 | 4.032457 | -5.61727 | 3.04E-32 | 4.72E-30 |
| GUCA2A | 28563.16 | 588.9907 | -5.59977 | 3.95E-32 | 5.66E-30 |
| VSTM2A | 333.8824 | 6.896445 | -5.59734 | 1.03E-33 | 2.41E-31 |
| CD177 | 29911.43 | 659.7759 | -5.50258 | 1.55E-28 | 4.90E-27 |
| LGI1 | 114.5098 | 2.550232 | -5.4887 | 6.33E-37 | 2.69E-34 |
| MGAM | 1910.765 | 44.22566 | -5.43312 | 1.53E-17 | 8.35E-17 |
| BMP3 | 3180.098 | 74.22875 | -5.42095 | 2.30E-32 | 3.90E-30 |
| RERGL | 236.4902 | 5.686244 | -5.37816 | 1.42E-33 | 3.27E-31 |
| CUBN | 1233.49 | 29.80216 | -5.37119 | 3.65E-13 | 1.30E-12 |
| CDKN2B-AS1 | 2398.118 | 58.29366 | -5.36242 | 4.00E-32 | 5.70E-30 |
| HSD3BP2 | 24.41176 | 0.598145 | -5.35094 | 1.53E-30 | 1.00E-28 |
| CLDN8 | 3314.176 | 81.36476 | -5.3481 | 4.04E-31 | 3.44E-29 |
| DPP6 | 634.2745 | 15.6136 | -5.34423 | 2.85E-29 | 1.10E-27 |
| CA4 | 21508.65 | 542.6832 | -5.30866 | 1.37E-31 | 1.48E-29 |
| MT1CP | 8.960784 | 0.227202 | -5.30157 | 2.70E-50 | 1.09E-46 |
| SLC25A47P1 | 12.29412 | 0.313756 | -5.29218 | 3.41E-46 | 5.51E-43 |
| CA2 | 67104.35 | 1769.546 | -5.24496 | 6.78E-31 | 5.35E-29 |
| NGB | 82.47059 | 2.213292 | -5.21961 | 2.82E-37 | 1.34E-34 |
| GBA3 | 1385.137 | 37.60742 | -5.20287 | 1.02E-30 | 7.34E-29 |
| SCGN | 855.5686 | 23.27357 | -5.20012 | 1.26E-32 | 2.35E-30 |
| NPTX1 | 1612.235 | 43.93663 | -5.19749 | 1.13E-25 | 1.86E-24 |
| RP11-489D6.2 | 6.882353 | 0.188563 | -5.18979 | 3.12E-44 | 3.15E-41 |
| RP11-209E8.1 | 57.23529 | 1.601236 | -5.15965 | 1.35E-32 | 2.45E-30 |
| MORN5 | 217.7255 | 6.139104 | -5.14834 | 1.25E-18 | 7.65E-18 |
| DUSP21 | 5.72549 | 0.162287 | -5.14078 | 1.74E-49 | 5.26E-46 |
| SCN7A | 1660.627 | 47.11283 | -5.13946 | 1.03E-30 | 7.41E-29 |
| LINC00974 | 74.33333 | 2.11592 | -5.13465 | 1.27E-36 | 4.90E-34 |
| RP11-521D12.4 | 9.352941 | 0.267388 | -5.12841 | 7.81E-39 | 5.11E-36 |
| CHAT | 31.5098 | 0.907264 | -5.11813 | 5.10E-42 | 4.26E-39 |
| DAO | 196.9216 | 5.704791 | -5.1093 | 6.35E-32 | 8.26E-30 |
| PGM5-AS1 | 76.67647 | 2.23493 | -5.10048 | 2.24E-31 | 2.13E-29 |
| RN7SKP127 | 50.82353 | 1.497682 | -5.08469 | 4.64E-36 | 1.70E-33 |
| ABCG2 | 3862.961 | 113.8764 | -5.08417 | 1.79E-31 | 1.83E-29 |
| LINC00682 | 17.07843 | 0.50541 | -5.07858 | 3.81E-46 | 5.77E-43 |
| CEACAM20 | 107.3529 | 3.179289 | -5.07751 | 1.33E-07 | 2.85E-07 |
| RP11-135D11.2 | 8.313725 | 0.251932 | -5.04439 | 7.15E-57 | 8.65E-53 |
| ATP1A2 | 2314.824 | 70.21329 | -5.04301 | 4.58E-30 | 2.37E-28 |
| RBFOX3 | 877.6078 | 26.91345 | -5.02718 | 2.13E-19 | 1.41E-18 |
| CMTM5 | 54.21569 | 1.664606 | -5.02546 | 8.74E-35 | 2.58E-32 |
| SCARA5 | 4684.353 | 143.8779 | -5.02493 | 1.40E-32 | 2.50E-30 |
| RP11-389G6.3 | 29.58824 | 0.919629 | -5.00783 | 3.68E-32 | 5.40E-30 |
| PCSK2 | 322.6078 | 10.22257 | -4.97995 | 1.57E-33 | 3.55E-31 |
| NRXN1 | 509.9216 | 16.40031 | -4.95848 | 5.58E-32 | 7.43E-30 |
| ZG16 | 66752.65 | 2188.051 | -4.93111 | 2.67E-31 | 2.42E-29 |
| RP11-396O20.2 | 443.1569 | 14.52705 | -4.931 | 5.12E-30 | 2.59E-28 |
| SPIB | 2884.765 | 94.97527 | -4.92476 | 3.90E-32 | 5.66E-30 |
| PRIMA1 | 1062.667 | 35.10819 | -4.91974 | 1.34E-31 | 1.46E-29 |
| MYOC | 363.5294 | 12.01236 | -4.91948 | 5.64E-35 | 1.73E-32 |
| HAND2-AS1 | 1113.059 | 36.95518 | -4.91261 | 5.21E-27 | 1.13E-25 |
| SLC26A3 | 186864 | 6219.964 | -4.90894 | 7.63E-32 | 9.19E-30 |
| ABCB11 | 249.9412 | 8.33694 | -4.90593 | 7.38E-30 | 3.48E-28 |
| MAMDC2 | 1445.784 | 48.39876 | -4.90074 | 4.23E-32 | 5.85E-30 |
| SLC4A4 | 14816.22 | 496.7032 | -4.89865 | 3.32E-32 | 5.13E-30 |
| ALPI | 2473.627 | 82.93663 | -4.89847 | 1.92E-31 | 1.93E-29 |
| FAM180B | 42.01961 | 1.415765 | -4.89141 | 7.88E-36 | 2.77E-33 |
| MT1M | 3123.176 | 105.881 | -4.8825 | 7.75E-31 | 5.88E-29 |
| FRMPD4 | 79.70588 | 2.712519 | -4.87698 | 2.93E-31 | 2.62E-29 |
| SMYD1 | 389.7255 | 13.45595 | -4.85614 | 1.57E-13 | 5.80E-13 |
| PPY | 38.82353 | 1.344668 | -4.85161 | 4.23E-32 | 5.85E-30 |
| RP11-167N24.6 | 21.15686 | 0.737249 | -4.84283 | 6.48E-26 | 1.12E-24 |
| NPY2R | 74.72549 | 2.608964 | -4.84005 | 7.40E-38 | 3.81E-35 |
| CHGA | 14793.92 | 518.2365 | -4.83525 | 7.35E-32 | 9.00E-30 |
| PHOX2B | 115.2353 | 4.089645 | -4.81646 | 3.03E-34 | 7.97E-32 |
| CADM3 | 1231.078 | 43.92736 | -4.80866 | 5.00E-32 | 6.76E-30 |
| GSTA2 | 159.2549 | 5.734158 | -4.79561 | 6.82E-13 | 2.38E-12 |
| AC007182.6 | 197.8235 | 7.153014 | -4.78952 | 7.03E-32 | 8.77E-30 |
| ASB5 | 247.6078 | 9.054096 | -4.77334 | 1.37E-27 | 3.40E-26 |
| CTD-2023N9.1 | 7.431373 | 0.27357 | -4.76365 | 3.55E-50 | 1.23E-46 |
| CADM2 | 197 | 7.309119 | -4.75235 | 2.52E-32 | 4.10E-30 |
| TMEM72 | 1208.51 | 46.5796 | -4.69739 | 3.45E-31 | 2.97E-29 |
| CTD-2385L22.2 | 140.9804 | 5.449768 | -4.69316 | 1.12E-32 | 2.13E-30 |
| VSTM2A-OT1 | 7.980392 | 0.31221 | -4.67587 | 4.94E-61 | 1.20E-56 |
| ABCA8 | 2050.608 | 81.07573 | -4.66064 | 2.48E-32 | 4.10E-30 |
| STMN4 | 66.72549 | 2.664606 | -4.64624 | 5.38E-34 | 1.37E-31 |
| KHDRBS2 | 37.09804 | 1.482226 | -4.64551 | 8.20E-37 | 3.31E-34 |
| ANGPTL7 | 217.0392 | 8.695518 | -4.64154 | 6.11E-31 | 4.88E-29 |
| FAM135B | 138.5686 | 5.619784 | -4.62394 | 9.60E-33 | 1.91E-30 |
| FIGF | 214.1765 | 8.768161 | -4.61038 | 1.31E-31 | 1.43E-29 |
| RSPO2 | 552.098 | 22.64606 | -4.60759 | 1.08E-31 | 1.22E-29 |
| SGCG | 49.68627 | 2.063369 | -4.58977 | 1.73E-34 | 4.70E-32 |
| RP1-35C21.1 | 55.17647 | 2.306028 | -4.58057 | 9.39E-31 | 6.89E-29 |
| GPM6A | 902.1373 | 37.85162 | -4.57492 | 5.17E-28 | 1.43E-26 |
| LINC00507 | 21.90196 | 0.935085 | -4.54982 | 5.74E-37 | 2.48E-34 |
| HS3ST6 | 53.98039 | 2.313756 | -4.54413 | 1.62E-27 | 3.95E-26 |
| UGT1A5 | 7.392157 | 0.319938 | -4.53013 | 5.16E-20 | 3.68E-19 |
| MT1H | 2409.216 | 105.0572 | -4.51932 | 8.73E-28 | 2.31E-26 |
| RP11-92A5.2 | 20.27451 | 0.885626 | -4.51683 | 9.00E-42 | 7.03E-39 |
| MYH11 | 255684.8 | 11230.73 | -4.50884 | 2.08E-26 | 3.96E-25 |
| XKR4 | 164.7451 | 7.2983 | -4.49653 | 1.46E-31 | 1.56E-29 |
| UGT2B17 | 26610.71 | 1181.376 | -4.49347 | 1.05E-18 | 6.48E-18 |
| UGT1A8 | 732.9608 | 32.57342 | -4.49197 | 1.82E-31 | 1.85E-29 |
| B4GALNT2 | 8048.02 | 358.3369 | -4.48925 | 7.53E-29 | 2.60E-27 |
| ADCYAP1R1 | 261.2353 | 11.64451 | -4.48763 | 1.39E-30 | 9.38E-29 |
| EPHA6 | 94.66667 | 4.239567 | -4.48087 | 4.92E-24 | 6.13E-23 |
| PI16 | 1216.471 | 54.5456 | -4.47909 | 4.11E-31 | 3.48E-29 |
| CEACAM7 | 141252.2 | 6353.751 | -4.47452 | 1.02E-29 | 4.58E-28 |
| CLEC3B | 2179.137 | 98.27975 | -4.47072 | 2.38E-32 | 4.00E-30 |
| RP11-2N1.2 | 24.17647 | 1.092736 | -4.46759 | 5.56E-34 | 1.39E-31 |
| CYP3A4 | 1764.275 | 79.80371 | -4.46648 | 1.29E-17 | 7.09E-17 |
| TEX11 | 286.4902 | 13.08655 | -4.45233 | 1.45E-30 | 9.68E-29 |
| SYNM | 21855.43 | 1011.181 | -4.43388 | 3.32E-24 | 4.24E-23 |
| SCNN1G | 641.9804 | 29.74498 | -4.43181 | 1.77E-22 | 1.73E-21 |
| TRPM6 | 6214.863 | 289.0479 | -4.42634 | 2.14E-30 | 1.30E-28 |
| RP11-467L13.4 | 23.84314 | 1.109737 | -4.42528 | 9.11E-35 | 2.63E-32 |
| RP11-855A2.5 | 19.54902 | 0.910355 | -4.42452 | 2.15E-36 | 8.12E-34 |
| MMP27 | 15.21569 | 0.710974 | -4.41962 | 2.49E-45 | 2.87E-42 |
| CDKN2B-AS | 14.78431 | 0.692427 | -4.41626 | 1.51E-38 | 8.91E-36 |
| TMEFF2 | 64.60784 | 3.03864 | -4.41021 | 3.84E-33 | 8.23E-31 |
| C2orf40 | 310.0588 | 14.6136 | -4.40716 | 5.94E-31 | 4.77E-29 |
| PMP2 | 72.58824 | 3.42813 | -4.40424 | 1.12E-34 | 3.11E-32 |
| GCG | 3247.078 | 153.442 | -4.40338 | 6.12E-32 | 8.05E-30 |
| MAS1L | 13.68627 | 0.64915 | -4.39803 | 6.17E-48 | 1.66E-44 |
| SLC5A7 | 84.39216 | 4.017002 | -4.39292 | 8.18E-32 | 9.72E-30 |
| AADACL2 | 21.39216 | 1.018547 | -4.3925 | 1.95E-47 | 4.72E-44 |
| POU3F4 | 14.96078 | 0.712519 | -4.39211 | 2.31E-25 | 3.63E-24 |
| LINC01289 | 8.294118 | 0.395672 | -4.38971 | 2.58E-32 | 4.14E-30 |
| RP11-1090M7.1 | 50.45098 | 2.409583 | -4.38803 | 2.61E-31 | 2.39E-29 |
| APOB | 8853.235 | 424.1716 | -4.38348 | 1.17E-10 | 3.33E-10 |
| SLC51A | 3013.961 | 145.153 | -4.37601 | 1.16E-23 | 1.36E-22 |
| PIRT | 264.2549 | 12.73261 | -4.37533 | 1.09E-31 | 1.22E-29 |
| AMPD1 | 254.4902 | 12.34158 | -4.36601 | 4.23E-31 | 3.57E-29 |
| BCHE | 819.549 | 40.06801 | -4.35431 | 2.45E-30 | 1.42E-28 |
| CDH19 | 448.0196 | 21.9459 | -4.35154 | 5.58E-30 | 2.78E-28 |
| ADH1C | 19243.82 | 948.7651 | -4.3422 | 1.01E-31 | 1.15E-29 |
| SCNN1B | 6467.294 | 318.8918 | -4.34202 | 2.06E-30 | 1.26E-28 |
| CDH10 | 17.39216 | 0.857805 | -4.34164 | 8.63E-42 | 6.97E-39 |
| RXRG | 104.6275 | 5.166924 | -4.33981 | 1.41E-32 | 2.50E-30 |
| LYVE1 | 2434.627 | 120.5054 | -4.33653 | 1.55E-31 | 1.65E-29 |
| PKIB | 6707.373 | 332.8037 | -4.333 | 3.41E-32 | 5.23E-30 |
| ANPEP | 83161.61 | 4133.776 | -4.33039 | 4.35E-28 | 1.23E-26 |
| RP11-434H14.1 | 19 | 0.950541 | -4.32111 | 1.02E-43 | 9.89E-41 |
| SLC26A2 | 91947.53 | 4606.861 | -4.31895 | 9.30E-30 | 4.24E-28 |
| SLC25A34 | 864.7647 | 43.41267 | -4.31612 | 3.66E-32 | 5.40E-30 |
| AC106869.2 | 47.60784 | 2.391036 | -4.31549 | 2.47E-33 | 5.44E-31 |
| CHP2 | 17231.41 | 865.4328 | -4.31548 | 1.58E-31 | 1.67E-29 |
| B3GALT5-AS1 | 610.9608 | 30.89645 | -4.30557 | 1.35E-25 | 2.21E-24 |
| SLC51B | 3628.118 | 183.8269 | -4.3028 | 3.68E-32 | 5.40E-30 |
| RP11-753A21.1 | 15.64706 | 0.802164 | -4.28585 | 9.79E-20 | 6.74E-19 |
| PDZRN4 | 755.6275 | 38.87326 | -4.28083 | 1.96E-25 | 3.12E-24 |
| TCEAL6 | 17.5098 | 0.901082 | -4.28036 | 1.95E-37 | 9.43E-35 |
| SFRP1 | 2746.451 | 141.4436 | -4.27927 | 7.13E-31 | 5.57E-29 |
| SULT1A2 | 970.0392 | 50.19165 | -4.27252 | 7.54E-32 | 9.13E-30 |
| GLP2R | 644.098 | 33.46986 | -4.26635 | 1.40E-32 | 2.50E-30 |
| DPT | 5869.647 | 305.5471 | -4.26381 | 1.85E-30 | 1.16E-28 |
| C11orf86 | 1813.275 | 95.21484 | -4.25127 | 4.80E-31 | 3.97E-29 |
| HMP19 | 285.7451 | 15.00464 | -4.25125 | 2.13E-31 | 2.07E-29 |
| JCHAIN | 217036.3 | 11407.37 | -4.2499 | 2.28E-30 | 1.35E-28 |
| ACTG2 | 92549.57 | 4943.468 | -4.22663 | 2.67E-22 | 2.55E-21 |
| RP11-420K14.2 | 9.254902 | 0.496136 | -4.22141 | 1.23E-18 | 7.55E-18 |
| FEV | 142.4314 | 7.639876 | -4.22057 | 1.34E-32 | 2.45E-30 |
| PRKG2 | 706.4314 | 37.89645 | -4.22041 | 8.55E-32 | 1.00E-29 |
| HAND1 | 729.9804 | 39.17311 | -4.21992 | 1.19E-14 | 4.87E-14 |
| P2RX2 | 51.23529 | 2.752705 | -4.21822 | 9.21E-33 | 1.87E-30 |
| ASPA | 342.3529 | 18.39413 | -4.21817 | 1.95E-32 | 3.34E-30 |
| TMEM35 | 979.8431 | 52.66615 | -4.2176 | 1.27E-28 | 4.07E-27 |
| CWH43 | 2122.627 | 114.2318 | -4.21581 | 6.07E-30 | 2.98E-28 |
| RP11-148L24.1 | 13.43137 | 0.723338 | -4.21479 | 2.03E-30 | 1.25E-28 |
| RP11-747D18.1 | 109.3137 | 5.893354 | -4.21324 | 3.17E-30 | 1.76E-28 |
| RP11-17M24.1 | 9 | 0.485317 | -4.21293 | 1.96E-45 | 2.37E-42 |
| CASQ2 | 1525.255 | 82.38331 | -4.21055 | 3.29E-26 | 6.07E-25 |
| ANGPTL1 | 1300.824 | 70.26275 | -4.21052 | 1.27E-29 | 5.54E-28 |
| CMA1 | 143.5294 | 7.763524 | -4.20849 | 6.49E-32 | 8.36E-30 |
| RP11-678G14.3 | 56.84314 | 3.075734 | -4.20798 | 9.51E-29 | 3.19E-27 |
| GPR119 | 9.72549 | 0.531685 | -4.19313 | 1.74E-45 | 2.22E-42 |
| AVPR1B | 14.45098 | 0.79289 | -4.1879 | 3.18E-40 | 2.27E-37 |
| MT1JP | 12.27451 | 0.673879 | -4.18703 | 1.20E-35 | 3.98E-33 |
| USP2 | 1800.02 | 99.21947 | -4.18125 | 1.81E-31 | 1.85E-29 |
| RP3-407E4.3 | 25.01961 | 1.386399 | -4.17364 | 8.18E-37 | 3.31E-34 |
| CNTN2 | 186.2941 | 10.39258 | -4.16396 | 1.14E-30 | 8.04E-29 |
| PGM5P4 | 28.19608 | 1.581144 | -4.15645 | 9.58E-33 | 1.91E-30 |
| SH2D7 | 286.2549 | 16.06955 | -4.1549 | 1.33E-30 | 9.11E-29 |
| CNN1 | 35061.39 | 1972.071 | -4.1521 | 3.85E-23 | 4.14E-22 |
| SLC5A12 | 348.9216 | 19.63679 | -4.15127 | 7.42E-16 | 3.42E-15 |
| TNXB | 5619.647 | 318.7682 | -4.1399 | 5.09E-30 | 2.59E-28 |
| SPHKAP | 13.84314 | 0.788253 | -4.13437 | 1.63E-38 | 9.39E-36 |
| CNTFR | 939.2549 | 53.60742 | -4.13101 | 1.25E-29 | 5.47E-28 |
| BEST2 | 1795.412 | 103.2658 | -4.11988 | 1.68E-29 | 7.04E-28 |
| LDB3 | 1091.843 | 62.8609 | -4.11846 | 9.82E-27 | 2.01E-25 |
| TACR2 | 2362.451 | 137.1236 | -4.10674 | 2.29E-23 | 2.55E-22 |
| CREB3L3 | 1433.647 | 83.37713 | -4.10389 | 1.11E-21 | 9.72E-21 |
| GSTA1 | 1323.059 | 77.01391 | -4.10261 | 5.78E-24 | 7.12E-23 |
| APOA1 | 7089.627 | 412.8423 | -4.10205 | 0.006035 | 0.007822 |
| PYGM | 724.7059 | 42.29675 | -4.09878 | 1.47E-28 | 4.66E-27 |
| NR1H4 | 737.8431 | 43.22411 | -4.09341 | 2.08E-24 | 2.75E-23 |
| OGN | 2280.765 | 133.9196 | -4.09008 | 2.23E-30 | 1.34E-28 |
| IGHA2 | 320720 | 18840.57 | -4.0894 | 1.16E-29 | 5.14E-28 |
| CNR1 | 421.451 | 24.90726 | -4.08073 | 1.35E-30 | 9.17E-29 |
| RP11-887P2.5 | 176.2353 | 10.43122 | -4.07852 | 2.03E-22 | 1.96E-21 |
| RP11-202A13.1 | 23.29412 | 1.38949 | -4.06734 | 8.16E-34 | 2.00E-31 |
| AL928768.3 | 110.1765 | 6.576507 | -4.06635 | 1.98E-30 | 1.23E-28 |
| LYPD8 | 19548.75 | 1170.32 | -4.0621 | 7.89E-30 | 3.70E-28 |
| HAND2 | 1411.431 | 84.63833 | -4.0597 | 2.15E-25 | 3.40E-24 |
| SERPINA9 | 96.5098 | 5.809892 | -4.05409 | 3.73E-19 | 2.41E-18 |
| PCDH11Y | 8.862745 | 0.534776 | -4.05075 | 2.68E-10 | 7.35E-10 |
| RP11-356O9.2 | 96.17647 | 5.803709 | -4.05064 | 2.67E-31 | 2.42E-29 |
| TMEM100 | 523.5294 | 31.59969 | -4.05029 | 3.51E-32 | 5.31E-30 |
| ADH1B | 6811.451 | 412.2674 | -4.04631 | 2.01E-31 | 1.99E-29 |
| RP4-659I19.1 | 11 | 0.669243 | -4.03883 | 2.53E-36 | 9.41E-34 |
| MAL | 286.0392 | 17.4575 | -4.03429 | 2.08E-31 | 2.05E-29 |
| RP4-811M8.1 | 47.01961 | 2.882535 | -4.02785 | 9.10E-30 | 4.16E-28 |
| AF001548.6 | 55.03922 | 3.378671 | -4.02593 | 2.39E-21 | 2.00E-20 |
| GCNT2 | 1107.765 | 68.09737 | -4.02391 | 2.96E-32 | 4.62E-30 |
| GALR1 | 31.54902 | 1.956723 | -4.01108 | 9.94E-34 | 2.35E-31 |
| C2orf88 | 5313.588 | 329.7187 | -4.01038 | 4.19E-32 | 5.85E-30 |
| KCNA1 | 50.21569 | 3.173107 | -3.98417 | 9.44E-22 | 8.30E-21 |
| ELANE | 39.82353 | 2.51932 | -3.98251 | 3.19E-33 | 6.89E-31 |
| MAPK4 | 256.1176 | 16.20711 | -3.98211 | 2.17E-27 | 5.08E-26 |
| GFRA2 | 396.8431 | 25.11901 | -3.98172 | 3.46E-32 | 5.27E-30 |
| ADAMDEC1 | 14172.98 | 897.2164 | -3.98154 | 2.21E-30 | 1.34E-28 |
| SORCS1 | 392.9412 | 24.95209 | -3.97708 | 3.42E-30 | 1.87E-28 |
| GREM2 | 2479.863 | 157.6646 | -3.97533 | 1.30E-31 | 1.43E-29 |
| SLC9A3 | 15717.12 | 1000.172 | -3.97402 | 2.12E-18 | 1.27E-17 |
| SPOCK3 | 74.7451 | 4.758887 | -3.97328 | 7.18E-33 | 1.49E-30 |
| RP3-422G23.4 | 22.7451 | 1.448223 | -3.9732 | 5.08E-36 | 1.84E-33 |
| GRIK3 | 437.2745 | 28.22411 | -3.95354 | 2.93E-30 | 1.64E-28 |
| IGHV3OR16-13 | 180 | 11.67079 | -3.94702 | 1.73E-27 | 4.20E-26 |
| IGHV3-7 | 545.5294 | 35.45286 | -3.94368 | 1.90E-28 | 5.86E-27 |
| MYOT | 140.2745 | 9.149923 | -3.93835 | 2.47E-31 | 2.29E-29 |
| CNGB1 | 46.5098 | 3.034003 | -3.93824 | 3.43E-31 | 2.97E-29 |
| SLC5A11 | 150.3725 | 9.84544 | -3.93294 | 6.72E-20 | 4.73E-19 |
| GPR15 | 534.0392 | 34.96909 | -3.93279 | 2.82E-30 | 1.61E-28 |
| PRPH | 374.7059 | 24.58423 | -3.92995 | 1.34E-30 | 9.16E-29 |
| PCP4L1 | 235.1569 | 15.50541 | -3.92278 | 1.55E-22 | 1.53E-21 |
| MUSK | 96.60784 | 6.372488 | -3.92221 | 6.83E-32 | 8.61E-30 |
| TRBV26OR9-2 | 4.333333 | 0.285935 | -3.92172 | 7.98E-30 | 3.74E-28 |
| TCEAL2 | 305.9608 | 20.20093 | -3.92085 | 1.39E-26 | 2.75E-25 |
| CCBE1 | 702.3333 | 46.64451 | -3.91238 | 1.01E-27 | 2.62E-26 |
| HTR3E | 81.19608 | 5.400309 | -3.9103 | 1.16E-29 | 5.13E-28 |
| PHOX2A | 39.92157 | 2.664606 | -3.90517 | 1.06E-32 | 2.03E-30 |
| PKHD1L1 | 181.3529 | 12.11283 | -3.90419 | 2.11E-31 | 2.06E-29 |
| RP11-396O20.1 | 10.47059 | 0.700155 | -3.90253 | 1.41E-30 | 9.48E-29 |
| RP11-753H16.3 | 27.56863 | 1.843895 | -3.9022 | 1.38E-17 | 7.57E-17 |
| CTNNA3 | 160.1569 | 10.72025 | -3.90108 | 9.12E-29 | 3.08E-27 |
| MT1G | 16632.43 | 1115.832 | -3.89781 | 1.31E-27 | 3.28E-26 |
| CASR | 27.07843 | 1.826893 | -3.88968 | 2.06E-30 | 1.26E-28 |
| HSD17B2 | 7853.333 | 529.9521 | -3.88937 | 5.39E-30 | 2.70E-28 |
| AC103563.5 | 58.35294 | 3.948995 | -3.88525 | 2.24E-31 | 2.13E-29 |
| WISP2 | 606.5882 | 41.07883 | -3.88425 | 4.14E-30 | 2.17E-28 |
| KCNB1 | 300.3725 | 20.3493 | -3.8837 | 2.10E-27 | 4.93E-26 |
| NAP1L2 | 223.4118 | 15.15456 | -3.88188 | 5.18E-32 | 6.93E-30 |
| SYNPO2 | 27521.02 | 1874.836 | -3.8757 | 2.34E-25 | 3.67E-24 |
| TMEM236 | 5272.49 | 359.5348 | -3.87428 | 2.15E-31 | 2.07E-29 |
| NLGN1 | 123.8627 | 8.463679 | -3.87131 | 2.35E-29 | 9.28E-28 |
| SCN2B | 195.8824 | 13.3864 | -3.87115 | 1.70E-29 | 7.11E-28 |
| TAGLN3 | 129.7059 | 9.001546 | -3.84893 | 5.33E-29 | 1.91E-27 |
| UGT1A10 | 2337.843 | 162.2504 | -3.84888 | 5.27E-31 | 4.28E-29 |
| SLC7A14 | 108.2157 | 7.511592 | -3.84865 | 1.59E-31 | 1.67E-29 |
| FOXD3 | 57.05882 | 3.962906 | -3.84782 | 1.29E-30 | 8.90E-29 |
| C7 | 6051.686 | 421.0263 | -3.84536 | 4.60E-29 | 1.69E-27 |
| C16orf89 | 475.7647 | 33.35394 | -3.83432 | 5.14E-31 | 4.19E-29 |
| HSPB8 | 5067.078 | 355.8114 | -3.83197 | 2.53E-24 | 3.30E-23 |
| ADGRB3 | 134.3529 | 9.44204 | -3.83079 | 1.07E-30 | 7.56E-29 |
| IGKV2-30 | 528.4902 | 37.16847 | -3.82973 | 2.28E-28 | 6.87E-27 |
| AC079776.2 | 5.823529 | 0.409583 | -3.82967 | 1.04E-42 | 9.33E-40 |
| DES | 103282.1 | 7308.159 | -3.82094 | 2.39E-23 | 2.65E-22 |
| MRGPRX2 | 4.607843 | 0.326121 | -3.82061 | 1.56E-40 | 1.18E-37 |
| LMO3 | 1579.843 | 111.8995 | -3.81951 | 4.36E-25 | 6.48E-24 |
| SEMA3E | 282.6667 | 20.05719 | -3.81691 | 8.68E-31 | 6.47E-29 |
| LONRF2 | 434.0588 | 30.83308 | -3.81534 | 3.82E-26 | 6.95E-25 |
| SLCO4C1 | 269.2745 | 19.22411 | -3.80809 | 2.12E-28 | 6.44E-27 |
| ANGPTL5 | 19.19608 | 1.372488 | -3.80595 | 8.58E-33 | 1.76E-30 |
| NEFM | 84.17647 | 6.034003 | -3.80223 | 4.35E-29 | 1.60E-27 |
| AP001627.1 | 67.60784 | 4.890263 | -3.78921 | 1.24E-25 | 2.02E-24 |
| ENPP6 | 130.902 | 9.49459 | -3.78524 | 2.64E-32 | 4.21E-30 |
| SYT10 | 29.41176 | 2.137558 | -3.78236 | 5.82E-36 | 2.07E-33 |
| LRRN2 | 1997.157 | 145.1901 | -3.78193 | 2.95E-30 | 1.65E-28 |
| BVES-AS1 | 27.84314 | 2.044822 | -3.76727 | 5.30E-29 | 1.90E-27 |
| CTNND2 | 104.451 | 7.717156 | -3.75861 | 7.79E-32 | 9.34E-30 |
| NPY6R | 356.0196 | 26.38176 | -3.75434 | 2.42E-29 | 9.50E-28 |
| ROPN1 | 20.80392 | 1.545595 | -3.75062 | 1.18E-31 | 1.31E-29 |
| ASTN1 | 83.52941 | 6.210201 | -3.74957 | 1.25E-31 | 1.38E-29 |
| RP11-441F2.5 | 23.17647 | 1.723338 | -3.74938 | 1.38E-24 | 1.89E-23 |
| DNASE1L3 | 1417.51 | 105.4869 | -3.74822 | 1.75E-29 | 7.27E-28 |
| RP11-35P15.1 | 83.29412 | 6.217929 | -3.74371 | 1.22E-30 | 8.49E-29 |
| FRRS1L | 106.2941 | 7.94745 | -3.74143 | 5.79E-29 | 2.06E-27 |
| PDE6A | 544.1569 | 40.93972 | -3.73245 | 7.03E-29 | 2.44E-27 |
| NAALADL1 | 2182.745 | 164.5765 | -3.72931 | 4.08E-30 | 2.15E-28 |
| ELAVL3 | 33.29412 | 2.514683 | -3.72682 | 1.06E-30 | 7.53E-29 |
| PGM5 | 6503.784 | 491.6569 | -3.72556 | 2.28E-27 | 5.32E-26 |
| RP11-532N4.2 | 3.862745 | 0.292117 | -3.72501 | 1.53E-33 | 3.49E-31 |
| RP11-380P13.1 | 17.11765 | 1.295209 | -3.72423 | 2.99E-34 | 7.96E-32 |
| LINC00955 | 55.11765 | 4.179289 | -3.72118 | 3.47E-15 | 1.50E-14 |
| MAB21L1 | 55.62745 | 4.22102 | -3.72013 | 2.16E-31 | 2.07E-29 |
| CHRM2 | 755.9804 | 57.37713 | -3.7198 | 1.16E-22 | 1.16E-21 |
| ARPP21 | 17.80392 | 1.352396 | -3.71861 | 5.70E-34 | 1.41E-31 |
| SFRP5 | 276.902 | 21.08501 | -3.71509 | 1.38E-27 | 3.42E-26 |
| GDPD2 | 1182.471 | 90.23957 | -3.7119 | 1.65E-30 | 1.05E-28 |
| AC116035.1 | 6.392157 | 0.488408 | -3.71014 | 1.65E-43 | 1.54E-40 |
| IGHV3OR16-7 | 15.96078 | 1.222566 | -3.70655 | 7.39E-31 | 5.73E-29 |
| TMEM82 | 860.7059 | 65.93972 | -3.7063 | 5.09E-31 | 4.16E-29 |
| TUBB4A | 204.2549 | 15.7558 | -3.69642 | 1.46E-30 | 9.69E-29 |
| FAM107A | 1413.549 | 109.2056 | -3.6942 | 1.63E-31 | 1.71E-29 |
| POPDC2 | 1204.745 | 93.11128 | -3.69363 | 7.89E-17 | 4.00E-16 |
| TRDV3 | 9.156863 | 0.710974 | -3.68699 | 1.31E-27 | 3.26E-26 |
| DHRS9 | 7622.882 | 592.0618 | -3.68652 | 4.51E-30 | 2.34E-28 |
| KRT27 | 5.862745 | 0.455951 | -3.68463 | 7.18E-37 | 3.00E-34 |
| LMOD1 | 13306.39 | 1035.828 | -3.68326 | 1.37E-26 | 2.70E-25 |
| KIAA2022 | 137.2941 | 10.69861 | -3.68177 | 1.72E-30 | 1.09E-28 |
| RP11-542M13.2 | 93.23529 | 7.278207 | -3.67922 | 2.65E-30 | 1.52E-28 |
| LRRC3B | 12.7451 | 0.995363 | -3.67858 | 1.15E-35 | 3.87E-33 |
| RP13-497K6.1 | 46.11765 | 3.604328 | -3.67752 | 4.34E-31 | 3.65E-29 |
| B3GNT7 | 17795.84 | 1392.923 | -3.67535 | 2.71E-27 | 6.20E-26 |
| SCN9A | 890.7451 | 69.79134 | -3.67389 | 8.48E-32 | 1.00E-29 |
| BRINP3 | 586.8431 | 46.06028 | -3.67138 | 2.26E-30 | 1.35E-28 |
| GNAO1 | 1906.059 | 149.7388 | -3.67007 | 1.77E-28 | 5.51E-27 |
| C20orf166-AS1 | 140.2157 | 11.02628 | -3.66863 | 4.30E-28 | 1.22E-26 |
| TNFRSF13B | 194.0392 | 15.26893 | -3.66768 | 8.59E-30 | 3.95E-28 |
| TNFRSF17 | 633.8431 | 49.93045 | -3.66613 | 1.20E-29 | 5.27E-28 |
| FRMD6-AS2 | 12.15686 | 0.959815 | -3.66287 | 3.94E-37 | 1.80E-34 |
| HSPB6 | 4541.118 | 358.6182 | -3.66253 | 2.62E-26 | 4.92E-25 |
| TMEM151B | 69.80392 | 5.534776 | -3.65671 | 8.97E-31 | 6.64E-29 |
| LRAT | 92.37255 | 7.36476 | -3.64875 | 3.39E-23 | 3.67E-22 |
| PLN | 3676.784 | 294.762 | -3.64082 | 3.26E-23 | 3.54E-22 |
| B3GALT1 | 803.549 | 64.4374 | -3.64042 | 4.88E-30 | 2.50E-28 |
| AC092652.1 | 12.84314 | 1.030912 | -3.639 | 4.19E-46 | 5.96E-43 |
| RP11-232L2.2 | 5.333333 | 0.42813 | -3.63892 | 8.85E-29 | 3.01E-27 |
| MAGEE2 | 11.62745 | 0.933539 | -3.63868 | 1.27E-37 | 6.26E-35 |
| RIMS4 | 131.451 | 10.55951 | -3.63791 | 1.05E-30 | 7.53E-29 |
| RP11-132N15.3 | 5.941176 | 0.477589 | -3.63691 | 7.28E-16 | 3.35E-15 |
| SCN11A | 105.8235 | 8.51932 | -3.63478 | 2.34E-30 | 1.37E-28 |
| SLC17A4 | 2287.804 | 184.3385 | -3.63353 | 6.03E-30 | 2.97E-28 |
| PNCK | 784.4706 | 63.28284 | -3.63183 | 9.74E-16 | 4.44E-15 |
| PLA2G2C | 21.94118 | 1.771252 | -3.6308 | 3.92E-18 | 2.27E-17 |
| PSD | 2636.51 | 213.0124 | -3.62962 | 2.26E-23 | 2.52E-22 |
| CTSG | 409.1373 | 33.06028 | -3.62941 | 1.50E-30 | 9.90E-29 |
| MGAT4C | 38.29412 | 3.100464 | -3.62657 | 9.29E-33 | 1.87E-30 |
| TCEAL5 | 30.88235 | 2.500773 | -3.62634 | 8.02E-35 | 2.40E-32 |
| HPSE2 | 297.6863 | 24.16538 | -3.62278 | 3.66E-32 | 5.40E-30 |
| PADI2 | 26851.63 | 2182.141 | -3.62119 | 1.91E-30 | 1.19E-28 |
| EPHA7 | 1136.706 | 92.70634 | -3.61605 | 1.18E-28 | 3.83E-27 |
| EDN3 | 3231.118 | 263.8578 | -3.6142 | 3.21E-31 | 2.80E-29 |
| C2orf71 | 4.941176 | 0.404946 | -3.60905 | 7.27E-47 | 1.35E-43 |
| ADAMTS9-AS1 | 258.451 | 21.18547 | -3.60874 | 4.18E-23 | 4.46E-22 |
| MCHR2 | 4.176471 | 0.343122 | -3.60549 | 2.85E-39 | 1.92E-36 |
| BTNL8 | 4482.373 | 368.8238 | -3.60326 | 4.62E-29 | 1.69E-27 |
| RBPMS2 | 1203.961 | 99.07728 | -3.60309 | 6.98E-26 | 1.20E-24 |
| MIR143 | 18.29412 | 1.510046 | -3.59872 | 3.97E-30 | 2.10E-28 |
| SLITRK3 | 121.2549 | 10.01855 | -3.5973 | 3.58E-30 | 1.94E-28 |
| ABCC13 | 400.4706 | 33.08964 | -3.59724 | 3.10E-31 | 2.74E-29 |
| RP11-475B2.1 | 7.803922 | 0.646059 | -3.59446 | 1.47E-24 | 1.99E-23 |
| GABRG1 | 5.411765 | 0.448223 | -3.59381 | 4.51E-52 | 3.64E-48 |
| KLHL1 | 4.235294 | 0.35085 | -3.59354 | 2.12E-44 | 2.24E-41 |
| DMRTA1 | 129.7451 | 10.81917 | -3.58402 | 4.66E-31 | 3.87E-29 |
| ADGRG4 | 11.78431 | 0.982998 | -3.58353 | 2.99E-45 | 3.29E-42 |
| AC053503.12 | 77.2549 | 6.445131 | -3.58334 | 1.64E-22 | 1.61E-21 |
| FCRL4 | 56.92157 | 4.771252 | -3.57654 | 1.69E-24 | 2.27E-23 |
| XXbac-BPG13B8.10 | 18.68627 | 1.567233 | -3.57569 | 1.05E-34 | 2.99E-32 |
| KIAA0408 | 18.43137 | 1.547141 | -3.57449 | 7.22E-32 | 8.89E-30 |
| PLD5 | 18.92157 | 1.590417 | -3.57255 | 1.49E-32 | 2.63E-30 |
| AC074363.1 | 19.07843 | 1.604328 | -3.5719 | 8.15E-32 | 9.72E-30 |
| METTL24 | 141.5686 | 11.92581 | -3.56934 | 1.38E-26 | 2.74E-25 |
| HRASLS2 | 554.7451 | 46.76971 | -3.56818 | 4.65E-28 | 1.30E-26 |
| CCL23 | 125.8235 | 10.66615 | -3.56029 | 7.15E-31 | 5.57E-29 |
| SLAMF6P1 | 76.86275 | 6.516229 | -3.56018 | 1.30E-30 | 8.96E-29 |
| FMN2 | 287.8627 | 24.46213 | -3.55676 | 1.19E-26 | 2.40E-25 |
| UGT2B29P | 6.529412 | 0.554869 | -3.55673 | 6.08E-28 | 1.66E-26 |
| AC002398.12 | 14.01961 | 1.191654 | -3.55641 | 1.17E-22 | 1.16E-21 |
| MAB21L2 | 4090.765 | 347.796 | -3.55606 | 6.85E-23 | 7.08E-22 |
| RALYL | 14.66667 | 1.247295 | -3.55567 | 4.81E-35 | 1.51E-32 |
| TCL1A | 367.9608 | 31.44359 | -3.54871 | 1.10E-17 | 6.11E-17 |
| IGLJCOR18 | 3.666667 | 0.313756 | -3.54676 | 7.69E-24 | 9.27E-23 |
| FHL1 | 12000.04 | 1028.182 | -3.54487 | 2.09E-29 | 8.46E-28 |
| SI | 6690.02 | 573.2241 | -3.54484 | 4.37E-26 | 7.83E-25 |
| PPP1R1A | 412.7255 | 35.77434 | -3.52819 | 1.52E-22 | 1.50E-21 |
| PDK4 | 8450.02 | 733.4668 | -3.52615 | 2.89E-31 | 2.60E-29 |
| NRSN1 | 32.80392 | 2.848532 | -3.52558 | 5.75E-30 | 2.85E-28 |
| P2RY4 | 55.66667 | 4.843895 | -3.52257 | 2.34E-25 | 3.67E-24 |
| RPL7P3 | 3.745098 | 0.326121 | -3.52153 | 2.17E-22 | 2.09E-21 |
| ST8SIA3 | 34.07843 | 2.969088 | -3.52077 | 9.88E-33 | 1.94E-30 |
| TM6SF2 | 380.451 | 33.15765 | -3.5203 | 4.15E-25 | 6.20E-24 |
| AGTR1 | 351.2745 | 30.63524 | -3.51933 | 2.19E-25 | 3.45E-24 |
| CTD-2339F6.1 | 5 | 0.437403 | -3.51489 | 4.16E-37 | 1.86E-34 |
| CDKN2B | 6880.824 | 602.5394 | -3.51345 | 8.05E-31 | 6.05E-29 |
| VIP | 2556.529 | 224.2087 | -3.51127 | 5.29E-30 | 2.65E-28 |
| IGKV3D-7 | 36.64706 | 3.219474 | -3.5088 | 4.54E-28 | 1.28E-26 |
| UGT2A3 | 5824.098 | 511.8702 | -3.50818 | 4.94E-30 | 2.52E-28 |
| FLNC | 21549.73 | 1902.04 | -3.50205 | 4.58E-19 | 2.94E-18 |
| FGFBP2 | 76.78431 | 6.783617 | -3.50069 | 3.06E-30 | 1.71E-28 |
| ABCB5 | 60.37255 | 5.344668 | -3.49772 | 1.93E-27 | 4.60E-26 |
| CLCNKB | 41.56863 | 3.684699 | -3.49588 | 2.97E-22 | 2.81E-21 |
| RP11-307N16.7 | 14.60784 | 1.295209 | -3.49549 | 1.80E-21 | 1.53E-20 |
| LVRN | 81.21569 | 7.205564 | -3.49458 | 1.17E-28 | 3.79E-27 |
| ACTG1P22 | 35.68627 | 3.173107 | -3.4914 | 7.30E-28 | 1.97E-26 |
| RP11-384P7.7 | 65.05882 | 5.785162 | -3.49132 | 1.44E-27 | 3.55E-26 |
| FABP2 | 3213.608 | 287.0046 | -3.48505 | 1.44E-29 | 6.18E-28 |
| ELAVL4 | 155.3922 | 13.89645 | -3.48313 | 1.53E-30 | 1.00E-28 |
| MYLK | 47372.27 | 4246.471 | -3.47971 | 1.68E-26 | 3.26E-25 |
| WI2-2373I1.2 | 4.117647 | 0.369397 | -3.47858 | 3.31E-34 | 8.62E-32 |
| RP11-481J13.1 | 12.07843 | 1.085008 | -3.47666 | 1.94E-19 | 1.29E-18 |
| ZMAT4 | 22.19608 | 1.995363 | -3.47558 | 2.42E-29 | 9.50E-28 |
| AP000472.2 | 31.7451 | 2.85626 | -3.47433 | 9.92E-34 | 2.35E-31 |
| RP11-307B6.3 | 15.41176 | 1.387944 | -3.47301 | 2.76E-23 | 3.04E-22 |
| LIFR | 1432.471 | 129.408 | -3.46851 | 3.18E-31 | 2.79E-29 |
| ADTRP | 5709.333 | 516.0185 | -3.46783 | 1.52E-29 | 6.44E-28 |
| RP11-357D18.1 | 5 | 0.452859 | -3.46479 | 5.03E-47 | 1.11E-43 |
| CPEB1 | 158.4902 | 14.38176 | -3.46208 | 8.69E-30 | 3.99E-28 |
| AC004947.2 | 29.72549 | 2.703246 | -3.45894 | 1.86E-31 | 1.88E-29 |
| AICDA | 40.47059 | 3.684699 | -3.45726 | 2.72E-21 | 2.27E-20 |
| CLDN23 | 6290.706 | 574.8578 | -3.45194 | 7.75E-31 | 5.88E-29 |
| IGSF11 | 63.19608 | 5.775889 | -3.45172 | 6.05E-29 | 2.14E-27 |
| RP11-6O2.3 | 184.9608 | 16.90726 | -3.4515 | 1.31E-23 | 1.50E-22 |
| GPT | 4022.235 | 367.7002 | -3.4514 | 1.58E-30 | 1.03E-28 |
| AC092667.2 | 5.215686 | 0.477589 | -3.44902 | 2.74E-35 | 8.74E-33 |
| RNF150 | 1406.824 | 128.83 | -3.4489 | 1.16E-24 | 1.60E-23 |
| MMRN1 | 1409.392 | 129.102 | -3.44849 | 5.88E-30 | 2.90E-28 |
| IGHV3OR16-6 | 57.90196 | 5.315301 | -3.44539 | 7.89E-27 | 1.65E-25 |
| MYL9 | 77808.63 | 7179.652 | -3.43794 | 6.20E-21 | 4.94E-20 |
| IGKV6D-21 | 318.8627 | 29.42504 | -3.43782 | 1.06E-23 | 1.24E-22 |
| OLFM3 | 43.45098 | 4.017002 | -3.4352 | 7.21E-32 | 8.89E-30 |
| AKR1B10 | 6617.549 | 611.9815 | -3.43474 | 3.36E-30 | 1.85E-28 |
| KRT9 | 20.13725 | 1.868624 | -3.42982 | 3.59E-30 | 1.94E-28 |
| MASP1 | 1680.353 | 156.0835 | -3.42837 | 6.71E-23 | 6.96E-22 |
| CA3-AS1 | 111.4118 | 10.35858 | -3.427 | 2.31E-30 | 1.36E-28 |
| MT1F | 3452.039 | 321.0618 | -3.42653 | 5.53E-27 | 1.19E-25 |
| HPGDS | 295.0392 | 27.45595 | -3.42572 | 7.43E-32 | 9.05E-30 |
| KCNMB1 | 2541.902 | 237.5054 | -3.41988 | 2.03E-24 | 2.69E-23 |
| RP11-164H13.1 | 18.5098 | 1.732612 | -3.41727 | 2.77E-19 | 1.81E-18 |
| PDZD4 | 521.2745 | 48.87326 | -3.41493 | 6.75E-30 | 3.23E-28 |
| RNU6-140P | 19.72549 | 1.851623 | -3.4132 | 8.66E-32 | 1.01E-29 |
| IGKV1-12 | 287.3529 | 26.97372 | -3.4132 | 8.55E-26 | 1.44E-24 |
| TRIM40 | 760.451 | 71.45595 | -3.41173 | 1.38E-26 | 2.73E-25 |
| MIR1-1HG | 4.686275 | 0.440495 | -3.41125 | 2.73E-25 | 4.22E-24 |
| MYOM1 | 917.8824 | 86.2983 | -3.41091 | 1.11E-28 | 3.64E-27 |
| RASGEF1C | 49.52941 | 4.656878 | -3.41085 | 2.24E-29 | 8.94E-28 |
| SNAP91 | 78.45098 | 7.378671 | -3.41036 | 2.45E-29 | 9.60E-28 |
| RP11-654D12.2 | 7.705882 | 0.724884 | -3.41014 | 6.75E-31 | 5.34E-29 |
| FUT9 | 19.23529 | 1.811437 | -3.40855 | 1.65E-30 | 1.05E-28 |
| KIAA1644 | 959.2157 | 90.64451 | -3.40356 | 5.22E-25 | 7.65E-24 |
| TPH1 | 612.6863 | 57.966 | -3.40187 | 6.62E-30 | 3.19E-28 |
| LINC00891 | 12.92157 | 1.224111 | -3.39997 | 6.01E-32 | 7.95E-30 |
| FAM129A | 6214.275 | 588.7944 | -3.39975 | 1.24E-27 | 3.12E-26 |
| NRG2 | 68.56863 | 6.500773 | -3.39887 | 1.25E-29 | 5.47E-28 |
| SEPP1 | 31658.9 | 3005.663 | -3.39686 | 3.45E-30 | 1.88E-28 |
| ZBTB16 | 316.1373 | 30.02009 | -3.39655 | 2.07E-26 | 3.94E-25 |
| PLAC8 | 16300.25 | 1548.723 | -3.39574 | 5.55E-30 | 2.77E-28 |
| RP11-308N19.1 | 8.764706 | 0.833076 | -3.39519 | 1.79E-27 | 4.30E-26 |
| G6PC | 205.5686 | 19.54714 | -3.39459 | 4.53E-19 | 2.91E-18 |
| RP11-420K14.1 | 3.509804 | 0.333849 | -3.39412 | 1.98E-26 | 3.79E-25 |
| ABI3BP | 2757.941 | 263.6955 | -3.38665 | 2.28E-30 | 1.35E-28 |
| TMEM253 | 460.2745 | 44.07419 | -3.38449 | 1.62E-30 | 1.04E-28 |
| SPINK2 | 83.54902 | 8.009274 | -3.38288 | 3.89E-30 | 2.07E-28 |
| LDHD | 3927.353 | 376.8964 | -3.38132 | 3.28E-31 | 2.85E-29 |
| NOS1 | 284.7059 | 27.3323 | -3.38079 | 3.00E-19 | 1.96E-18 |
| PCOLCE2 | 591.9412 | 56.83617 | -3.38057 | 7.78E-29 | 2.67E-27 |
| RP11-170L3.7 | 74.2549 | 7.142195 | -3.37805 | 3.06E-27 | 6.93E-26 |
| UGT2B15 | 1757.745 | 169.0711 | -3.37802 | 8.80E-20 | 6.11E-19 |
| GPR12 | 9.333333 | 0.902628 | -3.37019 | 1.16E-32 | 2.19E-30 |
| ANO5 | 651.8824 | 63.10355 | -3.36882 | 7.96E-31 | 6.01E-29 |
| ATCAY | 70.21569 | 6.802164 | -3.36773 | 4.63E-30 | 2.38E-28 |
| FXYD1 | 129.3137 | 12.53941 | -3.36633 | 5.59E-31 | 4.51E-29 |
| CPB1 | 104.7451 | 10.16383 | -3.36537 | 7.78E-23 | 7.95E-22 |
| UNC5D | 52.66667 | 5.111283 | -3.36513 | 1.00E-29 | 4.51E-28 |
| FLJ41941 | 9.745098 | 0.948995 | -3.3602 | 5.91E-27 | 1.26E-25 |
| IGLV10-54 | 1706.922 | 166.272 | -3.35978 | 3.32E-23 | 3.60E-22 |
| AC007556.3 | 6.803922 | 0.66306 | -3.35915 | 1.68E-34 | 4.63E-32 |
| WSCD1 | 2427.941 | 236.6244 | -3.35906 | 1.86E-30 | 1.16E-28 |
| CHODL | 176.7451 | 17.23184 | -3.35852 | 1.11E-29 | 4.93E-28 |
| GSG1L | 20.5098 | 2.001546 | -3.35713 | 5.15E-32 | 6.93E-30 |
| ANK2 | 1821.882 | 177.8501 | -3.3567 | 5.05E-29 | 1.83E-27 |
| UG0898H09 | 74.2549 | 7.276662 | -3.35114 | 1.57E-27 | 3.84E-26 |
| TMEM37 | 5811.686 | 569.6893 | -3.35071 | 5.13E-30 | 2.60E-28 |
| SECTM1 | 8873.765 | 872.0232 | -3.34711 | 1.46E-29 | 6.23E-28 |
| CHL1-AS2 | 11.58824 | 1.139104 | -3.34669 | 3.14E-31 | 2.77E-29 |
| PP7080 | 12804.55 | 1259.77 | -3.34542 | 5.34E-16 | 2.50E-15 |
| MOBP | 39.98039 | 3.935085 | -3.34483 | 1.65E-29 | 6.93E-28 |
| AFF3 | 508.7647 | 50.12828 | -3.3433 | 3.73E-30 | 2.00E-28 |
| SLC23A1 | 813.4314 | 80.35394 | -3.33958 | 1.47E-24 | 1.99E-23 |
| LINC01014 | 3.54902 | 0.35085 | -3.33849 | 2.31E-42 | 2.00E-39 |
| TMEM220 | 1053.02 | 104.1932 | -3.3372 | 8.68E-32 | 1.01E-29 |
| CBLN2 | 118.4902 | 11.72488 | -3.33712 | 6.69E-32 | 8.53E-30 |
| IGKV3OR2-268 | 146.9216 | 14.54405 | -3.33655 | 2.15E-27 | 5.04E-26 |
| C8orf88 | 160.9608 | 15.9459 | -3.33545 | 6.20E-25 | 8.97E-24 |
| IGKV1OR9-2 | 12.76471 | 1.265842 | -3.33399 | 1.80E-30 | 1.13E-28 |
| ADAMTSL3 | 1023.647 | 101.6739 | -3.3317 | 8.80E-27 | 1.82E-25 |
| RP11-646E18.2 | 5.176471 | 0.514683 | -3.33021 | 7.23E-32 | 8.89E-30 |
| VSIG2 | 9143.529 | 910.864 | -3.32744 | 5.49E-29 | 1.96E-27 |
| KCNMA1 | 5479.216 | 545.8501 | -3.32739 | 1.03E-27 | 2.67E-26 |
| IGKV2D-30 | 69 | 6.876352 | -3.32688 | 9.99E-29 | 3.33E-27 |
| IGLV7-43 | 1567.078 | 156.1901 | -3.3267 | 1.26E-27 | 3.15E-26 |
| RP11-245G13.2 | 28.43137 | 2.839258 | -3.3239 | 3.48E-30 | 1.89E-28 |
| GABRG2 | 37 | 3.7017 | -3.32127 | 8.40E-34 | 2.03E-31 |
| LINC01351 | 21.03922 | 2.106646 | -3.32006 | 1.48E-38 | 8.91E-36 |
| PENK | 203.4706 | 20.37713 | -3.3198 | 7.01E-16 | 3.24E-15 |
| RP11-131H24.4 | 24.62745 | 2.46677 | -3.31957 | 2.54E-31 | 2.34E-29 |
| IGHV3-72 | 2158.039 | 216.221 | -3.31914 | 5.10E-27 | 1.11E-25 |
| UBXN10-AS1 | 124.3333 | 12.49304 | -3.31502 | 8.12E-19 | 5.10E-18 |
| RP11-285G1.9 | 14.66667 | 1.477589 | -3.31122 | 9.66E-31 | 7.07E-29 |
| CTC-297N7.9 | 47.86275 | 4.826893 | -3.30974 | 1.64E-31 | 1.71E-29 |
| ZNF536 | 149.098 | 15.04173 | -3.30922 | 2.55E-31 | 2.34E-29 |
| FAM163B | 86.03922 | 8.693972 | -3.30691 | 1.62E-23 | 1.83E-22 |
| DAND5 | 52.88235 | 5.352396 | -3.30453 | 3.02E-15 | 1.31E-14 |
| IGKV1D-39 | 124.4902 | 12.6476 | -3.2991 | 3.05E-25 | 4.68E-24 |
| RPL12P14 | 147.0784 | 14.96291 | -3.29712 | 5.23E-30 | 2.63E-28 |
| CD163L1 | 2126.235 | 216.5549 | -3.2955 | 4.57E-29 | 1.68E-27 |
| ASPG | 626.8431 | 63.8609 | -3.2951 | 5.89E-24 | 7.25E-23 |
| SEMA6D | 2714.02 | 276.6801 | -3.29414 | 1.98E-31 | 1.98E-29 |
| IGKV1OR9-1 | 3.196078 | 0.326121 | -3.29283 | 4.91E-37 | 2.16E-34 |
| IGKV1OR2-108 | 471.8235 | 48.17774 | -3.29181 | 5.97E-25 | 8.67E-24 |
| IGKV1D-33 | 40 | 4.085008 | -3.29159 | 2.27E-24 | 2.97E-23 |
| ANKS1B | 95.01961 | 9.707883 | -3.291 | 5.52E-28 | 1.52E-26 |
| IGLV3-17 | 7.784314 | 0.795981 | -3.28976 | 1.91E-26 | 3.67E-25 |
| TRPV3 | 502.5882 | 51.43895 | -3.28844 | 2.09E-25 | 3.31E-24 |
| GAP43 | 297.7451 | 30.57187 | -3.2838 | 1.20E-28 | 3.89E-27 |
| GFRA1 | 1208.02 | 124.1963 | -3.28195 | 5.16E-29 | 1.87E-27 |
| C1orf95 | 969.549 | 99.80371 | -3.28015 | 1.07E-25 | 1.76E-24 |
| ASB2 | 1508.451 | 155.4003 | -3.27901 | 3.71E-29 | 1.40E-27 |
| RP11-467J12.4 | 31.13725 | 3.211747 | -3.27721 | 6.68E-27 | 1.42E-25 |
| CTD-2270F17.1 | 5.901961 | 0.608964 | -3.27676 | 2.73E-23 | 3.00E-22 |
| RP11-1151B14.4 | 5.352941 | 0.554869 | -3.27011 | 4.37E-33 | 9.20E-31 |
| SLC17A7 | 108.1961 | 11.2272 | -3.26858 | 1.46E-31 | 1.56E-29 |
| HMGCLL1 | 39.92157 | 4.156105 | -3.26386 | 4.66E-32 | 6.38E-30 |
| RP11-367F23.1 | 11.41176 | 1.190108 | -3.26136 | 2.66E-10 | 7.30E-10 |
| NEGR1 | 1487.569 | 155.575 | -3.25727 | 6.54E-30 | 3.17E-28 |
| CTA-150C2.13 | 19.05882 | 1.995363 | -3.25574 | 1.87E-28 | 5.78E-27 |
| NOVA1 | 306.3725 | 32.11901 | -3.25379 | 1.65E-29 | 6.93E-28 |
| ZDHHC22 | 23.35294 | 2.452859 | -3.25107 | 1.55E-30 | 1.01E-28 |
| SDPR | 2767.627 | 290.7527 | -3.25079 | 1.47E-28 | 4.67E-27 |
| OSTN-AS1 | 11.17647 | 1.174652 | -3.25016 | 1.77E-25 | 2.83E-24 |
| FAM46B | 515.0392 | 54.50541 | -3.24021 | 4.39E-20 | 3.16E-19 |
| PTCHD1 | 275.8431 | 29.23648 | -3.23801 | 1.31E-25 | 2.14E-24 |
| GSTM5 | 412.3725 | 43.75734 | -3.23635 | 2.66E-31 | 2.42E-29 |
| JPH2 | 1980.451 | 210.1777 | -3.23615 | 9.14E-23 | 9.23E-22 |
| LCN6 | 27.70588 | 2.941267 | -3.23568 | 4.26E-29 | 1.58E-27 |
| RP11-2I17.4 | 22.41176 | 2.386399 | -3.23135 | 5.46E-27 | 1.18E-25 |
| AMER2 | 9.921569 | 1.057187 | -3.23034 | 2.06E-24 | 2.72E-23 |
| RP11-867G23.10 | 82.41176 | 8.783617 | -3.22996 | 8.20E-24 | 9.83E-23 |
| NCR2 | 8.117647 | 0.867079 | -3.22683 | 7.67E-24 | 9.25E-23 |
| MPZ | 411.1373 | 43.92117 | -3.22663 | 3.97E-30 | 2.10E-28 |
| RP11-348F1.2 | 3.960784 | 0.423493 | -3.22538 | 1.69E-38 | 9.51E-36 |
| CHRDL1 | 3333.196 | 356.745 | -3.22394 | 7.70E-28 | 2.06E-26 |
| NXPE4 | 9851.647 | 1058.988 | -3.21768 | 2.87E-28 | 8.48E-27 |
| EDN2 | 339.3333 | 36.48223 | -3.21744 | 2.66E-19 | 1.74E-18 |
| FCER2 | 224.6863 | 24.16847 | -3.21671 | 7.62E-27 | 1.60E-25 |
| RP11-738O11.13 | 8.333333 | 0.896445 | -3.21661 | 1.67E-46 | 2.88E-43 |
| LGALS2 | 2361.216 | 254.3138 | -3.21485 | 3.95E-26 | 7.16E-25 |
| MYT1L | 18.76471 | 2.02473 | -3.21222 | 5.41E-31 | 4.38E-29 |
| PCAT18 | 22.41176 | 2.421947 | -3.21002 | 3.91E-33 | 8.30E-31 |
| UGT1A1 | 184.451 | 20.04482 | -3.20194 | 2.22E-24 | 2.92E-23 |
| CTD-2135D7.5 | 12.35294 | 1.347759 | -3.19622 | 1.05E-32 | 2.03E-30 |
| RP11-321G12.1 | 315.1961 | 34.40958 | -3.19537 | 4.24E-28 | 1.20E-26 |
| RNF152 | 2283.314 | 249.3694 | -3.19477 | 5.17E-30 | 2.61E-28 |
| C14orf180 | 35.07843 | 3.833076 | -3.19401 | 5.39E-35 | 1.67E-32 |
| TINCR | 370.6078 | 40.51777 | -3.19327 | 6.65E-30 | 3.19E-28 |
| RP11-384F7.2 | 5.72549 | 0.625966 | -3.19324 | 1.22E-51 | 7.36E-48 |
| PTENP1-AS | 4.490196 | 0.491499 | -3.19152 | 2.19E-29 | 8.78E-28 |
| GFRA3 | 297.549 | 32.5796 | -3.19109 | 6.10E-30 | 2.98E-28 |
| ENAM | 66.52941 | 7.285935 | -3.19081 | 3.27E-29 | 1.24E-27 |
| CCDC69 | 6413.49 | 703.7311 | -3.18801 | 1.10E-29 | 4.91E-28 |
| MEP1AP4 | 50.41176 | 5.536321 | -3.18676 | 1.15E-19 | 7.83E-19 |
| BEX1 | 106.1961 | 11.67233 | -3.18557 | 9.56E-30 | 4.33E-28 |
| MOGAT2 | 6524.314 | 717.2071 | -3.18536 | 2.91E-30 | 1.64E-28 |
| RP11-1036E20.9 | 13.21569 | 1.454405 | -3.18375 | 3.96E-26 | 7.17E-25 |
| FCRLA | 318.3725 | 35.04791 | -3.18332 | 1.05E-25 | 1.75E-24 |
| HPGD | 10496.86 | 1155.654 | -3.18318 | 4.37E-31 | 3.66E-29 |
| IGHV3OR16-9 | 83.7451 | 9.225657 | -3.18228 | 3.50E-27 | 7.88E-26 |
| IGKV2OR2-1 | 19.80392 | 2.18238 | -3.18181 | 5.64E-27 | 1.21E-25 |
| RP11-445F6.2 | 6 | 0.661515 | -3.18112 | 6.54E-29 | 2.29E-27 |
| SLC27A6 | 50.35294 | 5.553323 | -3.18065 | 7.47E-31 | 5.76E-29 |
| MMP28 | 2654.961 | 292.8794 | -3.18031 | 7.78E-31 | 5.88E-29 |
| PTPRZ1 | 196.5098 | 21.78362 | -3.17329 | 1.32E-27 | 3.28E-26 |
| IGKV1OR-3 | 7.333333 | 0.812983 | -3.17317 | 1.71E-31 | 1.77E-29 |
| CLEC9A | 57.88235 | 6.417311 | -3.17308 | 2.28E-30 | 1.35E-28 |
| ASB11 | 7.098039 | 0.789799 | -3.16786 | 1.39E-24 | 1.89E-23 |
| IGKV1OR2-11 | 22.47059 | 2.500773 | -3.16759 | 1.51E-26 | 2.95E-25 |
| SCRG1 | 182.1961 | 20.28284 | -3.16716 | 3.06E-20 | 2.25E-19 |
| IGHV2-5 | 714.3333 | 79.52859 | -3.16705 | 1.31E-24 | 1.80E-23 |
| CLVS2 | 40.64706 | 4.530139 | -3.16552 | 9.84E-29 | 3.29E-27 |
| NPY | 52.70588 | 5.874807 | -3.16535 | 2.87E-29 | 1.10E-27 |
| TMEM196 | 6.058824 | 0.675425 | -3.16517 | 2.02E-39 | 1.40E-36 |
| PTGS1 | 5637.961 | 629.915 | -3.16194 | 3.17E-29 | 1.21E-27 |
| SCGB2A1 | 199.1176 | 22.26121 | -3.16102 | 2.09E-27 | 4.92E-26 |
| JAZF1-AS1 | 12.13725 | 1.358578 | -3.15927 | 1.35E-23 | 1.55E-22 |
| IGKV1D-37 | 4.235294 | 0.474498 | -3.15799 | 3.18E-22 | 3.01E-21 |
| RP11-501J20.5 | 4.45098 | 0.499227 | -3.15635 | 1.65E-35 | 5.34E-33 |
| AC007403.2 | 3.333333 | 0.374034 | -3.15572 | 1.25E-23 | 1.45E-22 |
| IGHV6-1 | 322.7647 | 36.23338 | -3.15509 | 7.71E-27 | 1.62E-25 |
| NCAM1 | 1457.608 | 163.8284 | -3.15334 | 1.13E-28 | 3.70E-27 |
| GLDN | 662.7059 | 74.54096 | -3.15226 | 3.37E-17 | 1.77E-16 |
| SPATA31E1 | 3.901961 | 0.438949 | -3.15207 | 1.26E-30 | 8.73E-29 |
| MYOCD | 1671.275 | 188.0711 | -3.1516 | 6.33E-26 | 1.10E-24 |
| NKX2-3 | 1123.725 | 126.6213 | -3.1497 | 1.41E-31 | 1.52E-29 |
| IGKV6-21 | 484.0196 | 54.55178 | -3.14937 | 4.50E-26 | 8.04E-25 |
| PCDH10 | 61.52941 | 6.945904 | -3.14704 | 8.84E-28 | 2.34E-26 |
| NTNG1 | 91.88235 | 10.3864 | -3.14509 | 6.47E-24 | 7.92E-23 |
| SPEG | 2203.02 | 249.323 | -3.14339 | 1.19E-21 | 1.03E-20 |
| RP11-475A13.1 | 4.392157 | 0.497682 | -3.14163 | 2.32E-22 | 2.23E-21 |
| RP11-266I3.9 | 5.333333 | 0.604328 | -3.14163 | 5.57E-34 | 1.39E-31 |
| MT1P3 | 12.58824 | 1.426584 | -3.14144 | 1.88E-18 | 1.13E-17 |
| IGKV1-37 | 4.960784 | 0.562597 | -3.1404 | 4.93E-28 | 1.37E-26 |
| CLEC10A | 883.0784 | 100.1731 | -3.14005 | 2.84E-30 | 1.61E-28 |
| CTD-2207O23.3 | 10.17647 | 1.160742 | -3.13212 | 6.08E-30 | 2.98E-28 |
| CHAD | 399.0392 | 45.53014 | -3.13164 | 1.00E-27 | 2.61E-26 |
| STMN2 | 1255.961 | 143.4389 | -3.13028 | 4.38E-31 | 3.66E-29 |
| IGLV5-48 | 53.33333 | 6.095827 | -3.12914 | 6.66E-25 | 9.56E-24 |
| IGHA1 | 318418.8 | 36433.49 | -3.12759 | 9.58E-28 | 2.52E-26 |
| ABCA9 | 338.1373 | 38.69397 | -3.12743 | 4.35E-28 | 1.23E-26 |
| GRIK5 | 357.7647 | 40.96754 | -3.12646 | 1.30E-26 | 2.59E-25 |
| LINC01158 | 4.960784 | 0.568779 | -3.12463 | 2.91E-40 | 2.13E-37 |
| KCNK3 | 281.2745 | 32.29985 | -3.12238 | 3.76E-28 | 1.08E-26 |
| LINC01625 | 3.588235 | 0.412674 | -3.1202 | 2.15E-21 | 1.81E-20 |
| HHLA2 | 10567.47 | 1215.587 | -3.1199 | 2.59E-30 | 1.50E-28 |
| TMEM59L | 161.2745 | 18.55641 | -3.11953 | 1.77E-29 | 7.35E-28 |
| MEF2C-AS1 | 45.35294 | 5.22102 | -3.11879 | 2.19E-29 | 8.79E-28 |
| CTD-3179P9.1 | 5.098039 | 0.587326 | -3.11771 | 6.35E-38 | 3.34E-35 |
| HCG23 | 10.47059 | 1.20711 | -3.11671 | 1.50E-29 | 6.39E-28 |
| LRRC19 | 6953.196 | 802.8995 | -3.11438 | 1.01E-30 | 7.32E-29 |
| IGKV2OR2-7D | 2.901961 | 0.335394 | -3.1131 | 9.90E-28 | 2.58E-26 |
| EPHA5 | 26.07843 | 3.017002 | -3.11167 | 5.66E-26 | 9.97E-25 |
| KCNA4 | 3.058824 | 0.353941 | -3.11139 | 9.76E-36 | 3.38E-33 |
| NECAB1 | 257.2745 | 29.80526 | -3.10967 | 2.63E-24 | 3.41E-23 |
| IGLJ1 | 13.96078 | 1.619784 | -3.10751 | 3.51E-28 | 1.02E-26 |
| IGHV3-76 | 34.13725 | 3.964451 | -3.10615 | 1.47E-25 | 2.38E-24 |
| RP11-305L7.6 | 22.15686 | 2.573416 | -3.106 | 4.36E-30 | 2.27E-28 |
| P2RY12 | 89.90196 | 10.44668 | -3.10531 | 9.96E-30 | 4.49E-28 |
| HTR4 | 252.3333 | 29.32148 | -3.1053 | 2.37E-29 | 9.35E-28 |
| ADCY5 | 1675.647 | 194.728 | -3.10519 | 2.18E-29 | 8.77E-28 |
| RP11-733O18.1 | 28.54902 | 3.318393 | -3.10488 | 1.78E-29 | 7.38E-28 |
| CLEC4M | 12.07843 | 1.404946 | -3.10385 | 2.00E-33 | 4.45E-31 |
| SLC52A1 | 323.7451 | 37.68624 | -3.10275 | 1.52E-19 | 1.02E-18 |
| LINC01080 | 11.88235 | 1.383308 | -3.10263 | 1.24E-30 | 8.66E-29 |
| TMOD1 | 534.6667 | 62.56723 | -3.09516 | 8.18E-27 | 1.71E-25 |
| AC104024.1 | 14.66667 | 1.717156 | -3.09445 | 2.93E-28 | 8.63E-27 |
| IGLV2-8 | 3627.529 | 425.2674 | -3.09255 | 3.19E-27 | 7.22E-26 |
| RIC3 | 116.7843 | 13.69397 | -3.09223 | 3.12E-27 | 7.08E-26 |
| CYP4B1 | 109.1569 | 12.80526 | -3.09159 | 2.24E-24 | 2.95E-23 |
| AGTR2 | 5 | 0.587326 | -3.08969 | 4.31E-27 | 9.52E-26 |
| NPAS4 | 27.37255 | 3.217929 | -3.08853 | 1.41E-20 | 1.08E-19 |
| RP3-467K16.2 | 3.470588 | 0.408037 | -3.08841 | 4.81E-13 | 1.70E-12 |
| GLRA4 | 101.451 | 11.9289 | -3.08825 | 1.14E-28 | 3.72E-27 |
| CXCR5 | 40.09804 | 4.718702 | -3.08707 | 2.34E-23 | 2.59E-22 |
| PCDH11X | 7.098039 | 0.837713 | -3.08289 | 3.94E-32 | 5.66E-30 |
| IGHV1OR15-1 | 4.372549 | 0.516229 | -3.08239 | 1.07E-25 | 1.76E-24 |
| CFD | 6046.824 | 714.6383 | -3.08089 | 3.17E-30 | 1.76E-28 |
| CLEC4G | 80.62745 | 9.531685 | -3.08047 | 5.88E-27 | 1.26E-25 |
| HHATL-AS1 | 3.098039 | 0.366306 | -3.08023 | 3.11E-31 | 2.74E-29 |
| AC245028.1 | 8.490196 | 1.004637 | -3.07912 | 1.83E-26 | 3.52E-25 |
| CTD-2001J20.1 | 11.15686 | 1.321484 | -3.0777 | 1.26E-32 | 2.35E-30 |
| SLC4A10 | 256.4902 | 30.39258 | -3.07711 | 6.53E-30 | 3.17E-28 |
| SLC8A2 | 263.0588 | 31.24111 | -3.07387 | 1.06E-13 | 3.98E-13 |
| BTNL3 | 3983.431 | 474.7558 | -3.06875 | 1.04E-28 | 3.46E-27 |
| CTC-296K1.4 | 36.07843 | 4.302937 | -3.06774 | 9.74E-28 | 2.55E-26 |
| COL19A1 | 33.43137 | 3.989181 | -3.06704 | 2.21E-29 | 8.83E-28 |
| RP11-96D1.6 | 83.45098 | 9.958269 | -3.06696 | 2.01E-29 | 8.20E-28 |
| PROKR1 | 14.45098 | 1.724884 | -3.0666 | 8.87E-29 | 3.01E-27 |
| KCNE2 | 32.11765 | 3.836167 | -3.06563 | 1.11E-29 | 4.93E-28 |
| C1QTNF9 | 34.52941 | 4.134467 | -3.06205 | 1.05E-29 | 4.67E-28 |
| PCK1 | 17423.88 | 2087.198 | -3.06143 | 5.83E-26 | 1.02E-24 |
| CASC18 | 25.64706 | 3.072643 | -3.06124 | 2.19E-22 | 2.11E-21 |
| ADHFE1 | 178.5294 | 21.38949 | -3.06119 | 3.08E-31 | 2.74E-29 |
| SLC16A12 | 33.21569 | 3.981453 | -3.0605 | 1.90E-29 | 7.80E-28 |
| IGKV1OR2-3 | 26.7451 | 3.210201 | -3.05854 | 5.77E-25 | 8.40E-24 |
| KRT222 | 18.94118 | 2.276662 | -3.05653 | 2.44E-31 | 2.28E-29 |
| IGHVIII-67-2 | 5.862745 | 0.704791 | -3.05631 | 1.32E-26 | 2.62E-25 |
| AC093627.8 | 28.90196 | 3.474498 | -3.05629 | 5.76E-22 | 5.24E-21 |
| SRPX | 1528.49 | 183.9057 | -3.05507 | 1.82E-29 | 7.54E-28 |
| NR3C2 | 6500.431 | 782.6121 | -3.05417 | 2.36E-31 | 2.22E-29 |
| IL6R | 3944.137 | 475.3153 | -3.05275 | 4.16E-32 | 5.85E-30 |
| CR2 | 1478.941 | 178.3802 | -3.05154 | 1.84E-21 | 1.56E-20 |
| LINC00461 | 19.62745 | 2.367852 | -3.05122 | 6.48E-25 | 9.34E-24 |
| AHCYL2 | 26000.75 | 3137.026 | -3.05108 | 1.07E-30 | 7.59E-29 |
| KRT20 | 88323.43 | 10665.63 | -3.04983 | 1.59E-30 | 1.03E-28 |
| SGK1 | 12605.98 | 1523.564 | -3.04859 | 1.72E-24 | 2.30E-23 |
| CYP2B7P | 219.5294 | 26.53787 | -3.04829 | 6.42E-23 | 6.67E-22 |
| SLC18A3 | 25.43137 | 3.075734 | -3.04761 | 4.62E-28 | 1.30E-26 |
| TSPAN7 | 4767.314 | 576.9459 | -3.04667 | 1.16E-31 | 1.29E-29 |
| RP1-117B12.4 | 27.37255 | 3.318393 | -3.04417 | 1.14E-28 | 3.72E-27 |
| HBB | 4675.51 | 568.1515 | -3.04078 | 1.24E-26 | 2.48E-25 |
| IGKV1-39 | 174.9804 | 21.26584 | -3.04058 | 1.09E-25 | 1.80E-24 |
| RP1-148H17.1 | 4.215686 | 0.513138 | -3.03835 | 1.58E-30 | 1.03E-28 |
| RP11-320N21.2 | 18.35294 | 2.238022 | -3.03572 | 1.54E-21 | 1.32E-20 |
| PTGDR2 | 460.8431 | 56.20866 | -3.03541 | 8.31E-30 | 3.84E-28 |
| IGLC5 | 5.607843 | 0.684699 | -3.03391 | 2.91E-25 | 4.48E-24 |
| ST6GALNAC6 | 12048.25 | 1471.482 | -3.03348 | 7.75E-29 | 2.67E-27 |
| SMPDL3A | 5695.863 | 695.8918 | -3.03298 | 1.01E-30 | 7.32E-29 |
| B3GALT5 | 9357.392 | 1144.017 | -3.032 | 7.38E-27 | 1.55E-25 |
| CALY | 61.92157 | 7.576507 | -3.03084 | 1.32E-28 | 4.22E-27 |
| TAGLN | 72710.59 | 8896.858 | -3.0308 | 2.53E-18 | 1.50E-17 |
| CTB-118N6.2 | 11.84314 | 1.449768 | -3.03016 | 1.88E-31 | 1.90E-29 |
| EYA2 | 1172.784 | 143.7465 | -3.02834 | 4.52E-26 | 8.07E-25 |
| RP11-1166P10.8 | 77.47059 | 9.50541 | -3.02683 | 2.94E-27 | 6.70E-26 |
| CTA-929C8.7 | 5.098039 | 0.625966 | -3.02579 | 1.19E-29 | 5.23E-28 |
| AKR1B10P1 | 35 | 4.2983 | -3.02552 | 1.44E-18 | 8.78E-18 |
| CLCA1 | 80379.29 | 9878.165 | -3.02451 | 1.56E-27 | 3.83E-26 |
| IGHV3OR16-16 | 13.23529 | 1.630603 | -3.02091 | 8.62E-24 | 1.03E-22 |
| ATRNL1 | 173.2745 | 21.35703 | -3.02028 | 1.39E-25 | 2.26E-24 |
| NTN1 | 1639.118 | 202.085 | -3.01989 | 1.48E-29 | 6.32E-28 |
| FOXD3-AS1 | 28.39216 | 3.503864 | -3.01847 | 4.29E-27 | 9.48E-26 |
| IGKV1OR2-9 | 11.96078 | 1.477589 | -3.017 | 2.27E-24 | 2.97E-23 |
| DCLK1 | 600.7843 | 74.33849 | -3.01467 | 7.53E-29 | 2.60E-27 |
| PLIN4 | 3395.157 | 420.3895 | -3.01368 | 7.85E-22 | 7.00E-21 |
| IGKV1D-27 | 87.29412 | 10.81453 | -3.01291 | 2.03E-22 | 1.97E-21 |
| RP3-525N10.2 | 12.19608 | 1.511592 | -3.01228 | 4.93E-31 | 4.05E-29 |
| LAMA1 | 996.6471 | 123.5317 | -3.0122 | 1.08E-27 | 2.76E-26 |
| NUS1P2 | 19.37255 | 2.409583 | -3.00716 | 3.79E-28 | 1.09E-26 |
| SLC30A8 | 15.27451 | 1.901082 | -3.00623 | 2.52E-32 | 4.10E-30 |
| COL6A5 | 61.56863 | 7.672334 | -3.00446 | 1.21E-17 | 6.66E-17 |
| TUBAL3 | 1826.039 | 227.7481 | -3.00321 | 1.15E-28 | 3.74E-27 |
| LINC01082 | 174.3725 | 21.7527 | -3.00291 | 6.69E-31 | 5.31E-29 |
| BVES | 624.1176 | 77.86862 | -3.0027 | 4.31E-23 | 4.60E-22 |
| C10orf142 | 3.823529 | 0.477589 | -3.00106 | 5.24E-28 | 1.45E-26 |
| IGKV1OR1-1 | 8.54902 | 1.071097 | -2.99667 | 8.64E-25 | 1.22E-23 |
| FGL2 | 5865.765 | 735.0031 | -2.9965 | 1.99E-31 | 1.98E-29 |
| FAM19A4 | 12.13725 | 1.520866 | -2.99648 | 2.58E-17 | 1.37E-16 |
| SLC15A1 | 1513.392 | 189.6816 | -2.99613 | 4.79E-15 | 2.04E-14 |
| DNER | 174.5098 | 21.949 | -2.99108 | 2.36E-28 | 7.07E-27 |
| RP11-812E19.9 | 79.80392 | 10.04019 | -2.99067 | 2.13E-25 | 3.36E-24 |
| TMEM220-AS1 | 152.2745 | 19.1932 | -2.98801 | 1.27E-31 | 1.40E-29 |
| PKNOX2 | 161.4706 | 20.3864 | -2.98559 | 9.98E-31 | 7.26E-29 |
| MYPN | 101.2549 | 12.7898 | -2.98493 | 1.75E-25 | 2.80E-24 |
| CD300LG | 64.7451 | 8.187017 | -2.98336 | 2.99E-28 | 8.76E-27 |
| SORCS3 | 18.68627 | 2.363215 | -2.98316 | 1.88E-29 | 7.75E-28 |
| GRIA4 | 84.29412 | 10.66306 | -2.98281 | 4.13E-28 | 1.18E-26 |
| LINC01055 | 21.21569 | 2.684699 | -2.9823 | 2.84E-25 | 4.38E-24 |
| CTB-158E9.1 | 2.784314 | 0.352396 | -2.98205 | 4.68E-15 | 2.00E-14 |
| RP11-567C2.1 | 25 | 3.165379 | -2.98148 | 3.82E-27 | 8.55E-26 |
| CTC-296K1.3 | 48.07843 | 6.09119 | -2.98059 | 1.13E-26 | 2.29E-25 |
| EPB41L3 | 4677.902 | 593.0201 | -2.97971 | 1.29E-30 | 8.90E-29 |
| RP11-320N21.1 | 69.88235 | 8.874807 | -2.97714 | 6.10E-23 | 6.36E-22 |
| DUSP26 | 85.94118 | 10.92272 | -2.97602 | 2.70E-30 | 1.55E-28 |
| P2RY14 | 658.6078 | 83.87944 | -2.97303 | 6.80E-31 | 5.35E-29 |
| HTR3C | 32.29412 | 4.114374 | -2.97253 | 1.20E-30 | 8.39E-29 |
| IGLV4-3 | 42.56863 | 5.426584 | -2.97167 | 1.71E-23 | 1.93E-22 |
| LINC00092 | 48.70588 | 6.211747 | -2.97103 | 2.25E-31 | 2.13E-29 |
| IGHV3-74 | 5004.294 | 638.8068 | -2.96972 | 7.18E-27 | 1.52E-25 |
| LILRB5 | 856.8824 | 109.4668 | -2.9686 | 1.55E-30 | 1.01E-28 |
| LCN10 | 17.35294 | 2.217929 | -2.9679 | 5.34E-25 | 7.82E-24 |
| GNG7 | 806.5686 | 103.2009 | -2.96634 | 1.92E-31 | 1.93E-29 |
| PGM5P4-AS1 | 4.647059 | 0.595054 | -2.96523 | 2.48E-31 | 2.30E-29 |
| OSTN | 3.45098 | 0.44204 | -2.96476 | 1.41E-29 | 6.07E-28 |
| SLC2A4 | 1088.235 | 139.49 | -2.96376 | 2.65E-23 | 2.92E-22 |
| RP11-629G13.1 | 5.686275 | 0.731066 | -2.95941 | 2.65E-28 | 7.88E-27 |
| SCIN | 4548.294 | 585.0881 | -2.9586 | 1.63E-28 | 5.10E-27 |
| MT1E | 9659.078 | 1242.603 | -2.95852 | 6.16E-28 | 1.68E-26 |
| SULT1B1 | 16873.57 | 2170.963 | -2.95836 | 4.35E-30 | 2.27E-28 |
| PTN | 785.549 | 101.102 | -2.95789 | 2.38E-30 | 1.38E-28 |
| IGHV4OR15-8 | 13.03922 | 1.678516 | -2.9576 | 4.46E-22 | 4.13E-21 |
| RP11-532F6.3 | 196.902 | 25.35549 | -2.95711 | 4.59E-30 | 2.37E-28 |
| GLYATL3 | 5.901961 | 0.760433 | -2.9563 | 2.48E-38 | 1.36E-35 |
| RP11-885N19.6 | 4.882353 | 0.630603 | -2.95277 | 8.53E-24 | 1.02E-22 |
| IGKV1OR22-1 | 71.47059 | 9.242658 | -2.95097 | 1.36E-25 | 2.22E-24 |
| IGLV1-50 | 87.2549 | 11.28594 | -2.95071 | 4.51E-25 | 6.70E-24 |
| RBM20 | 148.1569 | 19.25039 | -2.94417 | 8.77E-26 | 1.47E-24 |
| DRD5 | 59.37255 | 7.715611 | -2.94394 | 4.66E-12 | 1.51E-11 |
| IGLV7-35 | 8.039216 | 1.044822 | -2.9438 | 1.92E-30 | 1.19E-28 |
| PEG3 | 407.902 | 53.11128 | -2.94113 | 5.46E-27 | 1.18E-25 |
| INA | 155.1765 | 20.21484 | -2.94042 | 1.48E-28 | 4.68E-27 |
| IGHV1OR16-3 | 15.84314 | 2.069552 | -2.93647 | 2.60E-26 | 4.88E-25 |
| RP11-88I18.3 | 5.54902 | 0.724884 | -2.93641 | 1.80E-34 | 4.85E-32 |
| IGHV3OR16-10 | 27.56863 | 3.602782 | -2.93584 | 4.28E-27 | 9.47E-26 |
| LINC00908 | 17.09804 | 2.244204 | -2.92956 | 2.28E-30 | 1.35E-28 |
| SLITRK2 | 30.23529 | 3.972179 | -2.92823 | 5.27E-29 | 1.90E-27 |
| FCER1A | 188.8431 | 24.82998 | -2.92703 | 2.01E-28 | 6.16E-27 |
| RP11-403A3.3 | 3.901961 | 0.513138 | -2.92678 | 1.26E-26 | 2.52E-25 |
| GPM6B | 987.6275 | 130.1144 | -2.92419 | 1.05E-26 | 2.14E-25 |
| RP11-867O8.5 | 9.666667 | 1.27357 | -2.92414 | 5.82E-23 | 6.08E-22 |
| LGALS9C | 1036.353 | 136.5842 | -2.92365 | 1.59E-26 | 3.10E-25 |
| VPREB3 | 174.8039 | 23.04791 | -2.92303 | 1.36E-24 | 1.86E-23 |
| ADAMTS9-AS2 | 78.39216 | 10.34158 | -2.92225 | 5.67E-27 | 1.22E-25 |
| MS4A1 | 894.5098 | 118.0263 | -2.92199 | 3.76E-22 | 3.52E-21 |
| KLF4 | 20904.9 | 2761.297 | -2.92042 | 1.43E-30 | 9.61E-29 |
| IGKV3-7 | 308.3725 | 40.79134 | -2.91834 | 6.72E-26 | 1.16E-24 |
| FABP1 | 125322 | 16601.88 | -2.91622 | 2.10E-28 | 6.39E-27 |
| PLAC9 | 493.4902 | 65.37558 | -2.9162 | 3.47E-31 | 2.98E-29 |
| PDE2A | 1003.882 | 133.1298 | -2.91468 | 1.98E-29 | 8.13E-28 |
| EMILIN3 | 84.98039 | 11.28439 | -2.9128 | 1.00E-28 | 3.34E-27 |
| SPATA4 | 4.352941 | 0.578053 | -2.91272 | 6.97E-17 | 3.55E-16 |
| FDCSP | 555.4902 | 73.78053 | -2.91245 | 7.06E-19 | 4.46E-18 |
| IGHV3OR16-15 | 21.62745 | 2.874807 | -2.91133 | 3.71E-28 | 1.07E-26 |
| RP11-459C13.1 | 12.88235 | 1.714065 | -2.9099 | 8.98E-29 | 3.04E-27 |
| RP11-77K12.7 | 40.13725 | 5.35085 | -2.9071 | 8.40E-26 | 1.42E-24 |
| KY | 43.37255 | 5.799073 | -2.90289 | 7.16E-17 | 3.64E-16 |
| BMP5 | 561.902 | 75.13601 | -2.90274 | 3.31E-29 | 1.25E-27 |
| MBNL1-AS1 | 972.2745 | 130.0866 | -2.90189 | 2.65E-26 | 4.96E-25 |
| RBMS3-AS3 | 9.980392 | 1.335394 | -2.90183 | 1.48E-30 | 9.82E-29 |
| RP11-249C24.12 | 7 | 0.936631 | -2.9018 | 1.16E-20 | 8.91E-20 |
| CTA-929C8.6 | 9.54902 | 1.279753 | -2.89949 | 1.04E-27 | 2.69E-26 |
| SFTA1P | 37.15686 | 4.979907 | -2.89944 | 1.07E-26 | 2.18E-25 |
| GALNT16 | 330.2549 | 44.26893 | -2.89921 | 5.24E-28 | 1.45E-26 |
| KRT1 | 61.76471 | 8.281298 | -2.89885 | 1.40E-21 | 1.21E-20 |
| RP5-965F6.2 | 11.19608 | 1.502318 | -2.89773 | 9.71E-31 | 7.08E-29 |
| CXCL12 | 6924.784 | 929.2226 | -2.89767 | 4.73E-30 | 2.43E-28 |
| RP11-76C10.4 | 4.568627 | 0.613601 | -2.89639 | 3.06E-10 | 8.35E-10 |
| CDH18 | 3.529412 | 0.474498 | -2.89495 | 3.76E-30 | 2.00E-28 |
| CNTNAP3 | 92.29412 | 12.40958 | -2.89478 | 9.06E-27 | 1.87E-25 |
| KCNA5 | 107.7451 | 14.5085 | -2.89265 | 4.67E-21 | 3.79E-20 |
| AC093642.1 | 29.11765 | 3.925811 | -2.89083 | 7.29E-26 | 1.25E-24 |
| IGSF10 | 393.451 | 53.05873 | -2.89052 | 3.57E-27 | 8.04E-26 |
| ENHO | 135.7059 | 18.32148 | -2.88888 | 2.83E-30 | 1.61E-28 |
| AP000892.6 | 1282.176 | 173.2179 | -2.88793 | 2.44E-16 | 1.18E-15 |
| IGLV2-33 | 36.92157 | 4.993818 | -2.88625 | 3.46E-24 | 4.40E-23 |
| PDE9A | 6125.941 | 829.2303 | -2.88509 | 1.44E-30 | 9.63E-29 |
| TMEM179 | 82.64706 | 11.18856 | -2.88494 | 2.32E-27 | 5.40E-26 |
| CILP | 1725.765 | 233.6337 | -2.88492 | 3.87E-26 | 7.04E-25 |
| MRGPRF-AS1 | 14.45098 | 1.958269 | -2.88352 | 2.26E-21 | 1.90E-20 |
| GRIN2A | 60.11765 | 8.157651 | -2.88156 | 1.25E-28 | 4.02E-27 |
| RP11-238K6.1 | 2.960784 | 0.401855 | -2.88123 | 2.29E-29 | 9.08E-28 |
| PPP1R12B | 17260.76 | 2343.658 | -2.88066 | 1.50E-24 | 2.03E-23 |
| CTC-490G23.2 | 186.3137 | 25.34003 | -2.87824 | 3.48E-14 | 1.37E-13 |
| PPP1R14A | 1545.157 | 210.1592 | -2.8782 | 6.05E-26 | 1.06E-24 |
| HNRNPA1P33 | 10 | 1.360124 | -2.87819 | 2.07E-25 | 3.27E-24 |
| TNS1 | 27680.59 | 3769.941 | -2.87626 | 8.36E-22 | 7.41E-21 |
| GPAT3 | 2056.863 | 280.1917 | -2.87596 | 2.15E-31 | 2.07E-29 |
| IGKV1-17 | 2470.569 | 336.6754 | -2.87541 | 1.32E-24 | 1.81E-23 |
| NUPR2 | 15.64706 | 2.132921 | -2.87499 | 2.75E-33 | 5.99E-31 |
| RP11-124N19.3 | 7.882353 | 1.075734 | -2.8733 | 1.68E-30 | 1.07E-28 |
| LEXM | 50.72549 | 6.924266 | -2.87298 | 4.00E-29 | 1.49E-27 |
| IGHV3OR16-8 | 77.31373 | 10.55951 | -2.87218 | 7.65E-25 | 1.09E-23 |
| MIR137HG | 2.568627 | 0.35085 | -2.87207 | 7.35E-26 | 1.26E-24 |
| IGKV2D-28 | 43.07843 | 5.890263 | -2.87056 | 8.68E-28 | 2.31E-26 |
| AL117187.1 | 4.117647 | 0.564142 | -2.86769 | 1.35E-24 | 1.84E-23 |
| CFL2 | 3176.137 | 435.609 | -2.86617 | 4.27E-24 | 5.36E-23 |
| RP5-1024C24.1 | 3.019608 | 0.414219 | -2.86589 | 1.52E-18 | 9.25E-18 |
| IGHV3-6 | 17.21569 | 2.36476 | -2.86396 | 3.05E-27 | 6.93E-26 |
| WDR17 | 87.84314 | 12.0711 | -2.86337 | 1.45E-26 | 2.84E-25 |
| BLK | 250.451 | 34.42813 | -2.86287 | 1.17E-25 | 1.92E-24 |
| F13A1 | 4123.882 | 568.4668 | -2.85886 | 7.68E-27 | 1.61E-25 |
| IGHV3-75 | 24.98039 | 3.443586 | -2.85881 | 8.42E-23 | 8.54E-22 |
| PLCXD3 | 101.7647 | 14.02937 | -2.85872 | 1.09E-27 | 2.79E-26 |
| RP11-719K4.3 | 15.2549 | 2.103555 | -2.85837 | 1.85E-10 | 5.16E-10 |
| BEND4 | 55.03922 | 7.591963 | -2.85792 | 1.73E-28 | 5.40E-27 |
| PHLPP2 | 5170.765 | 713.8006 | -2.85678 | 5.69E-29 | 2.03E-27 |
| AC009133.17 | 27.31373 | 3.774343 | -2.85533 | 6.52E-28 | 1.77E-26 |
| NWD2 | 95.45098 | 13.19474 | -2.8548 | 3.07E-25 | 4.70E-24 |
| GCSAML | 61.94118 | 8.567233 | -2.854 | 2.53E-29 | 9.88E-28 |
| RP11-126O1.6 | 5.313725 | 0.735703 | -2.85253 | 4.45E-26 | 7.97E-25 |
| AC073283.4 | 279.5294 | 38.73416 | -2.85132 | 2.05E-29 | 8.34E-28 |
| MYH2 | 12.41176 | 1.720247 | -2.85102 | 4.82E-14 | 1.87E-13 |
| TP53INP2 | 11652 | 1617.926 | -2.84836 | 3.86E-31 | 3.29E-29 |
| LIX1 | 27.33333 | 3.795981 | -2.84812 | 1.57E-17 | 8.55E-17 |
| C1QTNF7 | 386.6275 | 53.69706 | -2.84803 | 3.03E-28 | 8.89E-27 |
| MKX-AS1 | 2.803922 | 0.38949 | -2.84779 | 2.73E-15 | 1.19E-14 |
| RP11-876N24.1 | 9.980392 | 1.387944 | -2.84615 | 2.47E-17 | 1.32E-16 |
| IGHV1-58 | 559.5294 | 77.8238 | -2.84593 | 1.26E-22 | 1.25E-21 |
| CDHR5 | 30680.04 | 4275.082 | -2.84328 | 8.37E-29 | 2.85E-27 |
| CHST5 | 1217.196 | 169.7496 | -2.84208 | 8.29E-26 | 1.40E-24 |
| ZNF728 | 7.784314 | 1.088099 | -2.83876 | 3.72E-27 | 8.36E-26 |
| DPEP3 | 21.21569 | 2.965997 | -2.83854 | 7.96E-26 | 1.35E-24 |
| IGKV1OR22-5 | 36.11765 | 5.055641 | -2.83674 | 7.78E-23 | 7.95E-22 |
| LRMP | 891.1176 | 124.7481 | -2.8366 | 2.86E-28 | 8.48E-27 |
| RP11-416N2.3 | 3.686275 | 0.516229 | -2.83608 | 1.55E-26 | 3.03E-25 |
| RP11-415C15.2 | 4.745098 | 0.664606 | -2.83587 | 2.21E-31 | 2.12E-29 |
| CD79A | 2325.157 | 326.1901 | -2.83354 | 3.66E-26 | 6.69E-25 |
| SLC14A2 | 120.2157 | 16.86862 | -2.83321 | 2.45E-12 | 8.12E-12 |
| PGR | 290 | 40.70634 | -2.83273 | 1.56E-25 | 2.51E-24 |
| HAPLN1 | 653 | 91.71097 | -2.83192 | 9.50E-30 | 4.32E-28 |
| VIT | 124.0392 | 17.43122 | -2.83105 | 5.44E-29 | 1.95E-27 |
| P2RY1 | 2080 | 292.306 | -2.83103 | 9.12E-32 | 1.06E-29 |
| RP11-100L22.1 | 4.431373 | 0.622875 | -2.83074 | 2.07E-30 | 1.26E-28 |
| SDCBP2 | 12605.29 | 1773.671 | -2.82922 | 1.11E-29 | 4.92E-28 |
| HRK | 49.52941 | 6.976816 | -2.82764 | 9.11E-29 | 3.08E-27 |
| C15orf48 | 29905.59 | 4216.994 | -2.82613 | 1.13E-30 | 7.95E-29 |
| RP11-479A21.1 | 6.117647 | 0.863988 | -2.82389 | 2.63E-27 | 6.05E-26 |
| LYNX1 | 763.6471 | 107.8872 | -2.82338 | 3.66E-26 | 6.69E-25 |
| CNTN1 | 439.8039 | 62.17156 | -2.82253 | 6.82E-28 | 1.85E-26 |
| HTR3A | 79.31373 | 11.21793 | -2.82176 | 1.53E-26 | 2.99E-25 |
| S100B | 650.7843 | 92.04791 | -2.82172 | 5.36E-27 | 1.16E-25 |
| LINC00940 | 79.70588 | 11.29521 | -2.81898 | 1.00E-26 | 2.05E-25 |
| HOXD1 | 124.8039 | 17.69861 | -2.81796 | 1.14E-28 | 3.73E-27 |
| MFAP5 | 2231.275 | 316.493 | -2.81762 | 1.17E-26 | 2.36E-25 |
| IGKV1-16 | 2358.51 | 334.6971 | -2.81695 | 4.01E-25 | 6.00E-24 |
| IGHV1OR16-4 | 2.764706 | 0.392581 | -2.81606 | 2.17E-24 | 2.86E-23 |
| HRCT1 | 1675.451 | 237.9119 | -2.81605 | 9.29E-26 | 1.56E-24 |
| ENTPD5 | 15889.18 | 2257.779 | -2.81507 | 8.15E-30 | 3.79E-28 |
| KCTD8 | 15.21569 | 2.162287 | -2.81493 | 2.29E-20 | 1.70E-19 |
| RGS13 | 158.1569 | 22.48995 | -2.814 | 1.81E-26 | 3.49E-25 |
| CPM | 4573.824 | 650.8671 | -2.81297 | 1.38E-29 | 5.98E-28 |
| KIF1A | 471.3333 | 67.21329 | -2.80993 | 1.82E-28 | 5.63E-27 |
| ARHGAP20 | 249.3922 | 35.57342 | -2.80954 | 2.05E-30 | 1.26E-28 |
| GNG8 | 21.13725 | 3.017002 | -2.8086 | 1.32E-29 | 5.75E-28 |
| NAP1L6 | 11.21569 | 1.601236 | -2.80826 | 8.05E-23 | 8.20E-22 |
| SEC14L5 | 134.1961 | 19.16074 | -2.80812 | 9.59E-28 | 2.52E-26 |
| CD22 | 829.5294 | 118.5162 | -2.80721 | 6.08E-23 | 6.34E-22 |
| BCAS1 | 11010.69 | 1573.637 | -2.80673 | 3.82E-29 | 1.43E-27 |
| RP11-554A11.5 | 5.588235 | 0.799073 | -2.80599 | 3.32E-15 | 1.44E-14 |
| NUGGC | 226.7255 | 32.44049 | -2.80508 | 1.44E-26 | 2.83E-25 |
| CTD-2269F5.1 | 42.90196 | 6.140649 | -2.80458 | 8.23E-30 | 3.82E-28 |
| RP11-640L9.2 | 97.7451 | 13.99691 | -2.80392 | 1.01E-27 | 2.61E-26 |
| AC138430.4 | 3.666667 | 0.525502 | -2.8027 | 0.000101 | 0.000161 |
| FOLR2 | 1413.804 | 202.6801 | -2.80231 | 1.09E-28 | 3.58E-27 |
| AOC3 | 6722.392 | 964.3802 | -2.8013 | 1.14E-24 | 1.58E-23 |
| IGHV3OR15-7 | 84.60784 | 12.14992 | -2.79984 | 4.77E-25 | 7.05E-24 |
| NXPH2 | 3.098039 | 0.445131 | -2.79905 | 1.48E-11 | 4.58E-11 |
| RP11-138I17.1 | 2.45098 | 0.352396 | -2.79809 | 3.43E-29 | 1.29E-27 |
| CTC-436P18.3 | 55.03922 | 7.913447 | -2.79808 | 4.44E-27 | 9.79E-26 |
| HEPACAM | 12.5098 | 1.799073 | -2.79773 | 2.51E-32 | 4.10E-30 |
| HTR7 | 62.27451 | 8.958269 | -2.79735 | 6.91E-30 | 3.29E-28 |
| CHL1 | 863.1765 | 124.2009 | -2.79698 | 3.22E-29 | 1.23E-27 |
| ATP13A4 | 274.3333 | 39.49304 | -2.79626 | 1.13E-26 | 2.29E-25 |
| HEPACAM2 | 4904.039 | 706.2179 | -2.79579 | 4.63E-27 | 1.02E-25 |
| TPM2 | 37478.86 | 5398.325 | -2.79549 | 1.24E-13 | 4.64E-13 |
| BEND5 | 142.0392 | 20.46213 | -2.79526 | 1.05E-28 | 3.46E-27 |
| LINC01475 | 29.4902 | 4.250386 | -2.79457 | 3.41E-26 | 6.28E-25 |
| GNG13 | 70.47059 | 10.15765 | -2.79445 | 3.12E-25 | 4.76E-24 |
| FAM151A | 332.6471 | 47.949 | -2.79442 | 1.87E-27 | 4.47E-26 |
| AC007392.4 | 4.921569 | 0.709428 | -2.79439 | 3.97E-24 | 5.01E-23 |
| DSCAML1 | 157.7255 | 22.76971 | -2.79223 | 5.38E-27 | 1.16E-25 |
| IGHV3-52 | 70.33333 | 10.15456 | -2.79208 | 3.11E-25 | 4.75E-24 |
| IGHJ3P | 70.13725 | 10.13447 | -2.79091 | 8.17E-25 | 1.16E-23 |
| ATP2B3 | 26.13725 | 3.777434 | -2.79063 | 3.49E-30 | 1.89E-28 |
| RP11-671J11.7 | 4.607843 | 0.666151 | -2.79017 | 2.06E-27 | 4.86E-26 |
| RELN | 447.8039 | 64.77125 | -2.78944 | 9.71E-27 | 1.99E-25 |
| MRGPRF | 2487.157 | 360.0572 | -2.7882 | 2.90E-23 | 3.19E-22 |
| NEFL | 318.6078 | 46.12828 | -2.78806 | 2.33E-28 | 7.00E-27 |
| AARD | 43.4902 | 6.299845 | -2.7873 | 1.43E-24 | 1.94E-23 |
| RP11-461O7.1 | 18.37255 | 2.666151 | -2.78472 | 2.46E-24 | 3.21E-23 |
| ALK | 40.70588 | 5.907264 | -2.78468 | 2.45E-26 | 4.61E-25 |
| HLF | 640.549 | 93.06955 | -2.78293 | 8.62E-25 | 1.22E-23 |
| RPH3A | 26.05882 | 3.786708 | -2.78276 | 5.06E-27 | 1.10E-25 |
| JPH4 | 91.43137 | 13.28903 | -2.78245 | 4.01E-30 | 2.12E-28 |
| IGHV3OR16-11 | 23.5098 | 3.418856 | -2.78168 | 1.50E-25 | 2.43E-24 |
| AF064860.7 | 20.66667 | 3.006182 | -2.7813 | 6.60E-21 | 5.25E-20 |
| RP5-887A10.1 | 22.82353 | 3.319938 | -2.78129 | 2.55E-26 | 4.78E-25 |
| RP1-278O22.1 | 241.0588 | 35.07728 | -2.78078 | 9.33E-28 | 2.45E-26 |
| IGKV2-24 | 1703.98 | 248.4266 | -2.77802 | 1.46E-25 | 2.36E-24 |
| ARL14 | 2179.627 | 318.0294 | -2.77685 | 2.48E-28 | 7.40E-27 |
| SYT9 | 13.17647 | 1.92272 | -2.77674 | 5.87E-31 | 4.72E-29 |
| MADCAM1 | 333.6471 | 48.76043 | -2.77454 | 1.38E-28 | 4.38E-27 |
| STAP1 | 118.549 | 17.3524 | -2.77228 | 6.62E-26 | 1.14E-24 |
| FXYD6 | 2824.196 | 413.6043 | -2.77152 | 8.24E-24 | 9.87E-23 |
| SLC22A18AS | 1720.843 | 252.0958 | -2.77107 | 2.33E-30 | 1.37E-28 |
| RP11-103J17.2 | 13.37255 | 1.959815 | -2.77049 | 2.41E-25 | 3.77E-24 |
| ADRB3 | 22.72549 | 3.330757 | -2.77039 | 7.56E-27 | 1.59E-25 |
| MALL | 2591.843 | 379.8903 | -2.77032 | 1.41E-29 | 6.07E-28 |
| GAS1RR | 34.01961 | 4.995363 | -2.76771 | 5.26E-22 | 4.82E-21 |
| CNTN3 | 396 | 58.17774 | -2.76696 | 3.36E-28 | 9.79E-27 |
| DCAF12L2 | 11.11765 | 1.636785 | -2.76391 | 1.20E-27 | 3.05E-26 |
| IGKV1OR2-6 | 97.58824 | 14.36785 | -2.76386 | 3.71E-25 | 5.59E-24 |
| IRF4 | 1197.608 | 176.3818 | -2.76338 | 4.04E-26 | 7.30E-25 |
| PLCL2 | 1655.922 | 243.9181 | -2.76317 | 3.24E-30 | 1.79E-28 |
| CCL13 | 563.7843 | 83.21175 | -2.76028 | 4.23E-24 | 5.32E-23 |
| FCGBP | 192175 | 28372.72 | -2.75984 | 1.51E-25 | 2.43E-24 |
| AC104699.1 | 41.52941 | 6.134467 | -2.75912 | 2.40E-25 | 3.75E-24 |
| RP11-805I24.3 | 93.54902 | 13.82071 | -2.75889 | 2.84E-30 | 1.61E-28 |
| RP11-542M13.3 | 5.627451 | 0.83153 | -2.75864 | 7.41E-26 | 1.27E-24 |
| RP11-94C24.13 | 40.54902 | 5.993818 | -2.75812 | 3.13E-26 | 5.79E-25 |
| MT1DP | 37.11765 | 5.486862 | -2.75805 | 2.45E-25 | 3.83E-24 |
| IGKV1OR2-118 | 8.196078 | 1.211747 | -2.75785 | 2.82E-30 | 1.61E-28 |
| CRHBP | 38.01961 | 5.625966 | -2.75657 | 4.85E-31 | 4.00E-29 |
| CTD-3193O13.8 | 2.960784 | 0.438949 | -2.75385 | 6.70E-11 | 1.94E-10 |
| SSTR2 | 270.2941 | 40.12519 | -2.75195 | 4.69E-29 | 1.72E-27 |
| SCUBE2 | 1125.608 | 167.1453 | -2.75153 | 1.67E-28 | 5.21E-27 |
| LINC01537 | 23.7451 | 3.531685 | -2.7492 | 2.87E-26 | 5.35E-25 |
| RP11-803B1.2 | 4.235294 | 0.630603 | -2.74766 | 5.83E-23 | 6.09E-22 |
| IGLV8-61 | 2290.314 | 341.2457 | -2.74666 | 4.25E-18 | 2.45E-17 |
| METTL7A | 11419.75 | 1701.493 | -2.74666 | 4.89E-32 | 6.66E-30 |
| CELF4 | 53.96078 | 8.043277 | -2.74606 | 2.20E-28 | 6.65E-27 |
| TREH | 464.5882 | 69.32303 | -2.74455 | 8.84E-15 | 3.67E-14 |
| PCSK5 | 3236.118 | 483.592 | -2.7424 | 5.67E-30 | 2.82E-28 |
| APOBEC3A | 198.9412 | 29.7357 | -2.74207 | 6.92E-21 | 5.48E-20 |
| RUNDC3B | 423.902 | 63.48995 | -2.73913 | 1.66E-29 | 6.96E-28 |
| LINC00582 | 21.03922 | 3.151468 | -2.73898 | 5.99E-26 | 1.05E-24 |
| IGHV1OR16-1 | 20.13725 | 3.018547 | -2.73794 | 1.23E-23 | 1.43E-22 |
| STOX2 | 224.2745 | 33.64915 | -2.73662 | 4.00E-29 | 1.49E-27 |
| NEU4 | 2398.157 | 360.1607 | -2.73521 | 8.17E-28 | 2.18E-26 |
| IGHV3-65 | 14.11765 | 2.120556 | -2.73498 | 3.51E-25 | 5.32E-24 |
| IGKV2OR22-3 | 39.5098 | 5.936631 | -2.73449 | 1.37E-21 | 1.19E-20 |
| PRKCB | 1454.647 | 218.7481 | -2.73333 | 6.24E-29 | 2.20E-27 |
| DHRS11 | 8827.51 | 1328.971 | -2.7317 | 1.06E-30 | 7.53E-29 |
| RP13-672B3.2 | 8.078431 | 1.216383 | -2.73148 | 1.46E-27 | 3.58E-26 |
| XXbac-B476C20.9 | 379.6667 | 57.20247 | -2.73058 | 2.83E-29 | 1.10E-27 |
| RP11-400K9.3 | 6.039216 | 0.910355 | -2.72986 | 4.68E-20 | 3.35E-19 |
| LIMS2 | 4775.706 | 720.1917 | -2.72926 | 1.82E-22 | 1.78E-21 |
| IGKV1-27 | 1793.294 | 270.5595 | -2.72859 | 4.06E-26 | 7.34E-25 |
| LMNTD1 | 4.333333 | 0.653787 | -2.72859 | 4.51E-15 | 1.93E-14 |
| BAALC | 128.7843 | 19.43122 | -2.72851 | 9.77E-30 | 4.41E-28 |
| RP11-294C11.5 | 3.666667 | 0.553323 | -2.72828 | 6.53E-32 | 8.37E-30 |
| IGKV1D-42 | 83.13725 | 12.55023 | -2.72778 | 2.33E-23 | 2.59E-22 |
| DACT3 | 1086.961 | 164.2519 | -2.72632 | 1.84E-23 | 2.07E-22 |
| CCL8 | 395.6471 | 59.81144 | -2.72572 | 8.29E-23 | 8.41E-22 |
| RP5-894D12.5 | 6.372549 | 0.964451 | -2.72409 | 5.08E-28 | 1.41E-26 |
| CNTNAP4 | 10.09804 | 1.528594 | -2.7238 | 6.63E-19 | 4.20E-18 |
| WSCD2 | 120.3922 | 18.22566 | -2.7237 | 1.27E-24 | 1.74E-23 |
| CYP4F25P | 5.45098 | 0.825348 | -2.72344 | 1.03E-20 | 8.00E-20 |
| RP11-342A23.2 | 5.058824 | 0.766615 | -2.72223 | 1.74E-25 | 2.78E-24 |
| TSPAN1 | 75422.33 | 11434.05 | -2.72166 | 1.64E-29 | 6.92E-28 |
| EFHC2 | 109.3333 | 16.58423 | -2.72085 | 2.94E-29 | 1.13E-27 |
| ADGRL3 | 518 | 78.60433 | -2.72027 | 3.74E-30 | 2.00E-28 |
| RP11-182J1.14 | 24.5098 | 3.720247 | -2.71989 | 3.76E-22 | 3.51E-21 |
| IGLV4-60 | 682.451 | 103.7342 | -2.71783 | 2.03E-23 | 2.27E-22 |
| MEP1A | 26986.16 | 4103.628 | -2.71725 | 7.47E-26 | 1.28E-24 |
| HSPB7 | 1651.49 | 251.1561 | -2.71711 | 1.96E-19 | 1.30E-18 |
| FILIP1 | 735.5098 | 111.8655 | -2.71698 | 2.96E-22 | 2.81E-21 |
| KIF5A | 373.1176 | 56.76662 | -2.71652 | 8.21E-26 | 1.39E-24 |
| PNLIPRP2 | 761.1373 | 115.8439 | -2.71597 | 2.10E-15 | 9.28E-15 |
| GATA5 | 71.41176 | 10.88253 | -2.71415 | 0.004361 | 0.005755 |
| JAM2 | 1243.765 | 189.6182 | -2.71354 | 4.05E-30 | 2.13E-28 |
| ENTPD8 | 4832.275 | 737.8099 | -2.71138 | 6.39E-27 | 1.36E-25 |
| IGHJ2P | 3.647059 | 0.55796 | -2.7085 | 3.13E-19 | 2.04E-18 |
| TMEM171 | 3541.216 | 541.7743 | -2.70848 | 2.00E-29 | 8.19E-28 |
| SH2D6 | 200.8039 | 30.79289 | -2.70512 | 1.50E-23 | 1.71E-22 |
| C1orf115 | 6897.647 | 1057.983 | -2.70479 | 5.09E-30 | 2.59E-28 |
| RP11-567J20.3 | 2.607843 | 0.400309 | -2.70367 | 5.16E-14 | 1.99E-13 |
| SIGLEC8 | 173.4118 | 26.64915 | -2.70204 | 3.33E-26 | 6.15E-25 |
| LINC01624 | 13.4902 | 2.074189 | -2.70129 | 1.21E-26 | 2.44E-25 |
| IGLV1-62 | 7.823529 | 1.204019 | -2.69996 | 2.79E-27 | 6.37E-26 |
| IGKV2D-24 | 100.6275 | 15.48686 | -2.69991 | 3.65E-24 | 4.63E-23 |
| TMCC3 | 5771.804 | 888.3663 | -2.6998 | 6.72E-30 | 3.22E-28 |
| ATP6V1G2 | 80.13725 | 12.33849 | -2.69931 | 2.55E-29 | 9.92E-28 |
| HSD11B2 | 29091.57 | 4479.603 | -2.69916 | 1.27E-29 | 5.54E-28 |
| LINC01571 | 7.921569 | 1.22102 | -2.6977 | 1.27E-36 | 4.90E-34 |
| PIANP | 141.0392 | 21.74034 | -2.69765 | 2.33E-30 | 1.37E-28 |
| IGKV3D-15 | 261.2745 | 40.28748 | -2.69716 | 1.21E-25 | 1.99E-24 |
| PAPPA2 | 143.7255 | 22.16383 | -2.69704 | 7.70E-23 | 7.89E-22 |
| IGKV2-28 | 88.5098 | 13.66306 | -2.69556 | 4.48E-26 | 8.02E-25 |
| SLC13A2 | 2215.725 | 342.0495 | -2.6955 | 9.77E-22 | 8.59E-21 |
| RP11-116O18.1 | 93.4902 | 14.43586 | -2.69516 | 5.38E-10 | 1.44E-09 |
| IGHV7-40 | 2.431373 | 0.37558 | -2.69458 | 4.93E-14 | 1.91E-13 |
| RP11-122C5.3 | 2.490196 | 0.384853 | -2.69388 | 2.19E-27 | 5.12E-26 |
| RP11-297B17.3 | 3.607843 | 0.55796 | -2.6929 | 1.62E-21 | 1.39E-20 |
| TMEM255A | 210.5686 | 32.58423 | -2.69204 | 9.34E-29 | 3.14E-27 |
| IGLV8OR8-1 | 9.098039 | 1.408037 | -2.69187 | 5.31E-18 | 3.03E-17 |
| DNAJB5 | 1263.902 | 195.9196 | -2.68955 | 1.98E-21 | 1.68E-20 |
| ITM2C | 113326.5 | 17571.57 | -2.68917 | 7.43E-31 | 5.75E-29 |
| RP4-651E10.4 | 18.47059 | 2.863988 | -2.68913 | 2.23E-22 | 2.15E-21 |
| IGHV4-4 | 194.2353 | 30.12828 | -2.68861 | 1.44E-21 | 1.24E-20 |
| C8orf46 | 29.45098 | 4.57187 | -2.68746 | 9.71E-26 | 1.62E-24 |
| ADRA1A | 25.66667 | 3.984544 | -2.68741 | 6.62E-29 | 2.32E-27 |
| CLEC17A | 63.23529 | 9.822257 | -2.6866 | 9.19E-22 | 8.11E-21 |
| FER1L6 | 1424.157 | 221.3199 | -2.6859 | 2.90E-25 | 4.47E-24 |
| RP11-598D12.1 | 2.862745 | 0.445131 | -2.6851 | 2.22E-24 | 2.92E-23 |
| RP11-358D17.2 | 29.62745 | 4.613601 | -2.68297 | 3.30E-21 | 2.72E-20 |
| ACKR2 | 219.1176 | 34.12674 | -2.68273 | 2.50E-29 | 9.78E-28 |
| FAM189A2 | 231.4706 | 36.10665 | -2.68049 | 1.58E-28 | 4.98E-27 |
| CD36 | 2267.922 | 353.8547 | -2.68014 | 6.88E-30 | 3.28E-28 |
| OR2S1P | 43.52941 | 6.800618 | -2.67825 | 1.11E-28 | 3.66E-27 |
| SLIT3 | 3632.098 | 567.4807 | -2.67816 | 1.82E-27 | 4.36E-26 |
| RP11-380I10.4 | 2.333333 | 0.36476 | -2.67737 | 1.68E-22 | 1.65E-21 |
| XDH | 6018.627 | 942.5703 | -2.67476 | 1.81E-26 | 3.48E-25 |
| CD209 | 1418.725 | 222.3246 | -2.67386 | 1.49E-28 | 4.70E-27 |
| IGKV3D-20 | 958.1765 | 150.2195 | -2.67322 | 1.85E-24 | 2.47E-23 |
| RP11-349K16.1 | 416.7843 | 65.36012 | -2.67282 | 2.38E-28 | 7.13E-27 |
| IGLV4-69 | 2866.922 | 449.6306 | -2.67269 | 1.63E-25 | 2.62E-24 |
| IGHV1-2 | 3436.333 | 539.3029 | -2.6717 | 3.49E-22 | 3.27E-21 |
| CDHR2 | 10594.47 | 1662.859 | -2.67157 | 1.59E-23 | 1.81E-22 |
| PHF24 | 38.66667 | 6.069552 | -2.67143 | 1.61E-27 | 3.92E-26 |
| RGMA | 1815.078 | 284.9413 | -2.6713 | 4.27E-27 | 9.45E-26 |
| IGKV2-18 | 14.35294 | 2.253478 | -2.67112 | 1.52E-26 | 2.98E-25 |
| RP11-384J4.2 | 2.686275 | 0.421947 | -2.67047 | 2.10E-25 | 3.33E-24 |
| MFSD4 | 3020.686 | 475.187 | -2.66831 | 1.45E-24 | 1.96E-23 |
| RP11-88G17.6 | 8.921569 | 1.406491 | -2.6652 | 1.05E-09 | 2.74E-09 |
| KCNN3 | 836.9216 | 132.0015 | -2.66454 | 2.57E-27 | 5.91E-26 |
| CNR2 | 75.68627 | 11.94436 | -2.6637 | 2.94E-23 | 3.23E-22 |
| SEMA6A | 5818.647 | 918.3323 | -2.6636 | 4.93E-30 | 2.52E-28 |
| REEP2 | 367.2549 | 57.99691 | -2.66273 | 4.35E-25 | 6.46E-24 |
| SNTG2 | 19.11765 | 3.020093 | -2.66224 | 5.27E-29 | 1.90E-27 |
| IGHV3-19 | 82.68627 | 13.08501 | -2.65973 | 9.49E-24 | 1.12E-22 |
| IGHV3-36 | 8.588235 | 1.360124 | -2.65862 | 9.28E-22 | 8.18E-21 |
| CNTN4 | 584.8627 | 92.66615 | -2.65798 | 7.11E-30 | 3.37E-28 |
| IGKV1OR-2 | 5.784314 | 0.916538 | -2.65788 | 2.55E-19 | 1.68E-18 |
| IGHV4-59 | 4792.235 | 759.5116 | -2.65755 | 8.21E-24 | 9.84E-23 |
| RNF112 | 139.1961 | 22.06646 | -2.65719 | 4.24E-30 | 2.22E-28 |
| AF131217.1 | 49.60784 | 7.871716 | -2.65582 | 5.27E-27 | 1.14E-25 |
| GGTA1P | 554.8039 | 88.12674 | -2.65433 | 8.57E-30 | 3.95E-28 |
| CIDEC | 641.5098 | 101.9088 | -2.65419 | 2.05E-23 | 2.29E-22 |
| HMGCS2 | 45127.84 | 7176.187 | -2.65273 | 5.70E-25 | 8.31E-24 |
| CCDC152 | 330.2353 | 52.53323 | -2.65219 | 2.41E-28 | 7.19E-27 |
| IGHV3-21 | 3253.667 | 518.4436 | -2.64981 | 6.10E-24 | 7.49E-23 |
| C10orf105 | 9.078431 | 1.446677 | -2.6497 | 1.27E-28 | 4.07E-27 |
| GRIA1 | 31.05882 | 4.950541 | -2.64935 | 3.45E-26 | 6.35E-25 |
| MT1X | 4542.118 | 724.2334 | -2.64884 | 1.28E-28 | 4.11E-27 |
| VWC2 | 41.58824 | 6.633694 | -2.64829 | 2.34E-27 | 5.43E-26 |
| IGKV1-9 | 3298.451 | 526.2566 | -2.64795 | 1.41E-24 | 1.92E-23 |
| RPL15P21 | 17.03922 | 2.720247 | -2.64705 | 2.95E-27 | 6.72E-26 |
| IGHV3-11 | 3260.255 | 520.6631 | -2.64656 | 6.59E-25 | 9.49E-24 |
| SNAP25 | 183.7647 | 29.34776 | -2.64654 | 7.33E-27 | 1.54E-25 |
| TLR10 | 197.2549 | 31.51159 | -2.64611 | 2.94E-24 | 3.78E-23 |
| IGHV3-54 | 7.72549 | 1.23493 | -2.6452 | 1.06E-26 | 2.16E-25 |
| RP11-449D8.5 | 4.117647 | 0.658423 | -2.64473 | 3.52E-25 | 5.32E-24 |
| TMEM130 | 159.6471 | 25.53478 | -2.64435 | 1.52E-24 | 2.06E-23 |
| AC073900.4 | 3.666667 | 0.587326 | -2.64224 | 1.36E-21 | 1.18E-20 |
| MT2A | 13413.37 | 2148.764 | -2.64209 | 3.83E-28 | 1.10E-26 |
| CELA3B | 28.56863 | 4.578053 | -2.64163 | 2.68E-24 | 3.47E-23 |
| CPED1 | 2082.118 | 333.8485 | -2.64079 | 1.28E-25 | 2.09E-24 |
| PDZK1 | 306.9608 | 49.23957 | -2.64016 | 7.26E-09 | 1.75E-08 |
| MRGPRE | 7.137255 | 1.145286 | -2.63966 | 4.86E-22 | 4.47E-21 |
| AC109309.4 | 4.607843 | 0.74034 | -2.63783 | 3.38E-28 | 9.82E-27 |
| CA12 | 26940.1 | 4331.896 | -2.63668 | 5.53E-28 | 1.52E-26 |
| RP11-348F1.3 | 2.45098 | 0.394127 | -2.63663 | 3.32E-25 | 5.04E-24 |
| MIR497HG | 83.80392 | 13.47604 | -2.63662 | 5.66E-28 | 1.55E-26 |
| FBXL22 | 389.7059 | 62.79444 | -2.63368 | 2.95E-22 | 2.80E-21 |
| TMEM74 | 29.31373 | 4.72643 | -2.63275 | 3.24E-19 | 2.10E-18 |
| RP11-1336O20.2 | 58.13725 | 9.374034 | -2.63272 | 7.77E-24 | 9.36E-23 |
| GFI1B | 76.01961 | 12.26121 | -2.63227 | 1.29E-27 | 3.23E-26 |
| IGHV3-35 | 101.1961 | 16.3524 | -2.62958 | 2.25E-24 | 2.95E-23 |
| TRDV1 | 27.19608 | 4.398764 | -2.62823 | 3.16E-21 | 2.61E-20 |
| CASQ1 | 44.78431 | 7.244204 | -2.62809 | 3.37E-16 | 1.61E-15 |
| ACADS | 10982.65 | 1779.005 | -2.62608 | 7.13E-31 | 5.57E-29 |
| PKD1L2 | 41.68627 | 6.75425 | -2.6257 | 3.85E-26 | 7.01E-25 |
| IGKV3-20 | 16032.61 | 2599.206 | -2.62487 | 8.84E-26 | 1.49E-24 |
| THRB | 1834.941 | 297.6662 | -2.62397 | 1.20E-27 | 3.05E-26 |
| ATP1B2 | 198.2157 | 32.1592 | -2.62377 | 8.10E-29 | 2.77E-27 |
| RP11-294C11.2 | 38.15686 | 6.197836 | -2.62211 | 9.09E-27 | 1.87E-25 |
| PLA2G5 | 228.5098 | 37.17002 | -2.62004 | 2.46E-19 | 1.62E-18 |
| C1orf186 | 136.2745 | 22.17311 | -2.61963 | 1.04E-28 | 3.45E-27 |
| RP11-342A1.1 | 2.72549 | 0.443586 | -2.61923 | 1.80E-26 | 3.47E-25 |
| CYP2D6 | 440.6863 | 71.74498 | -2.6188 | 0.00046 | 0.000684 |
| TPSG1 | 1285.353 | 209.3879 | -2.61791 | 5.82E-27 | 1.25E-25 |
| IGHV3-49 | 2679.824 | 436.8501 | -2.61693 | 8.22E-25 | 1.16E-23 |
| IGLV5-45 | 813.5098 | 132.7713 | -2.61522 | 2.74E-26 | 5.10E-25 |
| CD79B | 667.6667 | 109.0618 | -2.61398 | 3.88E-26 | 7.05E-25 |
| GSG1 | 5.764706 | 0.942813 | -2.6122 | 5.27E-12 | 1.69E-11 |
| MROH2B | 8.372549 | 1.369397 | -2.61213 | 4.55E-25 | 6.74E-24 |
| CEND1 | 63.27451 | 10.3524 | -2.61166 | 1.33E-25 | 2.18E-24 |
| RP11-1100L3.4 | 8.27451 | 1.355487 | -2.60986 | 3.80E-27 | 8.52E-26 |
| IGLC7 | 603.5882 | 98.89799 | -2.60955 | 2.26E-19 | 1.49E-18 |
| LINC00488 | 12.68627 | 2.078825 | -2.60943 | 1.32E-15 | 5.93E-15 |
| LINC01013 | 22.52941 | 3.692427 | -2.60917 | 2.42E-29 | 9.50E-28 |
| PLCD1 | 3941.373 | 645.9815 | -2.60913 | 4.41E-31 | 3.67E-29 |
| DPF3 | 382.2157 | 62.65224 | -2.60895 | 3.65E-30 | 1.96E-28 |
| CES3 | 8642.745 | 1418.671 | -2.60695 | 3.55E-28 | 1.02E-26 |
| TBPL2 | 6.823529 | 1.120556 | -2.6063 | 2.73E-25 | 4.22E-24 |
| SGCA | 381.4314 | 62.63988 | -2.60627 | 2.15E-26 | 4.08E-25 |
| IGHD6-25 | 2.333333 | 0.383308 | -2.60582 | 8.21E-26 | 1.39E-24 |
| NR5A2 | 2583.314 | 424.5657 | -2.60516 | 2.67E-30 | 1.53E-28 |
| IGHV3-63 | 71.82353 | 11.80526 | -2.60503 | 1.98E-26 | 3.79E-25 |
| IGKV1OR2-2 | 3.196078 | 0.525502 | -2.60453 | 1.75E-30 | 1.10E-28 |
| IGKV3OR2-5 | 13.56863 | 2.23493 | -2.60197 | 9.58E-16 | 4.37E-15 |
| TTLL6 | 476.1176 | 78.4544 | -2.60139 | 1.04E-27 | 2.69E-26 |
| FCRL2 | 188.7451 | 31.10355 | -2.60129 | 1.87E-25 | 2.98E-24 |
| NRAP | 87.78431 | 14.47141 | -2.60076 | 1.43E-21 | 1.23E-20 |
| DISP2 | 1116.471 | 184.1685 | -2.59985 | 1.54E-27 | 3.78E-26 |
| HLX-AS1 | 3.529412 | 0.582689 | -2.59863 | 1.45E-24 | 1.96E-23 |
| ARHGEF25 | 1472.137 | 243.2164 | -2.5976 | 1.43E-21 | 1.24E-20 |
| AC078941.1 | 17.94118 | 2.964451 | -2.59744 | 1.06E-10 | 3.01E-10 |
| RP11-432I5.8 | 9.490196 | 1.570325 | -2.59538 | 4.23E-19 | 2.72E-18 |
| LIFR-AS1 | 28.43137 | 4.709428 | -2.59386 | 2.82E-26 | 5.25E-25 |
| MST1L | 163.7255 | 27.12828 | -2.59341 | 5.88E-22 | 5.35E-21 |
| MTCO3P12 | 2577.549 | 427.1376 | -2.59323 | 2.39E-05 | 4.10E-05 |
| IGLV3-1 | 3127.765 | 518.5719 | -2.59252 | 5.50E-24 | 6.80E-23 |
| IGHV3-38 | 75.84314 | 12.58887 | -2.59087 | 2.36E-24 | 3.09E-23 |
| IGHV3OR16-12 | 21.56863 | 3.581144 | -2.59044 | 6.88E-24 | 8.37E-23 |
| ITLN1 | 17509.63 | 2907.274 | -2.59041 | 2.84E-24 | 3.66E-23 |
| IGHV3-13 | 651.4314 | 108.1685 | -2.59033 | 8.55E-25 | 1.21E-23 |
| IGHVIII-51-1 | 10.09804 | 1.676971 | -2.59015 | 9.33E-22 | 8.22E-21 |
| IGLV3-9 | 1081.196 | 179.575 | -2.58997 | 2.14E-22 | 2.06E-21 |
| RDH5 | 424.6078 | 70.54096 | -2.5896 | 1.69E-31 | 1.75E-29 |
| LINC01354 | 21.07843 | 3.502318 | -2.58939 | 8.14E-26 | 1.38E-24 |
| MSRB3 | 4686.725 | 778.8207 | -2.58922 | 5.31E-21 | 4.27E-20 |
| KCNIP4 | 339.7647 | 56.5085 | -2.588 | 1.25E-27 | 3.13E-26 |
| DPP10-AS1 | 185.098 | 30.79134 | -2.58769 | 1.35E-26 | 2.68E-25 |
| GPX3 | 7050.529 | 1173.032 | -2.58749 | 1.31E-28 | 4.16E-27 |
| ZBTB7C | 4359 | 725.5873 | -2.58678 | 8.40E-28 | 2.23E-26 |
| SMPX | 190.6078 | 31.73879 | -2.58629 | 1.11E-06 | 2.16E-06 |
| MIR4269 | 3.117647 | 0.51932 | -2.58576 | 6.26E-17 | 3.21E-16 |
| RP11-266O8.1 | 3.45098 | 0.574961 | -2.58547 | 7.41E-25 | 1.06E-23 |
| EML1 | 1867.608 | 311.1855 | -2.58534 | 9.69E-23 | 9.77E-22 |
| RP13-514E23.1 | 11.64706 | 1.941267 | -2.58489 | 6.88E-28 | 1.86E-26 |
| CTD-2089N3.2 | 2.529412 | 0.421947 | -2.58367 | 4.52E-21 | 3.67E-20 |
| ABCC8 | 69.41176 | 11.58114 | -2.5834 | 1.58E-27 | 3.85E-26 |
| IGLV7-46 | 2187.843 | 365.1731 | -2.58286 | 1.03E-25 | 1.71E-24 |
| LINC00844 | 2.803922 | 0.468315 | -2.58189 | 2.87E-38 | 1.55E-35 |
| MB | 662.5294 | 110.6955 | -2.58139 | 2.04E-26 | 3.88E-25 |
| SIGLEC6 | 63.31373 | 10.58578 | -2.58039 | 1.23E-27 | 3.11E-26 |
| IGHV3-71 | 82.41176 | 13.78053 | -2.58022 | 6.77E-24 | 8.24E-23 |
| ISX | 4954.196 | 828.6692 | -2.57978 | 8.70E-27 | 1.80E-25 |
| PIK3C2G | 15.41176 | 2.579598 | -2.57881 | 1.10E-34 | 3.10E-32 |
| SSTR3 | 36.31373 | 6.078825 | -2.57865 | 1.21E-27 | 3.05E-26 |
| IGHV1OR15-2 | 132.9804 | 22.26275 | -2.57851 | 7.22E-24 | 8.76E-23 |
| FOXF2 | 1238.216 | 207.5904 | -2.57645 | 2.01E-29 | 8.20E-28 |
| IGHV1OR15-3 | 2.764706 | 0.463679 | -2.57593 | 1.27E-22 | 1.26E-21 |
| IGHV1-14 | 35.84314 | 6.018547 | -2.57421 | 4.86E-22 | 4.47E-21 |
| HMCN2 | 3307.549 | 555.4127 | -2.57413 | 2.26E-26 | 4.28E-25 |
| UNC5C | 653.2745 | 109.7651 | -2.57327 | 1.42E-29 | 6.09E-28 |
| RP11-400K9.4 | 171.5686 | 28.91499 | -2.5689 | 3.53E-24 | 4.49E-23 |
| RP11-350G8.5 | 14.39216 | 2.426584 | -2.56828 | 1.56E-29 | 6.60E-28 |
| CEACAM1 | 51478.25 | 8682.479 | -2.56778 | 7.89E-26 | 1.34E-24 |
| RP11-708H21.1 | 3.176471 | 0.536321 | -2.56625 | 1.94E-13 | 7.11E-13 |
| CYP4F24P | 16.41176 | 2.771252 | -2.56612 | 1.64E-09 | 4.20E-09 |
| CCL28 | 5720.667 | 966.0355 | -2.56604 | 1.14E-27 | 2.92E-26 |
| CRYAB | 2567.941 | 434.0232 | -2.56477 | 5.21E-24 | 6.47E-23 |
| MAPT | 421.4902 | 71.23957 | -2.56475 | 4.09E-22 | 3.81E-21 |
| XXyac-YM21GA2.4 | 10.27451 | 1.737249 | -2.56419 | 8.77E-27 | 1.81E-25 |
| RP11-788M5.3 | 4.803922 | 0.812983 | -2.56292 | 6.48E-23 | 6.73E-22 |
| CD160 | 107.3529 | 18.16847 | -2.56285 | 1.59E-26 | 3.09E-25 |
| GPR26 | 5.784314 | 0.979907 | -2.56143 | 7.88E-22 | 7.03E-21 |
| HIF3A | 496.6667 | 84.16229 | -2.56103 | 6.90E-24 | 8.38E-23 |
| RP11-963H4.3 | 42.23529 | 7.157651 | -2.56089 | 1.11E-27 | 2.85E-26 |
| IGHV2OR16-5 | 3.764706 | 0.638331 | -2.56016 | 3.75E-24 | 4.75E-23 |
| RP5-875O13.1 | 9.784314 | 1.659969 | -2.55931 | 2.76E-20 | 2.04E-19 |
| RBM24 | 276.3137 | 46.87944 | -2.55928 | 4.25E-23 | 4.54E-22 |
| TRHDE | 429.8824 | 73.00309 | -2.55791 | 2.91E-25 | 4.47E-24 |
| HHATL | 4.529412 | 0.769706 | -2.55694 | 3.24E-29 | 1.23E-27 |
| IGHV7-27 | 21.68627 | 3.686244 | -2.55656 | 2.13E-23 | 2.38E-22 |
| HAGLR | 2197.275 | 373.5889 | -2.55619 | 2.39E-28 | 7.17E-27 |
| GPER1 | 573.3529 | 97.48995 | -2.5561 | 4.83E-28 | 1.35E-26 |
| TNNT3 | 26.03922 | 4.429675 | -2.55541 | 3.31E-27 | 7.48E-26 |
| C9orf135 | 4.215686 | 0.717156 | -2.55541 | 9.06E-24 | 1.08E-22 |
| IGHV3-66 | 540.7255 | 91.99227 | -2.55531 | 6.42E-22 | 5.80E-21 |
| CAP2 | 1025.863 | 174.6337 | -2.55443 | 6.23E-18 | 3.53E-17 |
| SLC16A9 | 2522.941 | 429.6321 | -2.55393 | 5.83E-25 | 8.48E-24 |
| FAM47DP | 2.196078 | 0.374034 | -2.55369 | 9.18E-19 | 5.73E-18 |
| RP11-295P22.2 | 76.84314 | 13.09737 | -2.55264 | 5.36E-24 | 6.65E-23 |
| RP11-129J12.1 | 9.137255 | 1.55796 | -2.5521 | 7.79E-26 | 1.33E-24 |
| TRPC7 | 23.29412 | 3.97527 | -2.55084 | 2.90E-27 | 6.61E-26 |
| ATP6V0D2 | 256.6863 | 43.83308 | -2.54991 | 1.91E-22 | 1.86E-21 |
| GNG3 | 59.62745 | 10.18238 | -2.5499 | 3.88E-28 | 1.11E-26 |
| CCL19 | 879.8039 | 150.4699 | -2.54771 | 1.81E-23 | 2.04E-22 |
| RP11-1069G10.1 | 172.8431 | 29.60124 | -2.54573 | 9.31E-26 | 1.56E-24 |
| PRELP | 4539.176 | 777.5317 | -2.54546 | 4.06E-23 | 4.35E-22 |
| IGLV2-14 | 8827.922 | 1512.291 | -2.54534 | 3.60E-24 | 4.58E-23 |
| ABCA6 | 324.2157 | 55.62906 | -2.54304 | 2.41E-27 | 5.57E-26 |
| MAOB | 2019.137 | 346.459 | -2.54298 | 5.40E-24 | 6.70E-23 |
| RP4-568B10.1 | 11.96078 | 2.054096 | -2.54174 | 1.11E-18 | 6.86E-18 |
| PBLD | 4741.196 | 814.7774 | -2.54077 | 1.73E-27 | 4.18E-26 |
| RP11-1101H11.1 | 2.254902 | 0.387944 | -2.53914 | 1.95E-32 | 3.34E-30 |
| WDR78 | 682.0392 | 117.3709 | -2.53878 | 1.68E-27 | 4.07E-26 |
| RP11-361C13.1 | 2.235294 | 0.384853 | -2.53808 | 2.49E-27 | 5.75E-26 |
| GCNT4 | 363.4706 | 62.60278 | -2.53754 | 8.78E-28 | 2.32E-26 |
| BTNL2 | 2.647059 | 0.455951 | -2.53744 | 1.11E-18 | 6.86E-18 |
| COL4A6 | 241.6471 | 41.6507 | -2.53649 | 4.45E-28 | 1.25E-26 |
| SULT1A1 | 4083.745 | 704.0773 | -2.53609 | 1.94E-27 | 4.61E-26 |
| RND2 | 125.1961 | 21.59505 | -2.53542 | 1.95E-23 | 2.20E-22 |
| LL22NC03-88E1.18 | 6.54902 | 1.12983 | -2.53517 | 1.68E-24 | 2.25E-23 |
| SIGLEC11 | 53.09804 | 9.163833 | -2.53464 | 1.20E-26 | 2.41E-25 |
| RP11-448G15.1 | 14.27451 | 2.465224 | -2.53365 | 2.19E-24 | 2.88E-23 |
| OSR1 | 294.1569 | 50.82998 | -2.53283 | 6.06E-27 | 1.29E-25 |
| IGHV3-60 | 53.21569 | 9.196291 | -2.53273 | 4.19E-25 | 6.25E-24 |
| TLL1 | 172.2157 | 29.77434 | -2.53207 | 4.26E-27 | 9.45E-26 |
| ZNF676 | 14.78431 | 2.556414 | -2.53187 | 9.80E-28 | 2.56E-26 |
| MAFA | 17.09804 | 2.956723 | -2.53176 | 4.21E-23 | 4.49E-22 |
| GCNT3 | 20993.25 | 3630.394 | -2.53173 | 4.60E-28 | 1.29E-26 |
| ARHGAP44 | 4026.627 | 696.8547 | -2.53064 | 5.69E-28 | 1.56E-26 |
| CNGA3 | 185.2157 | 32.07728 | -2.52958 | 2.89E-30 | 1.63E-28 |
| RP11-120J1.1 | 6.294118 | 1.09119 | -2.5281 | 1.18E-18 | 7.24E-18 |
| HSPB3 | 103.3333 | 17.91499 | -2.52807 | 6.62E-23 | 6.87E-22 |
| RP11-963H4.5 | 3.372549 | 0.585781 | -2.52541 | 8.86E-30 | 4.06E-28 |
| RP11-334E6.3 | 2.215686 | 0.384853 | -2.52537 | 8.89E-18 | 4.96E-17 |
| TCF21 | 869.7255 | 151.1623 | -2.52446 | 9.11E-31 | 6.73E-29 |
| RP11-640L9.1 | 14.90196 | 2.590417 | -2.52425 | 6.04E-26 | 1.06E-24 |
| P2RX1 | 431.451 | 75.0541 | -2.52319 | 7.84E-30 | 3.69E-28 |
| RP11-470M17.2 | 5.196078 | 0.904173 | -2.52275 | 1.89E-18 | 1.13E-17 |
| UNC80 | 25.5098 | 4.44204 | -2.52176 | 1.13E-26 | 2.28E-25 |
| NOTO | 2.490196 | 0.434312 | -2.51945 | 2.87E-16 | 1.37E-15 |
| RAB9B | 232.2941 | 40.51777 | -2.51933 | 4.43E-23 | 4.71E-22 |
| RP11-13K12.5 | 64.07843 | 11.18083 | -2.51881 | 5.88E-21 | 4.70E-20 |
| CSRP1 | 50104.43 | 8746.009 | -2.51824 | 1.17E-24 | 1.62E-23 |
| RP11-109L13.1 | 188.5098 | 32.91345 | -2.51789 | 3.69E-09 | 9.13E-09 |
| TMEM155 | 24.5098 | 4.279753 | -2.51776 | 5.09E-23 | 5.37E-22 |
| RP11-345K9.2 | 2.176471 | 0.380216 | -2.5171 | 8.71E-24 | 1.04E-22 |
| PRAMENP | 47.01961 | 8.214838 | -2.51696 | 5.65E-20 | 4.01E-19 |
| RP11-526A4.1 | 29.35294 | 5.128284 | -2.51696 | 4.69E-26 | 8.36E-25 |
| ARL4D | 556.2353 | 97.18702 | -2.51686 | 1.24E-23 | 1.44E-22 |
| MYLKP1 | 2.352941 | 0.411128 | -2.5168 | 8.06E-16 | 3.70E-15 |
| SLC9A2 | 4626.627 | 808.6213 | -2.51642 | 1.90E-28 | 5.85E-27 |
| RGS9 | 216.7451 | 37.88872 | -2.51616 | 2.11E-28 | 6.43E-27 |
| IGLV1-44 | 4344.176 | 759.9614 | -2.51508 | 1.03E-24 | 1.44E-23 |
| IGKV2OR22-4 | 82.78431 | 14.4915 | -2.51415 | 2.66E-23 | 2.94E-22 |
| FGFR2 | 3504.98 | 613.609 | -2.51401 | 3.71E-29 | 1.40E-27 |
| SHISA3 | 353.3137 | 61.8949 | -2.51306 | 6.30E-27 | 1.34E-25 |
| ACKR1 | 1460.431 | 256.1082 | -2.51157 | 1.40E-27 | 3.46E-26 |
| RP11-284N8.3 | 247.902 | 43.51623 | -2.51014 | 1.09E-26 | 2.21E-25 |
| ABHD17AP6 | 7.764706 | 1.363215 | -2.50992 | 3.17E-18 | 1.86E-17 |
| IGHV5-51 | 7458.373 | 1309.881 | -2.50943 | 9.90E-24 | 1.17E-22 |
| RYR3 | 249.7059 | 43.86708 | -2.50902 | 7.31E-23 | 7.54E-22 |
| ITM2A | 1507.275 | 264.813 | -2.5089 | 8.01E-30 | 3.75E-28 |
| GDPD3 | 1984.608 | 349.051 | -2.50734 | 1.07E-28 | 3.55E-27 |
| IL1R2 | 2286.863 | 402.2751 | -2.50712 | 5.21E-25 | 7.64E-24 |
| LPAR1 | 1821.588 | 320.4946 | -2.50682 | 1.01E-31 | 1.15E-29 |
| RP11-250H24.2 | 2.352941 | 0.414219 | -2.506 | 1.72E-11 | 5.29E-11 |
| RP11-779O18.3 | 4.27451 | 0.752705 | -2.5056 | 3.21E-27 | 7.25E-26 |
| AC244250.2 | 254.2549 | 44.80216 | -2.50464 | 2.01E-11 | 6.13E-11 |
| HMGN2P47 | 2.490196 | 0.438949 | -2.50413 | 2.88E-16 | 1.38E-15 |
| LINC00473 | 34.88235 | 6.157651 | -2.50205 | 6.85E-08 | 1.51E-07 |
| AQP4 | 12.68627 | 2.239567 | -2.50198 | 8.30E-30 | 3.84E-28 |
| AC103563.3 | 4.254902 | 0.751159 | -2.50194 | 1.76E-20 | 1.33E-19 |
| SCG2 | 805.9804 | 142.3014 | -2.50179 | 4.24E-27 | 9.40E-26 |
| AC016995.3 | 20.58824 | 3.63524 | -2.5017 | 6.30E-16 | 2.92E-15 |
| SH3GL3 | 6.588235 | 1.163833 | -2.50101 | 2.02E-20 | 1.52E-19 |
| KCNA3 | 130.3137 | 23.02628 | -2.50064 | 9.31E-27 | 1.91E-25 |
| RP11-115J16.2 | 5.901961 | 1.043277 | -2.50007 | 4.30E-10 | 1.16E-09 |
| NLRP7 | 37.43137 | 6.616692 | -2.50007 | 1.46E-24 | 1.98E-23 |
| RP11-158I9.5 | 7.254902 | 1.282844 | -2.49961 | 2.60E-15 | 1.13E-14 |
| RP11-359E19.2 | 5.45098 | 0.964451 | -2.49874 | 2.45E-22 | 2.35E-21 |
| IGHV3-73 | 1327.686 | 234.9737 | -2.49834 | 5.59E-23 | 5.85E-22 |
| RP11-498E2.9 | 11.35294 | 2.010819 | -2.49721 | 1.28E-24 | 1.76E-23 |
| IGLV3-10 | 2127.706 | 377.0711 | -2.49639 | 2.25E-21 | 1.89E-20 |
| RP11-554A11.4 | 97.86275 | 17.3524 | -2.49562 | 1.44E-19 | 9.77E-19 |
| CPXM2 | 2294.686 | 407.2658 | -2.49425 | 8.95E-20 | 6.20E-19 |
| FGF16 | 2.686275 | 0.477589 | -2.49177 | 7.36E-18 | 4.14E-17 |
| CYP4F29P | 50.56863 | 8.990726 | -2.49173 | 2.20E-16 | 1.06E-15 |
| IGKV4-1 | 16565.25 | 2945.385 | -2.49163 | 1.56E-24 | 2.11E-23 |
| RP11-64B16.2 | 68.94118 | 12.26739 | -2.49054 | 4.26E-16 | 2.01E-15 |
| NXPE1 | 3759 | 668.9335 | -2.49041 | 1.50E-25 | 2.42E-24 |
| RP11-76C10.3 | 3.647059 | 0.64915 | -2.49011 | 2.75E-09 | 6.89E-09 |
| CCR10 | 109.7255 | 19.54869 | -2.48876 | 7.25E-28 | 1.95E-26 |
| LY9 | 296.5294 | 52.83771 | -2.48854 | 1.09E-26 | 2.22E-25 |
| IGHV3-22 | 22.11765 | 3.941267 | -2.48847 | 2.70E-22 | 2.57E-21 |
| CAND2 | 263.0392 | 46.87635 | -2.48835 | 3.62E-27 | 8.15E-26 |
| RP11-863P13.4 | 20.11765 | 3.588872 | -2.48686 | 2.62E-25 | 4.08E-24 |
| GDNF | 216.9412 | 38.70634 | -2.48666 | 3.42E-26 | 6.30E-25 |
| IGHV3-15 | 5286.745 | 943.2658 | -2.48664 | 3.64E-24 | 4.62E-23 |
| CTC-558O2.1 | 43.78431 | 7.814529 | -2.48618 | 6.56E-26 | 1.14E-24 |
| LINC00621 | 4.078431 | 0.727975 | -2.48605 | 1.47E-22 | 1.45E-21 |
| RP11-677M14.3 | 152.8627 | 27.30448 | -2.48503 | 3.43E-30 | 1.88E-28 |
| RP11-634B7.4 | 3.823529 | 0.683153 | -2.48462 | 3.87E-24 | 4.90E-23 |
| NBEA | 638.6863 | 114.1175 | -2.48459 | 7.41E-23 | 7.62E-22 |
| RP11-170L3.5 | 2.568627 | 0.459042 | -2.4843 | 1.86E-23 | 2.10E-22 |
| TRIM9 | 220.6078 | 39.45131 | -2.48334 | 1.73E-25 | 2.77E-24 |
| TCEAL7 | 280 | 50.07573 | -2.48324 | 4.23E-26 | 7.62E-25 |
| IGHV3-37 | 16.52941 | 2.958269 | -2.48221 | 8.15E-25 | 1.16E-23 |
| AC007386.2 | 5.215686 | 0.933539 | -2.48207 | 3.27E-20 | 2.39E-19 |
| SYNDIG1L | 18.58824 | 3.330757 | -2.48047 | 2.96E-28 | 8.70E-27 |
| RP4-668J24.2 | 19.27451 | 3.455951 | -2.47954 | 9.64E-29 | 3.23E-27 |
| IGHV3-23 | 9146.804 | 1640.416 | -2.47921 | 9.08E-25 | 1.27E-23 |
| OMD | 254.5882 | 45.67543 | -2.47868 | 2.82E-24 | 3.64E-23 |
| KIAA0125 | 352.8235 | 63.30294 | -2.4786 | 1.87E-25 | 2.98E-24 |
| CARTPT | 95.31373 | 17.10355 | -2.47839 | 3.40E-09 | 8.44E-09 |
| CCL14 | 154.0588 | 27.6507 | -2.47809 | 6.56E-30 | 3.18E-28 |
| SLC9A9 | 621.3529 | 111.5611 | -2.47758 | 6.78E-30 | 3.24E-28 |
| REP15 | 337.5686 | 60.62751 | -2.47714 | 4.07E-26 | 7.35E-25 |
| CORO2B | 258.1373 | 46.41577 | -2.47545 | 1.07E-27 | 2.75E-26 |
| ACTL6B | 10.7451 | 1.933539 | -2.47436 | 3.86E-30 | 2.05E-28 |
| GHR | 727.7059 | 131.0726 | -2.47299 | 2.93E-26 | 5.44E-25 |
| ZNF575 | 636.1373 | 114.711 | -2.47133 | 2.52E-30 | 1.46E-28 |
| SLC32A1 | 4.686275 | 0.84544 | -2.47067 | 2.07E-17 | 1.11E-16 |
| FOXP2 | 1100.255 | 198.6105 | -2.46982 | 7.29E-24 | 8.83E-23 |
| IGHV1OR15-6 | 2.901961 | 0.523957 | -2.46951 | 5.90E-18 | 3.35E-17 |
| CCDC68 | 2461.059 | 444.4668 | -2.46913 | 8.75E-29 | 2.98E-27 |
| DIRC3 | 33.29412 | 6.018547 | -2.46778 | 1.05E-25 | 1.74E-24 |
| SLC17A1 | 24.66667 | 4.459042 | -2.46776 | 2.67E-25 | 4.13E-24 |
| IGLV5-37 | 148.1569 | 26.81917 | -2.46579 | 9.34E-21 | 7.28E-20 |
| SCN4B | 412.7059 | 74.79598 | -2.46408 | 2.83E-29 | 1.10E-27 |
| LLNLF-187D8.1 | 2.607843 | 0.472952 | -2.46309 | 3.27E-20 | 2.39E-19 |
| RP11-798K3.2 | 30.13725 | 5.46677 | -2.46279 | 1.75E-16 | 8.56E-16 |
| IGKV1D-16 | 431.6275 | 78.35549 | -2.46168 | 2.64E-24 | 3.42E-23 |
| SCN3A | 128.5098 | 23.33385 | -2.46138 | 6.73E-29 | 2.35E-27 |
| MS4A8 | 1569.843 | 285.0819 | -2.46117 | 1.60E-20 | 1.21E-19 |
| LRFN5 | 72.70588 | 13.20556 | -2.46093 | 2.38E-23 | 2.65E-22 |
| CLU | 15001.55 | 2727.73 | -2.45934 | 1.41E-26 | 2.79E-25 |
| ENTPD3 | 231.6863 | 42.13292 | -2.45915 | 2.08E-27 | 4.90E-26 |
| CA10 | 27.54902 | 5.012365 | -2.45844 | 5.06E-30 | 2.58E-28 |
| PNOC | 101.1961 | 18.42504 | -2.45741 | 3.88E-26 | 7.05E-25 |
| IGKV1D-17 | 130.9412 | 23.8609 | -2.4562 | 2.94E-20 | 2.17E-19 |
| RP3-333H23.9 | 3.313725 | 0.604328 | -2.45505 | 2.97E-26 | 5.51E-25 |
| TARID | 46.03922 | 8.398764 | -2.45461 | 3.09E-29 | 1.19E-27 |
| RERG | 746.9804 | 136.2798 | -2.4545 | 8.11E-22 | 7.21E-21 |
| DIRAS2 | 33.64706 | 6.14374 | -2.45329 | 1.73E-27 | 4.18E-26 |
| IGKV1OR10-1 | 16.98039 | 3.102009 | -2.45259 | 5.71E-29 | 2.03E-27 |
| ADCY2 | 259.6078 | 47.44359 | -2.45205 | 6.80E-22 | 6.12E-21 |
| PTPRH | 8728.569 | 1595.219 | -2.45199 | 2.85E-28 | 8.44E-27 |
| AC007392.3 | 12.43137 | 2.272025 | -2.45194 | 4.75E-20 | 3.40E-19 |
| SPX | 14.68627 | 2.686244 | -2.45081 | 3.05E-23 | 3.33E-22 |
| RP11-63P12.7 | 13.07843 | 2.395672 | -2.44869 | 2.92E-16 | 1.40E-15 |
| RP11-531A24.3 | 11.01961 | 2.018547 | -2.44868 | 8.53E-30 | 3.94E-28 |
| FCRL1 | 106.7059 | 19.55178 | -2.44827 | 1.19E-22 | 1.19E-21 |
| IGKV1-6 | 1630.647 | 299.0093 | -2.44718 | 3.91E-24 | 4.95E-23 |
| CHRM4 | 51.41176 | 9.42813 | -2.44706 | 5.05E-25 | 7.44E-24 |
| KCNG3 | 50.72549 | 9.302937 | -2.44695 | 9.57E-25 | 1.34E-23 |
| AC027612.4 | 3.941176 | 0.723338 | -2.44588 | 5.67E-18 | 3.23E-17 |
| CASP5 | 1206.059 | 221.3617 | -2.44582 | 5.75E-24 | 7.08E-23 |
| NAAA | 6129.51 | 1126.634 | -2.44375 | 3.84E-29 | 1.43E-27 |
| IGFBP6 | 1777.275 | 326.7002 | -2.44363 | 4.81E-28 | 1.34E-26 |
| DKFZp779M0652 | 11.11765 | 2.047913 | -2.44062 | 2.26E-27 | 5.26E-26 |
| KIAA1683 | 403.5686 | 74.36012 | -2.44021 | 5.27E-27 | 1.14E-25 |
| PPP1R3C | 508.9608 | 93.78825 | -2.44008 | 1.82E-21 | 1.54E-20 |
| PDE7B | 289.8824 | 53.47913 | -2.43842 | 7.50E-30 | 3.53E-28 |
| CCL21 | 3404.608 | 628.1994 | -2.43819 | 2.14E-26 | 4.07E-25 |
| CTD-3193O13.1 | 6.215686 | 1.148377 | -2.43632 | 3.34E-22 | 3.14E-21 |
| RP11-473E2.4 | 2.156863 | 0.398764 | -2.43533 | 7.34E-23 | 7.56E-22 |
| STON1-GTF2A1L | 12.37255 | 2.289026 | -2.43434 | 3.19E-09 | 7.95E-09 |
| ADAM33 | 664.9608 | 123.1607 | -2.43273 | 5.81E-26 | 1.02E-24 |
| IGKV2-29 | 197.9216 | 36.6847 | -2.43168 | 3.56E-14 | 1.40E-13 |
| RP11-77K12.10 | 25.27451 | 4.686244 | -2.43118 | 1.68E-20 | 1.27E-19 |
| LUZP2 | 147.451 | 27.34467 | -2.4309 | 2.15E-28 | 6.51E-27 |
| SVOPL | 90.54902 | 16.81453 | -2.42899 | 5.01E-21 | 4.04E-20 |
| RP11-407N17.3 | 9.372549 | 1.743431 | -2.42651 | 6.31E-15 | 2.65E-14 |
| KLHL30-AS1 | 7.078431 | 1.316847 | -2.42634 | 5.11E-16 | 2.39E-15 |
| AF186192.5 | 31.90196 | 5.938176 | -2.42555 | 1.21E-27 | 3.07E-26 |
| STAB2 | 161.4118 | 30.06491 | -2.42459 | 3.96E-26 | 7.17E-25 |
| LRCH2 | 285.1176 | 53.14374 | -2.42359 | 3.56E-22 | 3.34E-21 |
| FGF10 | 146.9412 | 27.38949 | -2.42354 | 2.90E-22 | 2.75E-21 |
| MFAP4 | 8961.137 | 1671.031 | -2.42294 | 6.80E-28 | 1.84E-26 |
| FGF14-AS2 | 61.5098 | 11.47141 | -2.42277 | 1.07E-27 | 2.75E-26 |
| IGKV3D-11 | 287.9608 | 53.70479 | -2.42275 | 3.84E-24 | 4.86E-23 |
| CTD-2525P14.5 | 6.960784 | 1.299845 | -2.42091 | 1.30E-23 | 1.50E-22 |
| SERTM1 | 8.392157 | 1.567233 | -2.42082 | 6.19E-28 | 1.69E-26 |
| FAM163A | 79.86275 | 14.9289 | -2.41941 | 1.18E-25 | 1.93E-24 |
| KCTD4 | 46.96078 | 8.782071 | -2.41882 | 2.26E-27 | 5.26E-26 |
| SLCO2A1 | 4924.392 | 921.0773 | -2.41855 | 2.11E-27 | 4.96E-26 |
| IGHV3-53 | 1157.49 | 216.5348 | -2.41833 | 3.60E-23 | 3.89E-22 |
| FNDC9 | 20.05882 | 3.75425 | -2.41764 | 7.04E-24 | 8.55E-23 |
| IGLL5 | 8028.51 | 1504.631 | -2.41572 | 1.66E-24 | 2.23E-23 |
| UGP2 | 20096.69 | 3767.612 | -2.41524 | 1.05E-31 | 1.18E-29 |
| RPRM | 98.94118 | 18.55178 | -2.41501 | 2.58E-08 | 5.92E-08 |
| CADM3-AS1 | 45.58824 | 8.548686 | -2.41489 | 1.07E-25 | 1.76E-24 |
| IGHM | 39943.33 | 7490.995 | -2.41473 | 6.59E-23 | 6.83E-22 |
| LGI4 | 834.7843 | 156.6352 | -2.41399 | 2.65E-27 | 6.08E-26 |
| KCNS2 | 21.13725 | 3.969088 | -2.41291 | 2.41E-23 | 2.67E-22 |
| RP11-536C5.2 | 6 | 1.126739 | -2.41281 | 4.04E-23 | 4.33E-22 |
| RP11-440I14.2 | 18.96078 | 3.561051 | -2.41264 | 1.99E-27 | 4.71E-26 |
| PRDM6 | 345.8627 | 64.97836 | -2.41217 | 6.19E-19 | 3.93E-18 |
| FFAR1 | 2.960784 | 0.556414 | -2.41175 | 1.95E-18 | 1.17E-17 |
| TRANK1 | 6440.922 | 1211.08 | -2.41097 | 3.13E-26 | 5.79E-25 |
| KIR2DL4 | 70.90196 | 13.34312 | -2.40973 | 1.37E-23 | 1.58E-22 |
| IGKV2D-29 | 638.0784 | 120.0866 | -2.40966 | 3.82E-21 | 3.13E-20 |
| SLC35F1 | 124.3529 | 23.4034 | -2.40965 | 1.26E-25 | 2.07E-24 |
| GS1-204I12.4 | 24.35294 | 4.585781 | -2.40886 | 3.47E-20 | 2.53E-19 |
| KLB | 157 | 29.57342 | -2.40839 | 1.21E-28 | 3.90E-27 |
| KIAA1211 | 2364.627 | 445.9196 | -2.40676 | 1.45E-26 | 2.84E-25 |
| GS1-72M22.1 | 2.490196 | 0.469861 | -2.40595 | 7.90E-24 | 9.49E-23 |
| CA14 | 117.8235 | 22.24111 | -2.40533 | 1.02E-21 | 8.94E-21 |
| RP11-404E16.1 | 10.66667 | 2.01391 | -2.40504 | 2.47E-12 | 8.18E-12 |
| CLIC5 | 10576.61 | 2001.731 | -2.40156 | 1.46E-27 | 3.58E-26 |
| SALL2 | 418.1373 | 79.16074 | -2.40112 | 5.69E-25 | 8.30E-24 |
| MIR8071-2 | 18.05882 | 3.418856 | -2.40112 | 1.00E-21 | 8.78E-21 |
| LINC00402 | 20.58824 | 3.901082 | -2.39987 | 7.46E-27 | 1.57E-25 |
| MZB1 | 2558.392 | 484.9274 | -2.3994 | 1.34E-24 | 1.84E-23 |
| CCR2 | 405.7059 | 76.92272 | -2.39895 | 9.31E-27 | 1.91E-25 |
| TACR1 | 95.92157 | 18.19165 | -2.39858 | 1.09E-21 | 9.55E-21 |
| CTD-2089N3.1 | 7.058824 | 1.340031 | -2.39716 | 7.78E-20 | 5.43E-19 |
| IGKC | 184849.5 | 35095.25 | -2.397 | 3.80E-25 | 5.71E-24 |
| ASXL3 | 221.8039 | 42.11437 | -2.3969 | 1.54E-27 | 3.78E-26 |
| FLNA | 148090.3 | 28120.37 | -2.39679 | 1.96E-15 | 8.68E-15 |
| SPARCL1 | 15732.92 | 2987.709 | -2.39667 | 5.33E-25 | 7.81E-24 |
| CD19 | 297.5098 | 56.51777 | -2.39616 | 6.64E-21 | 5.28E-20 |
| MAP6 | 301.9804 | 57.37403 | -2.39599 | 7.64E-22 | 6.83E-21 |
| RHAG | 7.54902 | 1.434312 | -2.39593 | 6.57E-26 | 1.14E-24 |
| PSMA8 | 4.333333 | 0.823802 | -2.39511 | 1.36E-18 | 8.32E-18 |
| RERG-IT1 | 2.901961 | 0.551777 | -2.39487 | 8.23E-18 | 4.61E-17 |
| LY6H | 89.96078 | 17.11128 | -2.39435 | 2.96E-09 | 7.40E-09 |
| CPNE5 | 1072.529 | 204.0201 | -2.39423 | 4.33E-28 | 1.23E-26 |
| PGPEP1L | 5.45098 | 1.037094 | -2.39397 | 1.81E-20 | 1.36E-19 |
| RP11-294C11.4 | 10.56863 | 2.010819 | -2.39393 | 2.61E-22 | 2.49E-21 |
| CES2 | 41016.1 | 7806.088 | -2.39352 | 3.59E-28 | 1.04E-26 |
| RP11-832A4.7 | 62.2549 | 11.85008 | -2.39329 | 2.35E-27 | 5.44E-26 |
| IGHV1-12 | 71.47059 | 13.61051 | -2.39263 | 6.12E-23 | 6.39E-22 |
| IGLV2-23 | 6998.863 | 1332.937 | -2.39251 | 5.55E-25 | 8.11E-24 |
| KCNC1 | 21.43137 | 4.09119 | -2.38913 | 4.21E-24 | 5.30E-23 |
| RPL10L | 55.27451 | 10.55178 | -2.38913 | 4.69E-27 | 1.03E-25 |
| CAV1 | 8182.745 | 1562.462 | -2.38876 | 8.91E-25 | 1.25E-23 |
| MPEG1 | 4651.961 | 888.5054 | -2.38839 | 7.98E-28 | 2.13E-26 |
| IL2 | 4.490196 | 0.857805 | -2.38806 | 7.55E-25 | 1.08E-23 |
| RP11-470C13.4 | 10.54902 | 2.015456 | -2.38793 | 2.26E-31 | 2.13E-29 |
| IGHV4-28 | 605.8824 | 115.7867 | -2.38757 | 3.35E-22 | 3.15E-21 |
| CYP2C18 | 550.098 | 105.1638 | -2.38705 | 5.44E-17 | 2.80E-16 |
| HBA2 | 728.2353 | 139.2968 | -2.38624 | 6.86E-20 | 4.82E-19 |
| CCDC129 | 56.56863 | 10.82689 | -2.38538 | 1.04E-21 | 9.09E-21 |
| CNKSR2 | 70.37255 | 13.47913 | -2.38428 | 3.87E-27 | 8.66E-26 |
| NACAD | 276.3137 | 52.93972 | -2.38388 | 3.54E-25 | 5.35E-24 |
| ADAMTS8 | 351 | 67.31376 | -2.3825 | 6.05E-19 | 3.85E-18 |
| RP11-236L14.2 | 11.03922 | 2.117465 | -2.38223 | 6.84E-26 | 1.18E-24 |
| RASD2 | 1726.843 | 331.323 | -2.38183 | 6.27E-27 | 1.34E-25 |
| IGKV2-40 | 3.431373 | 0.658423 | -2.3817 | 1.58E-28 | 4.98E-27 |
| IGKV1-8 | 413.8039 | 79.43586 | -2.38109 | 7.84E-24 | 9.44E-23 |
| RP11-264B14.1 | 2.294118 | 0.440495 | -2.38074 | 1.50E-35 | 4.90E-33 |
| LINC01352 | 10.58824 | 2.034003 | -2.38007 | 3.68E-26 | 6.73E-25 |
| MS4A2 | 241.902 | 46.47604 | -2.37986 | 2.41E-26 | 4.55E-25 |
| EDIL3 | 4619.176 | 887.9289 | -2.37912 | 3.30E-30 | 1.82E-28 |
| HIPK4 | 16.31373 | 3.137558 | -2.37837 | 6.46E-22 | 5.83E-21 |
| CPNE8 | 1144.51 | 220.1886 | -2.37792 | 1.74E-29 | 7.27E-28 |
| FBXO32 | 8276.765 | 1594.3 | -2.37614 | 4.53E-24 | 5.68E-23 |
| ATP2B4 | 12325.8 | 2374.44 | -2.37602 | 8.00E-19 | 5.03E-18 |
| FSIP2 | 909.8627 | 175.3926 | -2.37506 | 1.23E-25 | 2.02E-24 |
| MPP2 | 317.0392 | 61.19629 | -2.37315 | 3.66E-25 | 5.53E-24 |
| TLR3 | 1830.51 | 353.3478 | -2.37308 | 1.27E-29 | 5.54E-28 |
| LMX1A | 38.31373 | 7.398764 | -2.37251 | 7.73E-29 | 2.66E-27 |
| SELENBP1 | 59338.57 | 11464 | -2.37186 | 1.07E-25 | 1.76E-24 |
| AC004637.1 | 9.117647 | 1.761978 | -2.37147 | 2.24E-19 | 1.48E-18 |
| RP11-725G5.2 | 20.05882 | 3.876352 | -2.37147 | 3.99E-24 | 5.04E-23 |
| ABCG8 | 121.7843 | 23.5425 | -2.37099 | 8.68E-05 | 0.00014 |
| FAM46C | 4744.922 | 918.6847 | -2.36874 | 1.26E-27 | 3.17E-26 |
| IGHV7-56 | 37 | 7.163833 | -2.36872 | 1.70E-20 | 1.29E-19 |
| CALN1 | 12.07843 | 2.341577 | -2.36688 | 1.42E-11 | 4.38E-11 |
| OR2W3 | 37 | 7.173107 | -2.36686 | 2.06E-23 | 2.30E-22 |
| VAT1L | 433.3333 | 84.07883 | -2.36566 | 4.96E-29 | 1.80E-27 |
| RP11-209D14.2 | 2.803922 | 0.544049 | -2.36564 | 4.56E-23 | 4.84E-22 |
| CH17-437K3.1 | 19.05882 | 3.700155 | -2.3648 | 6.23E-20 | 4.40E-19 |
| BMX | 357.7059 | 69.53168 | -2.36303 | 6.88E-29 | 2.40E-27 |
| AKAP6 | 600.5686 | 116.7944 | -2.36236 | 8.52E-22 | 7.54E-21 |
| PAPSS2 | 16284.61 | 3167.734 | -2.36199 | 6.25E-29 | 2.20E-27 |
| PDLIM3 | 4585.196 | 892.3895 | -2.36124 | 3.54E-21 | 2.91E-20 |
| RP11-296E3.2 | 40.62745 | 7.907264 | -2.3612 | 5.16E-29 | 1.87E-27 |
| TRGV4 | 13.39216 | 2.607419 | -2.36069 | 1.56E-17 | 8.52E-17 |
| SORBS1 | 11317.29 | 2205.68 | -2.35923 | 3.65E-18 | 2.12E-17 |
| ZNF835 | 57.54902 | 11.21793 | -2.35899 | 1.87E-25 | 2.98E-24 |
| LINC00163 | 4.431373 | 0.863988 | -2.35867 | 3.46E-21 | 2.84E-20 |
| KLRF2 | 3.058824 | 0.5966 | -2.35814 | 7.95E-20 | 5.54E-19 |
| RP11-452I5.2 | 274.5098 | 53.56105 | -2.3576 | 1.44E-20 | 1.10E-19 |
| SCUBE1 | 575.0196 | 112.2241 | -2.35723 | 7.44E-28 | 2.00E-26 |
| RP4-735C1.4 | 4.254902 | 0.83153 | -2.35529 | 1.50E-25 | 2.42E-24 |
| RP6-149D17.1 | 6.764706 | 1.323029 | -2.35418 | 1.84E-11 | 5.64E-11 |
| CYS1 | 465.9804 | 91.16538 | -2.35371 | 6.21E-26 | 1.08E-24 |
| SEMA3D | 285.6078 | 55.87944 | -2.35365 | 1.13E-26 | 2.28E-25 |
| IGHV1OR21-1 | 35.21569 | 6.896445 | -2.35229 | 5.97E-21 | 4.77E-20 |
| DYNC1I1 | 281.8039 | 55.18856 | -2.35225 | 8.69E-18 | 4.85E-17 |
| FMO5 | 3344.961 | 655.6507 | -2.35099 | 1.33E-27 | 3.31E-26 |
| CTXN2 | 3 | 0.588872 | -2.34894 | 7.52E-35 | 2.28E-32 |
| PLCE1 | 5129.333 | 1006.994 | -2.34872 | 1.40E-29 | 6.04E-28 |
| GPR142 | 10.15686 | 1.995363 | -2.34773 | 8.19E-26 | 1.39E-24 |
| MYO1A | 14547.14 | 2859.167 | -2.34707 | 4.92E-27 | 1.07E-25 |
| AC012507.3 | 5.078431 | 0.998454 | -2.34661 | 7.61E-25 | 1.08E-23 |
| GDNF-AS1 | 17.92157 | 3.523957 | -2.34643 | 2.68E-20 | 1.98E-19 |
| RNU6-1065P | 5.705882 | 1.122102 | -2.34625 | 5.33E-20 | 3.79E-19 |
| PCDHB1 | 4.862745 | 0.956723 | -2.3456 | 3.14E-22 | 2.97E-21 |
| LILRA4 | 43.84314 | 8.629057 | -2.34508 | 7.90E-22 | 7.04E-21 |
| IGHV1OR15-9 | 108.9216 | 21.44359 | -2.34467 | 5.67E-22 | 5.17E-21 |
| SETBP1 | 1034.176 | 203.6368 | -2.34441 | 1.19E-26 | 2.39E-25 |
| RP11-429O1.1 | 2.078431 | 0.409583 | -2.34327 | 8.54E-19 | 5.34E-18 |
| COL21A1 | 217 | 42.78671 | -2.34246 | 5.31E-19 | 3.39E-18 |
| IGLV2-18 | 685.902 | 135.4389 | -2.34036 | 2.37E-24 | 3.09E-23 |
| RP11-472K17.3 | 3.411765 | 0.673879 | -2.33996 | 3.30E-17 | 1.74E-16 |
| AF064860.5 | 4.529412 | 0.896445 | -2.33704 | 4.22E-16 | 1.99E-15 |
| KB-1448A5.1 | 6.588235 | 1.304482 | -2.33641 | 5.28E-24 | 6.56E-23 |
| GLIPR2 | 3399.353 | 673.3879 | -2.33575 | 1.44E-30 | 9.63E-29 |
| RP11-350E12.4 | 8.215686 | 1.627512 | -2.33571 | 1.95E-23 | 2.19E-22 |
| CLDN5 | 1440.333 | 285.4111 | -2.33529 | 1.82E-28 | 5.63E-27 |
| IGLC6 | 53.82353 | 10.66615 | -2.3352 | 1.65E-22 | 1.62E-21 |
| ADAMTSL1 | 798.9804 | 158.3879 | -2.3347 | 4.09E-28 | 1.17E-26 |
| AL122127.25 | 7.882353 | 1.562597 | -2.33468 | 3.62E-23 | 3.91E-22 |
| DMD | 2683.275 | 532.1808 | -2.33401 | 7.19E-20 | 5.03E-19 |
| THBS4 | 2351.569 | 466.5549 | -2.3335 | 5.52E-15 | 2.34E-14 |
| KLK15 | 234.4118 | 46.53632 | -2.33262 | 1.22E-22 | 1.21E-21 |
| IGLV1-51 | 4976.667 | 988.0479 | -2.33253 | 1.71E-24 | 2.30E-23 |
| IGHV5-78 | 68.88235 | 13.68315 | -2.33173 | 7.04E-23 | 7.27E-22 |
| LGALS4 | 203646.5 | 40478.29 | -2.33085 | 1.49E-29 | 6.34E-28 |
| MSTN | 33.07843 | 6.574961 | -2.33084 | 1.90E-20 | 1.42E-19 |
| NAT8B | 203.9216 | 40.5626 | -2.32979 | 4.42E-18 | 2.54E-17 |
| NRG3 | 13.09804 | 2.605873 | -2.32951 | 1.80E-23 | 2.03E-22 |
| DPYSL5 | 46.78431 | 9.310665 | -2.32907 | 1.13E-28 | 3.72E-27 |
| IGHV3-41 | 63.5098 | 12.63988 | -2.329 | 5.97E-22 | 5.42E-21 |
| KLRF1 | 28.35294 | 5.642968 | -2.32897 | 4.10E-27 | 9.11E-26 |
| RP11-64C12.1 | 2.490196 | 0.496136 | -2.32745 | 1.52E-15 | 6.82E-15 |
| XPNPEP2 | 3303.157 | 658.2844 | -2.32706 | 1.98E-12 | 6.61E-12 |
| CTD-2588E21.1 | 22.39216 | 4.463679 | -2.32669 | 6.08E-28 | 1.66E-26 |
| C2orf80 | 2.254902 | 0.449768 | -2.32581 | 5.05E-21 | 4.07E-20 |
| TRPC7-AS1 | 38.35294 | 7.650696 | -2.32567 | 2.99E-27 | 6.81E-26 |
| TAS1R1 | 17.21569 | 3.434312 | -2.32563 | 5.20E-25 | 7.63E-24 |
| TMIGD3 | 422.8824 | 84.37403 | -2.32539 | 5.65E-26 | 9.96E-25 |
| RP11-554D14.4 | 10.09804 | 2.015456 | -2.3249 | 6.16E-29 | 2.17E-27 |
| RP11-958F21.1 | 2.137255 | 0.426584 | -2.32486 | 3.64E-15 | 1.57E-14 |
| BARX2 | 1157.549 | 231.0417 | -2.32485 | 3.68E-25 | 5.55E-24 |
| SSBP2 | 1037.216 | 207.1345 | -2.32408 | 2.99E-30 | 1.67E-28 |
| LGALS9B | 582.8627 | 116.4142 | -2.32389 | 6.83E-23 | 7.07E-22 |
| CTD-2531D15.4 | 1.941176 | 0.387944 | -2.32301 | 1.74E-17 | 9.45E-17 |
| GRIA3 | 55.45098 | 11.08192 | -2.32301 | 2.36E-28 | 7.07E-27 |
| C5orf67 | 2.196078 | 0.438949 | -2.3228 | 7.61E-14 | 2.89E-13 |
| CTC-303L1.1 | 7.862745 | 1.57187 | -2.32255 | 6.58E-16 | 3.05E-15 |
| CYBRD1 | 8361.863 | 1671.674 | -2.32253 | 1.03E-26 | 2.10E-25 |
| MAOA | 22742.41 | 4548.014 | -2.32208 | 9.54E-30 | 4.33E-28 |
| FAM129C | 119.4706 | 23.89645 | -2.32179 | 5.45E-20 | 3.88E-19 |
| IGKV2D-18 | 7.196078 | 1.440495 | -2.32065 | 2.63E-24 | 3.42E-23 |
| FRMD3 | 1314.157 | 263.0665 | -2.32064 | 9.99E-28 | 2.60E-26 |
| NKAPL | 25.03922 | 5.01391 | -2.32018 | 1.96E-19 | 1.30E-18 |
| KLHL34 | 109.6275 | 21.97372 | -2.31876 | 1.20E-24 | 1.66E-23 |
| GAPDHP74 | 2.588235 | 0.51932 | -2.31727 | 5.61E-11 | 1.64E-10 |
| UBE2QL1 | 204.4314 | 41.02782 | -2.31694 | 8.79E-25 | 1.24E-23 |
| MIA2 | 24.21569 | 4.868624 | -2.31436 | 1.02E-10 | 2.91E-10 |
| KIRREL3 | 96.11765 | 19.34158 | -2.3131 | 1.23E-23 | 1.42E-22 |
| AC005220.3 | 2.019608 | 0.406491 | -2.31278 | 2.60E-21 | 2.17E-20 |
| CLECL1 | 49.07843 | 9.879444 | -2.31259 | 8.12E-26 | 1.38E-24 |
| LINC01016 | 11.47059 | 2.309119 | -2.31252 | 1.70E-26 | 3.29E-25 |
| PRKAR2B | 1276.49 | 257.0108 | -2.31228 | 7.87E-29 | 2.70E-27 |
| CRYBA2 | 83.72549 | 16.85935 | -2.31212 | 2.83E-25 | 4.37E-24 |
| LINC00643 | 6.647059 | 1.338485 | -2.31211 | 6.76E-18 | 3.82E-17 |
| RP11-6O2.4 | 9.843137 | 1.982998 | -2.31143 | 4.23E-19 | 2.72E-18 |
| RP11-394O4.5 | 1495.373 | 301.5595 | -2.30999 | 2.90E-17 | 1.54E-16 |
| RP3-368B9.2 | 2.843137 | 0.573416 | -2.30983 | 3.72E-09 | 9.20E-09 |
| TPSB2 | 1192.549 | 240.527 | -2.30978 | 1.58E-24 | 2.13E-23 |
| SNCB | 11.03922 | 2.230294 | -2.30733 | 2.97E-21 | 2.46E-20 |
| RP11-374M1.5 | 4.490196 | 0.907264 | -2.30718 | 6.55E-21 | 5.21E-20 |
| GGT8P | 7.294118 | 1.474498 | -2.30651 | 4.84E-21 | 3.92E-20 |
| LINC01394 | 5.27451 | 1.066461 | -2.30621 | 1.78E-23 | 2.01E-22 |
| BMP2 | 3110.49 | 628.9598 | -2.3061 | 9.51E-27 | 1.95E-25 |
| IGKV3-11 | 13502.49 | 2731.383 | -2.30552 | 2.54E-24 | 3.30E-23 |
| IGKV1D-35 | 6.901961 | 1.397218 | -2.30445 | 1.56E-22 | 1.53E-21 |
| RSPO1 | 48.39216 | 9.800618 | -2.30383 | 7.29E-27 | 1.54E-25 |
| PPP2R2B | 136.5098 | 27.65224 | -2.30354 | 1.20E-24 | 1.65E-23 |
| SUGCT | 502.8627 | 101.8748 | -2.30337 | 3.73E-27 | 8.36E-26 |
| SYNGR1 | 613.8627 | 124.3833 | -2.30312 | 4.09E-26 | 7.38E-25 |
| FLVCR2 | 1058.078 | 214.6832 | -2.30117 | 5.21E-27 | 1.13E-25 |
| AC073046.1 | 1.980392 | 0.401855 | -2.30104 | 3.66E-11 | 1.09E-10 |
| IGKV1-33 | 58.94118 | 11.96136 | -2.30089 | 4.70E-23 | 4.99E-22 |
| FAM150B | 114.6471 | 23.28748 | -2.29957 | 1.58E-28 | 4.98E-27 |
| POU2AF1 | 1701.118 | 345.7666 | -2.29861 | 5.34E-24 | 6.63E-23 |
| RP11-326K13.5 | 4.019608 | 0.81762 | -2.29755 | 1.11E-25 | 1.82E-24 |
| FENDRR | 2115.549 | 430.3957 | -2.2973 | 2.37E-27 | 5.48E-26 |
| GPR88 | 27.64706 | 5.625966 | -2.29695 | 2.93E-25 | 4.50E-24 |
| SLC36A1 | 4646.255 | 945.5147 | -2.2969 | 4.75E-26 | 8.45E-25 |
| PDCD4 | 17204.76 | 3505.743 | -2.29502 | 2.20E-30 | 1.33E-28 |
| ZNF229 | 86.29412 | 17.58423 | -2.29498 | 3.07E-26 | 5.69E-25 |
| NAT8 | 86.11765 | 17.55641 | -2.29431 | 1.22E-10 | 3.46E-10 |
| GRIA2 | 9.196078 | 1.874807 | -2.29428 | 5.54E-23 | 5.81E-22 |
| RP11-693J15.5 | 27.56863 | 5.621329 | -2.29404 | 1.60E-22 | 1.57E-21 |
| RP11-862G15.2 | 2.470588 | 0.503864 | -2.29375 | 1.63E-21 | 1.39E-20 |
| ERICH3 | 15.82353 | 3.227202 | -2.29372 | 2.86E-31 | 2.57E-29 |
| LINC00675 | 3586 | 731.9073 | -2.29264 | 4.01E-29 | 1.49E-27 |
| HCG4 | 18.70588 | 3.819165 | -2.29216 | 1.36E-13 | 5.07E-13 |
| ADRB2 | 136.1373 | 27.80062 | -2.29187 | 3.76E-29 | 1.41E-27 |
| FCRL3 | 219.8235 | 44.9119 | -2.29118 | 6.15E-20 | 4.34E-19 |
| DLGAP2 | 13.37255 | 2.732612 | -2.29092 | 2.94E-24 | 3.78E-23 |
| TRGC2 | 118.2941 | 24.18547 | -2.29017 | 1.16E-23 | 1.35E-22 |
| IGKV1-5 | 11373.63 | 2328.216 | -2.2884 | 6.76E-24 | 8.24E-23 |
| UCHL1 | 898.9216 | 184.1808 | -2.28707 | 2.95E-25 | 4.53E-24 |
| SLC28A2 | 1742.549 | 357.0355 | -2.28706 | 3.42E-15 | 1.48E-14 |
| IGHV3-29 | 2.745098 | 0.562597 | -2.28668 | 1.26E-20 | 9.63E-20 |
| IGHV3-16 | 11.56863 | 2.370943 | -2.28668 | 2.88E-23 | 3.16E-22 |
| GPA33 | 42245.55 | 8667.233 | -2.28516 | 7.98E-28 | 2.13E-26 |
| C10orf99 | 13654.57 | 2804.17 | -2.28374 | 3.90E-27 | 8.71E-26 |
| RAB3C | 146.0588 | 29.99691 | -2.28366 | 4.60E-27 | 1.01E-25 |
| IGHV3-48 | 980.4902 | 201.3895 | -2.28351 | 2.00E-22 | 1.94E-21 |
| WBSCR17 | 263.3529 | 54.12828 | -2.28254 | 1.20E-20 | 9.23E-20 |
| NCAM1-AS1 | 3 | 0.616692 | -2.28234 | 3.18E-20 | 2.33E-19 |
| POU3F3 | 36.94118 | 7.595054 | -2.2821 | 3.95E-26 | 7.16E-25 |
| MIXL1 | 49.31373 | 10.1391 | -2.28206 | 1.34E-23 | 1.53E-22 |
| IRX6 | 9.333333 | 1.919629 | -2.28156 | 6.11E-25 | 8.85E-24 |
| RETSAT | 19549.06 | 4026.509 | -2.2795 | 7.26E-29 | 2.52E-27 |
| FAM95B1 | 3.77451 | 0.777434 | -2.2795 | 4.16E-23 | 4.45E-22 |
| RP11-437J2.3 | 1.980392 | 0.408037 | -2.27901 | 2.14E-26 | 4.07E-25 |
| MAPK10 | 316.6275 | 65.26584 | -2.27839 | 3.54E-28 | 1.02E-26 |
| RP5-1185I7.1 | 98.84314 | 20.38794 | -2.27742 | 3.39E-22 | 3.18E-21 |
| ABHD3 | 5854.353 | 1208.981 | -2.27572 | 1.04E-28 | 3.45E-27 |
| AC144831.3 | 54.76471 | 11.31221 | -2.27537 | 5.58E-27 | 1.20E-25 |
| NTRK3 | 155.4314 | 32.10819 | -2.27526 | 7.96E-27 | 1.66E-25 |
| SCN4A | 49.5098 | 10.23029 | -2.27487 | 1.62E-20 | 1.22E-19 |
| CLDN11 | 297.4118 | 61.46677 | -2.27458 | 6.04E-25 | 8.76E-24 |
| SYT4 | 101.902 | 21.06337 | -2.27437 | 4.77E-29 | 1.74E-27 |
| KIAA0513 | 2732.529 | 565.2427 | -2.27329 | 2.28E-29 | 9.08E-28 |
| SH3BGR | 369.9412 | 76.60896 | -2.27171 | 1.89E-22 | 1.84E-21 |
| NLGN4X | 252.3333 | 52.2643 | -2.27143 | 3.56E-26 | 6.52E-25 |
| PLPP1 | 4155.647 | 861.6414 | -2.26991 | 6.43E-32 | 8.33E-30 |
| AP003774.6 | 4.352941 | 0.902628 | -2.26979 | 1.20E-21 | 1.04E-20 |
| CTC-558O2.2 | 6.215686 | 1.289026 | -2.26963 | 7.15E-26 | 1.23E-24 |
| TRGV2 | 9.411765 | 1.952087 | -2.26945 | 7.60E-21 | 5.99E-20 |
| KCNIP1 | 18.35294 | 3.808346 | -2.26877 | 1.27E-23 | 1.46E-22 |
| PHGR1 | 28432.96 | 5900.304 | -2.2687 | 1.70E-23 | 1.92E-22 |
| PTH1R | 96.19608 | 19.96291 | -2.26866 | 7.91E-28 | 2.12E-26 |
| FAM189A1 | 783.9804 | 162.7774 | -2.26792 | 3.85E-25 | 5.79E-24 |
| SCG3 | 169.8431 | 35.28748 | -2.26697 | 5.29E-30 | 2.65E-28 |
| RP11-454H19.2 | 7.862745 | 1.633694 | -2.2669 | 4.62E-21 | 3.75E-20 |
| ADAMTS1 | 4377.804 | 909.7079 | -2.26673 | 9.35E-20 | 6.46E-19 |
| CFC1 | 2 | 0.415765 | -2.26616 | 2.25E-16 | 1.09E-15 |
| KLRB1 | 422.5686 | 87.86708 | -2.26579 | 1.76E-27 | 4.24E-26 |
| AOC1 | 38227.65 | 7953.235 | -2.265 | 1.57E-27 | 3.83E-26 |
| RP11-115H18.1 | 3.764706 | 0.783617 | -2.26432 | 8.23E-14 | 3.12E-13 |
| ITIH5 | 3686.451 | 767.6569 | -2.2637 | 2.66E-25 | 4.13E-24 |
| TRDN | 11.23529 | 2.340031 | -2.26344 | 4.87E-29 | 1.78E-27 |
| VIPR2 | 123.0784 | 25.67543 | -2.26112 | 4.36E-19 | 2.81E-18 |
| RP11-239H6.2 | 2.862745 | 0.598145 | -2.25883 | 1.19E-23 | 1.39E-22 |
| GNG2 | 1633 | 341.2102 | -2.25879 | 2.58E-29 | 1.00E-27 |
| LINC01502 | 12.86275 | 2.68779 | -2.25871 | 2.91E-05 | 4.94E-05 |
| SEZ6L | 69.94118 | 14.62133 | -2.25807 | 8.93E-28 | 2.36E-26 |
| SNX18P9 | 2.254902 | 0.471406 | -2.25802 | 6.32E-11 | 1.84E-10 |
| IGHV3-33-2 | 3.843137 | 0.803709 | -2.25754 | 1.15E-16 | 5.73E-16 |
| CALB2 | 637.9216 | 133.8223 | -2.25306 | 6.73E-25 | 9.66E-24 |
| RP11-246A10.1 | 2.333333 | 0.489954 | -2.25168 | 5.13E-16 | 2.40E-15 |
| PPP2R3A | 1392.941 | 292.7774 | -2.25026 | 7.71E-31 | 5.88E-29 |
| CCDC158 | 45.47059 | 9.561051 | -2.24969 | 4.83E-27 | 1.05E-25 |
| AC144831.1 | 122.5686 | 25.77898 | -2.24932 | 2.30E-25 | 3.61E-24 |
| IGKV7-3 | 26.45098 | 5.567233 | -2.24829 | 2.12E-18 | 1.27E-17 |
| NPY1R | 326.4902 | 68.74343 | -2.24775 | 3.17E-25 | 4.83E-24 |
| C5orf64 | 4 | 0.842349 | -2.24751 | 2.59E-19 | 1.70E-18 |
| CTB-134H23.3 | 130.3333 | 27.46677 | -2.24645 | 5.24E-19 | 3.35E-18 |
| HHIP | 717.8235 | 151.4297 | -2.24498 | 4.23E-25 | 6.30E-24 |
| PRKACB | 8894.549 | 1876.464 | -2.24491 | 6.58E-30 | 3.18E-28 |
| FP325317.1 | 6.196078 | 1.307573 | -2.24446 | 1.01E-20 | 7.84E-20 |
| AC090044.2 | 3.529412 | 0.744977 | -2.24416 | 7.78E-11 | 2.24E-10 |
| IGHV3-47 | 53.64706 | 11.32457 | -2.24404 | 1.10E-22 | 1.10E-21 |
| IGHV1-68 | 10.39216 | 2.194745 | -2.24337 | 5.96E-20 | 4.21E-19 |
| SVIL | 13854.2 | 2926.403 | -2.24312 | 1.24E-20 | 9.49E-20 |
| CCL15-CCL14 | 121.4314 | 25.6507 | -2.24307 | 1.91E-26 | 3.67E-25 |
| RP11-387A1.5 | 11.39216 | 2.408037 | -2.24211 | 2.53E-22 | 2.42E-21 |
| IGHV1-18 | 5254.118 | 1111.594 | -2.24082 | 2.92E-22 | 2.77E-21 |
| LINC01140 | 59.33333 | 12.55641 | -2.24042 | 1.01E-27 | 2.61E-26 |
| SNCG | 372.1961 | 78.7898 | -2.23998 | 1.88E-27 | 4.49E-26 |
| RN7SL526P | 2.627451 | 0.556414 | -2.23943 | 7.74E-22 | 6.91E-21 |
| PAX5 | 576.3922 | 122.0943 | -2.23906 | 3.86E-16 | 1.83E-15 |
| RP11-77K12.5 | 138.549 | 29.38331 | -2.23733 | 6.67E-21 | 5.30E-20 |
| CHRNA3 | 413.4706 | 87.72179 | -2.23678 | 3.39E-24 | 4.32E-23 |
| CXCL13 | 1112.98 | 236.136 | -2.23674 | 6.07E-15 | 2.56E-14 |
| DEFB1 | 573.9216 | 121.7697 | -2.2367 | 1.39E-21 | 1.20E-20 |
| RP11-203J24.9 | 69.07843 | 14.65688 | -2.23666 | 1.32E-14 | 5.37E-14 |
| FAM187B2P | 8.45098 | 1.795981 | -2.23435 | 2.49E-25 | 3.87E-24 |
| FADS6 | 189.3725 | 40.26275 | -2.23371 | 8.42E-15 | 3.50E-14 |
| PGM5P3-AS1 | 2.921569 | 0.621329 | -2.23331 | 9.37E-22 | 8.25E-21 |
| GSN | 57745.33 | 12286.38 | -2.23264 | 6.37E-30 | 3.10E-28 |
| CD27 | 562.6863 | 119.9583 | -2.2298 | 3.46E-26 | 6.35E-25 |
| FAM181B | 44.11765 | 9.406491 | -2.22963 | 5.52E-29 | 1.97E-27 |
| IGHV3-62 | 39.76471 | 8.479134 | -2.2295 | 3.57E-20 | 2.59E-19 |
| IGHV4-39 | 6299.451 | 1343.464 | -2.22927 | 1.42E-17 | 7.78E-17 |
| AQP7 | 522.8431 | 111.5147 | -2.22914 | 8.89E-25 | 1.25E-23 |
| CTB-133G6.1 | 65.09804 | 13.89181 | -2.22838 | 2.58E-22 | 2.47E-21 |
| PAQR5 | 3431.922 | 732.592 | -2.22793 | 7.21E-27 | 1.52E-25 |
| TDP2 | 16112.9 | 3439.787 | -2.22783 | 9.88E-29 | 3.30E-27 |
| GPIHBP1 | 101 | 21.5626 | -2.22775 | 8.98E-24 | 1.07E-22 |
| RP11-49G2.3 | 4.039216 | 0.862442 | -2.22758 | 1.09E-19 | 7.46E-19 |
| CPA3 | 1333.902 | 285.0819 | -2.2262 | 2.24E-25 | 3.52E-24 |
| IGLV1-47 | 2917.157 | 623.5703 | -2.22594 | 6.49E-24 | 7.94E-23 |
| RP11-95I16.6 | 40.33333 | 8.625966 | -2.22521 | 2.01E-24 | 2.67E-23 |
| GRIK1-AS1 | 5.196078 | 1.111283 | -2.2252 | 1.80E-21 | 1.53E-20 |
| KCNQ5 | 55.66667 | 11.90572 | -2.22516 | 2.64E-26 | 4.93E-25 |
| SMIM6 | 479.6471 | 102.6275 | -2.22456 | 1.16E-25 | 1.90E-24 |
| IGHV3-50 | 8.333333 | 1.783617 | -2.22409 | 5.51E-22 | 5.04E-21 |
| EIF4E3 | 3558.902 | 761.7774 | -2.22399 | 6.89E-32 | 8.64E-30 |
| IL5RA | 35.15686 | 7.531685 | -2.22276 | 5.85E-25 | 8.50E-24 |
| ANKRD33B | 274.0392 | 58.73879 | -2.222 | 4.55E-26 | 8.13E-25 |
| BMP6 | 342.4118 | 73.44049 | -2.22108 | 4.00E-27 | 8.90E-26 |
| IGLV1-36 | 393.1373 | 84.35703 | -2.22045 | 1.47E-23 | 1.68E-22 |
| AC096579.13 | 16.78431 | 3.602782 | -2.21993 | 2.15E-22 | 2.07E-21 |
| RP11-361L15.4 | 2.490196 | 0.534776 | -2.21925 | 1.56E-20 | 1.18E-19 |
| CLIP3 | 1869.608 | 401.7682 | -2.2183 | 8.21E-22 | 7.29E-21 |
| RP11-542B15.1 | 15.80392 | 3.401855 | -2.21589 | 1.10E-22 | 1.10E-21 |
| RP11-526F3.1 | 2.843137 | 0.612056 | -2.21575 | 1.14E-35 | 3.87E-33 |
| SKIDA1 | 45.17647 | 9.729521 | -2.21513 | 2.53E-18 | 1.50E-17 |
| HEMGN | 5.058824 | 1.089645 | -2.21494 | 7.10E-17 | 3.61E-16 |
| RP4-798A17.5 | 12.72549 | 2.741886 | -2.21448 | 8.59E-22 | 7.60E-21 |
| MORC1 | 3.019608 | 0.650696 | -2.21431 | 3.06E-22 | 2.90E-21 |
| CLIP4 | 739.8824 | 159.507 | -2.21368 | 2.91E-24 | 3.75E-23 |
| RP11-326I11.4 | 7.490196 | 1.615147 | -2.21334 | 1.62E-20 | 1.22E-19 |
| CRTAC1 | 119.1176 | 25.69397 | -2.21289 | 3.97E-21 | 3.24E-20 |
| ANKRD35 | 160.1176 | 34.55332 | -2.21224 | 1.14E-26 | 2.31E-25 |
| IGKV1D-43 | 78.52941 | 16.94745 | -2.21216 | 3.20E-19 | 2.08E-18 |
| LINC00640 | 4.627451 | 1 | -2.21022 | 2.05E-23 | 2.30E-22 |
| CD48 | 1348.824 | 291.6491 | -2.2094 | 7.61E-26 | 1.30E-24 |
| PIGR | 481274.1 | 104113.4 | -2.2087 | 6.19E-25 | 8.96E-24 |
| IGLVI-70 | 113.5686 | 24.57187 | -2.20848 | 1.92E-16 | 9.38E-16 |
| IGHV4-31 | 1285.863 | 278.5116 | -2.20693 | 3.10E-19 | 2.02E-18 |
| IGLV1-40 | 5793.98 | 1255.569 | -2.20621 | 7.44E-23 | 7.64E-22 |
| C14orf132 | 1008.157 | 218.5889 | -2.20543 | 8.63E-26 | 1.45E-24 |
| RP11-528A4.2 | 197.7451 | 42.88717 | -2.20502 | 4.14E-26 | 7.47E-25 |
| RMDN2 | 881.2941 | 191.1391 | -2.205 | 1.65E-30 | 1.05E-28 |
| RP11-430C7.4 | 9.313725 | 2.020093 | -2.20494 | 1.12E-24 | 1.55E-23 |
| MDGA2 | 3.254902 | 0.706337 | -2.20419 | 6.05E-21 | 4.82E-20 |
| SLC1A1 | 3084.98 | 669.592 | -2.20391 | 9.56E-23 | 9.64E-22 |
| GH1 | 2.078431 | 0.451314 | -2.20329 | 8.06E-20 | 5.61E-19 |
| MIR4537 | 6.176471 | 1.341577 | -2.20285 | 9.74E-19 | 6.06E-18 |
| RP11-384O8.1 | 19.09804 | 4.149923 | -2.20227 | 1.22E-16 | 6.07E-16 |
| ABCD2 | 93.35294 | 20.28903 | -2.202 | 1.68E-26 | 3.26E-25 |
| RP11-1134I14.4 | 2.921569 | 0.63524 | -2.20137 | 2.16E-21 | 1.82E-20 |
| IGLV2-34 | 46.13725 | 10.03555 | -2.20081 | 9.54E-21 | 7.43E-20 |
| TMEM108 | 90.17647 | 19.63833 | -2.19908 | 1.29E-26 | 2.57E-25 |
| SORBS2 | 4526.529 | 985.8068 | -2.19903 | 1.25E-19 | 8.48E-19 |
| SLC22A5 | 3871.98 | 843.4992 | -2.19861 | 8.27E-27 | 1.72E-25 |
| CDC14A | 1105.294 | 240.9614 | -2.19756 | 1.54E-29 | 6.53E-28 |
| LILRP2 | 14.64706 | 3.193199 | -2.19754 | 1.20E-21 | 1.04E-20 |
| NAP1L3 | 200.4314 | 43.73107 | -2.19638 | 5.65E-24 | 6.97E-23 |
| MIER3 | 6134.235 | 1338.431 | -2.19634 | 2.54E-29 | 9.91E-28 |
| IGLV1-41 | 132.8824 | 29.01082 | -2.19549 | 2.28E-16 | 1.10E-15 |
| BFSP2 | 19.60784 | 4.281298 | -2.19531 | 1.51E-21 | 1.30E-20 |
| GGT6 | 10753.16 | 2348.008 | -2.19525 | 2.35E-27 | 5.44E-26 |
| GRIN3A | 166.6078 | 36.39413 | -2.19468 | 2.15E-23 | 2.40E-22 |
| B3GNT6 | 2691 | 588.0124 | -2.19422 | 1.42E-24 | 1.93E-23 |
| SMPD1 | 4668.431 | 1020.929 | -2.19306 | 1.22E-29 | 5.34E-28 |
| GALNT15 | 495.7843 | 108.4529 | -2.19264 | 8.48E-24 | 1.01E-22 |
| RP11-365O16.3 | 8.901961 | 1.94745 | -2.19254 | 1.59E-18 | 9.65E-18 |
| C5orf66-AS1 | 36.98039 | 8.098918 | -2.19096 | 6.26E-23 | 6.52E-22 |
| RP1-161P9.5 | 6.117647 | 1.340031 | -2.19071 | 5.20E-25 | 7.63E-24 |
| IL1RAPL1 | 8.784314 | 1.924266 | -2.19062 | 4.33E-24 | 5.44E-23 |
| RP11-203B7.2 | 8.627451 | 1.890263 | -2.19035 | 9.83E-11 | 2.81E-10 |
| AC067959.1 | 2.137255 | 0.468315 | -2.19021 | 5.11E-15 | 2.17E-14 |
| IGLV2-11 | 5060.667 | 1109.212 | -2.18979 | 5.74E-25 | 8.37E-24 |
| ASAP3 | 2071.137 | 454.0386 | -2.18954 | 1.99E-29 | 8.13E-28 |
| AC246787.4 | 22.52941 | 4.944359 | -2.18795 | 1.25E-20 | 9.56E-20 |
| RP11-770E5.1 | 4.72549 | 1.037094 | -2.18792 | 2.59E-24 | 3.36E-23 |
| KIF5C | 369.9412 | 81.2813 | -2.1863 | 6.12E-26 | 1.07E-24 |
| UCN3 | 226.7843 | 49.91654 | -2.18373 | 3.08E-25 | 4.71E-24 |
| IGHV3-43 | 431.8235 | 95.12674 | -2.18252 | 4.73E-21 | 3.83E-20 |
| PRRG3 | 72.27451 | 15.94281 | -2.18058 | 3.63E-23 | 3.92E-22 |
| PDE5A | 3819.039 | 842.8331 | -2.17989 | 3.78E-27 | 8.48E-26 |
| NEXN | 1848.353 | 407.9861 | -2.17965 | 6.45E-17 | 3.30E-16 |
| IGHV1-17 | 19 | 4.197836 | -2.17828 | 6.67E-21 | 5.29E-20 |
| LRRN4CL | 412.2353 | 91.13601 | -2.17737 | 3.95E-27 | 8.80E-26 |
| IGHV1-46 | 2961.333 | 655.1113 | -2.17643 | 2.16E-22 | 2.09E-21 |
| RP11-442O1.3 | 12.17647 | 2.693972 | -2.17629 | 1.43E-26 | 2.82E-25 |
| RCAN2 | 1390.392 | 307.6167 | -2.17629 | 3.26E-29 | 1.24E-27 |
| STBD1 | 580.7059 | 128.7295 | -2.17346 | 8.07E-30 | 3.77E-28 |
| TSHB | 2.196078 | 0.486862 | -2.17334 | 5.06E-15 | 2.15E-14 |
| SLC37A2 | 1751.098 | 388.3478 | -2.17284 | 6.75E-19 | 4.28E-18 |
| ETFDH | 3154.882 | 699.7682 | -2.17264 | 1.04E-31 | 1.18E-29 |
| KCNK10 | 266.9608 | 59.22411 | -2.17237 | 1.08E-24 | 1.50E-23 |
| RP11-295M18.6 | 5.078431 | 1.126739 | -2.17223 | 3.51E-23 | 3.80E-22 |
| CDKL2 | 75.47059 | 16.75116 | -2.17165 | 1.26E-24 | 1.73E-23 |
| FUCA1 | 13415.78 | 2978.454 | -2.1713 | 5.91E-30 | 2.92E-28 |
| RP11-731F5.1 | 4.176471 | 0.927357 | -2.17109 | 3.56E-20 | 2.59E-19 |
| RP11-20J15.3 | 4.392157 | 0.97527 | -2.17106 | 7.06E-21 | 5.59E-20 |
| SV2B | 164.098 | 36.45286 | -2.17045 | 6.18E-24 | 7.58E-23 |
| IGKV5-2 | 180.1373 | 40.04173 | -2.16952 | 2.33E-23 | 2.59E-22 |
| MIR8071-1 | 8.176471 | 1.819165 | -2.1682 | 1.01E-16 | 5.05E-16 |
| ARHGEF26-AS1 | 29.98039 | 6.672334 | -2.16776 | 1.12E-15 | 5.09E-15 |
| PLCG2 | 1744.608 | 388.4745 | -2.16701 | 4.72E-26 | 8.39E-25 |
| P3H2 | 2097.118 | 466.9907 | -2.16694 | 5.65E-24 | 6.97E-23 |
| CLMP | 2072.02 | 461.493 | -2.16666 | 8.11E-23 | 8.25E-22 |
| RP3-400B16.1 | 19 | 4.236476 | -2.16506 | 4.02E-25 | 6.01E-24 |
| RGS2 | 3406.765 | 759.7836 | -2.16474 | 4.67E-22 | 4.31E-21 |
| LGI3 | 33.11765 | 7.38949 | -2.16405 | 1.76E-24 | 2.36E-23 |
| KB-1517D11.4 | 5.333333 | 1.190108 | -2.16394 | 3.73E-24 | 4.73E-23 |
| ZNF667-AS1 | 251.3529 | 56.13138 | -2.16284 | 5.89E-25 | 8.55E-24 |
| RP11-354E11.2 | 23.43137 | 5.236476 | -2.16177 | 3.45E-24 | 4.39E-23 |
| AL035610.1 | 7.980392 | 1.785162 | -2.1604 | 5.16E-24 | 6.42E-23 |
| VLDLR-AS1 | 43.45098 | 9.720247 | -2.16032 | 1.70E-17 | 9.25E-17 |
| HIGD1A | 9523.804 | 2131.892 | -2.1594 | 1.51E-30 | 9.95E-29 |
| CLEC4F | 25.68627 | 5.751159 | -2.15907 | 2.08E-24 | 2.75E-23 |
| CETN4P | 6.039216 | 1.352396 | -2.15884 | 1.92E-25 | 3.05E-24 |
| SNCA | 275.3137 | 61.66306 | -2.1586 | 1.08E-22 | 1.08E-21 |
| TBX10 | 593.3529 | 132.915 | -2.15839 | 2.24E-18 | 1.33E-17 |
| RP11-616M22.5 | 5.333333 | 1.194745 | -2.15833 | 2.65E-13 | 9.60E-13 |
| LRRC66 | 1143.627 | 256.2195 | -2.15817 | 3.80E-25 | 5.71E-24 |
| CH17-360D5.1 | 22.80392 | 5.111283 | -2.15752 | 9.48E-20 | 6.54E-19 |
| TRGC1 | 47 | 10.53478 | -2.1575 | 7.47E-21 | 5.90E-20 |
| CACNA1H | 3411.176 | 764.6352 | -2.15743 | 1.58E-17 | 8.60E-17 |
| ITGA7 | 2470.078 | 553.9026 | -2.15685 | 1.71E-19 | 1.14E-18 |
| CTD-2308L22.1 | 21.39216 | 4.797527 | -2.15672 | 8.86E-26 | 1.49E-24 |
| IGHV3-42 | 23.2549 | 5.217929 | -2.15599 | 5.04E-22 | 4.62E-21 |
| TNFRSF13C | 351.4118 | 78.87017 | -2.15561 | 4.11E-17 | 2.14E-16 |
| AC004791.2 | 5.921569 | 1.329212 | -2.15541 | 4.77E-14 | 1.85E-13 |
| CACNA2D1 | 820.902 | 184.2968 | -2.15518 | 3.80E-18 | 2.20E-17 |
| DUX4L27 | 3.941176 | 0.885626 | -2.15386 | 8.27E-23 | 8.40E-22 |
| XCL2 | 31.7451 | 7.134467 | -2.15366 | 4.46E-21 | 3.63E-20 |
| DIRAS1 | 148.5882 | 33.4034 | -2.15325 | 7.55E-28 | 2.03E-26 |
| ARMCX1 | 680.6863 | 153.0309 | -2.15317 | 9.03E-25 | 1.27E-23 |
| IGKV2-4 | 13.66667 | 3.075734 | -2.15166 | 1.24E-20 | 9.53E-20 |
| RP11-483I13.6 | 21.62745 | 4.868624 | -2.15128 | 2.17E-28 | 6.57E-27 |
| MEIS3P1 | 227.3529 | 51.18083 | -2.15126 | 4.72E-28 | 1.32E-26 |
| EMP1 | 19859.61 | 4472.107 | -2.15081 | 7.63E-23 | 7.83E-22 |
| IGLC3 | 11931.16 | 2687.635 | -2.15032 | 8.59E-22 | 7.61E-21 |
| SPINK5 | 918.5686 | 207.0355 | -2.14951 | 3.50E-17 | 1.84E-16 |
| AC053503.6 | 39.17647 | 8.833076 | -2.149 | 1.02E-16 | 5.09E-16 |
| RP11-227H15.4 | 56.94118 | 12.84235 | -2.14856 | 4.69E-20 | 3.36E-19 |
| RP11-367G18.1 | 26.05882 | 5.879444 | -2.14802 | 8.78E-17 | 4.43E-16 |
| KANK2 | 9617.549 | 2169.935 | -2.14802 | 7.82E-23 | 7.99E-22 |
| IGKV6D-41 | 13.86275 | 3.128284 | -2.14777 | 7.76E-21 | 6.11E-20 |
| RP11-148O21.2 | 12.58824 | 2.840804 | -2.1477 | 5.35E-20 | 3.81E-19 |
| STAC | 108.7059 | 24.53787 | -2.14735 | 6.83E-12 | 2.17E-11 |
| APPL2 | 9448.059 | 2133.57 | -2.14675 | 7.91E-29 | 2.71E-27 |
| CLEC4GP1 | 5.117647 | 1.156105 | -2.14621 | 2.13E-21 | 1.79E-20 |
| ZDHHC15 | 78.70588 | 17.78825 | -2.14555 | 1.38E-24 | 1.89E-23 |
| MPPED2 | 86.64706 | 19.58578 | -2.14534 | 4.34E-20 | 3.12E-19 |
| SH2D1B | 58.92157 | 13.32303 | -2.14487 | 2.32E-25 | 3.64E-24 |
| HIGD1AP1 | 8.27451 | 1.871716 | -2.14431 | 1.50E-21 | 1.29E-20 |
| FNBP1 | 5701.588 | 1289.944 | -2.14406 | 3.13E-28 | 9.16E-27 |
| PYY2 | 25.90196 | 5.860896 | -2.14387 | 2.77E-21 | 2.31E-20 |
| AF127936.5 | 15.54902 | 3.522411 | -2.14219 | 3.21E-24 | 4.10E-23 |
| RP11-33I11.3 | 4.705882 | 1.066461 | -2.14163 | 2.06E-20 | 1.54E-19 |
| SIGLEC17P | 53.52941 | 12.13138 | -2.14159 | 3.80E-26 | 6.93E-25 |
| RP11-108M9.3 | 24.56863 | 5.568779 | -2.14138 | 5.68E-18 | 3.23E-17 |
| DAAM2 | 1394.51 | 316.1901 | -2.14089 | 1.85E-27 | 4.42E-26 |
| WFIKKN2 | 20.2549 | 4.595054 | -2.14012 | 1.61E-19 | 1.09E-18 |
| SLC39A12 | 3.078431 | 0.698609 | -2.13964 | 1.70E-22 | 1.66E-21 |
| AMN | 7287.431 | 1653.869 | -2.13957 | 4.80E-22 | 4.42E-21 |
| PDE3A | 4149 | 941.8145 | -2.13925 | 1.46E-27 | 3.58E-26 |
| CAPN13 | 1426.137 | 323.8547 | -2.13869 | 1.91E-22 | 1.86E-21 |
| CMBL | 11706.76 | 2658.785 | -2.1385 | 5.27E-29 | 1.90E-27 |
| MIR22HG | 1742.941 | 395.9196 | -2.13824 | 1.32E-29 | 5.75E-28 |
| APOBR | 8042.627 | 1827.456 | -2.13783 | 5.93E-23 | 6.20E-22 |
| RP11-1399P15.1 | 24.45098 | 5.556414 | -2.13767 | 2.66E-21 | 2.21E-20 |
| MEGF10 | 58.2549 | 13.24111 | -2.13736 | 3.83E-24 | 4.85E-23 |
| MEIS2 | 864.4902 | 196.507 | -2.13727 | 6.33E-18 | 3.58E-17 |
| ELL2P1 | 10.94118 | 2.488408 | -2.13647 | 8.34E-26 | 1.41E-24 |
| FAM218A | 22.68627 | 5.162287 | -2.13574 | 1.07E-25 | 1.76E-24 |
| UGDH | 11763.57 | 2676.978 | -2.13565 | 4.34E-29 | 1.60E-27 |
| FOXI2 | 10.66667 | 2.429675 | -2.13427 | 4.61E-22 | 4.26E-21 |
| TCN2 | 3427.706 | 781.1577 | -2.13356 | 8.85E-29 | 3.01E-27 |
| FLJ22763 | 519.6863 | 118.4668 | -2.13316 | 6.45E-22 | 5.83E-21 |
| SLAMF7 | 2304.137 | 525.4235 | -2.13267 | 1.96E-23 | 2.20E-22 |
| CCDC178 | 16.37255 | 3.734158 | -2.13242 | 2.91E-24 | 3.75E-23 |
| IGHV4-55 | 311.2745 | 71.01855 | -2.13192 | 1.90E-19 | 1.26E-18 |
| ART1 | 3.823529 | 0.873261 | -2.13042 | 4.74E-07 | 9.60E-07 |
| CFP | 294.9216 | 67.36631 | -2.13023 | 9.29E-28 | 2.45E-26 |
| IL16 | 1328.647 | 304 | -2.12781 | 3.06E-27 | 6.93E-26 |
| MXD1 | 10255.96 | 2347.236 | -2.12743 | 4.71E-25 | 6.96E-24 |
| RP11-563M4.1 | 2.039216 | 0.46677 | -2.12723 | 2.35E-27 | 5.44E-26 |
| LINC01489 | 2.686275 | 0.615147 | -2.1266 | 3.70E-10 | 1.00E-09 |
| RP11-1070N10.3 | 20.41176 | 4.675425 | -2.12623 | 4.81E-21 | 3.89E-20 |
| CITED2 | 4172.392 | 955.8083 | -2.12608 | 1.01E-31 | 1.15E-29 |
| RP11-710C12.1 | 25.78431 | 5.907264 | -2.12593 | 2.01E-28 | 6.16E-27 |
| RHBDL2 | 1493.373 | 342.2906 | -2.12528 | 6.17E-25 | 8.93E-24 |
| PALM | 1184.451 | 271.7929 | -2.12364 | 4.68E-25 | 6.94E-24 |
| CCDC13 | 55.47059 | 12.72952 | -2.12354 | 7.33E-28 | 1.97E-26 |
| ITGA8 | 754.1765 | 173.1314 | -2.12303 | 9.36E-29 | 3.14E-27 |
| RP11-713P17.3 | 35.13725 | 8.066461 | -2.12299 | 7.20E-19 | 4.55E-18 |
| SEMA3G | 1027.588 | 235.9413 | -2.12276 | 3.78E-25 | 5.69E-24 |
| GAPT | 138.4118 | 31.79598 | -2.12205 | 1.33E-22 | 1.32E-21 |
| CD1D | 386.5098 | 88.80526 | -2.12179 | 2.27E-28 | 6.83E-27 |
| NCF1B | 99.33333 | 22.83771 | -2.12086 | 2.07E-24 | 2.74E-23 |
| IGKV1D-8 | 244.6667 | 56.26121 | -2.12061 | 1.39E-22 | 1.37E-21 |
| LTK | 954.2549 | 219.4513 | -2.12047 | 2.93E-22 | 2.78E-21 |
| TMEM132D | 27.4902 | 6.323029 | -2.12023 | 9.31E-24 | 1.10E-22 |
| IGHV3-33 | 1907.294 | 438.847 | -2.11974 | 6.02E-22 | 5.47E-21 |
| ETHE1 | 19861.31 | 4571.17 | -2.11933 | 4.34E-29 | 1.60E-27 |
| UBE2FP1 | 85.03922 | 19.57805 | -2.11889 | 7.82E-27 | 1.64E-25 |
| CAMK2A | 59.27451 | 13.6476 | -2.11876 | 1.00E-23 | 1.18E-22 |
| TRIM50 | 9.666667 | 2.227202 | -2.11779 | 6.59E-23 | 6.84E-22 |
| SLC25A15P3 | 3.313725 | 0.763524 | -2.11771 | 1.31E-12 | 4.45E-12 |
| RP11-146D12.2 | 9.764706 | 2.250386 | -2.1174 | 4.26E-22 | 3.96E-21 |
| RP11-541E12.1 | 4.392157 | 1.012365 | -2.1172 | 2.86E-07 | 5.91E-07 |
| SCN3B | 121.4902 | 28.01546 | -2.11655 | 2.08E-26 | 3.95E-25 |
| RP11-368J22.2 | 1.862745 | 0.429675 | -2.11611 | 7.50E-14 | 2.85E-13 |
| OR5K2 | 2.686275 | 0.619784 | -2.11577 | 9.07E-15 | 3.76E-14 |
| C2orf74 | 47.35294 | 10.9289 | -2.11531 | 2.22E-24 | 2.92E-23 |
| ADRA2A | 2131.549 | 492.085 | -2.11492 | 2.51E-20 | 1.86E-19 |
| B4GALT1-AS1 | 149.3137 | 34.47913 | -2.11455 | 2.28E-29 | 9.08E-28 |
| LINC01266 | 9.509804 | 2.196291 | -2.11435 | 1.45E-17 | 7.91E-17 |
| LRRC55 | 72.37255 | 16.7187 | -2.11398 | 1.88E-24 | 2.51E-23 |
| AC244250.4 | 29.03922 | 6.718702 | -2.11175 | 2.90E-21 | 2.41E-20 |
| IGHD3-9 | 4.882353 | 1.12983 | -2.11147 | 3.29E-20 | 2.40E-19 |
| CTC-378H22.2 | 20.88235 | 4.834621 | -2.11081 | 2.33E-23 | 2.59E-22 |
| DDR2 | 4334.137 | 1003.827 | -2.11023 | 3.05E-21 | 2.53E-20 |
| ATP2A3 | 19425.45 | 4500.298 | -2.10986 | 3.61E-26 | 6.60E-25 |
| C14orf39 | 4.294118 | 0.995363 | -2.10907 | 6.94E-07 | 1.38E-06 |
| SHOX | 18.7451 | 4.346213 | -2.10868 | 1.38E-22 | 1.36E-21 |
| CTD-2006M22.2 | 41.80392 | 9.693972 | -2.10848 | 1.41E-22 | 1.39E-21 |
| KLF9 | 4034.667 | 935.711 | -2.10831 | 1.88E-27 | 4.50E-26 |
| RP11-960L18.1 | 16.21569 | 3.766615 | -2.10605 | 3.35E-21 | 2.76E-20 |
| MEIS1 | 1329.647 | 308.9196 | -2.10574 | 2.37E-22 | 2.27E-21 |
| IGLV3-15 | 1.882353 | 0.437403 | -2.1055 | 6.49E-14 | 2.48E-13 |
| SLC23A3 | 288.3137 | 67.00464 | -2.10531 | 1.30E-11 | 4.04E-11 |
| SERTAD4-AS1 | 160.902 | 37.39722 | -2.10518 | 2.15E-18 | 1.29E-17 |
| FGF13-AS1 | 5.72549 | 1.330757 | -2.10515 | 2.82E-18 | 1.66E-17 |
| AFF2 | 42.01961 | 9.768161 | -2.1049 | 8.66E-18 | 4.84E-17 |
| PTGDR | 584.8824 | 135.9907 | -2.10464 | 2.59E-25 | 4.04E-24 |
| RP11-834C11.7 | 15.80392 | 3.675425 | -2.1043 | 3.26E-24 | 4.17E-23 |
| SVILP1 | 82.19608 | 19.12674 | -2.10348 | 2.70E-19 | 1.77E-18 |
| CYP11A1 | 29.68627 | 6.911901 | -2.10264 | 6.94E-27 | 1.47E-25 |
| RP11-284H19.1 | 1.745098 | 0.406491 | -2.10201 | 2.67E-28 | 7.92E-27 |
| LYPLAL1-AS1 | 5.705882 | 1.329212 | -2.10188 | 2.25E-13 | 8.21E-13 |
| SMIM14 | 10794.69 | 2515.355 | -2.10149 | 3.34E-30 | 1.84E-28 |
| GYPC | 1310.059 | 305.4498 | -2.10062 | 2.05E-27 | 4.83E-26 |
| PRKAA2 | 495.2549 | 115.4961 | -2.10033 | 6.19E-22 | 5.61E-21 |
| RPL7AP28 | 8.509804 | 1.984544 | -2.10032 | 2.42E-20 | 1.80E-19 |
| TEF | 2800.608 | 653.238 | -2.10006 | 1.83E-29 | 7.57E-28 |
| ANXA8L1 | 44.41176 | 10.36321 | -2.09947 | 6.73E-22 | 6.06E-21 |
| TPSAB1 | 1010.824 | 235.8903 | -2.09934 | 1.79E-25 | 2.86E-24 |
| TUB | 323.2549 | 75.44204 | -2.09923 | 8.85E-24 | 1.05E-22 |
| LINC01022 | 2 | 0.46677 | -2.09922 | 1.45E-16 | 7.17E-16 |
| LINC01135 | 18.84314 | 4.398764 | -2.09887 | 6.70E-26 | 1.16E-24 |
| RP11-293M10.6 | 103.1961 | 24.09119 | -2.09881 | 3.21E-30 | 1.78E-28 |
| CDKL1 | 283.7843 | 66.2813 | -2.09812 | 6.60E-30 | 3.18E-28 |
| NPPC | 19.82353 | 4.633694 | -2.09698 | 5.43E-16 | 2.53E-15 |
| RP1-193H18.3 | 4.901961 | 1.146832 | -2.09571 | 1.87E-28 | 5.78E-27 |
| RP11-12M5.3 | 10.15686 | 2.378671 | -2.09423 | 2.86E-20 | 2.11E-19 |
| PPARGC1A | 2097.667 | 491.5518 | -2.09337 | 4.56E-27 | 1.00E-25 |
| TPO | 101.4118 | 23.79134 | -2.09172 | 8.51E-23 | 8.62E-22 |
| PTX3 | 118.7843 | 27.87944 | -2.09107 | 9.77E-26 | 1.63E-24 |
| CD1C | 185.6667 | 43.59196 | -2.09058 | 2.12E-23 | 2.37E-22 |
| AC009014.3 | 627.1569 | 147.2813 | -2.09025 | 2.87E-24 | 3.70E-23 |
| CYSLTR1 | 239.8627 | 56.34312 | -2.0899 | 4.53E-28 | 1.28E-26 |
| RP11-514D23.2 | 9.823529 | 2.307573 | -2.08986 | 1.73E-26 | 3.36E-25 |
| ACACB | 3188.333 | 749.0139 | -2.08974 | 2.11E-29 | 8.50E-28 |
| PEX26 | 10709.59 | 2516.247 | -2.08956 | 1.65E-27 | 4.02E-26 |
| PIK3CG | 515 | 121.0448 | -2.08903 | 4.97E-25 | 7.33E-24 |
| C20orf194 | 893.8824 | 210.1546 | -2.08863 | 7.70E-23 | 7.89E-22 |
| RP11-182J1.1 | 6.529412 | 1.536321 | -2.08747 | 1.04E-22 | 1.05E-21 |
| NKX3-2 | 209.2745 | 49.25039 | -2.08719 | 6.39E-14 | 2.44E-13 |
| MATN2 | 6486.235 | 1526.912 | -2.08676 | 6.59E-28 | 1.79E-26 |
| RP11-1060J15.4 | 147.4706 | 34.73879 | -2.08581 | 1.20E-22 | 1.19E-21 |
| SLC22A17 | 811.6078 | 191.1947 | -2.08574 | 3.41E-25 | 5.17E-24 |
| HIST2H2BA | 3.54902 | 0.836167 | -2.08556 | 4.40E-16 | 2.07E-15 |
| DCN | 31244.82 | 7365.937 | -2.08468 | 5.83E-25 | 8.48E-24 |
| RP11-524H19.2 | 34.70588 | 8.183926 | -2.08432 | 3.16E-23 | 3.44E-22 |
| SEC1P | 32.19608 | 7.593509 | -2.08405 | 4.92E-24 | 6.13E-23 |
| RP11-109E24.2 | 3.176471 | 0.749614 | -2.08321 | 4.76E-19 | 3.05E-18 |
| NDN | 1435.157 | 338.9629 | -2.08201 | 4.94E-28 | 1.37E-26 |
| IGLV3-19 | 5709.804 | 1348.753 | -2.08182 | 8.05E-23 | 8.20E-22 |
| SYNC | 321.5686 | 75.99536 | -2.08114 | 3.48E-26 | 6.39E-25 |
| RPSAP75 | 4.803922 | 1.136012 | -2.08023 | 6.30E-20 | 4.44E-19 |
| AMOTL1 | 2740.157 | 648.3354 | -2.07945 | 2.18E-18 | 1.30E-17 |
| GNA11 | 19750.31 | 4673.332 | -2.07935 | 2.01E-28 | 6.16E-27 |
| PIGZ | 4880.373 | 1155.117 | -2.07895 | 1.62E-24 | 2.19E-23 |
| ARHGEF26 | 848.0392 | 200.8903 | -2.07772 | 9.58E-18 | 5.32E-17 |
| CHRNA1 | 169.1569 | 40.07728 | -2.07751 | 1.58E-22 | 1.55E-21 |
| RP11-102N12.3 | 34.56863 | 8.191654 | -2.07724 | 2.29E-25 | 3.60E-24 |
| SLC25A23 | 11014.1 | 2610.889 | -2.07674 | 9.32E-31 | 6.86E-29 |
| HS6ST3 | 27.52941 | 6.527048 | -2.07647 | 7.34E-23 | 7.56E-22 |
| RP5-1125N11.2 | 20.52941 | 4.874807 | -2.07428 | 2.62E-10 | 7.21E-10 |
| TLR7 | 321.6078 | 76.36785 | -2.07427 | 1.35E-24 | 1.85E-23 |
| RP11-756A22.7 | 25.05882 | 5.950541 | -2.07423 | 1.08E-16 | 5.40E-16 |
| OLFM1 | 791.9608 | 188.0881 | -2.07402 | 1.54E-26 | 3.02E-25 |
| IGHV3-25 | 26.86275 | 6.380216 | -2.07393 | 3.72E-20 | 2.70E-19 |
| IGHV4-34 | 1880.863 | 446.7898 | -2.07373 | 8.83E-21 | 6.91E-20 |
| HPCAL4 | 20.05882 | 4.76507 | -2.07367 | 5.28E-20 | 3.76E-19 |
| JPH3 | 57.29412 | 13.6136 | -2.07334 | 3.90E-26 | 7.09E-25 |
| RP11-325F22.2 | 55.52941 | 13.19629 | -2.07312 | 1.00E-24 | 1.39E-23 |
| LINC01133 | 1977.137 | 470.0479 | -2.07253 | 2.82E-25 | 4.35E-24 |
| KCNK2 | 49.92157 | 11.86862 | -2.07251 | 2.63E-25 | 4.08E-24 |
| NR4A3 | 847.7647 | 201.575 | -2.07235 | 0.000201 | 0.000311 |
| CBX7 | 2979.353 | 708.6708 | -2.07181 | 3.42E-28 | 9.93E-27 |
| PWRN1 | 5.431373 | 1.292117 | -2.07158 | 1.95E-24 | 2.60E-23 |
| RP11-375H19.2 | 11.09804 | 2.642968 | -2.07007 | 9.08E-18 | 5.06E-17 |
| CTD-3157E16.1 | 138.7843 | 33.06801 | -2.06934 | 1.07E-27 | 2.75E-26 |
| ERBB4 | 11.86275 | 2.826893 | -2.06915 | 1.46E-24 | 1.98E-23 |
| SGCE | 1026.98 | 244.813 | -2.06866 | 2.96E-24 | 3.81E-23 |
| ATP8A2 | 97.54902 | 23.25811 | -2.06839 | 4.44E-24 | 5.57E-23 |
| AC005682.6 | 31.58824 | 7.53323 | -2.06805 | 9.05E-24 | 1.08E-22 |
| MTND6P3 | 8.098039 | 1.931994 | -2.06748 | 1.72E-10 | 4.80E-10 |
| STYK1 | 1564.275 | 373.2009 | -2.06747 | 3.06E-27 | 6.93E-26 |
| RP11-89C3.3 | 2.039216 | 0.486862 | -2.06643 | 1.34E-16 | 6.65E-16 |
| PAK3 | 71.60784 | 17.10201 | -2.06595 | 5.53E-24 | 6.84E-23 |
| ROR1 | 461.6275 | 110.4513 | -2.06332 | 1.28E-22 | 1.27E-21 |
| 12-Sep | 4.941176 | 1.18238 | -2.06316 | 5.37E-18 | 3.06E-17 |
| CNTNAP3B | 43.72549 | 10.47141 | -2.06202 | 4.69E-19 | 3.01E-18 |
| FAXC | 279.8627 | 67.03246 | -2.06179 | 4.12E-13 | 1.47E-12 |
| ABCB1 | 6594.314 | 1579.635 | -2.06163 | 6.50E-19 | 4.12E-18 |
| IGHV2-26 | 672.1961 | 161.119 | -2.06076 | 3.28E-20 | 2.40E-19 |
| RP11-160O5.1 | 178.9608 | 42.90263 | -2.06051 | 7.51E-29 | 2.60E-27 |
| DUSP5P2 | 2.372549 | 0.568779 | -2.0605 | 2.00E-17 | 1.08E-16 |
| AKR1B15 | 48.11765 | 11.53787 | -2.06019 | 4.53E-10 | 1.22E-09 |
| EPB41L4A-AS2 | 46.23529 | 11.09119 | -2.05958 | 1.49E-26 | 2.92E-25 |
| PTPRR | 1726.588 | 414.1994 | -2.05953 | 1.21E-20 | 9.29E-20 |
| RP11-157L3.9 | 2.137255 | 0.513138 | -2.05834 | 5.75E-14 | 2.21E-13 |
| RP3-413H6.2 | 2.098039 | 0.503864 | -2.05794 | 7.87E-14 | 2.99E-13 |
| WDR64 | 6.27451 | 1.506955 | -2.05787 | 5.81E-23 | 6.08E-22 |
| TUNAR | 5.039216 | 1.211747 | -2.05611 | 1.37E-20 | 1.05E-19 |
| RPS29P12 | 3.411765 | 0.820711 | -2.05557 | 6.97E-21 | 5.52E-20 |
| SYT6 | 14.39216 | 3.462133 | -2.05555 | 3.23E-18 | 1.89E-17 |
| MTRNR2L1 | 1536.039 | 369.51 | -2.05553 | 1.20E-06 | 2.33E-06 |
| CKB | 81075.94 | 19504.08 | -2.0555 | 2.32E-22 | 2.23E-21 |
| C4orf19 | 2568.98 | 618.1592 | -2.05515 | 4.91E-28 | 1.37E-26 |
| RP11-350N15.3 | 2.54902 | 0.613601 | -2.05457 | 2.64E-10 | 7.25E-10 |
| TTC22 | 4592.529 | 1105.683 | -2.05435 | 2.38E-26 | 4.49E-25 |
| RP11-446H18.1 | 5.72549 | 1.378671 | -2.05412 | 1.72E-18 | 1.04E-17 |
| MCOLN2 | 1575.353 | 379.4127 | -2.05384 | 1.74E-17 | 9.44E-17 |
| SLC10A4 | 41.43137 | 9.998454 | -2.05095 | 1.41E-25 | 2.29E-24 |
| ZZEF1 | 10452.33 | 2523.42 | -2.05037 | 5.08E-26 | 8.99E-25 |
| PPP1R16B | 1210.118 | 292.1561 | -2.05034 | 2.01E-26 | 3.84E-25 |
| ACAA2 | 16950.31 | 4093.836 | -2.04979 | 7.67E-28 | 2.06E-26 |
| NPR1 | 645.7451 | 156.0649 | -2.04882 | 1.38E-24 | 1.89E-23 |
| RGS22 | 25.80392 | 6.236476 | -2.04879 | 1.88E-24 | 2.50E-23 |
| CNTNAP3P2 | 10.86275 | 2.627512 | -2.04762 | 4.67E-20 | 3.35E-19 |
| PAMR1 | 768.8039 | 186.0015 | -2.0473 | 1.06E-27 | 2.74E-26 |
| MICU3 | 113.8039 | 27.53787 | -2.04706 | 2.09E-22 | 2.02E-21 |
| C1QTNF2 | 180.1765 | 43.6306 | -2.046 | 2.65E-28 | 7.88E-27 |
| RP11-344P13.6 | 3.254902 | 0.788253 | -2.04588 | 4.96E-21 | 4.01E-20 |
| IGKV2-26 | 19.43137 | 4.707883 | -2.04524 | 4.17E-19 | 2.69E-18 |
| ZNF92P3 | 1.862745 | 0.451314 | -2.04523 | 3.22E-22 | 3.03E-21 |
| ENDOD1 | 7782.745 | 1885.654 | -2.04521 | 7.15E-30 | 3.38E-28 |
| IGLV3-24 | 4.215686 | 1.021638 | -2.04488 | 2.67E-21 | 2.23E-20 |
| RP11-205A8.3 | 6.529412 | 1.582689 | -2.04457 | 1.67E-13 | 6.14E-13 |
| NAT2 | 795.4314 | 192.8253 | -2.04444 | 6.17E-26 | 1.08E-24 |
| LINC01215 | 52.13725 | 12.64451 | -2.0438 | 5.31E-18 | 3.03E-17 |
| NR3C1 | 2367.059 | 574.2457 | -2.04336 | 2.07E-27 | 4.88E-26 |
| RP11-290F5.1 | 185.1176 | 44.94127 | -2.04233 | 1.82E-22 | 1.78E-21 |
| IGHV7-34-1 | 7.823529 | 1.901082 | -2.041 | 9.41E-15 | 3.89E-14 |
| BTLA | 98.33333 | 23.91654 | -2.03967 | 9.35E-25 | 1.31E-23 |
| IGHV1-67 | 85.68627 | 20.85162 | -2.0389 | 9.86E-20 | 6.79E-19 |
| GPR37L1 | 336.2157 | 81.85935 | -2.03817 | 2.18E-24 | 2.87E-23 |
| CORO6 | 104.9804 | 25.58578 | -2.03671 | 2.88E-14 | 1.14E-13 |
| IGHV3-30 | 2800.843 | 682.7125 | -2.03651 | 2.24E-21 | 1.89E-20 |
| CTD-2256P15.5 | 8.666667 | 2.112828 | -2.0363 | 1.62E-16 | 7.96E-16 |
| KRT8P24 | 38.64706 | 9.434312 | -2.03437 | 5.59E-09 | 1.36E-08 |
| SIGLEC1 | 1219.98 | 298.0556 | -2.0332 | 1.61E-23 | 1.83E-22 |
| TRDC | 156.7647 | 38.30603 | -2.03296 | 1.06E-22 | 1.06E-21 |
| RP11-336A10.4 | 1.921569 | 0.469861 | -2.03198 | 2.95E-11 | 8.86E-11 |
| MAP1LC3C | 13.94118 | 3.409583 | -2.03169 | 5.36E-19 | 3.42E-18 |
| RP11-730A19.9 | 22.31373 | 5.459042 | -2.03121 | 5.12E-27 | 1.11E-25 |
| RP11-685G9.2 | 5.843137 | 1.429675 | -2.03106 | 1.83E-12 | 6.14E-12 |
| SHE | 522.4902 | 127.9428 | -2.0299 | 2.02E-27 | 4.79E-26 |
| RP11-90P13.1 | 10.29412 | 2.520866 | -2.02983 | 5.07E-19 | 3.24E-18 |
| C10orf54 | 5523.882 | 1353.436 | -2.02906 | 3.64E-30 | 1.96E-28 |
| AC083900.1 | 27.07843 | 6.63524 | -2.02892 | 1.10E-21 | 9.62E-21 |
| SEC14L6 | 25.52941 | 6.258114 | -2.02836 | 1.66E-23 | 1.88E-22 |
| CTD-2306A12.1 | 17.78431 | 4.36476 | -2.02663 | 4.65E-21 | 3.77E-20 |
| LL22NC03-75H12.2 | 10.33333 | 2.537867 | -2.02562 | 3.24E-20 | 2.36E-19 |
| TEPP | 5.039216 | 1.238022 | -2.02516 | 5.73E-19 | 3.65E-18 |
| A2M-AS1 | 70.35294 | 17.28748 | -2.02488 | 7.83E-22 | 6.99E-21 |
| GRID2 | 5.156863 | 1.267388 | -2.02464 | 5.40E-25 | 7.90E-24 |
| RN7SL417P | 2.490196 | 0.612056 | -2.02452 | 1.09E-19 | 7.44E-19 |
| ZNF582-AS1 | 47.58824 | 11.69706 | -2.02446 | 4.87E-26 | 8.63E-25 |
| DDX25 | 8.764706 | 2.15456 | -2.02431 | 7.50E-24 | 9.07E-23 |
| LINC00877 | 28.68627 | 7.05255 | -2.02414 | 4.29E-23 | 4.57E-22 |
| TBC1D27 | 42.31373 | 10.4034 | -2.02407 | 1.53E-18 | 9.31E-18 |
| TDRD10 | 40.45098 | 9.959815 | -2.02198 | 7.73E-28 | 2.07E-26 |
| CLC | 235.4314 | 57.98145 | -2.02164 | 1.91E-09 | 4.85E-09 |
| SYPL2 | 86.19608 | 21.23029 | -2.0215 | 1.78E-19 | 1.19E-18 |
| IGLV3-13 | 10.41176 | 2.565688 | -2.0208 | 2.32E-18 | 1.38E-17 |
| KCNB2 | 12.09804 | 2.981453 | -2.02069 | 1.74E-26 | 3.36E-25 |
| URAD | 829.098 | 204.3756 | -2.02032 | 4.40E-15 | 1.88E-14 |
| PAG1 | 4432.118 | 1092.771 | -2.02 | 8.08E-25 | 1.15E-23 |
| P2RY8 | 631.0392 | 155.6028 | -2.01986 | 1.24E-23 | 1.43E-22 |
| LRRTM3 | 3.509804 | 0.865533 | -2.01973 | 2.74E-25 | 4.23E-24 |
| PLA2G10 | 435.0588 | 107.3849 | -2.01842 | 1.27E-25 | 2.07E-24 |
| ACVRL1 | 9009.647 | 2224.598 | -2.01793 | 1.02E-27 | 2.63E-26 |
| LMO1 | 4.431373 | 1.094281 | -2.01777 | 2.99E-18 | 1.76E-17 |
| AC073316.1 | 6.137255 | 1.516229 | -2.01711 | 4.77E-20 | 3.41E-19 |
| SLIT2 | 952.5294 | 235.3338 | -2.01705 | 2.34E-21 | 1.96E-20 |
| GPRASP1 | 618.3725 | 152.9985 | -2.01496 | 8.10E-21 | 6.36E-20 |
| RTN1 | 424.7255 | 105.136 | -2.01427 | 1.50E-25 | 2.42E-24 |
| RP11-230B22.1 | 21.13725 | 5.233385 | -2.01397 | 2.49E-26 | 4.68E-25 |
| FAM13C | 216.902 | 53.71097 | -2.01375 | 7.32E-24 | 8.86E-23 |
| CNNM2 | 2429.922 | 601.8331 | -2.01347 | 1.21E-27 | 3.06E-26 |
| RP11-359E10.1 | 22.60784 | 5.599691 | -2.0134 | 3.83E-13 | 1.36E-12 |
| RP11-281P23.2 | 15.27451 | 3.785162 | -2.0127 | 8.32E-19 | 5.22E-18 |
| RP4-663N10.1 | 2.588235 | 0.641422 | -2.01262 | 3.24E-19 | 2.10E-18 |
| CASP7 | 9901.471 | 2454.553 | -2.01218 | 3.95E-28 | 1.13E-26 |
| KIT | 1307.157 | 324.7913 | -2.00885 | 3.03E-25 | 4.64E-24 |
| HORMAD2-AS1 | 5.392157 | 1.340031 | -2.0086 | 1.40E-24 | 1.91E-23 |
| IGSF9 | 3477.196 | 864.2457 | -2.00841 | 1.70E-21 | 1.44E-20 |
| MYH15 | 214.0588 | 53.20711 | -2.00832 | 2.24E-24 | 2.94E-23 |
| TIFAB | 88.52941 | 22.00773 | -2.00815 | 5.90E-22 | 5.36E-21 |
| RP11-875O11.1 | 44.7451 | 11.12365 | -2.0081 | 4.20E-25 | 6.27E-24 |
| FLT3 | 81.37255 | 20.23184 | -2.00791 | 1.77E-26 | 3.41E-25 |
| HRASLS5 | 111.0196 | 27.60896 | -2.00761 | 2.29E-21 | 1.92E-20 |
| CHST9 | 37.13725 | 9.238022 | -2.00721 | 4.36E-34 | 1.12E-31 |
| TMEM132B | 87.09804 | 21.66924 | -2.00699 | 1.02E-21 | 8.91E-21 |
| FMO4 | 514.3333 | 127.9722 | -2.00687 | 6.27E-28 | 1.71E-26 |
| RP11-425D10.10 | 44.07843 | 10.96754 | -2.00683 | 2.89E-22 | 2.74E-21 |
| IGLC2 | 16973.88 | 4226.331 | -2.00584 | 8.25E-23 | 8.38E-22 |
| SLC14A2-AS1 | 1.843137 | 0.459042 | -2.00547 | 3.06E-12 | 1.01E-11 |
| SPATA31C2 | 2.54902 | 0.63524 | -2.00457 | 6.95E-18 | 3.91E-17 |
| BEX4 | 1268.098 | 316.0587 | -2.0044 | 1.03E-25 | 1.71E-24 |
| KAT2B | 1765.804 | 440.3864 | -2.00348 | 2.03E-30 | 1.25E-28 |
| RELL1 | 537 | 133.9536 | -2.00319 | 2.63E-27 | 6.05E-26 |
| FERMT2 | 3474.922 | 866.8192 | -2.00318 | 8.68E-17 | 4.38E-16 |
| TSPAN2 | 1504.647 | 375.3864 | -2.00298 | 1.13E-15 | 5.10E-15 |
| RP11-7F17.3 | 9.588235 | 2.392581 | -2.0027 | 2.12E-21 | 1.79E-20 |
| ANK3 | 3511.667 | 876.4389 | -2.00243 | 1.11E-26 | 2.25E-25 |
| LINC01105 | 4 | 0.998454 | -2.00223 | 2.75E-22 | 2.62E-21 |
| RP11-172C16.5 | 2.941176 | 0.734158 | -2.00223 | 3.07E-20 | 2.26E-19 |
| CUX2 | 22.70588 | 5.667697 | -2.00223 | 2.15E-29 | 8.65E-28 |
| DOCK3 | 135.6275 | 33.86553 | -2.00176 | 1.48E-13 | 5.49E-13 |
| FAXDC2 | 2274.608 | 568.1607 | -2.00125 | 4.17E-23 | 4.46E-22 |
| CDK15 | 31.41176 | 7.846986 | -2.00109 | 4.39E-24 | 5.50E-23 |
| IGHV3-20 | 200.4706 | 50.10201 | -2.00045 | 3.05E-20 | 2.24E-19 |
| KCNMB2 | 22.62745 | 5.655332 | -2.00039 | 3.59E-21 | 2.95E-20 |
| DPP10 | 335.7451 | 83.92427 | -2.00021 | 1.08E-22 | 1.09E-21 |
| FOXP4-AS1 | 24.23529 | 96.983 | 2.000622 | 4.76E-19 | 3.05E-18 |
| RP11-1267H10.4 | 13.21569 | 52.93045 | 2.001847 | 0.006891 | 0.008865 |
| LINC01484 | 2.215686 | 8.88408 | 2.003469 | 3.58E-09 | 8.86E-09 |
| SLC17A9 | 554.2745 | 2223.153 | 2.003935 | 1.64E-13 | 6.04E-13 |
| RP11-261P13.5 | 1.058824 | 4.251932 | 2.005656 | 1.68E-09 | 4.29E-09 |
| RBMXP1 | 0.392157 | 1.574961 | 2.005814 | 4.49E-05 | 7.46E-05 |
| CCT6P2 | 1.588235 | 6.378671 | 2.005831 | 1.03E-10 | 2.95E-10 |
| CTD-2349P21.5 | 1.470588 | 5.907264 | 2.006097 | 3.58E-07 | 7.34E-07 |
| WI2-87327B8.2 | 1.647059 | 6.616692 | 2.006218 | 2.77E-06 | 5.20E-06 |
| RP11-168K11.3 | 0.764706 | 3.074189 | 2.007229 | 8.48E-08 | 1.85E-07 |
| KLHL2P1 | 1.54902 | 6.227202 | 2.007229 | 3.30E-10 | 8.98E-10 |
| ANKRD13B | 173.9412 | 699.3972 | 2.007513 | 3.41E-22 | 3.20E-21 |
| LINC01121 | 1.313725 | 5.285935 | 2.008495 | 0.005921 | 0.007684 |
| RP4-568F9.6 | 0.627451 | 2.528594 | 2.01076 | 0.011033 | 0.013834 |
| KIAA1549 | 293.4118 | 1182.606 | 2.010971 | 9.12E-22 | 8.05E-21 |
| PTP4A3 | 655.9216 | 2645.462 | 2.011925 | 7.00E-14 | 2.67E-13 |
| PCAT1 | 2.823529 | 11.40958 | 2.014674 | 1.70E-13 | 6.27E-13 |
| STIP1P3 | 1.392157 | 5.63524 | 2.017155 | 1.89E-08 | 4.39E-08 |
| CTB-193M12.3 | 0.45098 | 1.826893 | 2.018256 | 0.003215 | 0.004319 |
| GCKR | 2.352941 | 9.539413 | 2.019435 | 2.70E-05 | 4.60E-05 |
| C19orf45 | 40.92157 | 165.932 | 2.019659 | 2.61E-15 | 1.14E-14 |
| RP11-85I17.2 | 3.843137 | 15.58578 | 2.019874 | 4.27E-12 | 1.39E-11 |
| RP11-452H21.1 | 0.607843 | 2.468315 | 2.021756 | 1.30E-05 | 2.28E-05 |
| RPSAP52 | 3.039216 | 12.34312 | 2.021936 | 1.93E-13 | 7.06E-13 |
| L29074.3 | 0.45098 | 1.833076 | 2.02313 | 5.63E-05 | 9.25E-05 |
| RP11-5G9.5 | 1.372549 | 5.581144 | 2.023703 | 2.50E-05 | 4.26E-05 |
| CTC-537E7.2 | 2.803922 | 11.42658 | 2.026876 | 0.007627 | 0.009768 |
| SEPW1P | 0.588235 | 2.397218 | 2.026896 | 0.003361 | 0.004505 |
| CTC-329H14.1 | 1.54902 | 6.313756 | 2.027143 | 2.43E-05 | 4.15E-05 |
| AC108463.2 | 2.54902 | 10.40958 | 2.029898 | 2.79E-12 | 9.19E-12 |
| CTB-35F21.1 | 0.588235 | 2.4034 | 2.030612 | 9.04E-05 | 0.000145 |
| RP11-629O1.2 | 5.156863 | 21.0881 | 2.031863 | 4.91E-10 | 1.32E-09 |
| SCD | 5273.078 | 21567.39 | 2.032134 | 1.99E-18 | 1.19E-17 |
| ASPHD1 | 90.92157 | 372.0958 | 2.03298 | 1.26E-14 | 5.15E-14 |
| RP11-93K22.1 | 0.313725 | 1.284389 | 2.033508 | 9.71E-06 | 1.73E-05 |
| SULT1C2P1 | 1.313725 | 5.380216 | 2.034 | 1.72E-05 | 2.98E-05 |
| WBSCR28 | 3.019608 | 12.38794 | 2.036504 | 1.42E-13 | 5.27E-13 |
| MIR3153 | 0.784314 | 3.217929 | 2.03663 | 5.40E-07 | 1.09E-06 |
| MS4A15 | 5.666667 | 23.2643 | 2.037545 | 0.000133 | 0.00021 |
| AC012066.1 | 1 | 4.109737 | 2.039046 | 6.42E-09 | 1.55E-08 |
| TDGF1P5 | 0.647059 | 2.661515 | 2.040279 | 0.011782 | 0.014723 |
| HILPDA | 320.1961 | 1319.034 | 2.042454 | 8.46E-23 | 8.57E-22 |
| COLEC10 | 4.196078 | 17.2983 | 2.043517 | 0.03573 | 0.042101 |
| CTD-2227E11.1 | 22.98039 | 94.76662 | 2.043975 | 9.37E-14 | 3.54E-13 |
| RP13-216E22.5 | 1.117647 | 4.61051 | 2.044462 | 0.000791 | 0.001144 |
| IZUMO1 | 1.862745 | 7.690881 | 2.045719 | 3.67E-08 | 8.30E-08 |
| MFAP2 | 187.2157 | 773.983 | 2.047601 | 9.75E-15 | 4.03E-14 |
| SEPHS1P4 | 0.764706 | 3.162287 | 2.047992 | 1.54E-09 | 3.95E-09 |
| STRIP2 | 70.98039 | 293.592 | 2.04832 | 1.76E-20 | 1.33E-19 |
| RP11-661A12.9 | 5.352941 | 22.14219 | 2.048395 | 3.85E-08 | 8.70E-08 |
| CYP4A22-AS1 | 2.784314 | 11.52241 | 2.049049 | 9.26E-15 | 3.83E-14 |
| RP11-123K3.9 | 0.372549 | 1.542504 | 2.049772 | 8.53E-06 | 1.53E-05 |
| PDCD5P1 | 0.960784 | 3.978362 | 2.04989 | 1.62E-10 | 4.53E-10 |
| RP11-500B12.1 | 4.745098 | 19.65688 | 2.050524 | 0.038594 | 0.045302 |
| RP11-344L21.1 | 0.509804 | 2.112828 | 2.051161 | 0.00495 | 0.006488 |
| MPP4 | 0.921569 | 3.83153 | 2.055757 | 5.66E-11 | 1.65E-10 |
| ATP5G2P4 | 2.72549 | 11.3323 | 2.055853 | 3.74E-05 | 6.26E-05 |
| LINC01124 | 27.98039 | 116.3539 | 2.056032 | 5.08E-12 | 1.63E-11 |
| MACC1 | 749.5098 | 3118.116 | 2.056655 | 2.23E-17 | 1.19E-16 |
| MIR6797 | 0.705882 | 2.939722 | 2.05818 | 7.39E-10 | 1.95E-09 |
| AL022326.1 | 0.862745 | 3.599691 | 2.060867 | 2.82E-08 | 6.44E-08 |
| RP11-307P22.1 | 0.568627 | 2.37558 | 2.062724 | 4.47E-07 | 9.07E-07 |
| RP5-1029F21.2 | 0.54902 | 2.295209 | 2.063696 | 3.76E-06 | 6.97E-06 |
| FAM227A | 23.29412 | 97.4034 | 2.064006 | 1.25E-18 | 7.64E-18 |
| SNRPD2P1 | 0.627451 | 2.62442 | 2.064424 | 5.42E-07 | 1.09E-06 |
| GS1-600G8.5 | 2.137255 | 8.955178 | 2.066963 | 2.64E-05 | 4.50E-05 |
| PGLYRP4 | 1.843137 | 7.72643 | 2.067638 | 0.000118 | 0.000187 |
| GABRE | 341.6667 | 1434.58 | 2.069967 | 6.81E-14 | 2.60E-13 |
| RP11-832N8.1 | 13.15686 | 55.26275 | 2.070492 | 1.59E-11 | 4.88E-11 |
| UBE2SP2 | 2.156863 | 9.060278 | 2.070621 | 1.55E-13 | 5.74E-13 |
| INHBB | 99.37255 | 417.6337 | 2.071319 | 5.90E-05 | 9.68E-05 |
| HIST3H2BB | 7.372549 | 31.00773 | 2.072392 | 2.03E-06 | 3.87E-06 |
| ATP6V1C2 | 86.2549 | 363.0155 | 2.073353 | 1.21E-14 | 4.97E-14 |
| CYP2D7 | 13.01961 | 54.87017 | 2.075336 | 3.79E-17 | 1.98E-16 |
| RP11-510I6.2 | 1.196078 | 5.05255 | 2.0787 | 0.000128 | 0.000202 |
| RN7SKP173 | 1.196078 | 5.054096 | 2.079141 | 6.04E-06 | 1.10E-05 |
| MKRN5P | 1.529412 | 6.474498 | 2.081791 | 0.004044 | 0.005359 |
| SLC11A1 | 89.98039 | 380.9459 | 2.081904 | 2.47E-12 | 8.18E-12 |
| RP11-128M1.1 | 4.647059 | 19.68779 | 2.082911 | 2.42E-10 | 6.66E-10 |
| RP11-334A14.8 | 1.54902 | 6.565688 | 2.083591 | 8.69E-08 | 1.89E-07 |
| FSCN1P1 | 0.45098 | 1.914992 | 2.086202 | 0.000116 | 0.000184 |
| RP11-220D10.1 | 3.627451 | 15.4034 | 2.086221 | 3.80E-08 | 8.59E-08 |
| RPL31P2 | 0.823529 | 3.497682 | 2.086507 | 7.25E-08 | 1.59E-07 |
| ADAMTS2 | 459.9412 | 1954.652 | 2.087391 | 1.30E-11 | 4.04E-11 |
| RP11-47I22.2 | 1.431373 | 6.085008 | 2.08786 | 4.66E-08 | 1.04E-07 |
| GRHL1 | 60.2549 | 256.3014 | 2.088691 | 3.53E-18 | 2.05E-17 |
| WFDC10B | 2.901961 | 12.36785 | 2.091495 | 1.27E-09 | 3.28E-09 |
| TNNT2 | 11.15686 | 47.58578 | 2.092599 | 2.02E-09 | 5.14E-09 |
| SUPT4H1P1 | 0.411765 | 1.757342 | 2.093503 | 3.76E-06 | 6.98E-06 |
| CREG2 | 11.60784 | 49.58423 | 2.094782 | 0.016965 | 0.0208 |
| TMEM105 | 19.29412 | 82.41731 | 2.094786 | 1.55E-15 | 6.93E-15 |
| IL1A | 24.92157 | 106.7048 | 2.098158 | 4.50E-11 | 1.33E-10 |
| MIR7111 | 1.372549 | 5.877898 | 2.098443 | 3.34E-16 | 1.59E-15 |
| RP11-10A14.4 | 3.019608 | 12.94127 | 2.099546 | 1.90E-09 | 4.83E-09 |
| GCM1 | 1.392157 | 5.97527 | 2.101682 | 0.000173 | 0.00027 |
| RP11-606P2.1 | 0.607843 | 2.608964 | 2.101706 | 0.000195 | 0.000303 |
| CXCL6 | 31.27451 | 134.3308 | 2.10273 | 1.15E-06 | 2.23E-06 |
| RP11-181K12.1 | 1.294118 | 5.559505 | 2.102988 | 0.0002 | 0.00031 |
| RNU6-1161P | 0.627451 | 2.695518 | 2.102988 | 1.75E-09 | 4.47E-09 |
| RP1-40G4P.1 | 8.411765 | 36.1762 | 2.10456 | 3.32E-05 | 5.61E-05 |
| RP11-709P2.1 | 0.313725 | 1.349304 | 2.104641 | 4.46E-05 | 7.41E-05 |
| WNT3 | 34.60784 | 148.9289 | 2.105453 | 8.39E-20 | 5.83E-19 |
| GK-IT1 | 1.176471 | 5.068006 | 2.106953 | 3.12E-05 | 5.28E-05 |
| TMEM132A | 369.4902 | 1591.983 | 2.107217 | 1.18E-23 | 1.38E-22 |
| CTD-2535L24.2 | 0.803922 | 3.46677 | 2.108465 | 2.86E-08 | 6.52E-08 |
| EIF4EP1 | 6.254902 | 27.00464 | 2.110148 | 1.67E-06 | 3.19E-06 |
| AQP9 | 60.78431 | 262.9737 | 2.113148 | 2.86E-06 | 5.37E-06 |
| AC144450.1 | 1.72549 | 7.46677 | 2.113478 | 0.034341 | 0.040567 |
| FDPSP5 | 0.470588 | 2.037094 | 2.113976 | 3.40E-07 | 6.98E-07 |
| TMEM213 | 1.960784 | 8.496136 | 2.115376 | 5.55E-05 | 9.14E-05 |
| DNAH5 | 15.45098 | 67.06955 | 2.11796 | 3.01E-12 | 9.90E-12 |
| RPL37P1 | 0.529412 | 2.299845 | 2.119075 | 3.86E-08 | 8.73E-08 |
| C1QBPP2 | 1.058824 | 4.604328 | 2.120528 | 1.66E-07 | 3.51E-07 |
| RP11-391L3.5 | 1.54902 | 6.743431 | 2.122127 | 0.00127 | 0.001793 |
| RP11-14D22.1 | 0.568627 | 2.477589 | 2.123381 | 0.00043 | 0.000642 |
| DBNDD1 | 223.8039 | 975.7403 | 2.124262 | 1.96E-20 | 1.47E-19 |
| RP11-219B4.3 | 0.705882 | 3.083462 | 2.127051 | 1.24E-08 | 2.93E-08 |
| APCDD1 | 944.1176 | 4124.666 | 2.127239 | 0.001134 | 0.00161 |
| NT5DC4 | 5.039216 | 22.017 | 2.127347 | 2.49E-09 | 6.26E-09 |
| RN7SL15P | 0.686275 | 3.004637 | 2.130333 | 0.000656 | 0.000959 |
| PHLDA1 | 1447.373 | 6337.362 | 2.130446 | 8.34E-19 | 5.23E-18 |
| GOLGA7B | 85.35294 | 373.7388 | 2.130518 | 1.17E-13 | 4.39E-13 |
| RP11-290D2.3 | 1.45098 | 6.358578 | 2.131676 | 0.020393 | 0.024765 |
| WDR97 | 18.31373 | 80.36167 | 2.133582 | 2.56E-13 | 9.27E-13 |
| ERICH2 | 8.411765 | 36.92272 | 2.134028 | 3.38E-09 | 8.39E-09 |
| RP11-242J7.1 | 0.647059 | 2.842349 | 2.135115 | 0.022342 | 0.026978 |
| POLD2P1 | 0.647059 | 2.848532 | 2.13825 | 3.35E-06 | 6.23E-06 |
| RP1-41C23.4 | 0.686275 | 3.02473 | 2.139948 | 2.77E-09 | 6.94E-09 |
| LA16c-60H5.7 | 1.411765 | 6.231839 | 2.142158 | 3.77E-05 | 6.31E-05 |
| FAM131C | 4.568627 | 20.19011 | 2.143816 | 5.73E-12 | 1.83E-11 |
| PRR7-AS1 | 8 | 35.36476 | 2.144241 | 6.52E-19 | 4.14E-18 |
| AC004510.3 | 0.352941 | 1.561051 | 2.145018 | 4.81E-05 | 7.97E-05 |
| HCAR3 | 7.705882 | 34.09428 | 2.145498 | 3.51E-05 | 5.91E-05 |
| RP11-807E13.3 | 0.294118 | 1.301391 | 2.145589 | 1.41E-06 | 2.73E-06 |
| DLX4 | 7.764706 | 34.36631 | 2.145992 | 2.41E-15 | 1.06E-14 |
| RP11-152L20.3 | 0.647059 | 2.863988 | 2.146056 | 0.037673 | 0.044278 |
| BNIP3P24 | 0.509804 | 2.262751 | 2.150064 | 0.00529 | 0.006907 |
| RP11-843B15.4 | 2.078431 | 9.236476 | 2.151847 | 1.10E-12 | 3.76E-12 |
| AXIN2 | 1393.686 | 6193.558 | 2.151863 | 9.24E-15 | 3.83E-14 |
| RP11-473M20.9 | 25.07843 | 111.4699 | 2.152135 | 7.41E-06 | 1.33E-05 |
| HNRNPA3P11 | 2.54902 | 11.33076 | 2.15223 | 0.010377 | 0.013055 |
| RPSAP13 | 0.294118 | 1.307573 | 2.152427 | 0.000172 | 0.000268 |
| LRRC8E | 10.54902 | 46.93509 | 2.153558 | 2.29E-18 | 1.36E-17 |
| LRRC9 | 2.176471 | 9.684699 | 2.153717 | 3.78E-06 | 7.01E-06 |
| RP11-234K24.3 | 3.882353 | 17.30294 | 2.156014 | 9.26E-05 | 0.000149 |
| RPS8P3 | 0.72549 | 3.233385 | 2.156017 | 3.23E-06 | 6.03E-06 |
| COL6A4P1 | 2.27451 | 10.14374 | 2.156962 | 4.92E-14 | 1.90E-13 |
| RP4-616B8.6 | 0.686275 | 3.066461 | 2.159717 | 4.34E-10 | 1.17E-09 |
| RP11-283C24.1 | 0.411765 | 1.842349 | 2.161655 | 9.54E-07 | 1.87E-06 |
| IMMP1LP1 | 0.509804 | 2.282844 | 2.162818 | 0.004385 | 0.005786 |
| THBS2 | 1447.353 | 6491.338 | 2.165099 | 5.11E-06 | 9.35E-06 |
| IL17A | 3.313725 | 14.86708 | 2.165595 | 1.51E-06 | 2.91E-06 |
| CGB7 | 0.490196 | 2.20711 | 2.170728 | 0.000414 | 0.000619 |
| RP11-9E13.4 | 0.431373 | 1.942813 | 2.171141 | 3.24E-07 | 6.66E-07 |
| DLL3 | 4.941176 | 22.27666 | 2.172607 | 0.000114 | 0.000182 |
| CTA-390C10.9 | 0.568627 | 2.565688 | 2.17379 | 1.18E-05 | 2.08E-05 |
| PKMP5 | 1.137255 | 5.131376 | 2.17379 | 2.82E-07 | 5.84E-07 |
| RP11-49C9.2 | 1.647059 | 7.432767 | 2.174007 | 9.91E-12 | 3.11E-11 |
| CLDN9 | 18.35294 | 82.97991 | 2.176751 | 2.40E-07 | 5.01E-07 |
| KISS1 | 6.980392 | 31.5796 | 2.177613 | 1.76E-17 | 9.54E-17 |
| WNT8B | 2.078431 | 9.418856 | 2.180057 | 5.78E-10 | 1.54E-09 |
| RP11-322D14.2 | 1.196078 | 5.421947 | 2.180499 | 2.11E-07 | 4.42E-07 |
| RP11-645C24.4 | 1.45098 | 6.582689 | 2.181649 | 5.32E-10 | 1.42E-09 |
| GLS2 | 9.568627 | 43.41886 | 2.181938 | 1.15E-19 | 7.83E-19 |
| RP11-120K18.2 | 0.960784 | 4.366306 | 2.184129 | 7.33E-06 | 1.32E-05 |
| SULT1C2 | 114.098 | 518.5657 | 2.184253 | 1.35E-09 | 3.47E-09 |
| LINCR-0001 | 6 | 27.27512 | 2.184551 | 0.001871 | 0.002589 |
| RP11-568A7.3 | 1.666667 | 7.581144 | 2.18545 | 2.67E-06 | 5.03E-06 |
| SRRM1P3 | 1.666667 | 7.581144 | 2.18545 | 1.88E-07 | 3.96E-07 |
| RP3-405J10.5 | 0.686275 | 3.122102 | 2.18566 | 6.58E-05 | 0.000107 |
| RPL36P16 | 0.686275 | 3.125193 | 2.187088 | 5.02E-10 | 1.34E-09 |
| ODAM | 37.21569 | 169.5085 | 2.187375 | 0.000102 | 0.000163 |
| RP11-181E10.3 | 1.294118 | 5.905719 | 2.190144 | 2.15E-06 | 4.08E-06 |
| RP11-229P13.15 | 0.607843 | 2.775889 | 2.191179 | 1.63E-07 | 3.45E-07 |
| SMCO2 | 2.039216 | 9.338485 | 2.195174 | 5.51E-15 | 2.33E-14 |
| RP11-524D16__A.3 | 16.03922 | 73.46677 | 2.195488 | 6.79E-08 | 1.50E-07 |
| RP11-104E19.1 | 0.45098 | 2.066461 | 2.196025 | 0.002869 | 0.003881 |
| AC021218.2 | 185.4706 | 850.3539 | 2.196873 | 2.01E-18 | 1.20E-17 |
| RP11-67P15.1 | 0.294118 | 1.349304 | 2.197751 | 1.03E-05 | 1.82E-05 |
| ITGBL1 | 54.4902 | 250.2782 | 2.199464 | 0.000943 | 0.001351 |
| SNORD94 | 5.215686 | 23.983 | 2.201083 | 3.26E-06 | 6.08E-06 |
| AC006483.5 | 0.784314 | 3.607419 | 2.201464 | 8.65E-13 | 3.00E-12 |
| AC090505.5 | 0.372549 | 1.715611 | 2.20322 | 5.68E-06 | 1.03E-05 |
| ANKRD1 | 1.568627 | 7.224111 | 2.203317 | 4.06E-05 | 6.79E-05 |
| RP11-216L13.21 | 1.882353 | 8.675425 | 2.204397 | 6.72E-12 | 2.13E-11 |
| FLJ16779 | 6.313725 | 29.13292 | 2.206087 | 9.05E-05 | 0.000146 |
| LINC01564 | 1.490196 | 6.876352 | 2.206141 | 0.000975 | 0.001394 |
| MYO18B | 3.745098 | 17.29366 | 2.207168 | 0.004931 | 0.006465 |
| EGOT | 0.784314 | 3.622102 | 2.207324 | 4.37E-06 | 8.04E-06 |
| RP11-22B23.2 | 0.392157 | 1.812983 | 2.208863 | 2.58E-05 | 4.40E-05 |
| MMP10 | 37.68627 | 174.2751 | 2.209255 | 2.44E-15 | 1.07E-14 |
| RP11-303E16.6 | 0.54902 | 2.542504 | 2.21132 | 0.001234 | 0.001744 |
| C20orf195 | 10.33333 | 47.99536 | 2.215589 | 2.73E-13 | 9.87E-13 |
| RP11-10J21.4 | 1.784314 | 8.301391 | 2.217984 | 2.38E-14 | 9.49E-14 |
| ALG1L3P | 5.588235 | 26.00309 | 2.218218 | 6.00E-06 | 1.09E-05 |
| RP1-68D18.4 | 4.235294 | 19.72798 | 2.219709 | 2.77E-11 | 8.34E-11 |
| RP11-302F12.10 | 0.745098 | 3.471406 | 2.220018 | 0.042148 | 0.049258 |
| RP11-796E10.1 | 4.529412 | 21.11283 | 2.220724 | 1.49E-16 | 7.32E-16 |
| AC092573.2 | 2.313725 | 10.79907 | 2.222618 | 1.21E-10 | 3.42E-10 |
| XKRX | 61.52941 | 287.6121 | 2.224776 | 2.05E-15 | 9.05E-15 |
| ERHP1 | 1.098039 | 5.134467 | 2.225285 | 3.30E-06 | 6.16E-06 |
| TMEM78 | 0.254902 | 1.193199 | 2.226821 | 1.05E-05 | 1.87E-05 |
| DSG1 | 4.960784 | 23.25966 | 2.22919 | 2.62E-10 | 7.20E-10 |
| LINC00618 | 1.098039 | 5.151468 | 2.230054 | 3.07E-12 | 1.01E-11 |
| RPL30P4 | 2.509804 | 11.77898 | 2.230568 | 2.83E-09 | 7.09E-09 |
| TREM1 | 38.66667 | 181.5966 | 2.231575 | 2.41E-09 | 6.07E-09 |
| RP11-449H3.2 | 0.431373 | 2.026275 | 2.231824 | 3.17E-05 | 5.36E-05 |
| PGAM1P7 | 0.392157 | 1.843895 | 2.233254 | 3.21E-06 | 6.00E-06 |
| WNT11 | 199.9608 | 940.5889 | 2.233847 | 0.002065 | 0.002843 |
| ACTR3P2 | 0.431373 | 2.029366 | 2.234023 | 0.000105 | 0.000168 |
| RP11-875O11.2 | 0.509804 | 2.398764 | 2.234277 | 0.000644 | 0.000942 |
| CTD-3035D6.1 | 23.68627 | 111.5008 | 2.234931 | 2.78E-10 | 7.61E-10 |
| KB-1980E6.2 | 0.294118 | 1.384853 | 2.235268 | 2.38E-06 | 4.49E-06 |
| C2orf48 | 7.941176 | 37.40031 | 2.235626 | 1.31E-14 | 5.37E-14 |
| RP11-596D21.1 | 5 | 23.5796 | 2.237539 | 0.001766 | 0.002451 |
| SNORD14E | 2.686275 | 12.67388 | 2.23818 | 1.05E-12 | 3.61E-12 |
| RP11-152C15.1 | 0.705882 | 3.333849 | 2.239689 | 8.25E-08 | 1.80E-07 |
| CTD-2587H19.3 | 0.666667 | 3.151468 | 2.240987 | 0.000312 | 0.000473 |
| RN7SL146P | 0.431373 | 2.040185 | 2.241694 | 2.02E-05 | 3.49E-05 |
| CD300LD | 0.568627 | 2.693972 | 2.244179 | 0.004849 | 0.006364 |
| ERVMER34-1 | 29.13725 | 138.0804 | 2.244571 | 1.47E-05 | 2.57E-05 |
| VPS9D1-AS1 | 108.8431 | 515.8624 | 2.244736 | 8.60E-21 | 6.74E-20 |
| AC005534.8 | 1.705882 | 8.088099 | 2.245282 | 2.51E-11 | 7.59E-11 |
| CYSRT1 | 11.05882 | 52.44513 | 2.245611 | 1.21E-21 | 1.06E-20 |
| PPATP1 | 0.215686 | 1.023184 | 2.246059 | 9.12E-07 | 1.79E-06 |
| CTA-126B4.7 | 3.54902 | 16.85781 | 2.247924 | 3.94E-06 | 7.28E-06 |
| GATA2-AS1 | 33.15686 | 157.6677 | 2.249508 | 4.78E-12 | 1.54E-11 |
| RP11-70L8.4 | 7.431373 | 35.37403 | 2.25099 | 3.42E-09 | 8.48E-09 |
| WDR66 | 25.68627 | 122.3029 | 2.251389 | 3.41E-20 | 2.49E-19 |
| RP5-1025A1.3 | 0.431373 | 2.055641 | 2.252582 | 0.00016 | 0.000251 |
| RP11-429J17.8 | 1.901961 | 9.066461 | 2.253052 | 8.15E-12 | 2.57E-11 |
| SNORD53_SNORD92 | 0.490196 | 2.340031 | 2.255097 | 6.78E-05 | 0.00011 |
| SLC7A11 | 217.0784 | 1038.323 | 2.257967 | 5.94E-16 | 2.76E-15 |
| RP3-388N13.5 | 0.54902 | 2.627512 | 2.258768 | 2.45E-10 | 6.75E-10 |
| RP11-598F7.6 | 2.745098 | 13.1391 | 2.258937 | 1.61E-16 | 7.90E-16 |
| AC016738.3 | 0.313725 | 1.502318 | 2.259616 | 5.76E-05 | 9.45E-05 |
| LAIR2 | 5.411765 | 25.966 | 2.262453 | 3.04E-08 | 6.94E-08 |
| LINC00862 | 0.72549 | 3.482226 | 2.262982 | 1.18E-06 | 2.29E-06 |
| PCSK9 | 559.902 | 2689.179 | 2.26392 | 2.99E-17 | 1.58E-16 |
| PRDX2P1 | 0.54902 | 2.641422 | 2.266385 | 2.39E-05 | 4.10E-05 |
| AC079145.4 | 1.313725 | 6.321484 | 2.266599 | 9.28E-11 | 2.66E-10 |
| CCDC187 | 7.254902 | 34.91963 | 2.26701 | 7.20E-07 | 1.43E-06 |
| RPL12P11 | 0.627451 | 3.020093 | 2.267018 | 0.01425 | 0.017629 |
| RP11-54A9.1 | 0.764706 | 3.684699 | 2.26857 | 1.45E-11 | 4.49E-11 |
| CTD-2553C6.1 | 0.529412 | 2.556414 | 2.271659 | 0.000425 | 0.000635 |
| RNA5SP323 | 0.333333 | 1.61051 | 2.27248 | 1.36E-05 | 2.38E-05 |
| RP11-326C3.13 | 1.588235 | 7.681607 | 2.273984 | 2.37E-08 | 5.45E-08 |
| RP11-82L18.2 | 8.215686 | 39.77743 | 2.275497 | 2.68E-14 | 1.06E-13 |
| LL0XNC01-131B10.2 | 3.882353 | 18.80062 | 2.275777 | 1.96E-08 | 4.54E-08 |
| GBP7 | 1.431373 | 6.936631 | 2.276836 | 1.48E-05 | 2.59E-05 |
| RP11-539L10.4 | 0.352941 | 1.714065 | 2.279922 | 7.07E-06 | 1.27E-05 |
| RP13-46H24.1 | 1.196078 | 5.81762 | 2.282117 | 0.000218 | 0.000336 |
| IFNL3P1 | 1.137255 | 5.534776 | 2.282969 | 6.20E-09 | 1.50E-08 |
| RP3-324O17.7 | 0.745098 | 3.627512 | 2.283478 | 0.000115 | 0.000183 |
| KCNH8 | 46.09804 | 224.4869 | 2.283854 | 2.94E-13 | 1.06E-12 |
| CPSF1P1 | 8.843137 | 43.11592 | 2.28559 | 1.11E-07 | 2.38E-07 |
| SNORA14B | 2.019608 | 9.871716 | 2.289226 | 4.04E-07 | 8.22E-07 |
| NR5A1 | 0.588235 | 2.877898 | 2.29055 | 0.002112 | 0.002903 |
| RP11-560I19.2 | 0.352941 | 1.727975 | 2.291583 | 6.18E-06 | 1.12E-05 |
| AC007899.3 | 0.490196 | 2.401855 | 2.292718 | 0.005039 | 0.006598 |
| RP11-304F15.5 | 0.235294 | 1.153014 | 2.292873 | 5.71E-06 | 1.04E-05 |
| C6orf183 | 24.58824 | 120.8161 | 2.296772 | 6.72E-13 | 2.34E-12 |
| AP000695.4 | 1.803922 | 8.87017 | 2.297825 | 3.82E-13 | 1.36E-12 |
| RP4-545L17.12 | 0.823529 | 4.051005 | 2.298388 | 0.001016 | 0.00145 |
| RNVU1-4 | 0.627451 | 3.089645 | 2.299866 | 1.11E-06 | 2.17E-06 |
| CYP26A1 | 3.27451 | 16.12983 | 2.30038 | 0.003833 | 0.005094 |
| SNORD67 | 0.45098 | 2.223338 | 2.301591 | 1.17E-06 | 2.28E-06 |
| CITED1 | 12.52941 | 61.86862 | 2.303889 | 2.01E-09 | 5.09E-09 |
| RP11-278L15.2 | 1 | 4.938176 | 2.303978 | 9.30E-10 | 2.43E-09 |
| SLC25A39P1 | 0.72549 | 3.582689 | 2.304015 | 0.015544 | 0.019149 |
| RP11-390P2.2 | 0.568627 | 2.808346 | 2.304165 | 7.42E-05 | 0.00012 |
| RP11-467I20.3 | 0.627451 | 3.100464 | 2.304909 | 1.34E-06 | 2.59E-06 |
| CCT7P1 | 0.647059 | 3.199382 | 2.305824 | 8.16E-06 | 1.46E-05 |
| COL4A2-AS2 | 0.568627 | 2.812983 | 2.306545 | 0.000447 | 0.000667 |
| RP11-356I18.1 | 0.411765 | 2.037094 | 2.306621 | 0.00025 | 0.000382 |
| CFAP44-AS1 | 0.490196 | 2.429675 | 2.309333 | 0.009897 | 0.012485 |
| RP11-351M8.1 | 1.039216 | 5.151468 | 2.309489 | 0.000395 | 0.000591 |
| AC058791.1 | 32.66667 | 161.9923 | 2.310034 | 2.34E-15 | 1.03E-14 |
| RP11-15E18.5 | 0.490196 | 2.431221 | 2.31025 | 1.03E-05 | 1.83E-05 |
| AC007966.1 | 2.490196 | 12.35858 | 2.311182 | 5.05E-06 | 9.24E-06 |
| AC073257.2 | 0.666667 | 3.31221 | 2.312757 | 1.82E-07 | 3.83E-07 |
| RP3-380B8.4 | 0.980392 | 4.885626 | 2.317113 | 8.21E-07 | 1.62E-06 |
| B4GALNT4 | 88.62745 | 441.677 | 2.317166 | 0.009015 | 0.011438 |
| MTND5P28 | 1.607843 | 8.032457 | 2.320715 | 7.45E-07 | 1.48E-06 |
| RP11-383I23.2 | 3.313725 | 16.58423 | 2.323286 | 1.06E-11 | 3.32E-11 |
| CHRNA6 | 0.941176 | 4.710974 | 2.323488 | 1.73E-06 | 3.31E-06 |
| LINC01146 | 3.882353 | 19.43895 | 2.323947 | 0.000128 | 0.000203 |
| RP11-690G19.4 | 0.372549 | 1.868624 | 2.326474 | 0.000456 | 0.000679 |
| BGN | 2217.588 | 11128.76 | 2.327229 | 4.31E-14 | 1.68E-13 |
| RP1-20B11.2 | 0.627451 | 3.149923 | 2.327742 | 0.000327 | 0.000494 |
| CTD-2349P21.3 | 4.117647 | 20.68624 | 2.32878 | 0.011658 | 0.014576 |
| FKBP4P6 | 0.411765 | 2.069552 | 2.329426 | 1.48E-06 | 2.85E-06 |
| GJC3 | 5.235294 | 26.34003 | 2.330915 | 2.10E-13 | 7.69E-13 |
| ALDOAP2 | 2.862745 | 14.4034 | 2.330938 | 3.59E-05 | 6.03E-05 |
| SNORD88A | 0.54902 | 2.763524 | 2.33158 | 2.05E-09 | 5.20E-09 |
| RP11-321A17.3 | 1.627451 | 8.211747 | 2.335075 | 1.43E-06 | 2.75E-06 |
| SIX4 | 13.90196 | 70.25657 | 2.337345 | 6.00E-09 | 1.46E-08 |
| LA16c-444G7.2 | 0.470588 | 2.378671 | 2.337618 | 0.003014 | 0.004064 |
| RP11-231I16.1 | 2.235294 | 11.3323 | 2.341904 | 8.00E-06 | 1.44E-05 |
| RP11-659E9.4 | 0.686275 | 3.48068 | 2.342512 | 3.14E-05 | 5.31E-05 |
| NTF4 | 0.54902 | 2.785162 | 2.342832 | 0.014702 | 0.018163 |
| GNG4 | 353.6667 | 1794.938 | 2.343472 | 0.001255 | 0.001773 |
| RP11-165D6.1 | 0.607843 | 3.086553 | 2.344226 | 0.007616 | 0.009755 |
| RP11-573D15.2 | 0.72549 | 3.7017 | 2.35116 | 1.33E-08 | 3.13E-08 |
| SPACA3 | 8.235294 | 42.03246 | 2.351612 | 0.026304 | 0.031505 |
| PCBP2P1 | 0.568627 | 2.904173 | 2.352572 | 2.21E-09 | 5.59E-09 |
| TATDN1P1 | 1.823529 | 9.316847 | 2.353108 | 2.52E-05 | 4.31E-05 |
| RP11-127L20.3 | 1.117647 | 5.710974 | 2.353272 | 6.05E-06 | 1.10E-05 |
| C1QTNF8 | 0.490196 | 2.510046 | 2.356283 | 0.00127 | 0.001793 |
| AC006273.5 | 4.803922 | 24.60433 | 2.356628 | 5.69E-16 | 2.65E-15 |
| NFE2L3 | 1083.569 | 5557.043 | 2.358527 | 6.67E-27 | 1.42E-25 |
| KCNK9 | 6.607843 | 33.90726 | 2.359343 | 4.40E-07 | 8.93E-07 |
| RP4-760C5.3 | 2.529412 | 12.98145 | 2.359578 | 1.01E-10 | 2.90E-10 |
| AC104297.1 | 1.72549 | 8.860896 | 2.360446 | 8.13E-14 | 3.08E-13 |
| CDKN2A | 64.43137 | 331.3246 | 2.36241 | 3.11E-13 | 1.12E-12 |
| BRSK2 | 49.37255 | 253.9382 | 2.362696 | 2.38E-06 | 4.50E-06 |
| DSG1-AS1 | 0.745098 | 3.836167 | 2.364163 | 3.38E-07 | 6.94E-07 |
| RN7SL381P | 4.666667 | 24.03246 | 2.36452 | 0.00641 | 0.008286 |
| RP11-1007I13.4 | 0.294118 | 1.516229 | 2.366022 | 4.01E-06 | 7.41E-06 |
| DDX18P5 | 0.72549 | 3.743431 | 2.367333 | 0.02126 | 0.025747 |
| PLAC1 | 4.27451 | 22.07728 | 2.368732 | 1.32E-05 | 2.32E-05 |
| RP11-54O7.1 | 1.431373 | 7.411128 | 2.372294 | 0.000123 | 0.000195 |
| CTD-2062A1.2 | 0.72549 | 3.760433 | 2.373871 | 0.007565 | 0.009692 |
| RP11-619A14.2 | 1.901961 | 9.859351 | 2.374005 | 0.000686 | 0.001 |
| RP11-83B20.3 | 0.607843 | 3.151468 | 2.374253 | 7.74E-05 | 0.000125 |
| HSPD1P2 | 0.529412 | 2.746522 | 2.375144 | 2.33E-06 | 4.41E-06 |
| RP1-90J4.1 | 0.509804 | 2.647604 | 2.376673 | 1.28E-07 | 2.74E-07 |
| HTR1D | 69.90196 | 363.3076 | 2.377787 | 5.04E-23 | 5.33E-22 |
| RP11-378J18.6 | 0.647059 | 3.366306 | 2.379198 | 1.09E-06 | 2.14E-06 |
| LINC01356 | 7.45098 | 38.77125 | 2.379485 | 4.09E-18 | 2.36E-17 |
| RP11-320N7.2 | 0.960784 | 5.004637 | 2.380981 | 0.002604 | 0.00354 |
| RP11-609L3.1 | 0.411765 | 2.145286 | 2.381278 | 1.75E-06 | 3.34E-06 |
| ONECUT2 | 42.70588 | 223.1453 | 2.385477 | 1.88E-10 | 5.23E-10 |
| WISP1 | 66.43137 | 347.4034 | 2.386675 | 9.42E-16 | 4.30E-15 |
| CTD-2014D20.1 | 1.647059 | 8.616692 | 2.387242 | 0.000157 | 0.000246 |
| LINC00958 | 2.901961 | 15.18238 | 2.387298 | 0.000415 | 0.00062 |
| RP11-496I2.5 | 0.627451 | 3.287481 | 2.389408 | 5.04E-05 | 8.33E-05 |
| AC139103.1 | 0.588235 | 3.086553 | 2.391531 | 0.010035 | 0.012646 |
| PRDM12 | 2.313725 | 12.14838 | 2.392474 | 7.24E-22 | 6.49E-21 |
| RP11-264E20.1 | 0.294118 | 1.545595 | 2.393697 | 2.56E-08 | 5.89E-08 |
| CXCL11 | 124.7451 | 655.9969 | 2.394706 | 1.69E-12 | 5.71E-12 |
| RN7SL28P | 0.509804 | 2.683153 | 2.395915 | 6.36E-07 | 1.27E-06 |
| MYCN | 37.92157 | 200.0866 | 2.399534 | 0.000434 | 0.000647 |
| RP11-42O4.2 | 0.745098 | 3.931994 | 2.399759 | 1.56E-11 | 4.82E-11 |
| PSAT1 | 460.7451 | 2438.764 | 2.404109 | 3.86E-21 | 3.16E-20 |
| NMU | 38.15686 | 202.1097 | 2.405124 | 3.10E-07 | 6.38E-07 |
| CLLU1OS | 1.784314 | 9.460587 | 2.40656 | 0.000199 | 0.000308 |
| CTC-137K3.1 | 0.392157 | 2.080371 | 2.407338 | 0.000194 | 0.000301 |
| RP11-677M24.1 | 0.431373 | 2.289026 | 2.407728 | 7.94E-06 | 1.42E-05 |
| NPW | 13.13725 | 69.74343 | 2.408393 | 8.86E-09 | 2.12E-08 |
| AC004386.4 | 2.45098 | 13.01546 | 2.408795 | 4.20E-07 | 8.53E-07 |
| RP11-598F7.5 | 3.529412 | 18.78516 | 2.412094 | 1.33E-20 | 1.02E-19 |
| MCEMP1 | 9.372549 | 49.88717 | 2.412156 | 6.35E-10 | 1.68E-09 |
| SPAG17 | 2.313725 | 12.31685 | 2.412343 | 4.85E-05 | 8.02E-05 |
| RNU6-377P | 0.509804 | 2.715611 | 2.413262 | 0.000148 | 0.000233 |
| RP11-429J17.7 | 5.137255 | 27.37094 | 2.413576 | 2.12E-18 | 1.27E-17 |
| RP11-734K21.5 | 4.941176 | 26.38794 | 2.416952 | 1.37E-10 | 3.86E-10 |
| WASIR2 | 1.352941 | 7.244204 | 2.420728 | 3.41E-08 | 7.73E-08 |
| COL1A1 | 20605.25 | 110333.8 | 2.420791 | 1.77E-12 | 5.96E-12 |
| PSMA2P3 | 0.294118 | 1.576507 | 2.422266 | 1.06E-07 | 2.28E-07 |
| RP11-20G13.3 | 0.72549 | 3.890263 | 2.42284 | 7.11E-08 | 1.56E-07 |
| ERP27 | 57 | 305.8223 | 2.42366 | 7.93E-07 | 1.57E-06 |
| CXCL1 | 571.2549 | 3066.696 | 2.424478 | 2.05E-19 | 1.36E-18 |
| SH3PXD2A-AS1 | 33.29412 | 178.7543 | 2.424638 | 1.78E-14 | 7.19E-14 |
| AZGP1P1 | 2.784314 | 14.95054 | 2.424804 | 1.81E-07 | 3.81E-07 |
| RP11-697N18.1 | 0.745098 | 4.001546 | 2.425055 | 0.021375 | 0.025875 |
| RP11-544M22.8 | 0.392157 | 2.109737 | 2.427561 | 1.25E-05 | 2.20E-05 |
| ASGR1 | 39.35294 | 211.7156 | 2.427584 | 1.66E-11 | 5.12E-11 |
| MRPL40P1 | 0.352941 | 1.901082 | 2.429321 | 1.48E-07 | 3.15E-07 |
| RBAKDN | 0.372549 | 2.007728 | 2.430062 | 5.33E-05 | 8.77E-05 |
| CXCL3 | 323.7059 | 1746.898 | 2.43204 | 4.36E-20 | 3.13E-19 |
| MAGOH3P | 0.588235 | 3.174652 | 2.432133 | 2.22E-09 | 5.62E-09 |
| RP1-90G24.10 | 0.901961 | 4.87017 | 2.432836 | 1.48E-05 | 2.58E-05 |
| LINC00885 | 0.666667 | 3.602782 | 2.434074 | 0.00013 | 0.000206 |
| NEB | 27.11765 | 146.7233 | 2.435794 | 1.06E-08 | 2.52E-08 |
| GDF15 | 1277.196 | 6913.298 | 2.436394 | 1.78E-22 | 1.74E-21 |
| PAX9 | 16 | 86.84853 | 2.44043 | 1.09E-09 | 2.83E-09 |
| LMO7-AS1 | 2.607843 | 14.18393 | 2.443328 | 5.94E-15 | 2.50E-14 |
| AC012354.8 | 1 | 5.440495 | 2.443738 | 1.88E-05 | 3.25E-05 |
| LINC00176 | 14.56863 | 79.27666 | 2.444031 | 3.50E-06 | 6.52E-06 |
| FUT1 | 63.45098 | 345.4312 | 2.444684 | 5.27E-28 | 1.45E-26 |
| FJX1 | 36.09804 | 196.6445 | 2.445598 | 2.27E-26 | 4.29E-25 |
| OACYLP | 2.235294 | 12.17774 | 2.44571 | 1.14E-16 | 5.69E-16 |
| IL17C | 2.862745 | 15.59969 | 2.446046 | 6.94E-08 | 1.53E-07 |
| AC078883.4 | 0.862745 | 4.7017 | 2.446176 | 0.008398 | 0.010696 |
| MYH16 | 3.960784 | 21.60742 | 2.447669 | 5.13E-13 | 1.81E-12 |
| DNAH2 | 38.27451 | 209.4173 | 2.451925 | 2.86E-16 | 1.37E-15 |
| POU4F1 | 3.745098 | 20.49923 | 2.452494 | 1.19E-05 | 2.10E-05 |
| RYKP1 | 1.509804 | 8.272025 | 2.453879 | 1.47E-10 | 4.13E-10 |
| CELSR3 | 178.6471 | 980.527 | 2.456445 | 5.93E-18 | 3.37E-17 |
| RP11-54O7.18 | 0.392157 | 2.153014 | 2.456855 | 1.01E-05 | 1.79E-05 |
| RDH16 | 7.607843 | 41.79907 | 2.457912 | 4.48E-11 | 1.32E-10 |
| RP11-103B5.4 | 2.627451 | 14.44513 | 2.458848 | 2.02E-11 | 6.15E-11 |
| RP1-10C16.1 | 1.686275 | 9.272025 | 2.459045 | 9.69E-05 | 0.000155 |
| FOXD1 | 15.19608 | 83.5626 | 2.459158 | 0.000135 | 0.000213 |
| MT-TS2 | 0.607843 | 3.347759 | 2.461425 | 0.002258 | 0.003092 |
| RP11-551G24.2 | 0.431373 | 2.377125 | 2.462212 | 0.000457 | 0.00068 |
| CALML3 | 7.27451 | 40.13756 | 2.464031 | 0.005576 | 0.007262 |
| SCDP1 | 0.882353 | 4.868624 | 2.464086 | 8.94E-12 | 2.81E-11 |
| RP11-438N5.2 | 0.431373 | 2.383308 | 2.465959 | 0.000235 | 0.000361 |
| GDPD5 | 279.0392 | 1548.75 | 2.472564 | 9.57E-20 | 6.59E-19 |
| GTF2F2P1 | 0.607843 | 3.37558 | 2.473364 | 2.38E-12 | 7.87E-12 |
| AC013410.2 | 0.333333 | 1.853168 | 2.474957 | 5.28E-07 | 1.06E-06 |
| RP13-463N16.6 | 1.54902 | 8.618238 | 2.476038 | 1.26E-10 | 3.58E-10 |
| RP4-676L2.1 | 0.431373 | 2.400309 | 2.476214 | 0.000135 | 0.000214 |
| ACP7 | 0.764706 | 4.25966 | 2.477761 | 0.003704 | 0.004933 |
| CTD-2349P21.6 | 0.686275 | 3.825348 | 2.478733 | 0.000208 | 0.000321 |
| SNRPCP2 | 0.921569 | 5.139104 | 2.479353 | 2.12E-06 | 4.02E-06 |
| GABRD | 10.5098 | 58.68161 | 2.481173 | 2.21E-23 | 2.46E-22 |
| RP11-229O3.1 | 0.803922 | 4.491499 | 2.48207 | 0.000594 | 0.000873 |
| RP11-422P24.9 | 1.411765 | 7.896445 | 2.483704 | 1.94E-07 | 4.07E-07 |
| AC118754.4 | 0.72549 | 4.058733 | 2.484001 | 2.28E-09 | 5.75E-09 |
| RP11-326C3.15 | 1.568627 | 8.777434 | 2.484297 | 8.05E-05 | 0.00013 |
| TMPRSS3 | 101.0784 | 566.4096 | 2.48637 | 1.18E-13 | 4.41E-13 |
| LINC01269 | 1.372549 | 7.703246 | 2.488609 | 1.21E-08 | 2.85E-08 |
| AC005363.11 | 0.235294 | 1.321484 | 2.489622 | 7.33E-09 | 1.77E-08 |
| RFX8 | 1.862745 | 10.47913 | 2.492017 | 8.65E-16 | 3.96E-15 |
| RP11-360L9.7 | 0.215686 | 1.214838 | 2.493757 | 1.50E-06 | 2.89E-06 |
| RP11-372E1.1 | 13.23529 | 74.61669 | 2.495108 | 2.77E-10 | 7.59E-10 |
| MLXIPL | 315.4706 | 1784.725 | 2.500124 | 3.50E-21 | 2.88E-20 |
| LCN1 | 0.431373 | 2.440495 | 2.500167 | 5.73E-06 | 1.04E-05 |
| EIF4BP5 | 0.627451 | 3.550232 | 2.500339 | 0.027714 | 0.033099 |
| FRGCA | 1.156863 | 6.547141 | 2.500647 | 1.32E-08 | 3.11E-08 |
| CTB-187L3.1 | 0.333333 | 1.887172 | 2.501188 | 3.54E-06 | 6.59E-06 |
| RP1-90G24.6 | 1.294118 | 7.358578 | 2.507458 | 8.33E-05 | 0.000135 |
| CTB-50L17.5 | 0.372549 | 2.123648 | 2.511042 | 2.82E-10 | 7.73E-10 |
| RP5-1009E24.8 | 2.647059 | 15.09274 | 2.511392 | 1.87E-14 | 7.54E-14 |
| RP11-55K22.2 | 0.705882 | 4.02473 | 2.511392 | 9.41E-06 | 1.68E-05 |
| PVT1 | 104.2549 | 594.796 | 2.51228 | 1.48E-28 | 4.68E-27 |
| CILP2 | 15.60784 | 89.06337 | 2.512561 | 1.04E-06 | 2.04E-06 |
| RP11-6F2.5 | 0.294118 | 1.680062 | 2.514049 | 1.14E-08 | 2.70E-08 |
| HOGA1 | 5.431373 | 31.06337 | 2.515826 | 6.96E-09 | 1.68E-08 |
| FAM83H-AS1 | 256.0784 | 1465.611 | 2.516844 | 8.70E-27 | 1.80E-25 |
| ALOXE3 | 1.921569 | 11.00464 | 2.517755 | 1.18E-08 | 2.80E-08 |
| RP11-329J18.4 | 0.470588 | 2.698609 | 2.519679 | 0.005365 | 0.007001 |
| RP11-436H11.1 | 9.137255 | 52.45595 | 2.521274 | 1.27E-06 | 2.46E-06 |
| TMEM74B | 49.37255 | 283.5827 | 2.521988 | 2.20E-18 | 1.31E-17 |
| PPEF1 | 3.117647 | 17.92117 | 2.523136 | 1.23E-16 | 6.12E-16 |
| DLEU7-AS1 | 2.607843 | 15.01546 | 2.525519 | 5.68E-12 | 1.81E-11 |
| RP11-74M13.5 | 0.313725 | 1.806801 | 2.525863 | 8.28E-06 | 1.48E-05 |
| RP11-5P18.10 | 0.411765 | 2.378671 | 2.530264 | 2.78E-08 | 6.36E-08 |
| SNRPFP1 | 0.647059 | 3.738794 | 2.530604 | 8.25E-11 | 2.37E-10 |
| RP11-426C22.4 | 4.098039 | 23.71406 | 2.532737 | 3.46E-10 | 9.38E-10 |
| RP5-837J1.4 | 0.745098 | 4.313756 | 2.533442 | 0.026346 | 0.031551 |
| VSNL1 | 212.2353 | 1232.462 | 2.537807 | 4.52E-19 | 2.91E-18 |
| MSLN | 526.4118 | 3057.756 | 2.53821 | 1.46E-05 | 2.56E-05 |
| HOXB8 | 240.9608 | 1399.869 | 2.538421 | 4.34E-13 | 1.54E-12 |
| TSPEAR-AS2 | 6.568627 | 38.18083 | 2.539185 | 1.22E-08 | 2.88E-08 |
| RP11-686G8.1 | 0.647059 | 3.763524 | 2.540115 | 3.20E-08 | 7.28E-08 |
| LGALS7B | 1.098039 | 6.392581 | 2.541469 | 1.21E-05 | 2.14E-05 |
| EDAR | 130.3137 | 759.3076 | 2.542695 | 4.87E-07 | 9.84E-07 |
| SRP72P2 | 1.745098 | 10.17311 | 2.54338 | 2.27E-05 | 3.89E-05 |
| CYP4X1 | 73.82353 | 430.3895 | 2.54349 | 0.000103 | 0.000164 |
| RP11-493L12.3 | 0.823529 | 4.803709 | 2.544257 | 1.73E-08 | 4.03E-08 |
| SNORD72 | 0.45098 | 2.633694 | 2.545951 | 2.56E-09 | 6.44E-09 |
| KB-1440D3.13 | 0.862745 | 5.043277 | 2.547355 | 1.01E-11 | 3.16E-11 |
| SULT2B1 | 167.2549 | 977.9583 | 2.547724 | 9.37E-24 | 1.11E-22 |
| KISS1R | 2.058824 | 12.04946 | 2.549076 | 4.96E-08 | 1.11E-07 |
| OXTR | 17.09804 | 100.2937 | 2.552328 | 1.13E-20 | 8.68E-20 |
| RP4-725G10.3 | 1.392157 | 8.179289 | 2.554654 | 1.35E-14 | 5.49E-14 |
| LRP8 | 164.4706 | 966.6306 | 2.555135 | 2.55E-26 | 4.78E-25 |
| EN1 | 1 | 5.882535 | 2.556438 | 0.008443 | 0.010751 |
| RP11-10A14.9 | 0.647059 | 3.812983 | 2.558951 | 5.08E-09 | 1.24E-08 |
| AC090945.1 | 2.607843 | 15.43895 | 2.565646 | 5.16E-20 | 3.68E-19 |
| RP11-252I14.2 | 0.647059 | 3.83153 | 2.565952 | 0.026439 | 0.031652 |
| RP11-1084J3.3 | 0.45098 | 2.670788 | 2.566129 | 3.95E-06 | 7.30E-06 |
| MATN3 | 25.68627 | 152.2442 | 2.567318 | 2.63E-09 | 6.60E-09 |
| RP1-146I3.1 | 1.45098 | 8.613601 | 2.569589 | 1.06E-07 | 2.30E-07 |
| RP11-956J14.1 | 0.686275 | 4.07728 | 2.570749 | 0.000856 | 0.001232 |
| SERPINB5 | 437.7843 | 2605.011 | 2.572997 | 2.80E-13 | 1.01E-12 |
| KCNQ1OT1 | 97.84314 | 582.7836 | 2.574418 | 5.95E-09 | 1.45E-08 |
| RP11-734K21.2 | 1.156863 | 6.901082 | 2.576605 | 6.18E-09 | 1.50E-08 |
| IZUMO2 | 3.313725 | 19.82226 | 2.580595 | 0.000317 | 0.00048 |
| CCNO | 61.60784 | 369.2303 | 2.583335 | 7.57E-24 | 9.15E-23 |
| IL23A | 22.33333 | 133.8624 | 2.583481 | 2.36E-19 | 1.55E-18 |
| RP11-713M15.2 | 34.86275 | 209.1036 | 2.58446 | 6.81E-17 | 3.48E-16 |
| CHI3L1 | 215.5098 | 1293.295 | 2.585226 | 3.57E-18 | 2.08E-17 |
| RP11-108K3.2 | 1.352941 | 8.134467 | 2.587949 | 1.88E-12 | 6.31E-12 |
| TAS2R38 | 2.352941 | 14.16692 | 2.589989 | 1.64E-13 | 6.07E-13 |
| RP11-434D9.2 | 0.215686 | 1.299845 | 2.591334 | 4.44E-06 | 8.16E-06 |
| RP11-174O3.3 | 0.588235 | 3.548686 | 2.59282 | 3.25E-11 | 9.70E-11 |
| IFNWP19 | 1.039216 | 6.282844 | 2.595923 | 1.04E-09 | 2.71E-09 |
| TREML3P | 0.54902 | 3.321484 | 2.596898 | 8.58E-07 | 1.69E-06 |
| AC083884.8 | 3.176471 | 19.22411 | 2.59742 | 0.000854 | 0.00123 |
| AL133493.2 | 10.58824 | 64.09274 | 2.597699 | 0.004715 | 0.006197 |
| LGR5 | 557.7059 | 3376.677 | 2.598028 | 8.24E-12 | 2.60E-11 |
| RP3-522D1.1 | 4.431373 | 26.83153 | 2.598104 | 3.14E-17 | 1.65E-16 |
| SEPT14P12 | 0.254902 | 1.544049 | 2.598705 | 2.19E-06 | 4.15E-06 |
| RP4-781L3.1 | 0.176471 | 1.069552 | 2.599507 | 1.36E-06 | 2.63E-06 |
| KRT4 | 2.137255 | 12.98145 | 2.602621 | 0.007171 | 0.009207 |
| MYH4 | 2.588235 | 15.72334 | 2.602867 | 0.000297 | 0.000452 |
| RP4-778K6.1 | 1.313725 | 7.981453 | 2.602988 | 3.79E-07 | 7.74E-07 |
| OR7E85P | 0.45098 | 2.741886 | 2.604032 | 1.31E-05 | 2.31E-05 |
| PSORS1C2 | 3 | 18.25193 | 2.605015 | 2.17E-08 | 5.01E-08 |
| IRX3 | 7.862745 | 47.90108 | 2.606953 | 5.11E-05 | 8.44E-05 |
| VGF | 41.80392 | 254.7805 | 2.607545 | 0.000146 | 0.000229 |
| RP11-295D4.7 | 0.411765 | 2.510046 | 2.607822 | 0.000467 | 0.000694 |
| SLC6A6 | 870.1569 | 5305.966 | 2.608268 | 1.95E-24 | 2.59E-23 |
| TSSC1-IT1 | 0.607843 | 3.707883 | 2.608825 | 1.02E-09 | 2.66E-09 |
| PALM3 | 26.37255 | 160.9567 | 2.609564 | 9.50E-07 | 1.86E-06 |
| RP11-428L21.2 | 0.921569 | 5.627512 | 2.610334 | 1.54E-05 | 2.68E-05 |
| RP11-1023L17.2 | 2.294118 | 14.02009 | 2.611485 | 0.000505 | 0.000748 |
| AC092198.1 | 1.490196 | 9.120556 | 2.61362 | 2.07E-07 | 4.34E-07 |
| RP11-445N18.3 | 1.666667 | 10.21484 | 2.615629 | 2.71E-10 | 7.43E-10 |
| LINC00922 | 0.823529 | 5.060278 | 2.619325 | 1.70E-10 | 4.76E-10 |
| RP11-380G5.3 | 5.078431 | 31.21947 | 2.619991 | 1.37E-08 | 3.22E-08 |
| HBQ1 | 0.980392 | 6.030912 | 2.620945 | 0.000745 | 0.001081 |
| CTC-273B12.10 | 0.470588 | 2.899536 | 2.623285 | 1.17E-13 | 4.38E-13 |
| RP4-781K5.4 | 0.333333 | 2.054096 | 2.623466 | 6.00E-05 | 9.84E-05 |
| PLPP4 | 17.43137 | 107.8918 | 2.629827 | 3.37E-10 | 9.17E-10 |
| FAM132B | 14.09804 | 87.36012 | 2.63148 | 1.45E-17 | 7.92E-17 |
| RP11-752L20.3 | 12.90196 | 79.966 | 2.631796 | 6.01E-09 | 1.46E-08 |
| RPS3AP46 | 1 | 6.202473 | 2.632844 | 1.95E-08 | 4.53E-08 |
| ARMC3 | 1.490196 | 9.248841 | 2.63377 | 0.00373 | 0.004965 |
| RPL7AP4 | 1.058824 | 6.574961 | 2.63452 | 6.98E-08 | 1.54E-07 |
| CLDN14 | 8.843137 | 54.93199 | 2.635016 | 3.10E-14 | 1.22E-13 |
| MYEOV | 190.1961 | 1181.935 | 2.635591 | 1.74E-15 | 7.72E-15 |
| TM4SF19 | 1.901961 | 11.8238 | 2.636135 | 2.07E-12 | 6.90E-12 |
| MUCL1 | 3.470588 | 21.58733 | 2.636932 | 4.81E-05 | 7.97E-05 |
| RP11-305P22.9 | 0.568627 | 3.539413 | 2.637954 | 0.000141 | 0.000222 |
| RP1-90G24.11 | 0.568627 | 3.540958 | 2.638584 | 9.97E-06 | 1.77E-05 |
| LINC01433 | 0.529412 | 3.299845 | 2.639936 | 6.39E-15 | 2.69E-14 |
| POU3F2 | 0.980392 | 6.111283 | 2.640044 | 2.34E-10 | 6.46E-10 |
| CTD-3216D2.5 | 2.705882 | 16.90572 | 2.64334 | 7.28E-20 | 5.09E-19 |
| KLK12 | 33.90196 | 212.238 | 2.646243 | 0.004496 | 0.005923 |
| SLC7A5 | 1257 | 7869.467 | 2.646281 | 6.22E-26 | 1.08E-24 |
| LINC01555 | 5.431373 | 34.00464 | 2.646343 | 1.67E-12 | 5.63E-12 |
| RP13-36G14.3 | 0.45098 | 2.825348 | 2.647292 | 2.48E-06 | 4.68E-06 |
| MIR17HG | 25.35294 | 158.9088 | 2.647974 | 2.08E-16 | 1.01E-15 |
| ATHL1 | 465.5098 | 2921.751 | 2.64995 | 5.22E-21 | 4.20E-20 |
| HES7 | 1.019608 | 6.401855 | 2.650476 | 9.79E-07 | 1.92E-06 |
| MTCO3P43 | 1.156863 | 7.267388 | 2.651219 | 8.71E-07 | 1.72E-06 |
| CTC-343N3.1 | 3.686275 | 23.20866 | 2.654428 | 0.021438 | 0.025949 |
| MEX3A | 114.549 | 722.5981 | 2.657228 | 2.21E-18 | 1.32E-17 |
| AC007790.4 | 0.72549 | 4.578053 | 2.657706 | 0.001203 | 0.001703 |
| CLDN16 | 6.72549 | 42.48377 | 2.659201 | 4.40E-15 | 1.88E-14 |
| RP11-25H12.1 | 0.72549 | 4.598145 | 2.664024 | 4.11E-06 | 7.58E-06 |
| STEAP2-AS1 | 0.352941 | 2.238022 | 2.664724 | 1.14E-08 | 2.69E-08 |
| RP11-1029J19.5 | 3.470588 | 22.10201 | 2.670925 | 3.45E-17 | 1.81E-16 |
| AC007193.6 | 0.470588 | 2.998454 | 2.671682 | 0.000775 | 0.001122 |
| RP11-132A1.4 | 35.01961 | 223.1747 | 2.671938 | 1.32E-15 | 5.94E-15 |
| HSPE1P5 | 0.352941 | 2.253478 | 2.674653 | 1.13E-12 | 3.87E-12 |
| ANOS1 | 62.05882 | 396.5997 | 2.675975 | 6.78E-13 | 2.36E-12 |
| AC073326.3 | 0.470588 | 3.010819 | 2.677619 | 4.50E-06 | 8.28E-06 |
| RNU1-106P | 1.607843 | 10.29675 | 2.678991 | 3.04E-18 | 1.79E-17 |
| RP11-384B12.3 | 0.313725 | 2.009274 | 2.679099 | 9.30E-06 | 1.66E-05 |
| HIST2H3D | 1.45098 | 9.301391 | 2.680418 | 0.012133 | 0.015138 |
| FGF8 | 0.627451 | 4.023184 | 2.680763 | 9.03E-09 | 2.16E-08 |
| LRRN4 | 2.45098 | 15.71561 | 2.680767 | 1.79E-12 | 6.01E-12 |
| MAFG-AS1 | 35.88235 | 230.2318 | 2.681741 | 4.94E-28 | 1.37E-26 |
| RPS19P1 | 6.960784 | 44.72952 | 2.683906 | 3.46E-11 | 1.03E-10 |
| AJUBA | 158.1373 | 1018.821 | 2.687651 | 2.34E-29 | 9.28E-28 |
| INHBA-AS1 | 1.215686 | 7.839258 | 2.688946 | 1.21E-08 | 2.85E-08 |
| EREG | 398.1176 | 2567.428 | 2.689057 | 3.73E-06 | 6.91E-06 |
| MPRIP-AS1 | 0.627451 | 4.054096 | 2.691806 | 0.028118 | 0.033564 |
| ANKRD33 | 0.666667 | 4.31221 | 2.69339 | 1.44E-07 | 3.06E-07 |
| DIAPH2-AS1 | 2.705882 | 17.53478 | 2.696048 | 0.000415 | 0.000621 |
| TNFSF9 | 50.52941 | 327.5641 | 2.696582 | 2.31E-14 | 9.22E-14 |
| REG3A | 1035.039 | 6710.768 | 2.696792 | 1.61E-09 | 4.13E-09 |
| SPTBN2 | 245.6078 | 1594.686 | 2.698844 | 2.60E-26 | 4.87E-25 |
| AC004231.2 | 1.803922 | 11.73725 | 2.701886 | 8.23E-07 | 1.62E-06 |
| PRDX2P4 | 0.686275 | 4.469861 | 2.703372 | 8.84E-08 | 1.92E-07 |
| APELA | 2.313725 | 15.08655 | 2.704974 | 1.63E-15 | 7.28E-15 |
| AC002331.1 | 0.843137 | 5.500773 | 2.705795 | 7.43E-11 | 2.15E-10 |
| SMARCE1P2 | 0.333333 | 2.176198 | 2.706772 | 6.55E-07 | 1.31E-06 |
| RP11-289K10.1 | 0.823529 | 5.384853 | 2.709015 | 1.63E-07 | 3.45E-07 |
| SRPX2 | 192.2941 | 1259.337 | 2.711278 | 3.45E-16 | 1.64E-15 |
| PPP1R14BP2 | 1.294118 | 8.476043 | 2.711422 | 5.67E-12 | 1.81E-11 |
| FER1L4 | 121.7843 | 798.4312 | 2.71284 | 2.74E-14 | 1.09E-13 |
| RP11-84A19.4 | 1.509804 | 9.905719 | 2.7139 | 0.002222 | 0.003046 |
| NKPD1 | 4.568627 | 30.05873 | 2.717951 | 3.08E-21 | 2.55E-20 |
| C1orf105 | 2.705882 | 17.81762 | 2.719134 | 2.86E-19 | 1.87E-18 |
| DUSP4 | 274.7843 | 1811.261 | 2.720623 | 1.28E-12 | 4.35E-12 |
| TDGF1 | 178.9412 | 1179.553 | 2.720683 | 1.27E-14 | 5.19E-14 |
| RP3-323A16.1 | 12.80392 | 84.48686 | 2.722141 | 2.20E-14 | 8.80E-14 |
| CTB-113P19.1 | 1.294118 | 8.554869 | 2.724777 | 5.83E-07 | 1.17E-06 |
| AC010287.1 | 0.647059 | 4.285935 | 2.727641 | 0.02831 | 0.033784 |
| RAET1K | 1.588235 | 10.52859 | 2.728816 | 2.30E-14 | 9.19E-14 |
| KLK11 | 60.54902 | 401.5209 | 2.729299 | 9.29E-05 | 0.000149 |
| ILF2P1 | 0.568627 | 3.771252 | 2.729488 | 3.25E-05 | 5.48E-05 |
| XXbac-BPG308K3.5 | 0.843137 | 5.605873 | 2.7331 | 2.36E-13 | 8.58E-13 |
| ANKUB1 | 0.607843 | 4.05255 | 2.737059 | 0.003723 | 0.004956 |
| AC005592.2 | 2.235294 | 14.94127 | 2.740766 | 2.36E-21 | 1.98E-20 |
| RP11-373E16.1 | 0.588235 | 3.933539 | 2.741363 | 0.000139 | 0.000219 |
| MALAT1 | 1707.686 | 11435.04 | 2.743346 | 0.000113 | 0.00018 |
| HEPHL1 | 13.62745 | 91.26893 | 2.743608 | 0.004893 | 0.006418 |
| RP11-1055B8.2 | 0.960784 | 6.438949 | 2.744541 | 1.02E-05 | 1.81E-05 |
| RP11-54O7.10 | 0.372549 | 2.500773 | 2.746872 | 4.66E-10 | 1.25E-09 |
| SLED1 | 0.72549 | 4.871716 | 2.747402 | 7.46E-05 | 0.000121 |
| GSDMC | 2.372549 | 15.94745 | 2.748816 | 6.43E-18 | 3.64E-17 |
| EPGN | 0.490196 | 3.296754 | 2.749616 | 3.01E-05 | 5.10E-05 |
| RP11-616M22.7 | 0.901961 | 6.066461 | 2.749718 | 8.32E-11 | 2.39E-10 |
| ZNF750 | 5.294118 | 35.66924 | 2.752218 | 0.016113 | 0.019818 |
| TFR2 | 35.52941 | 239.5842 | 2.753447 | 3.73E-19 | 2.42E-18 |
| CDIPT-AS1 | 1.294118 | 8.731066 | 2.754189 | 1.99E-08 | 4.61E-08 |
| ACTBL2 | 0.529412 | 3.57187 | 2.754217 | 0.000966 | 0.001383 |
| AC019129.2 | 1.529412 | 10.36785 | 2.761068 | 5.15E-10 | 1.38E-09 |
| RAET1L | 8.078431 | 54.80989 | 2.762289 | 4.98E-19 | 3.19E-18 |
| TDGF1P3 | 0.764706 | 5.191654 | 2.763217 | 2.91E-11 | 8.74E-11 |
| CTA-339C12.1 | 0.588235 | 4.001546 | 2.766092 | 1.80E-06 | 3.44E-06 |
| BHMG1 | 0.352941 | 2.406491 | 2.769432 | 5.87E-07 | 1.18E-06 |
| CTB-186G2.4 | 0.764706 | 5.217929 | 2.7705 | 0.012016 | 0.015 |
| TBX15 | 5.647059 | 38.60896 | 2.773364 | 2.52E-14 | 1.00E-13 |
| RP11-95M15.2 | 0.588235 | 4.023184 | 2.773872 | 4.41E-14 | 1.71E-13 |
| AP000577.2 | 0.666667 | 4.564142 | 2.775306 | 3.71E-07 | 7.60E-07 |
| RPL13P2 | 4.176471 | 28.61824 | 2.776579 | 2.63E-09 | 6.60E-09 |
| SPATA12 | 6.333333 | 43.4034 | 2.776771 | 9.36E-26 | 1.57E-24 |
| RP11-325E14.5 | 0.352941 | 2.418856 | 2.776825 | 5.38E-05 | 8.85E-05 |
| MIR31HG | 1.313725 | 9.004637 | 2.777004 | 1.67E-11 | 5.14E-11 |
| NOX4 | 16.56863 | 113.6538 | 2.77812 | 8.37E-16 | 3.84E-15 |
| CYP4F8 | 2.823529 | 19.39413 | 2.780048 | 4.87E-06 | 8.93E-06 |
| SBSN | 1.843137 | 12.67079 | 2.781271 | 1.10E-13 | 4.14E-13 |
| SPTBN5 | 43.23529 | 297.3818 | 2.782035 | 9.45E-19 | 5.89E-18 |
| RP11-492E3.2 | 16.11765 | 111.2566 | 2.787177 | 7.67E-19 | 4.83E-18 |
| RP11-278H7.5 | 0.980392 | 6.780526 | 2.789966 | 1.93E-12 | 6.46E-12 |
| AC009502.4 | 0.235294 | 1.627512 | 2.790131 | 1.31E-06 | 2.54E-06 |
| LA16c-352F7.1 | 1.196078 | 8.282844 | 2.791814 | 2.13E-06 | 4.04E-06 |
| PERM1 | 9.019608 | 62.57032 | 2.794342 | 5.91E-24 | 7.27E-23 |
| SIX2 | 9 | 62.49923 | 2.795841 | 0.000912 | 0.001309 |
| PNPT1P1 | 0.352941 | 2.454405 | 2.797874 | 1.02E-06 | 1.99E-06 |
| RPL31P61 | 1.588235 | 11.05255 | 2.798883 | 5.64E-07 | 1.13E-06 |
| AC000067.1 | 0.137255 | 0.955178 | 2.798912 | 1.30E-07 | 2.77E-07 |
| SCARNA9 | 8.117647 | 56.51777 | 2.799571 | 9.04E-09 | 2.16E-08 |
| SHISA2 | 23.43137 | 163.2875 | 2.800901 | 3.76E-19 | 2.43E-18 |
| RP11-717D12.1 | 0.666667 | 4.646059 | 2.80097 | 0.041345 | 0.048369 |
| MYADML2 | 13.92157 | 97.13447 | 2.802662 | 1.04E-13 | 3.92E-13 |
| CTD-2619J13.13 | 0.666667 | 4.658423 | 2.804804 | 1.99E-07 | 4.18E-07 |
| LA16c-325D7.1 | 1.745098 | 12.20093 | 2.805611 | 2.60E-17 | 1.38E-16 |
| RP11-212E4.1 | 0.529412 | 3.7017 | 2.805726 | 0.00143 | 0.002006 |
| TEX19 | 1 | 6.993818 | 2.80608 | 2.84E-07 | 5.88E-07 |
| DCSTAMP | 2.705882 | 18.95209 | 2.808186 | 1.85E-11 | 5.67E-11 |
| OBP2A | 0.686275 | 4.806801 | 2.808219 | 5.69E-07 | 1.14E-06 |
| EVX1 | 38.64706 | 270.7697 | 2.808636 | 1.42E-07 | 3.03E-07 |
| LUCAT1 | 4.098039 | 28.72952 | 2.809528 | 4.40E-12 | 1.42E-11 |
| RP11-389O22.4 | 1.215686 | 8.523957 | 2.809752 | 1.36E-07 | 2.91E-07 |
| CTD-2369P2.4 | 0.529412 | 3.712519 | 2.809936 | 2.88E-13 | 1.04E-12 |
| AZGP1 | 419.8431 | 2950.274 | 2.812926 | 6.06E-20 | 4.29E-19 |
| AC007750.5 | 1.45098 | 10.20402 | 2.814037 | 6.86E-10 | 1.81E-09 |
| SAP18P2 | 0.215686 | 1.517774 | 2.814951 | 4.05E-07 | 8.25E-07 |
| TFAP2A | 34.96078 | 246.8485 | 2.819817 | 1.29E-11 | 4.01E-11 |
| WDR72 | 97.17647 | 686.4096 | 2.820391 | 7.67E-08 | 1.68E-07 |
| PRR7 | 50.96078 | 360.6306 | 2.823062 | 2.14E-28 | 6.50E-27 |
| KLRG2 | 6.470588 | 45.90108 | 2.826559 | 0.000174 | 0.000272 |
| PABPC1L | 162.5882 | 1153.988 | 2.827333 | 1.82E-22 | 1.78E-21 |
| TH | 6.941176 | 49.26893 | 2.827426 | 1.73E-14 | 6.98E-14 |
| KRT87P | 0.72549 | 5.153014 | 2.828388 | 1.96E-10 | 5.46E-10 |
| CTC-348L5.1 | 0.980392 | 6.965997 | 2.828899 | 6.41E-07 | 1.28E-06 |
| RNU2-6P | 1.294118 | 9.197836 | 2.829326 | 0.000243 | 0.000372 |
| AC005264.2 | 0.470588 | 3.346213 | 2.829992 | 6.50E-15 | 2.73E-14 |
| RP11-401P9.6 | 5 | 35.56105 | 2.830298 | 0.001038 | 0.00148 |
| RP5-836N17.4 | 1.176471 | 8.367852 | 2.830392 | 2.25E-12 | 7.47E-12 |
| CTC-529P8.1 | 1.156863 | 8.231839 | 2.830997 | 5.19E-12 | 1.67E-11 |
| PRSS51 | 5.156863 | 36.74189 | 2.83286 | 0.000147 | 0.000232 |
| COL9A3 | 166.3725 | 1188.592 | 2.836764 | 9.59E-07 | 1.88E-06 |
| GLYATL1 | 14.4902 | 103.8377 | 2.841181 | 4.72E-11 | 1.39E-10 |
| RP11-353N14.5 | 12.07843 | 86.72488 | 2.844013 | 4.27E-20 | 3.07E-19 |
| TSPEAR | 9.862745 | 70.87481 | 2.845212 | 6.69E-08 | 1.48E-07 |
| RP11-603K19.1 | 0.784314 | 5.638331 | 2.845765 | 0.000318 | 0.000481 |
| MTATP6P26 | 1.019608 | 7.335394 | 2.84686 | 0.000204 | 0.000315 |
| CTB-43E15.2 | 0.588235 | 4.256569 | 2.855226 | 2.04E-11 | 6.22E-11 |
| RP11-307I14.2 | 0.54902 | 3.978362 | 2.857245 | 4.16E-09 | 1.02E-08 |
| RP11-59D5__B.2 | 2.784314 | 20.2272 | 2.860903 | 3.40E-14 | 1.34E-13 |
| RP11-71H17.8 | 0.843137 | 6.136012 | 2.863462 | 0.003372 | 0.004518 |
| LINC01060 | 0.333333 | 2.434312 | 2.868477 | 4.78E-06 | 8.77E-06 |
| GJA3 | 3.627451 | 26.49923 | 2.868922 | 1.62E-10 | 4.54E-10 |
| SNORD48 | 0.352941 | 2.581144 | 2.870511 | 3.94E-14 | 1.54E-13 |
| LY6G6C | 8.156863 | 59.71716 | 2.872059 | 7.07E-14 | 2.69E-13 |
| RHPN1 | 145.4118 | 1067.142 | 2.875537 | 1.75E-27 | 4.23E-26 |
| AC009362.2 | 2.156863 | 15.84699 | 2.877202 | 2.91E-08 | 6.64E-08 |
| BMP7 | 241.7647 | 1777.42 | 2.878109 | 1.02E-08 | 2.44E-08 |
| LINC01605 | 13.64706 | 100.609 | 2.882097 | 1.24E-23 | 1.44E-22 |
| EIF3IP1 | 0.490196 | 3.619784 | 2.884473 | 3.10E-07 | 6.39E-07 |
| RHBG | 0.745098 | 5.502318 | 2.884537 | 7.29E-13 | 2.54E-12 |
| RP11-386G11.5 | 6.235294 | 46.09428 | 2.886058 | 8.97E-24 | 1.07E-22 |
| LRRC36 | 20.29412 | 150.2148 | 2.887894 | 2.07E-17 | 1.11E-16 |
| RP11-290L1.3 | 0.568627 | 4.211747 | 2.888863 | 3.77E-16 | 1.79E-15 |
| RP11-58O9.2 | 37.01961 | 274.5317 | 2.890611 | 8.81E-14 | 3.33E-13 |
| MIR4648 | 0.156863 | 1.165379 | 2.893224 | 2.24E-07 | 4.67E-07 |
| RP11-100E13.1 | 0.588235 | 4.377125 | 2.895518 | 1.73E-11 | 5.32E-11 |
| RP11-527N22.2 | 2.156863 | 16.06646 | 2.897046 | 1.52E-15 | 6.82E-15 |
| SLC7A11-AS1 | 2.823529 | 21.03709 | 2.897364 | 6.91E-05 | 0.000112 |
| RP1-261D10.1 | 0.607843 | 4.530139 | 2.897784 | 0.002719 | 0.00369 |
| RP11-535A19.1 | 0.372549 | 2.780526 | 2.899855 | 5.09E-12 | 1.64E-11 |
| IGFL2 | 13.78431 | 102.9196 | 2.900419 | 0.00028 | 0.000427 |
| ARF1P2 | 0.431373 | 3.22102 | 2.900511 | 1.20E-05 | 2.11E-05 |
| PTGES2-AS1 | 4.705882 | 35.16229 | 2.901492 | 1.99E-22 | 1.93E-21 |
| TGFBI | 5006.255 | 37409.75 | 2.901611 | 3.55E-26 | 6.51E-25 |
| RP11-338N10.2 | 0.666667 | 4.982998 | 2.901977 | 5.86E-09 | 1.42E-08 |
| RP11-138A9.1 | 5.411765 | 40.50696 | 2.903999 | 0.003563 | 0.004757 |
| CTD-2047H16.2 | 3.588235 | 26.98145 | 2.910622 | 1.23E-20 | 9.47E-20 |
| APLN | 52.54902 | 396.2396 | 2.914637 | 6.17E-26 | 1.08E-24 |
| RP11-186F10.2 | 1.823529 | 13.77125 | 2.916854 | 5.20E-12 | 1.67E-11 |
| RP11-875O11.3 | 2.882353 | 21.85162 | 2.922422 | 2.85E-18 | 1.68E-17 |
| TECRP2 | 0.529412 | 4.021638 | 2.925321 | 7.53E-06 | 1.35E-05 |
| TBX4 | 4.686275 | 35.62751 | 2.926478 | 0.017422 | 0.021338 |
| HCRT | 0.607843 | 4.644513 | 2.933756 | 8.35E-10 | 2.19E-09 |
| MIR3189 | 2.568627 | 19.63988 | 2.934716 | 1.82E-15 | 8.07E-15 |
| HAGHL | 50.39216 | 385.5193 | 2.935532 | 2.90E-26 | 5.40E-25 |
| C2-AS1 | 0.960784 | 7.355487 | 2.936536 | 9.73E-17 | 4.89E-16 |
| LINC01267 | 0.647059 | 4.964451 | 2.939665 | 5.76E-08 | 1.28E-07 |
| CTD-2357A8.3 | 2.431373 | 18.70479 | 2.943565 | 1.60E-18 | 9.72E-18 |
| C2orf70 | 27.56863 | 212.136 | 2.94389 | 6.54E-24 | 8.00E-23 |
| MIR503HG | 7.803922 | 60.10201 | 2.945142 | 6.28E-18 | 3.55E-17 |
| RP11-829H16.2 | 1.470588 | 11.33694 | 2.946566 | 1.34E-05 | 2.35E-05 |
| C9orf57 | 0.313725 | 2.421947 | 2.948593 | 9.56E-08 | 2.07E-07 |
| COL7A1 | 206.5686 | 1596.45 | 2.950174 | 2.82E-22 | 2.68E-21 |
| RNF183 | 25.88235 | 200.5487 | 2.953912 | 1.51E-22 | 1.49E-21 |
| SH3TC2 | 58.86275 | 456.1128 | 2.953964 | 7.72E-25 | 1.10E-23 |
| AQP2 | 0.411765 | 3.191654 | 2.954412 | 1.94E-05 | 3.35E-05 |
| SCARNA8 | 0.647059 | 5.018547 | 2.955301 | 0.000301 | 0.000456 |
| FAP | 74.88235 | 583.456 | 2.961926 | 2.06E-15 | 9.07E-15 |
| CTD-2527I21.15 | 1.098039 | 8.565688 | 2.96364 | 0.000514 | 0.000761 |
| RP11-785D18.3 | 4.156863 | 32.44204 | 2.964298 | 5.29E-08 | 1.18E-07 |
| TPT1P12 | 1.392157 | 10.86708 | 2.964571 | 2.98E-06 | 5.58E-06 |
| RP11-568K15.2 | 1.039216 | 8.122102 | 2.966358 | 2.94E-11 | 8.84E-11 |
| SH2D5 | 2.45098 | 19.17311 | 2.967653 | 1.20E-14 | 4.90E-14 |
| RP11-60A8.1 | 5.607843 | 43.87944 | 2.968027 | 0.002939 | 0.003969 |
| ACAN | 54.43137 | 428.4436 | 2.976595 | 5.35E-25 | 7.83E-24 |
| AC112721.1 | 0.333333 | 2.62442 | 2.976961 | 8.08E-08 | 1.77E-07 |
| RPL34P31 | 2.568627 | 20.34158 | 2.985362 | 2.34E-06 | 4.43E-06 |
| SFRP4 | 254.1176 | 2020.793 | 2.991353 | 5.09E-07 | 1.03E-06 |
| AF196972.3 | 0.607843 | 4.837713 | 2.992554 | 1.70E-14 | 6.85E-14 |
| RP11-459A10.1 | 2.078431 | 16.5456 | 2.99288 | 5.24E-06 | 9.57E-06 |
| ANKRD20A19P | 0.392157 | 3.132921 | 2.998006 | 8.09E-08 | 1.77E-07 |
| RP11-333E13.2 | 2.392157 | 19.12056 | 2.998741 | 2.17E-13 | 7.90E-13 |
| RP11-366L20.2 | 5.117647 | 40.97527 | 3.001201 | 1.36E-08 | 3.20E-08 |
| WT1-AS | 0.901961 | 7.230294 | 3.002918 | 0.026905 | 0.032189 |
| TBX20 | 2.372549 | 19.07573 | 3.007229 | 0.004476 | 0.0059 |
| MSX1 | 35.66667 | 287.473 | 3.010778 | 3.12E-24 | 4.00E-23 |
| CAMTA1-IT1 | 0.176471 | 1.423493 | 3.011936 | 1.99E-07 | 4.18E-07 |
| RP13-487P22.1 | 0.607843 | 4.916538 | 3.015872 | 0.019773 | 0.024048 |
| HIST2H2BF | 12.19608 | 98.80989 | 3.018238 | 0.000327 | 0.000494 |
| OSBPL10-AS1 | 0.588235 | 4.766615 | 3.0185 | 0.026995 | 0.032294 |
| TERC | 0.509804 | 4.136012 | 3.020226 | 0.001266 | 0.001788 |
| TMPRSS13 | 56.11765 | 456.4065 | 3.023793 | 5.53E-15 | 2.34E-14 |
| RP11-93K22.6 | 0.215686 | 1.75425 | 3.023848 | 1.69E-06 | 3.24E-06 |
| VAC14-AS1 | 3.27451 | 26.67852 | 3.026328 | 1.48E-22 | 1.46E-21 |
| LINC01615 | 1.431373 | 11.6847 | 3.029149 | 4.17E-17 | 2.17E-16 |
| AC013439.4 | 0.764706 | 6.265842 | 3.034532 | 4.35E-08 | 9.79E-08 |
| CSF2 | 3.039216 | 24.9119 | 3.035064 | 1.02E-15 | 4.62E-15 |
| RP4-738P15.1 | 0.647059 | 5.306028 | 3.035663 | 0.013045 | 0.01622 |
| KIF26B | 78.68627 | 645.6337 | 3.036532 | 1.16E-17 | 6.39E-17 |
| AP001429.1 | 1.529412 | 12.55178 | 3.036843 | 6.18E-05 | 0.000101 |
| CXCL8 | 574.6471 | 4721.75 | 3.038573 | 8.89E-17 | 4.48E-16 |
| NPFFR1 | 20.66667 | 170.4838 | 3.044257 | 6.18E-19 | 3.93E-18 |
| MIOX | 1.803922 | 14.96136 | 3.052033 | 3.81E-10 | 1.03E-09 |
| SLCO4A1 | 356.7843 | 2965.255 | 3.055032 | 6.92E-29 | 2.41E-27 |
| TNS4 | 647.1176 | 5388.068 | 3.057668 | 3.47E-22 | 3.26E-21 |
| CTD-2666L21.3 | 5.372549 | 44.7357 | 3.057748 | 7.56E-11 | 2.18E-10 |
| RP5-856G1.1 | 0.313725 | 2.632148 | 3.068666 | 1.27E-06 | 2.46E-06 |
| RPL18P13 | 2 | 16.78362 | 3.068982 | 1.59E-07 | 3.36E-07 |
| TRPM2-AS | 21.31373 | 178.932 | 3.069557 | 6.70E-16 | 3.10E-15 |
| CTD-2171N6.1 | 1.411765 | 11.85626 | 3.070077 | 5.13E-11 | 1.51E-10 |
| PLEKHN1 | 20.5098 | 172.6986 | 3.073871 | 1.72E-28 | 5.37E-27 |
| RP11-126H7.4 | 0.705882 | 5.967543 | 3.079637 | 3.20E-05 | 5.41E-05 |
| AC005307.1 | 2.72549 | 23.0711 | 3.081499 | 0.001529 | 0.002136 |
| TMPRSS5 | 10.35294 | 87.66306 | 3.081928 | 1.53E-10 | 4.29E-10 |
| GAS6-AS1 | 75 | 635.7264 | 3.083444 | 8.40E-18 | 4.70E-17 |
| AC092168.2 | 1.392157 | 11.81453 | 3.085168 | 4.08E-05 | 6.81E-05 |
| AC018641.7 | 1.019608 | 8.659969 | 3.086347 | 2.06E-07 | 4.32E-07 |
| RP11-1029J19.4 | 5.411765 | 45.99227 | 3.08722 | 1.14E-21 | 9.91E-21 |
| TFAP2A-AS1 | 3.647059 | 31.06646 | 3.090553 | 9.01E-14 | 3.40E-13 |
| RP11-497G19.1 | 3.098039 | 26.39258 | 3.090705 | 0.000201 | 0.000312 |
| NAT16 | 1.294118 | 11.02782 | 3.091107 | 1.82E-09 | 4.65E-09 |
| CTA-414D7.1 | 1.745098 | 14.88253 | 3.09224 | 6.09E-07 | 1.22E-06 |
| GRIN2B | 36.62745 | 312.4158 | 3.09247 | 1.87E-20 | 1.40E-19 |
| RP11-2J18.1 | 0.607843 | 5.185471 | 3.092704 | 0.030583 | 0.036358 |
| RP11-1008C21.1 | 1.843137 | 15.73107 | 3.093381 | 7.52E-13 | 2.61E-12 |
| CKMT2 | 73.31373 | 625.9428 | 3.093876 | 0.018482 | 0.02255 |
| RP11-405F3.5 | 0.352941 | 3.02473 | 3.099306 | 2.41E-05 | 4.12E-05 |
| HS6ST2 | 77.41176 | 663.6677 | 3.099836 | 9.27E-06 | 1.65E-05 |
| CGREF1 | 129.0784 | 1107.216 | 3.100617 | 8.32E-26 | 1.40E-24 |
| RP11-326E7.1 | 0.411765 | 3.53323 | 3.101096 | 1.50E-08 | 3.52E-08 |
| KIAA1257 | 21.80392 | 188.2504 | 3.109993 | 2.71E-26 | 5.05E-25 |
| RPS24P16 | 0.529412 | 4.587326 | 3.115191 | 2.72E-06 | 5.12E-06 |
| AC073283.7 | 2.078431 | 18.04637 | 3.118141 | 4.22E-15 | 1.81E-14 |
| SNORA28 | 0.784314 | 6.825348 | 3.1214 | 0.004173 | 0.005521 |
| RP11-803D5.1 | 1.019608 | 8.896445 | 3.125215 | 9.05E-13 | 3.13E-12 |
| CSTL1 | 0.882353 | 7.724884 | 3.130086 | 2.06E-12 | 6.86E-12 |
| RP11-1430O6.1 | 0.235294 | 2.060278 | 3.130302 | 2.51E-06 | 4.73E-06 |
| IGFL3 | 1.176471 | 10.34158 | 3.135919 | 0.000564 | 0.000831 |
| SNORD12B | 1.490196 | 13.12056 | 3.138255 | 5.50E-14 | 2.12E-13 |
| GRK1 | 2.058824 | 18.1391 | 3.139211 | 6.71E-09 | 1.62E-08 |
| RP11-150O12.6 | 13.45098 | 118.5672 | 3.139922 | 1.10E-23 | 1.29E-22 |
| RP11-278H7.4 | 1.137255 | 10.03246 | 3.141047 | 6.58E-17 | 3.36E-16 |
| RP11-336A10.2 | 0.960784 | 8.49459 | 3.14426 | 0.039282 | 0.046078 |
| LINC00545 | 0.372549 | 3.299845 | 3.146896 | 1.96E-05 | 3.38E-05 |
| METTL11B | 0.27451 | 2.432767 | 3.147668 | 1.26E-08 | 2.97E-08 |
| AC004988.1 | 1.098039 | 9.763524 | 3.152472 | 5.82E-10 | 1.55E-09 |
| GIF | 8.803922 | 78.43122 | 3.15521 | 2.00E-07 | 4.20E-07 |
| SNORD70 | 0.127451 | 1.136012 | 3.155964 | 2.11E-05 | 3.63E-05 |
| RNF182 | 7.117647 | 63.45904 | 3.156353 | 0.001535 | 0.002143 |
| CACNG8 | 4.607843 | 41.37249 | 3.166508 | 1.77E-16 | 8.67E-16 |
| UROC1 | 0.54902 | 4.935085 | 3.168145 | 1.81E-06 | 3.45E-06 |
| RP3-416H24.1 | 1.078431 | 9.741886 | 3.175267 | 1.46E-14 | 5.91E-14 |
| AC137723.5 | 0.45098 | 4.075734 | 3.175923 | 8.34E-12 | 2.63E-11 |
| PRSS22 | 108.6275 | 983.6151 | 3.178705 | 9.55E-27 | 1.96E-25 |
| CORIN | 11.82353 | 107.1623 | 3.180065 | 8.44E-18 | 4.72E-17 |
| AC104809.4 | 2.745098 | 24.88563 | 3.180383 | 0.002086 | 0.002869 |
| SNORD12C | 0.392157 | 3.568779 | 3.185928 | 1.46E-10 | 4.12E-10 |
| ABCA12 | 9.078431 | 83.01391 | 3.192838 | 0.000169 | 0.000264 |
| F7 | 10.05882 | 92.05873 | 3.194093 | 1.23E-05 | 2.17E-05 |
| SCARNA22 | 1.137255 | 10.42349 | 3.196211 | 3.00E-05 | 5.09E-05 |
| VTCN1 | 2.176471 | 19.98609 | 3.198934 | 1.23E-06 | 2.38E-06 |
| SLC25A6P2 | 2.588235 | 23.80989 | 3.20152 | 2.55E-07 | 5.30E-07 |
| ANKRD18B | 1.568627 | 14.45131 | 3.203626 | 0.014812 | 0.018293 |
| EGFL6 | 16.45098 | 152.0325 | 3.208134 | 5.12E-25 | 7.53E-24 |
| CTC-508F8.1 | 0.529412 | 4.893354 | 3.208361 | 1.34E-05 | 2.36E-05 |
| RP1-142L7.9 | 0.509804 | 4.721793 | 3.21132 | 4.16E-06 | 7.67E-06 |
| ATG9B | 25.86275 | 239.7079 | 3.21233 | 2.07E-25 | 3.28E-24 |
| ADAMTS12 | 104.5882 | 970.3385 | 3.213768 | 1.94E-23 | 2.18E-22 |
| RP11-462G2.1 | 52.70588 | 489.0711 | 3.214008 | 0.00082 | 0.001183 |
| MDFI | 61.05882 | 567.5332 | 3.216433 | 1.28E-28 | 4.11E-27 |
| SLAMF9 | 2.156863 | 20.04791 | 3.216446 | 1.15E-17 | 6.34E-17 |
| RP11-264E20.2 | 0.137255 | 1.279753 | 3.220935 | 8.11E-09 | 1.95E-08 |
| RP11-326C3.2 | 23.13725 | 216.9722 | 3.22922 | 2.74E-14 | 1.09E-13 |
| RN7SL838P | 0.470588 | 4.414219 | 3.229621 | 0.000269 | 0.000411 |
| SP5 | 56.39216 | 529.6244 | 3.231403 | 7.00E-20 | 4.91E-19 |
| LHX5 | 0.588235 | 5.539413 | 3.235268 | 1.16E-05 | 2.04E-05 |
| LRP2 | 1.666667 | 15.71097 | 3.236735 | 0.00022 | 0.000339 |
| BTBD16 | 5.509804 | 51.95827 | 3.23728 | 1.57E-23 | 1.78E-22 |
| ST7-OT4 | 1.313725 | 12.40185 | 3.23882 | 4.40E-08 | 9.88E-08 |
| RP11-529H22.1 | 0.45098 | 4.262751 | 3.240648 | 0.002744 | 0.003722 |
| CCDC78 | 14.4902 | 137.6414 | 3.247766 | 2.29E-25 | 3.60E-24 |
| RP1-224A6.8 | 0.686275 | 6.528594 | 3.249915 | 0.004934 | 0.006468 |
| PTTG3P | 0.352941 | 3.36476 | 3.253004 | 4.30E-10 | 1.16E-09 |
| KRT7 | 53.43137 | 509.8099 | 3.2542 | 7.20E-16 | 3.32E-15 |
| AC112721.2 | 0.45098 | 4.306028 | 3.255221 | 7.60E-08 | 1.67E-07 |
| AC005702.3 | 0.509804 | 4.887172 | 3.260985 | 0.000546 | 0.000806 |
| KLHL35 | 23.21569 | 223.1283 | 3.264702 | 8.24E-31 | 6.18E-29 |
| KRT83 | 1.27451 | 12.27048 | 3.267177 | 1.29E-10 | 3.66E-10 |
| AL163953.2 | 3.254902 | 31.53941 | 3.27647 | 1.67E-18 | 1.01E-17 |
| C17orf77 | 10.33333 | 100.2071 | 3.277607 | 1.13E-05 | 2.00E-05 |
| CLEC5A | 13.70588 | 133.102 | 3.279665 | 4.43E-17 | 2.30E-16 |
| ALDH3B2 | 9.686275 | 94.23338 | 3.282224 | 2.00E-14 | 8.05E-14 |
| FAM222A-AS1 | 4.392157 | 42.73107 | 3.282284 | 7.22E-24 | 8.76E-23 |
| CACNG4 | 21.7451 | 212.0665 | 3.285754 | 0.036216 | 0.042635 |
| AC005702.4 | 0.509804 | 4.98609 | 3.289894 | 6.33E-07 | 1.26E-06 |
| RP11-728G15.1 | 0.823529 | 8.1051 | 3.298938 | 5.27E-12 | 1.69E-11 |
| LL22NC03-N14H11.1 | 0.803922 | 7.94745 | 3.305365 | 7.47E-08 | 1.64E-07 |
| PRKCG | 9.862745 | 97.60896 | 3.306952 | 2.12E-07 | 4.44E-07 |
| MTND4P26 | 1.313725 | 13.02937 | 3.310031 | 3.48E-08 | 7.90E-08 |
| LEMD1-AS1 | 0.54902 | 5.499227 | 3.324299 | 1.13E-09 | 2.94E-09 |
| AC017002.2 | 0.529412 | 5.310665 | 3.32643 | 0.00101 | 0.001443 |
| GJB4 | 16.31373 | 163.8269 | 3.328014 | 2.13E-19 | 1.41E-18 |
| GNGT1 | 1.72549 | 17.34312 | 3.329285 | 8.54E-06 | 1.53E-05 |
| RP11-20L24.1 | 0.235294 | 2.367852 | 3.331042 | 9.46E-06 | 1.68E-05 |
| LINC00658 | 0.568627 | 5.749614 | 3.337909 | 3.06E-06 | 5.71E-06 |
| PNPLA3 | 5.764706 | 58.46213 | 3.342184 | 3.10E-09 | 7.72E-09 |
| LINC01485 | 2.490196 | 25.28748 | 3.344092 | 1.62E-08 | 3.78E-08 |
| RP11-353N14.4 | 11.72549 | 119.6569 | 3.351183 | 4.19E-21 | 3.42E-20 |
| RP11-493L12.6 | 0.54902 | 5.607419 | 3.352407 | 1.73E-17 | 9.39E-17 |
| FAM150A | 7.862745 | 80.49304 | 3.355759 | 5.99E-17 | 3.07E-16 |
| SERPINA7 | 5.058824 | 51.81453 | 3.356483 | 0.020505 | 0.024889 |
| C17orf96 | 62.56863 | 641.8485 | 3.358722 | 3.24E-28 | 9.43E-27 |
| ATP5G1P6 | 0.333333 | 3.426584 | 3.361734 | 2.30E-08 | 5.31E-08 |
| RNU4ATAC | 1.176471 | 12.09892 | 3.362341 | 0.000535 | 0.00079 |
| CBX2 | 65.70588 | 676.66 | 3.364337 | 3.43E-27 | 7.73E-26 |
| CPN1 | 4.039216 | 41.91036 | 3.37516 | 0.034176 | 0.040381 |
| C15orf54 | 0.470588 | 4.901082 | 3.380563 | 1.90E-07 | 4.00E-07 |
| UNC93A | 16.19608 | 168.8238 | 3.381802 | 3.17E-12 | 1.04E-11 |
| RP11-159D23.2 | 0.294118 | 3.066461 | 3.382109 | 3.57E-09 | 8.84E-09 |
| TESC-AS1 | 0.137255 | 1.431221 | 3.382317 | 4.42E-08 | 9.93E-08 |
| U1 | 0.502262 | 5.250149 | 3.385845 | 1.40E-08 | 3.28E-08 |
| SNORA22 | 0.651961 | 6.828825 | 3.38878 | 0.02369 | 0.028511 |
| SAA2 | 27.4902 | 288.2009 | 3.390086 | 1.00E-07 | 2.17E-07 |
| RP4-598P13.1 | 0.529412 | 5.553323 | 3.390889 | 0.000638 | 0.000934 |
| CTD-3064M3.4 | 0.431373 | 4.537867 | 3.395008 | 2.29E-14 | 9.15E-14 |
| RP11-290L1.5 | 0.333333 | 3.513138 | 3.397723 | 5.10E-08 | 1.14E-07 |
| STC2 | 115.9804 | 1223.291 | 3.398814 | 7.66E-21 | 6.03E-20 |
| RP1-40E16.9 | 0.72549 | 7.667697 | 3.401765 | 0.020623 | 0.025022 |
| NANOS3 | 6.882353 | 72.75734 | 3.402119 | 7.27E-17 | 3.70E-16 |
| VENTX | 18.72549 | 198.51 | 3.406137 | 0.000268 | 0.00041 |
| SLCO4A1-AS1 | 41.11765 | 436.5131 | 3.408195 | 3.56E-18 | 2.07E-17 |
| LA16c-380H5.3 | 4.372549 | 46.4745 | 3.409893 | 3.08E-12 | 1.01E-11 |
| LBP | 15.5098 | 165.4652 | 3.415276 | 1.13E-06 | 2.19E-06 |
| RP11-568J23.1 | 1.803922 | 19.27512 | 3.417531 | 0.000506 | 0.000749 |
| SIX1 | 3.666667 | 39.28903 | 3.421585 | 6.45E-18 | 3.65E-17 |
| KDELC1P1 | 1.078431 | 11.5796 | 3.424579 | 2.16E-13 | 7.90E-13 |
| TRIB3 | 304.1961 | 3284.199 | 3.432468 | 2.37E-30 | 1.38E-28 |
| S100A11P1 | 0.254902 | 2.760433 | 3.43688 | 1.79E-10 | 5.01E-10 |
| ASCL2 | 390.6275 | 4235.175 | 3.438556 | 7.66E-22 | 6.84E-21 |
| AF064858.11 | 10.41176 | 112.8856 | 3.438575 | 1.59E-05 | 2.78E-05 |
| EVA1A | 34.58824 | 375.8532 | 3.441816 | 3.23E-25 | 4.92E-24 |
| SPDYC | 3.098039 | 33.70634 | 3.443593 | 1.54E-15 | 6.86E-15 |
| RP11-423E7.2 | 1.392157 | 15.18083 | 3.446857 | 1.10E-05 | 1.95E-05 |
| CLLU1 | 0.45098 | 4.928903 | 3.45013 | 3.23E-05 | 5.46E-05 |
| RP5-907D15.4 | 0.627451 | 6.877898 | 3.454393 | 4.30E-16 | 2.03E-15 |
| HOTAIR | 2.392157 | 26.26275 | 3.456634 | 0.001425 | 0.002 |
| RP11-346D19.1 | 0.745098 | 8.190108 | 3.45838 | 1.94E-15 | 8.56E-15 |
| RP11-392O1.4 | 0.627451 | 6.907264 | 3.46054 | 4.00E-14 | 1.56E-13 |
| AMH | 10.80392 | 119.3354 | 3.465395 | 2.52E-13 | 9.12E-13 |
| MUC5AC | 174.7843 | 1941.172 | 3.47328 | 3.88E-08 | 8.77E-08 |
| SCARNA12 | 4.019608 | 44.66461 | 3.474005 | 6.71E-11 | 1.95E-10 |
| AFP | 1.098039 | 12.21793 | 3.475998 | 1.33E-09 | 3.44E-09 |
| CPA4 | 1.431373 | 15.95209 | 3.478274 | 2.99E-17 | 1.58E-16 |
| OLR1 | 22.19608 | 247.5533 | 3.479363 | 8.50E-15 | 3.53E-14 |
| LINC01549 | 0.647059 | 7.219474 | 3.479925 | 4.20E-07 | 8.54E-07 |
| RP4-604A21.1 | 6.235294 | 69.70325 | 3.482696 | 5.81E-08 | 1.29E-07 |
| FGF19 | 13.4902 | 150.8393 | 3.483029 | 7.18E-21 | 5.68E-20 |
| TMEM249 | 2.352941 | 26.38176 | 3.487004 | 5.58E-19 | 3.55E-18 |
| STK31 | 12.92157 | 145.5935 | 3.494093 | 4.43E-21 | 3.60E-20 |
| DUXAP9 | 1.137255 | 12.81762 | 3.494501 | 5.21E-09 | 1.27E-08 |
| TMEM211 | 13.39216 | 151.0247 | 3.495325 | 4.23E-14 | 1.65E-13 |
| RP1-79C4.4 | 0.607843 | 6.877898 | 3.500197 | 2.22E-09 | 5.61E-09 |
| IGFBP1 | 3.490196 | 39.69397 | 3.50754 | 0.003241 | 0.004351 |
| MAT1A | 20.98039 | 239.3818 | 3.5122 | 8.13E-07 | 1.60E-06 |
| TBL1XR1-AS1 | 0.490196 | 5.595054 | 3.512721 | 0.000149 | 0.000233 |
| HCAR1 | 9.392157 | 107.5641 | 3.517597 | 4.82E-05 | 7.98E-05 |
| PCMTD1P3 | 0.333333 | 3.81762 | 3.517636 | 4.28E-12 | 1.39E-11 |
| SNHG25 | 13.62745 | 156.3292 | 3.52 | 2.66E-21 | 2.21E-20 |
| CTHRC1 | 113 | 1298.099 | 3.522006 | 4.04E-24 | 5.09E-23 |
| RPL35AP21 | 0.529412 | 6.097372 | 3.525726 | 2.74E-07 | 5.67E-07 |
| WFDC10A | 0.411765 | 4.743431 | 3.526039 | 5.24E-11 | 1.54E-10 |
| MUC16 | 5.72549 | 66.13292 | 3.529898 | 0.026787 | 0.032058 |
| TRIM29 | 304.1765 | 3518.521 | 3.531989 | 4.37E-25 | 6.49E-24 |
| RP11-68I3.7 | 0.215686 | 2.510046 | 3.540708 | 3.00E-11 | 8.99E-11 |
| RP11-1193F23.1 | 0.843137 | 9.842349 | 3.545163 | 6.71E-05 | 0.00011 |
| AC009410.1 | 1.372549 | 16.05564 | 3.548151 | 0.000556 | 0.000819 |
| RP11-353N14.2 | 2.294118 | 26.86553 | 3.549745 | 1.05E-24 | 1.46E-23 |
| CTD-2600O9.1 | 0.137255 | 1.61051 | 3.552588 | 3.82E-08 | 8.62E-08 |
| HOXC11 | 5.392157 | 63.30139 | 3.553303 | 1.85E-06 | 3.54E-06 |
| SRD5A2 | 0.568627 | 6.676971 | 3.553638 | 0.001192 | 0.001688 |
| HAMP | 7.509804 | 88.36476 | 3.556624 | 0.000486 | 0.000721 |
| SSTR5 | 4.921569 | 57.9459 | 3.557517 | 2.89E-09 | 7.23E-09 |
| RP11-332E4.1 | 1.039216 | 12.2813 | 3.562896 | 1.86E-12 | 6.25E-12 |
| RP11-546K22.1 | 1.54902 | 18.38794 | 3.569333 | 4.78E-16 | 2.24E-15 |
| RP11-436K8.1 | 2.647059 | 31.5085 | 3.573279 | 3.88E-15 | 1.67E-14 |
| RIPPLY3 | 5.196078 | 62.03709 | 3.577636 | 1.39E-12 | 4.72E-12 |
| CYP2W1 | 201.8824 | 2427.015 | 3.587597 | 7.68E-08 | 1.68E-07 |
| CTD-2184D3.1 | 1.490196 | 17.95054 | 3.590453 | 1.29E-09 | 3.34E-09 |
| RPS18P1 | 0.294118 | 3.550232 | 3.593448 | 2.55E-10 | 7.03E-10 |
| snoU2-30 | 0.137255 | 1.663833 | 3.599581 | 4.18E-06 | 7.71E-06 |
| VWA2 | 77.84314 | 944.6955 | 3.601208 | 4.68E-26 | 8.34E-25 |
| LINC00524 | 0.352941 | 4.292117 | 3.60419 | 1.92E-06 | 3.66E-06 |
| GRHL3 | 17.7451 | 215.9691 | 3.605332 | 3.28E-25 | 4.99E-24 |
| RP5-908M14.5 | 3.745098 | 45.58114 | 3.605362 | 1.33E-20 | 1.02E-19 |
| SLCO1A2 | 1.333333 | 16.23957 | 3.606404 | 0.000764 | 0.001107 |
| RP11-404O13.1 | 0.117647 | 1.437403 | 3.610928 | 2.82E-11 | 8.48E-11 |
| AC144450.2 | 0.784314 | 9.587326 | 3.611626 | 6.33E-08 | 1.40E-07 |
| RP1-27K12.2 | 2.098039 | 25.67543 | 3.613275 | 0.00897 | 0.011384 |
| RP11-211G23.2 | 3.039216 | 37.20247 | 3.613628 | 4.54E-16 | 2.14E-15 |
| DNAH17-AS1 | 0.764706 | 9.377125 | 3.616169 | 3.84E-15 | 1.65E-14 |
| DUXAP8 | 3.323529 | 40.86399 | 3.620042 | 6.01E-13 | 2.10E-12 |
| NPC1L1 | 20.4902 | 252.1932 | 3.621524 | 2.48E-05 | 4.24E-05 |
| AKR1C8P | 0.372549 | 4.585781 | 3.621665 | 2.89E-08 | 6.59E-08 |
| IL36RN | 0.666667 | 8.211747 | 3.622652 | 9.83E-09 | 2.35E-08 |
| AL589743.1 | 1.352941 | 16.71252 | 3.626758 | 1.51E-08 | 3.53E-08 |
| SPP1 | 535 | 6618.332 | 3.628857 | 1.84E-11 | 5.65E-11 |
| TLX1 | 11.41176 | 141.762 | 3.634877 | 3.39E-23 | 3.67E-22 |
| TPRXL | 4.215686 | 52.39876 | 3.635693 | 2.94E-07 | 6.06E-07 |
| DIRC1 | 0.254902 | 3.16847 | 3.635772 | 1.96E-09 | 4.99E-09 |
| H19 | 174.5098 | 2181.382 | 3.643862 | 1.86E-07 | 3.93E-07 |
| CYCSP6 | 2.54902 | 31.95827 | 3.648175 | 3.22E-05 | 5.44E-05 |
| RP5-1158E12.3 | 0.882353 | 11.11283 | 3.654726 | 1.51E-21 | 1.30E-20 |
| MCIDAS | 3.352941 | 42.40185 | 3.660628 | 3.99E-25 | 5.98E-24 |
| DMRT3 | 0.764706 | 9.724884 | 3.668704 | 1.48E-09 | 3.80E-09 |
| AC016735.2 | 30.09804 | 383.3879 | 3.671064 | 0.000212 | 0.000327 |
| ASCL5 | 4.921569 | 62.72952 | 3.671954 | 3.08E-22 | 2.91E-21 |
| SERPINB2 | 3.294118 | 42.034 | 3.673593 | 0.000953 | 0.001364 |
| ULBP1 | 3.039216 | 38.90263 | 3.678097 | 1.17E-22 | 1.17E-21 |
| SLC4A11 | 26.39216 | 338.4204 | 3.680635 | 1.16E-24 | 1.60E-23 |
| RP11-390N6.1 | 1.27451 | 16.41113 | 3.68666 | 4.61E-12 | 1.49E-11 |
| AC105009.1 | 0.392157 | 5.106646 | 3.702873 | 1.74E-07 | 3.68E-07 |
| RPL12P47 | 0.686275 | 8.953632 | 3.705615 | 6.47E-08 | 1.43E-07 |
| PRR36 | 28.09804 | 366.7419 | 3.706224 | 3.15E-22 | 2.98E-21 |
| ISM2 | 10.19608 | 133.2674 | 3.708237 | 9.93E-06 | 1.77E-05 |
| PKP1 | 40.64706 | 531.7079 | 3.709411 | 1.24E-18 | 7.63E-18 |
| KCNMB2-AS1 | 2.666667 | 35.11128 | 3.718825 | 9.96E-09 | 2.38E-08 |
| RP11-170M17.1 | 0.490196 | 6.463679 | 3.720925 | 1.15E-14 | 4.73E-14 |
| SMKR1 | 6.039216 | 79.66151 | 3.72145 | 1.53E-23 | 1.75E-22 |
| MMP20 | 0.705882 | 9.338485 | 3.725689 | 5.80E-11 | 1.69E-10 |
| IGF2-AS | 1.196078 | 15.90108 | 3.732741 | 0.000857 | 0.001234 |
| RP11-615I2.3 | 0.117647 | 1.565688 | 3.734259 | 4.54E-08 | 1.02E-07 |
| RP11-1143G9.5 | 8.588235 | 115.2504 | 3.746266 | 9.75E-16 | 4.44E-15 |
| DDX3P1 | 1.294118 | 17.39104 | 3.748303 | 7.87E-09 | 1.89E-08 |
| SERPIND1 | 7.215686 | 97.12056 | 3.750568 | 3.73E-06 | 6.92E-06 |
| UPK1A | 1.372549 | 18.48532 | 3.75145 | 9.65E-06 | 1.72E-05 |
| RP5-1011O1.2 | 1.117647 | 15.07419 | 3.753544 | 3.37E-13 | 1.21E-12 |
| TNNT1 | 11.82353 | 160.0077 | 3.758409 | 7.39E-05 | 0.00012 |
| IRX5 | 3.901961 | 53.10819 | 3.766663 | 9.61E-21 | 7.48E-20 |
| C2CD4A | 68.2549 | 933.5889 | 3.773783 | 4.30E-26 | 7.72E-25 |
| ABCA9-AS1 | 0.686275 | 9.435858 | 3.781296 | 6.23E-07 | 1.24E-06 |
| SNORA71C | 2.54902 | 35.10201 | 3.783539 | 5.82E-17 | 2.99E-16 |
| LINC01594 | 1.254902 | 17.43277 | 3.796155 | 6.91E-17 | 3.53E-16 |
| GJB5 | 10.90196 | 151.5085 | 3.796739 | 1.64E-06 | 3.14E-06 |
| RNA5SP18 | 0.176471 | 2.454405 | 3.797874 | 6.17E-09 | 1.50E-08 |
| RP11-776H12.1 | 6.27451 | 87.53787 | 3.802333 | 1.14E-15 | 5.18E-15 |
| MLK7-AS1 | 9.333333 | 130.221 | 3.802426 | 1.25E-11 | 3.90E-11 |
| LINC01429 | 0.235294 | 3.285935 | 3.803767 | 2.88E-08 | 6.58E-08 |
| PRDM13 | 1.254902 | 17.59815 | 3.809777 | 4.16E-07 | 8.46E-07 |
| ULBP2 | 5.588235 | 78.72179 | 3.816298 | 2.75E-25 | 4.25E-24 |
| PRSS41 | 1.019608 | 14.37094 | 3.817068 | 1.42E-13 | 5.28E-13 |
| RP11-416L21.2 | 0.215686 | 3.041731 | 3.817886 | 1.66E-07 | 3.51E-07 |
| RP11-3L10.3 | 3.27451 | 46.23493 | 3.819632 | 1.19E-10 | 3.37E-10 |
| KRTAP5-4 | 0.372549 | 5.261206 | 3.819891 | 3.72E-15 | 1.60E-14 |
| CST6 | 4.745098 | 67.55951 | 3.831649 | 0.000137 | 0.000217 |
| RP11-23P13.6 | 6.647059 | 94.66461 | 3.832037 | 1.15E-14 | 4.74E-14 |
| LINC00698 | 0.372549 | 5.327666 | 3.838002 | 5.22E-15 | 2.22E-14 |
| NCAPGP1 | 0.333333 | 4.775889 | 3.840732 | 2.01E-13 | 7.35E-13 |
| RP5-1120P11.3 | 0.27451 | 3.933539 | 3.840898 | 9.53E-12 | 2.99E-11 |
| DPCR1 | 1.470588 | 21.08192 | 3.841541 | 0.022926 | 0.027639 |
| IGF2BP3 | 13.92157 | 199.66 | 3.842151 | 0.013466 | 0.016719 |
| AC079612.1 | 0.137255 | 1.970634 | 3.84373 | 4.05E-11 | 1.20E-10 |
| RP11-161I6.2 | 0.686275 | 9.85626 | 3.844183 | 2.22E-17 | 1.19E-16 |
| RP11-114H23.2 | 0.333333 | 4.820711 | 3.854208 | 6.71E-11 | 1.95E-10 |
| DUSP9 | 2.156863 | 31.2473 | 3.856725 | 5.43E-15 | 2.30E-14 |
| TMEM40 | 0.823529 | 11.9459 | 3.858552 | 2.55E-12 | 8.44E-12 |
| NKD1 | 287.8431 | 4185.045 | 3.861888 | 3.38E-17 | 1.77E-16 |
| ADAM12 | 70.56863 | 1029.096 | 3.866207 | 1.10E-20 | 8.51E-20 |
| CYP4F23P | 1.392157 | 20.37094 | 3.871119 | 1.12E-13 | 4.20E-13 |
| ZDHHC4P1 | 0.509804 | 7.472952 | 3.873664 | 6.02E-05 | 9.87E-05 |
| DLX6 | 3.137255 | 46.20556 | 3.880492 | 2.84E-05 | 4.82E-05 |
| AC093732.1 | 0.72549 | 10.69243 | 3.881489 | 1.09E-14 | 4.50E-14 |
| ATP6V0A4 | 0.843137 | 12.48068 | 3.887785 | 7.56E-11 | 2.18E-10 |
| SLC13A3 | 25 | 371.0742 | 3.891708 | 1.93E-09 | 4.92E-09 |
| SNORA80B | 1.078431 | 16.03709 | 3.894406 | 6.73E-07 | 1.34E-06 |
| NKD2 | 66.01961 | 990.9706 | 3.907876 | 1.68E-24 | 2.26E-23 |
| WISP3 | 6.72549 | 101.306 | 3.912937 | 2.44E-06 | 4.62E-06 |
| CTD-2529O21.1 | 0.470588 | 7.123648 | 3.920079 | 1.96E-21 | 1.66E-20 |
| UCA1 | 43.66667 | 668.0039 | 3.935252 | 1.01E-13 | 3.80E-13 |
| TG | 28.80392 | 441.0386 | 3.936568 | 5.72E-21 | 4.58E-20 |
| AC140076.1 | 0.509804 | 7.828439 | 3.94071 | 9.42E-09 | 2.25E-08 |
| RP11-703I16.3 | 0.196078 | 3.01391 | 3.942134 | 1.46E-08 | 3.42E-08 |
| MAPK15 | 18.72549 | 288.6136 | 3.946064 | 8.86E-27 | 1.83E-25 |
| RPL37AP8 | 1.647059 | 25.45131 | 3.949776 | 3.06E-09 | 7.65E-09 |
| RP11-310P5.1 | 0.352941 | 5.459042 | 3.951148 | 2.85E-16 | 1.37E-15 |
| CTA-315H11.2 | 2.098039 | 33.02318 | 3.976366 | 2.30E-25 | 3.61E-24 |
| BEST3 | 1.509804 | 23.86399 | 3.982402 | 2.87E-09 | 7.19E-09 |
| IFNE | 0.313725 | 5.003091 | 3.995245 | 1.20E-07 | 2.57E-07 |
| RP11-30O15.1 | 0.176471 | 2.819165 | 3.997768 | 7.15E-13 | 2.49E-12 |
| RP1-140K8.5 | 2.137255 | 34.1592 | 3.998443 | 7.22E-17 | 3.67E-16 |
| RP11-419J16.1 | 0.647059 | 10.35085 | 3.999709 | 2.38E-10 | 6.58E-10 |
| AC004593.3 | 1.254902 | 20.08655 | 4.000583 | 4.93E-14 | 1.91E-13 |
| RP11-416I2.1 | 0.745098 | 11.94745 | 4.003129 | 7.50E-21 | 5.92E-20 |
| DLX6-AS1 | 4.352941 | 69.98454 | 4.006974 | 0.000481 | 0.000713 |
| SLC35D3 | 17.39216 | 279.8377 | 4.008082 | 2.82E-12 | 9.29E-12 |
| PIWIL1 | 16.41176 | 265.4714 | 4.015754 | 1.55E-14 | 6.30E-14 |
| RP11-874J12.4 | 0.666667 | 10.78825 | 4.016352 | 1.78E-13 | 6.54E-13 |
| CTD-2015G9.2 | 0.588235 | 9.567233 | 4.023637 | 2.48E-07 | 5.16E-07 |
| CCAT1 | 30.98039 | 504.6692 | 4.025911 | 4.69E-28 | 1.31E-26 |
| PLA2G3 | 5.54902 | 90.42658 | 4.026442 | 5.56E-08 | 1.24E-07 |
| DSG3 | 36.13725 | 590.1592 | 4.029545 | 4.68E-14 | 1.82E-13 |
| FOLR1 | 16.41176 | 268.575 | 4.032523 | 2.32E-07 | 4.84E-07 |
| CASC19 | 3.313725 | 54.24575 | 4.032984 | 9.31E-27 | 1.91E-25 |
| GYLTL1B | 100 | 1651.994 | 4.046136 | 8.40E-28 | 2.23E-26 |
| ACSL6 | 61.86275 | 1039.941 | 4.071287 | 3.22E-11 | 9.61E-11 |
| LINC00592 | 0.098039 | 1.650696 | 4.073571 | 1.85E-10 | 5.16E-10 |
| RP11-10A14.5 | 0.686275 | 11.57187 | 4.075692 | 1.00E-15 | 4.55E-15 |
| RP11-353N14.1 | 0.529412 | 8.928903 | 4.076021 | 3.16E-18 | 1.86E-17 |
| GAST | 0.235294 | 3.970634 | 4.076832 | 1.30E-08 | 3.06E-08 |
| LINC00941 | 2.470588 | 41.74652 | 4.07873 | 4.42E-14 | 1.72E-13 |
| RP11-89K21.1 | 3.196078 | 54.12983 | 4.082049 | 1.75E-08 | 4.07E-08 |
| XXbac-BPG32J3.20 | 0.235294 | 3.996909 | 4.086347 | 4.92E-12 | 1.59E-11 |
| RP13-204A15.5 | 0.352941 | 6.043277 | 4.097831 | 3.69E-08 | 8.35E-08 |
| MMP11 | 283.9216 | 4878.532 | 4.102883 | 3.68E-28 | 1.06E-26 |
| RP11-635N19.3 | 0.196078 | 3.394127 | 4.113538 | 1.65E-08 | 3.85E-08 |
| RP11-417E7.2 | 0.490196 | 8.486862 | 4.1138 | 3.00E-09 | 7.50E-09 |
| EPHX4 | 17.56863 | 305.473 | 4.119971 | 3.17E-29 | 1.21E-27 |
| RP11-350J20.12 | 3.333333 | 58.35085 | 4.129716 | 4.21E-24 | 5.30E-23 |
| PRSS1 | 3.921569 | 68.74498 | 4.131752 | 0.010758 | 0.013505 |
| SIM2 | 34.80392 | 613.1329 | 4.138878 | 2.71E-27 | 6.20E-26 |
| RP11-578B16.1 | 0.921569 | 16.25811 | 4.140925 | 1.02E-12 | 3.52E-12 |
| TESC | 119.451 | 2108.314 | 4.141599 | 2.79E-25 | 4.30E-24 |
| CXCL5 | 78.45098 | 1397.872 | 4.155297 | 5.16E-15 | 2.19E-14 |
| DSC3 | 36.11765 | 644.0355 | 4.156365 | 2.73E-05 | 4.65E-05 |
| RP4-784A16.3 | 0.078431 | 1.398764 | 4.156577 | 7.97E-09 | 1.92E-08 |
| DDN | 7.588235 | 137.2087 | 4.176463 | 6.47E-29 | 2.27E-27 |
| S100A2 | 28.11765 | 509.1252 | 4.178473 | 5.87E-26 | 1.03E-24 |
| MT-TM | 3.254902 | 59.06801 | 4.181691 | 3.35E-09 | 8.33E-09 |
| UCN2 | 1.235294 | 22.47913 | 4.18566 | 1.28E-26 | 2.55E-25 |
| RNU5F-1 | 0.352941 | 6.48068 | 4.198646 | 0.000296 | 0.00045 |
| MMP1 | 246.6078 | 4535.281 | 4.200901 | 2.97E-21 | 2.46E-20 |
| SERPINB7 | 2.333333 | 43.14838 | 4.208842 | 7.82E-11 | 2.25E-10 |
| IL11 | 9.960784 | 185.6692 | 4.220332 | 9.78E-25 | 1.37E-23 |
| IFITM5 | 0.215686 | 4.021638 | 4.220777 | 6.66E-10 | 1.76E-09 |
| AMBP | 21.4902 | 401.8238 | 4.224812 | 0.000755 | 0.001095 |
| FAM195CP | 0.607843 | 11.43431 | 4.233527 | 2.94E-18 | 1.73E-17 |
| EVX1-AS | 1.313725 | 24.74498 | 4.2354 | 1.03E-10 | 2.93E-10 |
| TCF24 | 0.568627 | 10.76043 | 4.242109 | 4.84E-17 | 2.50E-16 |
| SLC22A11 | 6.117647 | 115.9691 | 4.24462 | 5.75E-14 | 2.21E-13 |
| CAMKV | 3.294118 | 62.75734 | 4.25182 | 6.05E-10 | 1.61E-09 |
| LINC01296 | 0.686275 | 13.08192 | 4.252644 | 8.67E-12 | 2.73E-11 |
| RP5-1052M9.1 | 1.745098 | 33.28439 | 4.253466 | 6.78E-08 | 1.49E-07 |
| RP11-429J17.5 | 0.470588 | 9.012365 | 4.259369 | 5.68E-21 | 4.55E-20 |
| CTD-2147F2.2 | 0.45098 | 8.639876 | 4.259874 | 3.34E-09 | 8.30E-09 |
| AC012363.4 | 0.392157 | 7.562597 | 4.269379 | 3.14E-13 | 1.13E-12 |
| C6orf223 | 81.03922 | 1565.921 | 4.272248 | 8.09E-30 | 3.77E-28 |
| AF127577.8 | 0.764706 | 14.83617 | 4.27807 | 1.26E-08 | 2.97E-08 |
| RP5-1120P11.1 | 4.392157 | 85.55023 | 4.28377 | 2.82E-29 | 1.09E-27 |
| RP11-338N10.3 | 0.45098 | 8.833076 | 4.291779 | 6.53E-16 | 3.03E-15 |
| OFCC1 | 0.313725 | 6.20711 | 4.306347 | 1.32E-06 | 2.55E-06 |
| CNTD2 | 8.921569 | 176.5873 | 4.306941 | 6.21E-29 | 2.19E-27 |
| SCARNA13 | 6.823529 | 135.0711 | 4.307057 | 0.000107 | 0.000171 |
| MSX2 | 27.03922 | 535.6151 | 4.308072 | 2.99E-22 | 2.83E-21 |
| PRSS2 | 79 | 1571.331 | 4.31399 | 1.30E-08 | 3.07E-08 |
| RP11-141J13.5 | 0.72549 | 14.44822 | 4.315792 | 4.02E-15 | 1.72E-14 |
| SP8 | 2.196078 | 43.74343 | 4.316065 | 4.21E-18 | 2.43E-17 |
| GMCL1P2 | 0.137255 | 2.734158 | 4.316167 | 1.35E-09 | 3.49E-09 |
| SNORD15B | 2.27451 | 45.58733 | 4.325005 | 1.29E-10 | 3.65E-10 |
| PRSS33 | 16.80392 | 336.8501 | 4.325237 | 3.65E-13 | 1.30E-12 |
| UPK2 | 1.294118 | 26.21175 | 4.340173 | 1.03E-07 | 2.23E-07 |
| DUSP5P1 | 0.254902 | 5.187017 | 4.346891 | 2.28E-14 | 9.10E-14 |
| PDX1 | 36.05882 | 744.8887 | 4.368601 | 1.67E-28 | 5.21E-27 |
| CRAT40 | 0.54902 | 11.49923 | 4.388535 | 7.23E-09 | 1.74E-08 |
| LINC01021 | 2.156863 | 45.32612 | 4.393336 | 0.000841 | 0.001211 |
| CRNDE | 8.980392 | 188.7527 | 4.393575 | 2.00E-27 | 4.74E-26 |
| DHRS2 | 11.43137 | 240.4884 | 4.394897 | 2.45E-20 | 1.82E-19 |
| CALCA | 4.235294 | 89.99845 | 4.409366 | 6.95E-07 | 1.38E-06 |
| RP11-56A10.1 | 0.156863 | 3.352396 | 4.417618 | 3.54E-08 | 8.02E-08 |
| MYO16-AS1 | 0.196078 | 4.202473 | 4.421736 | 7.70E-07 | 1.52E-06 |
| LPO | 1.588235 | 34.58733 | 4.444747 | 6.13E-18 | 3.48E-17 |
| RP11-431J24.2 | 8.098039 | 176.7264 | 4.447801 | 0.001218 | 0.001723 |
| SOX1 | 4.078431 | 89.0541 | 4.448596 | 0.010843 | 0.013609 |
| PGLYRP3 | 0.235294 | 5.261206 | 4.482856 | 8.84E-07 | 1.74E-06 |
| SEC14L4 | 1.294118 | 29.40495 | 4.506018 | 6.08E-09 | 1.48E-08 |
| AC133785.1 | 0.27451 | 6.242658 | 4.507231 | 7.78E-13 | 2.70E-12 |
| CCAT2 | 0.45098 | 10.28439 | 4.511248 | 4.95E-11 | 1.45E-10 |
| BBOX1-AS1 | 4.764706 | 109.2241 | 4.51876 | 3.38E-17 | 1.78E-16 |
| ELFN1-AS1 | 15.07843 | 347.0742 | 4.524686 | 7.02E-28 | 1.89E-26 |
| MMP3 | 85.52941 | 1974.915 | 4.529226 | 3.74E-23 | 4.03E-22 |
| POU5F1B | 16.58824 | 383.4297 | 4.53073 | 7.31E-22 | 6.55E-21 |
| DMRTA2 | 2.647059 | 61.21175 | 4.531346 | 1.59E-05 | 2.78E-05 |
| PGC | 3.980392 | 92.62751 | 4.540458 | 7.49E-16 | 3.45E-15 |
| AC105399.2 | 0.470588 | 11.1762 | 4.56982 | 3.90E-17 | 2.03E-16 |
| GRIN2D | 62.39216 | 1484.598 | 4.572564 | 2.29E-30 | 1.35E-28 |
| CLDN1 | 176.9804 | 4217.182 | 4.574618 | 7.62E-31 | 5.86E-29 |
| TNNI3 | 2.980392 | 71.55023 | 4.585382 | 8.04E-18 | 4.50E-17 |
| RP11-378I6.1 | 0.137255 | 3.2983 | 4.586793 | 1.78E-08 | 4.14E-08 |
| CPB2 | 1.333333 | 32.19629 | 4.593785 | 6.49E-05 | 0.000106 |
| IGFL1P1 | 0.294118 | 7.183926 | 4.610307 | 1.36E-08 | 3.19E-08 |
| TACSTD2 | 74.19608 | 1812.801 | 4.610734 | 2.81E-20 | 2.08E-19 |
| TMEM75 | 0.764706 | 18.69397 | 4.611524 | 1.05E-11 | 3.28E-11 |
| NCAPD2P1 | 0.843137 | 21.16538 | 4.649795 | 7.78E-19 | 4.90E-18 |
| SALL4 | 6.352941 | 160.813 | 4.661815 | 1.87E-29 | 7.73E-28 |
| WT1 | 3.529412 | 89.36167 | 4.662156 | 5.09E-15 | 2.16E-14 |
| RP11-60L3.1 | 0.764706 | 19.39413 | 4.664571 | 3.15E-17 | 1.66E-16 |
| HIST1H4E | 7.117647 | 180.9274 | 4.667866 | 0.002051 | 0.002824 |
| AC010970.2 | 2.960784 | 75.56878 | 4.673739 | 0.016373 | 0.020116 |
| KLK10 | 75.62745 | 1932.978 | 4.675772 | 7.87E-21 | 6.20E-20 |
| SPATA20P1 | 0.215686 | 5.531685 | 4.680713 | 5.78E-11 | 1.69E-10 |
| AL162759.1 | 0.176471 | 4.534776 | 4.683532 | 1.33E-11 | 4.12E-11 |
| HIST1H2BO | 2.607843 | 67.49459 | 4.693843 | 0.011058 | 0.013864 |
| CST5 | 0.215686 | 5.672334 | 4.716936 | 6.80E-09 | 1.65E-08 |
| CHST4 | 5.176471 | 136.2813 | 4.718475 | 1.37E-06 | 2.64E-06 |
| ARNTL2-AS1 | 0.078431 | 2.072643 | 4.723897 | 3.69E-09 | 9.12E-09 |
| RP11-114H23.1 | 0.45098 | 11.94436 | 4.727121 | 5.09E-20 | 3.63E-19 |
| AC007128.1 | 1.568627 | 41.60433 | 4.729159 | 2.55E-25 | 3.97E-24 |
| RP11-547D24.1 | 0.490196 | 13.07883 | 4.73773 | 2.06E-26 | 3.93E-25 |
| PLAC4 | 5.686275 | 152.238 | 4.742701 | 1.53E-20 | 1.16E-19 |
| PADI3 | 5.941176 | 160.357 | 4.754395 | 2.40E-05 | 4.11E-05 |
| RN7SL648P | 0.588235 | 15.99227 | 4.764838 | 0.01608 | 0.019782 |
| LY6D | 2.705882 | 74.02937 | 4.773927 | 1.71E-09 | 4.36E-09 |
| ARHGAP40 | 0.54902 | 15.08192 | 4.779818 | 1.54E-12 | 5.22E-12 |
| TRIM72 | 5.686275 | 161.1484 | 4.824762 | 7.89E-06 | 1.42E-05 |
| IGF2BP1 | 8.568627 | 246.5471 | 4.846656 | 0.004795 | 0.006297 |
| HIST1H2AL | 1.156863 | 33.51314 | 4.856437 | 2.85E-05 | 4.84E-05 |
| AC012512.1 | 0.45098 | 13.11128 | 4.8616 | 4.22E-12 | 1.37E-11 |
| GAD1 | 4.490196 | 130.5549 | 4.861734 | 3.39E-24 | 4.32E-23 |
| AL773572.7 | 0.411765 | 12.14219 | 4.882065 | 1.67E-11 | 5.15E-11 |
| BAAT | 2.254902 | 67.2643 | 4.898704 | 9.34E-15 | 3.87E-14 |
| C2orf61 | 1.960784 | 59.03864 | 4.912157 | 4.86E-22 | 4.47E-21 |
| EPHA8 | 0.490196 | 14.81607 | 4.91766 | 3.52E-05 | 5.91E-05 |
| CEMIP | 202.4902 | 6133.529 | 4.920793 | 2.10E-31 | 2.06E-29 |
| ETV4 | 153.7451 | 4687.42 | 4.930182 | 3.58E-32 | 5.38E-30 |
| RNU6ATAC | 0.627451 | 19.15456 | 4.932041 | 0.029803 | 0.035473 |
| NXPH4 | 7.882353 | 244.7403 | 4.956482 | 3.54E-18 | 2.06E-17 |
| APOA2 | 9.568627 | 297.2813 | 4.957373 | 1.34E-08 | 3.14E-08 |
| RP11-53M11.3 | 0.352941 | 10.99536 | 4.961324 | 2.25E-18 | 1.34E-17 |
| CRAT37 | 0.686275 | 21.56569 | 4.973808 | 9.58E-10 | 2.50E-09 |
| AC007277.3 | 1.823529 | 57.66151 | 4.982803 | 1.10E-14 | 4.53E-14 |
| CXCL17 | 2.117647 | 68.15147 | 5.008211 | 1.14E-10 | 3.24E-10 |
| CNN2P4 | 0.196078 | 6.37558 | 5.023054 | 2.29E-07 | 4.78E-07 |
| AMELX | 0.411765 | 13.5255 | 5.037718 | 3.92E-19 | 2.53E-18 |
| INHBA | 55.13725 | 1815.046 | 5.040835 | 1.35E-30 | 9.17E-29 |
| BLACAT1 | 7.039216 | 233.3694 | 5.051057 | 1.88E-29 | 7.75E-28 |
| KRT17 | 38.84314 | 1288.522 | 5.051914 | 1.20E-23 | 1.40E-22 |
| LINC01050 | 0.196078 | 6.537867 | 5.059317 | 4.71E-16 | 2.21E-15 |
| CA9 | 55.92157 | 1873.114 | 5.06589 | 6.45E-21 | 5.14E-20 |
| HIST1H3J | 1.411765 | 47.39258 | 5.06909 | 9.01E-08 | 1.96E-07 |
| FOXI3 | 0.156863 | 5.276662 | 5.072051 | 5.73E-08 | 1.27E-07 |
| RP11-332K15.1 | 0.058824 | 1.989181 | 5.079637 | 1.09E-12 | 3.74E-12 |
| SNORD17 | 11.03922 | 383.9181 | 5.120089 | 5.59E-11 | 1.63E-10 |
| LINC01614 | 0.705882 | 24.56878 | 5.121255 | 3.50E-17 | 1.83E-16 |
| PCAT2 | 0.137255 | 4.829985 | 5.137089 | 1.81E-15 | 8.06E-15 |
| NXPH1 | 0.137255 | 4.851623 | 5.143538 | 3.06E-12 | 1.01E-11 |
| CLDN2 | 186.8235 | 6622.637 | 5.147658 | 5.83E-25 | 8.48E-24 |
| HIST1H2AJ | 1.72549 | 61.39876 | 5.153131 | 0.012181 | 0.015194 |
| AFAP1-AS1 | 10.58824 | 379.0603 | 5.161893 | 0.011059 | 0.013865 |
| WNT2 | 10.52941 | 377.83 | 5.16524 | 6.74E-32 | 8.54E-30 |
| PDX1-AS1 | 0.568627 | 20.67388 | 5.184182 | 7.51E-25 | 1.07E-23 |
| RP11-460N11.2 | 0.431373 | 15.83771 | 5.198286 | 0.000317 | 0.00048 |
| SAA4 | 0.470588 | 17.41422 | 5.209657 | 1.97E-07 | 4.14E-07 |
| RP4-594A5.1 | 0.313725 | 11.76507 | 5.228863 | 1.71E-22 | 1.67E-21 |
| KRT6B | 17.78431 | 674.017 | 5.244108 | 9.77E-22 | 8.59E-21 |
| RP11-742B18.1 | 0.156863 | 6.049459 | 5.269231 | 1.54E-08 | 3.60E-08 |
| AKAP4 | 0.254902 | 9.924266 | 5.282946 | 4.21E-10 | 1.14E-09 |
| RNU6-403P | 0.078431 | 3.063369 | 5.287545 | 2.97E-11 | 8.91E-11 |
| LY6G6D | 11.4902 | 449.9042 | 5.291142 | 1.30E-12 | 4.43E-12 |
| LINC01511 | 0.294118 | 11.52859 | 5.292679 | 3.29E-14 | 1.29E-13 |
| RP11-126O1.4 | 0.235294 | 9.239567 | 5.295288 | 9.10E-09 | 2.18E-08 |
| RP11-454P21.1 | 0.156863 | 6.205564 | 5.305988 | 9.95E-15 | 4.11E-14 |
| RP1-170O19.17 | 0.529412 | 20.9459 | 5.306134 | 1.91E-12 | 6.38E-12 |
| OTX1 | 2.960784 | 117.4961 | 5.31049 | 2.16E-28 | 6.55E-27 |
| AC012501.3 | 0.941176 | 37.4745 | 5.3153 | 1.50E-10 | 4.23E-10 |
| snoU2_19 | 0.034314 | 1.37442 | 5.323894 | 3.31E-10 | 9.00E-10 |
| NPSR1 | 5.529412 | 223.1654 | 5.334843 | 0.000854 | 0.00123 |
| CPNE7 | 29.82353 | 1218.008 | 5.351928 | 3.22E-31 | 2.80E-29 |
| ESM1 | 7.647059 | 319.1345 | 5.383116 | 2.90E-32 | 4.56E-30 |
| RP11-510M2.5 | 0.254902 | 10.64142 | 5.383605 | 1.31E-07 | 2.80E-07 |
| MAGEB17 | 3.078431 | 129.0881 | 5.390017 | 2.19E-06 | 4.15E-06 |
| MMP8 | 1.019608 | 42.84389 | 5.393003 | 2.80E-13 | 1.01E-12 |
| S100A7 | 0.470588 | 19.82844 | 5.396962 | 2.43E-05 | 4.15E-05 |
| CDH3 | 88.98039 | 3767.142 | 5.403839 | 2.71E-32 | 4.29E-30 |
| MYBPHL | 0.588235 | 24.92427 | 5.405014 | 1.85E-11 | 5.65E-11 |
| STRA6 | 12.80392 | 547.323 | 5.417735 | 4.89E-29 | 1.78E-27 |
| IGFL4 | 2.411765 | 104.1144 | 5.431936 | 1.09E-27 | 2.79E-26 |
| AQP5 | 5.254902 | 230.7187 | 5.456327 | 5.40E-07 | 1.09E-06 |
| RP11-115D19.1 | 1.882353 | 82.74652 | 5.45809 | 0.000194 | 0.000301 |
| RP11-54H7.4 | 9.823529 | 433.3354 | 5.463099 | 1.11E-19 | 7.60E-19 |
| RP11-817J15.2 | 0.45098 | 20.03709 | 5.473465 | 1.17E-16 | 5.81E-16 |
| XXbac-BPG32J3.19 | 9.647059 | 428.7774 | 5.473996 | 8.17E-14 | 3.10E-13 |
| WNT7B | 1.607843 | 71.83771 | 5.481543 | 3.36E-25 | 5.09E-24 |
| BHLHA9 | 0.176471 | 7.964451 | 5.496075 | 3.97E-09 | 9.78E-09 |
| CTD-2147F2.1 | 2.117647 | 96.38794 | 5.508319 | 1.36E-19 | 9.25E-19 |
| PAH | 4.156863 | 189.5626 | 5.511035 | 1.44E-14 | 5.84E-14 |
| RP1 | 0.215686 | 9.896445 | 5.519904 | 1.34E-15 | 6.03E-15 |
| USP26 | 0.058824 | 2.710974 | 5.526274 | 2.74E-10 | 7.50E-10 |
| HIST2H2AB | 0.666667 | 30.79753 | 5.529705 | 0.001141 | 0.001619 |
| KLC3 | 0.882353 | 40.89954 | 5.534585 | 2.92E-28 | 8.62E-27 |
| LINC01593 | 0.098039 | 4.565688 | 5.541329 | 7.66E-13 | 2.66E-12 |
| MMP13 | 2.156863 | 100.6754 | 5.544633 | 5.71E-22 | 5.21E-21 |
| SNORA74 | 0.095588 | 4.508308 | 5.559609 | 1.96E-08 | 4.55E-08 |
| PCAT14 | 2.352941 | 111.3308 | 5.564243 | 0.004821 | 0.00633 |
| F2 | 2.54902 | 120.745 | 5.565877 | 3.13E-11 | 9.37E-11 |
| TRIM71 | 0.745098 | 35.76971 | 5.585164 | 2.79E-08 | 6.38E-08 |
| AKR1C4 | 1.254902 | 60.39413 | 5.588762 | 2.93E-25 | 4.50E-24 |
| FIRRE | 1.490196 | 72.02318 | 5.594887 | 5.65E-22 | 5.16E-21 |
| AC142293.3 | 0.254902 | 12.37867 | 5.60177 | 5.20E-15 | 2.21E-14 |
| RP11-142A22.4 | 0.078431 | 3.84544 | 5.615574 | 9.97E-11 | 2.85E-10 |
| CLPSL2 | 0.039216 | 1.928903 | 5.620206 | 1.44E-11 | 4.46E-11 |
| CEL | 30.96078 | 1534.733 | 5.631401 | 4.63E-16 | 2.18E-15 |
| SCARNA6 | 1.598039 | 79.59969 | 5.638388 | 0.000559 | 0.000824 |
| LINC01169 | 0.313725 | 15.67697 | 5.643 | 4.17E-11 | 1.23E-10 |
| ZIC2 | 4.980392 | 251.3091 | 5.65706 | 2.46E-11 | 7.46E-11 |
| AC010967.2 | 0.058824 | 2.981453 | 5.663478 | 5.23E-13 | 1.84E-12 |
| TPSP2 | 0.176471 | 8.964451 | 5.666716 | 1.57E-14 | 6.38E-14 |
| AC006262.5 | 0.862745 | 44.13447 | 5.676828 | 5.17E-16 | 2.42E-15 |
| ZNF280A | 0.156863 | 8.089645 | 5.688502 | 7.06E-14 | 2.69E-13 |
| LINC00858 | 1.254902 | 65.17465 | 5.698664 | 1.77E-19 | 1.18E-18 |
| HIST1H4D | 2.392157 | 127.2813 | 5.733565 | 0.005173 | 0.006764 |
| AC123023.1 | 0.784314 | 41.74343 | 5.733975 | 2.65E-24 | 3.43E-23 |
| LEMD1 | 2.803922 | 150.238 | 5.74366 | 7.25E-29 | 2.52E-27 |
| RP11-815M8.1 | 0.196078 | 10.60433 | 5.757078 | 3.55E-18 | 2.07E-17 |
| ORM2 | 2.058824 | 114.3648 | 5.795679 | 1.08E-09 | 2.80E-09 |
| HIST1H3I | 0.568627 | 31.70943 | 5.801284 | 1.88E-05 | 3.25E-05 |
| EN2 | 1.294118 | 72.48995 | 5.80774 | 1.76E-12 | 5.92E-12 |
| LY6G6E | 0.254902 | 14.55796 | 5.835722 | 1.05E-19 | 7.17E-19 |
| SNORA71A | 2.254902 | 130.626 | 5.856233 | 2.59E-19 | 1.70E-18 |
| LINC01101 | 0.156863 | 9.103555 | 5.858855 | 3.03E-19 | 1.97E-18 |
| CST2 | 1.098039 | 63.79598 | 5.860464 | 9.83E-28 | 2.57E-26 |
| KRT16 | 3.019608 | 177.1097 | 5.874139 | 8.62E-19 | 5.39E-18 |
| COMP | 17.21569 | 1014.414 | 5.880779 | 6.15E-22 | 5.58E-21 |
| HIST1H2AH | 0.941176 | 55.72798 | 5.887793 | 8.39E-09 | 2.01E-08 |
| CLDN10-AS1 | 0.215686 | 12.78671 | 5.889567 | 1.15E-14 | 4.72E-14 |
| LINC00659 | 0.921569 | 54.74343 | 5.89245 | 3.30E-29 | 1.25E-27 |
| DMBX1 | 0.607843 | 36.11283 | 5.892669 | 2.40E-23 | 2.66E-22 |
| NEUROG2 | 0.313725 | 18.65842 | 5.894181 | 1.83E-08 | 4.26E-08 |
| RP11-469H8.6 | 0.372549 | 22.30603 | 5.90386 | 8.08E-24 | 9.70E-23 |
| MSLNL | 0.215686 | 13.14219 | 5.929128 | 2.36E-15 | 1.04E-14 |
| GBX2 | 0.235294 | 14.38485 | 5.933941 | 3.34E-21 | 2.75E-20 |
| AC068121.1 | 0.137255 | 8.559505 | 5.962598 | 2.06E-11 | 6.29E-11 |
| C5orf46 | 0.372549 | 23.54714 | 5.981978 | 1.37E-20 | 1.04E-19 |
| CTC-327F10.4 | 0.058824 | 3.748068 | 5.99361 | 1.66E-09 | 4.24E-09 |
| KRT6A | 8.039216 | 512.2535 | 5.993659 | 7.27E-18 | 4.09E-17 |
| FGF20 | 0.666667 | 42.80989 | 6.004835 | 2.85E-07 | 5.90E-07 |
| HIST1H2AB | 0.568627 | 36.72798 | 6.013252 | 7.00E-07 | 1.39E-06 |
| LY6G6F | 0.176471 | 11.52705 | 6.029452 | 7.00E-19 | 4.42E-18 |
| RP1-276N6.2 | 0.509804 | 33.42504 | 6.034843 | 1.43E-20 | 1.09E-19 |
| RP11-254F7.1 | 0.078431 | 5.151468 | 6.037409 | 2.22E-14 | 8.87E-14 |
| IBSP | 0.843137 | 57.05873 | 6.080536 | 3.13E-20 | 2.30E-19 |
| FOXQ1 | 25.7451 | 1755.278 | 6.091258 | 8.32E-31 | 6.22E-29 |
| RP11-663N22.1 | 0.039216 | 2.720247 | 6.116163 | 5.11E-10 | 1.37E-09 |
| KRT6C | 0.235294 | 16.72952 | 6.151787 | 8.26E-08 | 1.80E-07 |
| HIST1H1E | 4.686275 | 343.6244 | 6.196247 | 1.24E-09 | 3.20E-09 |
| CASC21 | 1.156863 | 85.03864 | 6.199829 | 9.81E-27 | 2.01E-25 |
| SLCO1B3 | 2.176471 | 162.6136 | 6.223314 | 2.90E-17 | 1.53E-16 |
| COL11A1 | 24.98039 | 1892.658 | 6.243474 | 6.34E-30 | 3.10E-28 |
| HIST1H4B | 0.901961 | 68.50077 | 6.246912 | 1.51E-07 | 3.20E-07 |
| KRT80 | 21.68627 | 1665.72 | 6.26322 | 1.83E-32 | 3.21E-30 |
| RP11-143E21.2 | 0.176471 | 13.60124 | 6.268166 | 1.72E-15 | 7.66E-15 |
| SCARNA7 | 2.196078 | 171.3895 | 6.286205 | 0.019692 | 0.023955 |
| AHSG | 2.627451 | 209.6491 | 6.318169 | 6.04E-10 | 1.60E-09 |
| RP11-191N8.2 | 0.039216 | 3.140649 | 6.323488 | 6.86E-12 | 2.18E-11 |
| RP5-884M6.1 | 0.882353 | 70.99845 | 6.330288 | 4.46E-30 | 2.32E-28 |
| ONECUT3 | 1.843137 | 149.221 | 6.339143 | 2.54E-06 | 4.80E-06 |
| MUC6 | 8.921569 | 754.2334 | 6.40157 | 1.11E-05 | 1.96E-05 |
| CTD-2034I21.2 | 0.137255 | 11.69861 | 6.413336 | 7.02E-10 | 1.85E-09 |
| SLCO1B1 | 0.196078 | 16.91808 | 6.430991 | 1.87E-09 | 4.77E-09 |
| RP11-167H9.4 | 0.176471 | 15.4034 | 6.447677 | 1.44E-18 | 8.78E-18 |
| NPSR1-AS1 | 0.45098 | 39.36476 | 6.447696 | 2.61E-22 | 2.49E-21 |
| RP11-401P9.7 | 0.529412 | 47.47759 | 6.486713 | 1.54E-18 | 9.33E-18 |
| LINC01411 | 0.941176 | 84.71561 | 6.492019 | 1.02E-18 | 6.35E-18 |
| LINC00460 | 0.823529 | 75.11437 | 6.511125 | 1.28E-28 | 4.09E-27 |
| C14orf105 | 0.235294 | 21.61824 | 6.52164 | 1.92E-13 | 7.03E-13 |
| SLC26A9 | 1.235294 | 118.6136 | 6.585271 | 1.18E-11 | 3.68E-11 |
| AC018359.1 | 0.294118 | 28.64606 | 6.6058 | 7.60E-24 | 9.17E-23 |
| HABP2 | 1.235294 | 121.8748 | 6.624402 | 3.85E-12 | 1.25E-11 |
| MMP7 | 27.11765 | 2707.196 | 6.641424 | 1.65E-29 | 6.93E-28 |
| AC005256.1 | 0.117647 | 11.83153 | 6.652028 | 3.87E-21 | 3.17E-20 |
| HIST1H4C | 1.686275 | 170.9985 | 6.664 | 3.79E-05 | 6.34E-05 |
| HIST1H1B | 3.352941 | 344.2798 | 6.68201 | 1.32E-07 | 2.83E-07 |
| SCARNA10 | 0.803922 | 83.05641 | 6.690893 | 0.005947 | 0.007715 |
| AC079466.1 | 0.235294 | 24.66151 | 6.711652 | 2.49E-10 | 6.85E-10 |
| GOLGA6L2 | 0.058824 | 6.173107 | 6.71346 | 4.06E-10 | 1.10E-09 |
| AC068580.7 | 0.058824 | 6.255023 | 6.732478 | 7.02E-13 | 2.45E-12 |
| CST4 | 0.215686 | 23.0711 | 6.741008 | 6.82E-23 | 7.07E-22 |
| RNU4-1 | 2.137255 | 230.5147 | 6.752956 | 0.025298 | 0.030346 |
| SERPINA4 | 0.627451 | 67.88872 | 6.757525 | 5.05E-25 | 7.44E-24 |
| C3P1 | 0.156863 | 18.04328 | 6.845815 | 6.03E-14 | 2.31E-13 |
| SNORA73B | 10.21569 | 1177.935 | 6.84933 | 2.85E-16 | 1.37E-15 |
| SFTA2 | 1.019608 | 118.4451 | 6.860061 | 9.28E-29 | 3.12E-27 |
| VGLL1 | 0.137255 | 16.44513 | 6.904659 | 6.88E-11 | 1.99E-10 |
| DUSP27 | 16.27451 | 1954.328 | 6.907914 | 1.26E-08 | 2.96E-08 |
| RNU4-2 | 5.509804 | 688.3354 | 6.964967 | 0.004874 | 0.006396 |
| TCN1 | 7.941176 | 997.1777 | 6.972354 | 9.48E-23 | 9.57E-22 |
| HIST1H2BI | 0.490196 | 64.77898 | 7.046023 | 1.15E-05 | 2.04E-05 |
| RP11-474D1.1 | 0.058824 | 7.902628 | 7.069795 | 6.57E-14 | 2.51E-13 |
| SPRR3 | 0.705882 | 96.83308 | 7.099928 | 5.20E-09 | 1.27E-08 |
| ADAM20P3 | 0.058824 | 8.170015 | 7.117802 | 6.27E-17 | 3.21E-16 |
| KRT23 | 22.88235 | 3185.184 | 7.120997 | 3.90E-25 | 5.85E-24 |
| ELF5 | 1.058824 | 147.7852 | 7.124895 | 4.19E-11 | 1.24E-10 |
| RPSAP71 | 0.137255 | 19.93199 | 7.182085 | 5.36E-11 | 1.57E-10 |
| KRT75 | 0.509804 | 74.56569 | 7.192426 | 9.96E-22 | 8.74E-21 |
| RP11-400N13.3 | 0.156863 | 23.7527 | 7.242445 | 2.16E-24 | 2.85E-23 |
| OBP2B | 0.196078 | 30.31994 | 7.272692 | 3.46E-13 | 1.24E-12 |
| EPYC | 0.607843 | 97.69552 | 7.32845 | 1.50E-16 | 7.40E-16 |
| LINC01234 | 1.019608 | 169.8686 | 7.380261 | 9.46E-20 | 6.53E-19 |
| SOX14 | 0.392157 | 67.18083 | 7.420475 | 8.81E-21 | 6.90E-20 |
| SPRR2A | 0.352941 | 65.89799 | 7.544663 | 7.19E-11 | 2.08E-10 |
| AC007099.1 | 0.196078 | 37.32303 | 7.572491 | 3.94E-27 | 8.78E-26 |
| SPERT | 0.176471 | 34.27048 | 7.601395 | 1.16E-26 | 2.34E-25 |
| LINC01602 | 0.098039 | 20.19165 | 7.686184 | 6.67E-16 | 3.09E-15 |
| COL10A1 | 5.705882 | 1205.682 | 7.723183 | 1.89E-28 | 5.85E-27 |
| AP000697.6 | 0.019608 | 4.177743 | 7.735149 | 5.47E-21 | 4.39E-20 |
| FGA | 8.666667 | 1949.938 | 7.813735 | 6.60E-13 | 2.30E-12 |
| SPRR1B | 0.333333 | 76.06801 | 7.83418 | 4.26E-12 | 1.38E-11 |
| HULC | 0.892157 | 208.0711 | 7.865563 | 1.20E-06 | 2.32E-06 |
| CST1 | 4.196078 | 1067.298 | 7.990706 | 1.61E-30 | 1.04E-28 |
| C8orf74 | 0.039216 | 9.979907 | 7.991452 | 8.11E-18 | 4.54E-17 |
| RP11-401P9.1 | 0.058824 | 14.97063 | 7.991526 | 9.66E-10 | 2.52E-09 |
| SCARNA5 | 1.411765 | 362.4946 | 8.004316 | 4.84E-05 | 8.01E-05 |
| SPRR2D | 0.254902 | 67.50541 | 8.048917 | 1.33E-13 | 4.95E-13 |
| RP11-474D1.3 | 2.960784 | 784.1391 | 8.048986 | 1.76E-24 | 2.36E-23 |
| AC064834.1 | 0.882353 | 239.5379 | 8.084682 | 3.83E-20 | 2.77E-19 |
| RP11-297P16.4 | 0.392157 | 106.8655 | 8.09015 | 5.23E-06 | 9.54E-06 |
| SNORA71D | 0.176471 | 48.50077 | 8.102436 | 2.66E-10 | 7.31E-10 |
| RNU5E-1 | 0.254902 | 72.48995 | 8.151695 | 3.75E-07 | 7.66E-07 |
| PRR9 | 0.156863 | 45.02473 | 8.165071 | 3.78E-09 | 9.35E-09 |
| ZIC5 | 0.372549 | 108.6461 | 8.18799 | 8.50E-17 | 4.30E-16 |
| RN7SK | 80.17647 | 23675.64 | 8.206009 | 0.026381 | 0.031591 |
| KLK7 | 0.843137 | 250.1113 | 8.212587 | 1.70E-25 | 2.73E-24 |
| DKK4 | 0.745098 | 221.4946 | 8.215625 | 6.49E-25 | 9.34E-24 |
| C6orf15 | 0.54902 | 174.3818 | 8.311176 | 2.44E-22 | 2.34E-21 |
| SNORA74A | 0.156863 | 50.19629 | 8.321934 | 8.80E-11 | 2.53E-10 |
| FGB | 4.392157 | 1477.167 | 8.393688 | 1.52E-13 | 5.63E-13 |
| NOTUM | 9 | 3148.009 | 8.450299 | 1.29E-28 | 4.11E-27 |
| FEZF1-AS1 | 0.529412 | 186.3215 | 8.459188 | 6.49E-26 | 1.13E-24 |
| PPBP | 6.117647 | 2171.014 | 8.471176 | 3.65E-12 | 1.19E-11 |
| FEZF1 | 0.27451 | 97.44049 | 8.47152 | 3.10E-22 | 2.93E-21 |
| RP11-400N13.2 | 0.098039 | 35.5966 | 8.504165 | 6.79E-13 | 2.37E-12 |
| RP11-474D1.4 | 0.098039 | 36.14838 | 8.526356 | 8.32E-22 | 7.38E-21 |
| SPRR1A | 0.137255 | 53.09428 | 8.595555 | 7.79E-12 | 2.46E-11 |
| AC012501.2 | 0.019608 | 8.409583 | 8.74446 | 7.06E-11 | 2.04E-10 |
| RP11-109M17.2 | 0.039216 | 17.53014 | 8.804191 | 1.92E-12 | 6.42E-12 |
| ORM1 | 1.941176 | 877.2133 | 8.819853 | 2.91E-06 | 5.46E-06 |
| RP11-138J23.1 | 0.058824 | 26.84699 | 8.834151 | 8.53E-26 | 1.44E-24 |
| AC003958.2 | 0.019608 | 9.251932 | 8.88218 | 4.80E-12 | 1.55E-11 |
| PAEP | 0.529412 | 259.9799 | 8.939794 | 3.16E-19 | 2.06E-18 |
| KLK8 | 0.215686 | 116.7743 | 9.080573 | 3.45E-25 | 5.22E-24 |
| KLK6 | 1.960784 | 1160.771 | 9.209437 | 1.71E-30 | 1.09E-28 |
| RP11-143E21.3 | 0.117647 | 87.7357 | 9.542555 | 1.76E-20 | 1.33E-19 |
| PRSS56 | 0.235294 | 218.1607 | 9.856711 | 3.99E-12 | 1.30E-11 |
| IGFL1 | 0.039216 | 44.2473 | 10.13994 | 1.01E-16 | 5.09E-16 |
| RP11-197K6.1 | 0.019608 | 26.15147 | 10.38125 | 5.25E-14 | 2.03E-13 |
| RP11-143E21.6 | 0 | 5.015456 | Inf | 3.13E-16 | 1.50E-15 |
