# Supplementary material for: Identification of Hub Genes Related to Carcinogenesis and Prognosis in Colorectal Cancer Based on Integrated Bioinformatics
Source: Mediators Inflamm. 2020 Apr 9;2020:5934821. doi: 10.1155/2020/5934821 (PMC7171686; doi:10.1155/2020/5934821)
Supplement: Supplementary 3 — Table S3: overlapping DEGs identified between six GEO datasets and TCGA database. [file 5934821.f3.docx]

| up | down |
| --- | --- |
| CXCL3 | CLCA4 |
| MMP3 | ZG16 |
| CDH3 | GUCA2A |
| MMP1 | MS4A12 |
| FOXQ1 | GUCA2B |
| MMP7 | CA4 |
| CXCL1 | AQP8 |
| KRT23 | CHP2 |
| NFE2L3 | ADH1C |
| TGFBI | MT1M |
| VSNL1 | SCNN1B |
| COL11A1 | CD177 |
| LGR5 | HSD17B2 |
| EPHX4 | DHRS9 |
| SLCO1B3 | CLDN8 |
| PSAT1 | CA2 |
| INHBA | CLEC3B |
| SLC7A5 | BTNL8 |
| NMU | AKR1B10 |
| CLDN1 | HPGD |
| CRNDE | LRRC19 |
| CEMIP | PCK1 |
| TCN1 | CA1 |
| CXCL11 | GCG |
| PPBP | CHGA |
| EGFL6 | ABCG2 |
| C2CD4A | CFD |
| CXCL8 | CLCA1 |
| SPP1 | TSPAN7 |
| REG3A | FCGBP |
| SLC35D3 | SLC26A3 |
| CLDN2 | SLC26A2 |
| SERPINB5 | HMGCS2 |
| LRP8 | NR3C2 |
| CYP4X1 | SRPX |
| CTHRC1 | LGALS2 |
| TDGF1 | ADH1B |
| KLK6 | UGT1A1 |
| CHI3L1 | SI |
| TMPRSS3 | CXCL12 |
| CKMT2 | BEST2 |
| KRT6B | AHCYL2 |
| TESC | GBA3 |
| ASCL2 | CKB |
| ETV4 | HSD11B2 |
| KRT80 | INSL5 |
|  | CA7 |
|  | SLC4A4 |
|  | GDPD3 |
|  | KRT20 |
|  | BCAS1 |
|  | CEACAM7 |
|  | ENTPD5 |
|  | CWH43 |
|  | PLAC8 |
|  | KLF4 |
|  | MEP1A |
|  | DPT |
|  | CDHR5 |
|  | GPX3 |
|  | SGK1 |
|  | DHRS11 |
|  | CLU |
|  | EPB41L3 |
|  | ADAMDEC1 |
|  | MALL |
|  | STMN2 |
|  | ARL14 |
|  | CA12 |
|  | CEACAM1 |
|  | BTNL3 |
|  | CDHR2 |
|  | MFAP5 |
|  | SPINK5 |
|  | CES2 |
|  | TUBAL3 |
|  | BCHE |
|  | GPA33 |
|  | PKIB |
|  | MAOA |
|  | CNN1 |
|  | ITM2C |
|  | VIP |
|  | RETSAT |
|  | SST |
|  | APPL2 |
|  | PAPSS2 |
|  | BEST4 |
|  | PDE9A |
|  | BMP2 |
|  | C2orf88 |
|  | GCNT3 |
|  | PLP1 |
|  | SELENBP1 |
|  | MT1F |
|  | FGL2 |
|  | TSPAN1 |
|  | FABP1 |
|  | EDN3 |
|  | UGT2A3 |
|  | MEP1B |
|  | SPIB |
|  | DEFB1 |
|  | SCGN |
|  | CHRDL1 |
|  | GHR |
|  | ABCB1 |
|  | HHLA2 |
|  | SEPP1 |
|  | PIGZ |
|  | NR1H4 |
|  | SLCO2A1 |
|  | TRPM6 |
|  | CHST5 |
|  | SECTM1 |
|  | SYNM |
|  | SLC17A4 |
|  | PTPRH |
|  | MT1H |
|  | C1orf115 |
|  | RUNDC3B |
|  | CLDN23 |
|  | NXPE4 |
|  | OGN |
|  | KRT24 |
|  | SLC30A10 |
|  | CCL19 |
|  | GREM2 |
|  | VSIG2 |
|  | SMPDL3A |
|  | MT1E |
|  | HEPACAM2 |
|  | CDKN2B |
|  | UGT2B17 |
|  | TEX11 |
|  | CILP |
|  | LDHD |
|  | CCDC68 |
|  | SCGB2A1 |
|  | PYY |
|  | TMEM100 |
|  | MT1G |
|  | ITLN1 |
|  | CCL23 |
|  | C2orf40 |
|  | ACTG2 |
|  | SCG2 |
|  | PCOLCE2 |
|  | TNFRSF17 |
|  | MT1X |
|  | PADI2 |
|  | CXCL13 |
|  | ANPEP |
|  | IL1R2 |
|  | PDE6A |
|  | MYH11 |
|  | MAMDC2 |
|  | ANO5 |
|  | TP53INP2 |
|  | CNTN3 |
|  | SCARA5 |
|  | IL6R |
|  | SLC16A9 |
|  | LAMA1 |
|  | C11orf86 |
|  | TMIGD1 |
