# Supplementary material for: Identification of Hub Genes Related to Carcinogenesis and Prognosis in Colorectal Cancer Based on Integrated Bioinformatics
Source: Mediators Inflamm. 2020 Apr 9;2020:5934821. doi: 10.1155/2020/5934821 (PMC7171686; doi:10.1155/2020/5934821)
Supplement: Supplementary 4 — Table S4: the clinical information of patients organized for survival analysis. [file 5934821.f4.docx]

| id | futime | fustat |
| --- | --- | --- |
| TCGA-AA-3970 | 1096 | 0 |
| TCGA-AA-A00E | 913 | 0 |
| TCGA-A6-6140 | 734 | 0 |
| TCGA-AZ-6608 | 59 | 1 |
| TCGA-F4-6463 | 1087 | 0 |
| TCGA-A6-4107 | 987 | 0 |
| TCGA-AA-3494 | 31 | 0 |
| TCGA-CM-6674 | 394 | 0 |
| TCGA-AD-6963 | 834 | 0 |
| TCGA-AA-A00N | 122 | 1 |
| TCGA-DM-A28M | 2895 | 0 |
| TCGA-D5-5537 | 1381 | 1 |
| TCGA-AA-A004 | 424 | 0 |
| TCGA-A6-5657 | 962 | 0 |
| TCGA-AA-A01Z | 1126 | 0 |
| TCGA-AA-3862 | 914 | 0 |
| TCGA-AA-3975 | 1036 | 0 |
| TCGA-DM-A28F | 1094 | 1 |
| TCGA-AA-A02E | 90 | 1 |
| TCGA-A6-6142 | 763 | 0 |
| TCGA-AA-A010 | 1064 | 0 |
| TCGA-AA-3971 | 489 | 0 |
| TCGA-AA-3675 | 1431 | 0 |
| TCGA-AA-A02K | 426 | 1 |
| TCGA-D5-6541 | 474 | 0 |
| TCGA-AA-3866 | 518 | 0 |
| TCGA-CK-5912 | 1493 | 1 |
| TCGA-A6-5666 | 995 | 0 |
| TCGA-CM-6162 | 365 | 0 |
| TCGA-DM-A1D9 | 4270 | 0 |
| TCGA-AA-3663 | 212 | 0 |
| TCGA-AA-3950 | 730 | 0 |
| TCGA-A6-A56B | 1711 | 1 |
| TCGA-CM-5348 | 699 | 0 |
| TCGA-NH-A50V | 588 | 0 |
| TCGA-QG-A5YW | 896 | 0 |
| TCGA-DM-A285 | 179 | 1 |
| TCGA-A6-2677 | 740 | 1 |
| TCGA-AA-3666 | 61 | 1 |
| TCGA-G4-6314 | 1093 | 0 |
| TCGA-A6-2685 | 1133 | 0 |
| TCGA-G4-6302 | 0 | 1 |
| TCGA-A6-5665 | 671 | 0 |
| TCGA-CM-6166 | 669 | 0 |
| TCGA-CM-4751 | 822 | 0 |
| TCGA-T9-A92H | 362 | 0 |
| TCGA-G4-6295 | 254 | 0 |
| TCGA-A6-3810 | 1111 | 0 |
| TCGA-AA-3681 | 182 | 0 |
| TCGA-AA-A029 | 1581 | 0 |
| TCGA-AA-A00A | 1157 | 0 |
| TCGA-CK-4947 | 534 | 0 |
| TCGA-CM-6164 | 883 | 0 |
| TCGA-A6-2679 | 1366 | 0 |
| TCGA-AA-3517 | 1186 | 0 |
| TCGA-DM-A28K | 2988 | 0 |
| TCGA-A6-5659 | 926 | 0 |
| TCGA-AA-A02R | 670 | 1 |
| TCGA-G4-6304 | 1631 | 0 |
| TCGA-AA-3672 | 0 | 0 |
| TCGA-AD-6890 | 746 | 0 |
| TCGA-D5-6932 | 346 | 0 |
| TCGA-CM-5862 | 153 | 1 |
| TCGA-D5-6539 | 380 | 0 |
| TCGA-AA-3855 | 975 | 0 |
| TCGA-AD-6964 | 331 | 1 |
| TCGA-F4-6856 | 1074 | 0 |
| TCGA-CM-6678 | 335 | 0 |
| TCGA-AA-A03J | 1246 | 0 |
| TCGA-AA-3867 | 731 | 0 |
| TCGA-3L-AA1B | 475 | 0 |
| TCGA-A6-6782 | 617 | 0 |
| TCGA-DM-A28A | 805 | 1 |
| TCGA-AA-A01G | 365 | 0 |
| TCGA-AA-3966 | 61 | 0 |
| TCGA-AM-5820 | 14 | 0 |
| TCGA-AA-A00Z | 669 | 0 |
| TCGA-NH-A6GB | 476 | 0 |
| TCGA-A6-6651 | 662 | 0 |
| TCGA-AA-3534 | 882 | 0 |
| TCGA-D5-5538 | 1661 | 1 |
| TCGA-5M-AAT6 | 290 | 1 |
| TCGA-CA-6718 | 306 | 1 |
| TCGA-A6-2682 | 424 | 1 |
| TCGA-AA-3542 | 395 | 0 |
| TCGA-AA-3538 | 791 | 0 |
| TCGA-CM-5349 | 915 | 0 |
| TCGA-QG-A5YX | 1003 | 0 |
| TCGA-AY-A54L | 525 | 0 |
| TCGA-A6-5664 | 672 | 0 |
| TCGA-AY-5543 | 1004 | 0 |
| TCGA-A6-5656 | 1001 | 0 |
| TCGA-AA-A01V | 31 | 0 |
| TCGA-D5-6927 | 287 | 0 |
| TCGA-DM-A28E | 3648 | 0 |
| TCGA-AA-A00K | 549 | 0 |
| TCGA-D5-6923 | 378 | 0 |
| TCGA-DM-A1D0 | 3974 | 0 |
| TCGA-A6-2676 | 1305 | 1 |
| TCGA-AA-3543 | 30 | 0 |
| TCGA-CM-6169 | 396 | 0 |
| TCGA-AA-3684 | 0 | 0 |
| TCGA-CM-6161 | 457 | 0 |
| TCGA-AA-3510 | 1946 | 0 |
| TCGA-DM-A1D4 | 2821 | 1 |
| TCGA-F4-6805 | 1047 | 0 |
| TCGA-AA-3872 | 0 | 0 |
| TCGA-AA-3977 | 761 | 0 |
| TCGA-DM-A1HB | 4126 | 0 |
| TCGA-5M-AAT4 | 49 | 1 |
| TCGA-AA-3667 | 426 | 0 |
| TCGA-AU-3779 | 441 | 0 |
| TCGA-AA-A02F | 1216 | 0 |
| TCGA-AA-3488 | 153 | 1 |
| TCGA-CM-5864 | 457 | 0 |
| TCGA-AA-3984 | 0 | 0 |
| TCGA-AA-3949 | 791 | 0 |
| TCGA-G4-6586 | 1089 | 0 |
| TCGA-A6-3807 | 1054 | 0 |
| TCGA-AD-A5EJ | 0 | 0 |
| TCGA-AA-A02H | 61 | 1 |
| TCGA-DM-A28G | 1849 | 1 |
| TCGA-CA-6716 | 371 | 0 |
| TCGA-F4-6569 | 1087 | 0 |
| TCGA-AZ-4313 | 2310 | 0 |
| TCGA-AA-3489 | 214 | 1 |
| TCGA-CM-5341 | 884 | 0 |
| TCGA-AA-3846 | 518 | 0 |
| TCGA-A6-2680 | 1068 | 0 |
| TCGA-D5-6529 | 614 | 0 |
| TCGA-AA-3956 | 1035 | 0 |
| TCGA-AA-3841 | 1124 | 0 |
| TCGA-AZ-6601 | 3042 | 1 |
| TCGA-D5-5540 | 1706 | 0 |
| TCGA-G4-6627 | 2175 | 0 |
| TCGA-AA-A01Q | 31 | 0 |
| TCGA-AA-A00F | 1035 | 0 |
| TCGA-G4-6321 | 672 | 0 |
| TCGA-AA-3495 | 1127 | 0 |
| TCGA-D5-6537 | 146 | 1 |
| TCGA-CM-6170 | 457 | 0 |
| TCGA-CK-5915 | 0 | 0 |
| TCGA-AA-3562 | 608 | 0 |
| TCGA-CA-6717 | 388 | 0 |
| TCGA-AA-3844 | 454 | 0 |
| TCGA-G4-6323 | 419 | 0 |
| TCGA-G4-6628 | 2424 | 0 |
| TCGA-AA-3986 | 580 | 0 |
| TCGA-A6-2684 | 1127 | 0 |
| TCGA-F4-6855 | 1442 | 0 |
| TCGA-DM-A288 | 427 | 1 |
| TCGA-WS-AB45 | 2130 | 0 |
| TCGA-AA-3852 | 0 | 1 |
| TCGA-AA-3837 | 1186 | 0 |
| TCGA-AA-3845 | 0 | 1 |
| TCGA-D5-6898 | 229 | 0 |
| TCGA-CK-6747 | 820 | 0 |
| TCGA-SS-A7HO | 1829 | 0 |
| TCGA-AA-3511 | 212 | 0 |
| TCGA-D5-6532 | 555 | 0 |
| TCGA-G4-6315 | 1883 | 0 |
| TCGA-DM-A280 | 236 | 1 |
| TCGA-AZ-6598 | 1503 | 1 |
| TCGA-A6-2683 | 504 | 1 |
| TCGA-G4-6309 | 2600 | 0 |
| TCGA-NH-A50U | 334 | 1 |
| TCGA-CM-6675 | 397 | 0 |
| TCGA-AA-A00D | 578 | 0 |
| TCGA-AA-A02W | 1247 | 0 |
| TCGA-G4-6626 | 1 | 1 |
| TCGA-AA-A00R | 30 | 0 |
| TCGA-AA-3518 | 31 | 0 |
| TCGA-CM-6171 | 427 | 0 |
| TCGA-AD-6889 | 2532 | 1 |
| TCGA-AZ-4684 | 1977 | 0 |
| TCGA-G4-6299 | 2268 | 0 |
| TCGA-AA-3548 | 1034 | 0 |
| TCGA-NH-A8F7 | 543 | 0 |
| TCGA-D5-6922 | 308 | 0 |
| TCGA-D5-6920 | 377 | 0 |
| TCGA-G4-6625 | 2792 | 0 |
| TCGA-AA-3818 | 30 | 1 |
| TCGA-G4-6293 | 4051 | 0 |
| TCGA-AY-4070 | 496 | 1 |
| TCGA-AA-A02Y | 1216 | 0 |
| TCGA-4N-A93T | 146 | 0 |
| TCGA-AA-3685 | 1127 | 0 |
| TCGA-AA-3532 | 882 | 0 |
| TCGA-AM-5821 | 28 | 0 |
| TCGA-AA-A01R | 1065 | 0 |
| TCGA-AA-3864 | 1612 | 0 |
| TCGA-A6-2678 | 1286 | 0 |
| TCGA-AA-3821 | 31 | 0 |
| TCGA-AA-A017 | 457 | 0 |
| TCGA-AU-6004 | 824 | 0 |
| TCGA-A6-2675 | 1321 | 0 |
| TCGA-AA-3516 | 396 | 1 |
| TCGA-NH-A5IV | 0 | 0 |
| TCGA-G4-6303 | 2003 | 1 |
| TCGA-AA-3555 | 911 | 0 |
| TCGA-F4-6807 | 1309 | 0 |
| TCGA-AA-3947 | 1004 | 0 |
| TCGA-AA-3979 | 730 | 0 |
| TCGA-A6-3809 | 996 | 0 |
| TCGA-A6-2674 | 1331 | 0 |
| TCGA-AA-3502 | 1065 | 0 |
| TCGA-DM-A1DA | 228 | 1 |
| TCGA-AZ-6600 | 368 | 1 |
| TCGA-A6-A5ZU | 293 | 0 |
| TCGA-AA-3692 | 1095 | 1 |
| TCGA-AA-3860 | 945 | 0 |
| TCGA-CK-4951 | 2134 | 1 |
| TCGA-AA-3848 | 306 | 1 |
| TCGA-A6-6780 | 612 | 0 |
| TCGA-AA-3549 | 639 | 0 |
| TCGA-G4-6310 | 1935 | 0 |
| TCGA-D5-6540 | 491 | 0 |
| TCGA-AA-3930 | 61 | 1 |
| TCGA-AA-3679 | 457 | 0 |
| TCGA-G4-6320 | 804 | 0 |
| TCGA-AY-6197 | 652 | 0 |
| TCGA-A6-6648 | 766 | 0 |
| TCGA-D5-6930 | 406 | 0 |
| TCGA-F4-6460 | 972 | 1 |
| TCGA-AA-3955 | 638 | 0 |
| TCGA-AA-3941 | 730 | 0 |
| TCGA-CK-5916 | 643 | 1 |
| TCGA-G4-6322 | 792 | 0 |
| TCGA-CM-6680 | 366 | 0 |
| TCGA-CM-5868 | 518 | 0 |
| TCGA-AA-3552 | 396 | 1 |
| TCGA-CM-5863 | 457 | 0 |
| TCGA-CM-5861 | 457 | 0 |
| TCGA-AA-3660 | 2375 | 0 |
| TCGA-F4-6703 | 1456 | 0 |
| TCGA-AZ-6606 | 357 | 1 |
| TCGA-G4-6306 | 1359 | 0 |
| TCGA-DM-A28C | 2475 | 1 |
| TCGA-AA-A022 | 0 | 0 |
| TCGA-F4-6809 | 403 | 1 |
| TCGA-A6-6649 | 735 | 0 |
| TCGA-AZ-6603 | 899 | 1 |
| TCGA-AD-6901 | 682 | 1 |
| TCGA-AA-3697 | 2587 | 0 |
| TCGA-AZ-4682 | 680 | 1 |
| TCGA-F4-6459 | 262 | 1 |
| TCGA-CM-4743 | 701 | 0 |
| TCGA-AA-3854 | 1096 | 0 |
| TCGA-AA-3520 | 731 | 0 |
| TCGA-AA-3492 | 1 | 1 |
| TCGA-D5-6931 | 365 | 0 |
| TCGA-AZ-4308 | 3324 | 0 |
| TCGA-NH-A8F8 | 511 | 1 |
| TCGA-AZ-4614 | 172 | 1 |
| TCGA-AY-A8YK | 573 | 0 |
| TCGA-AA-3680 | 335 | 1 |
| TCGA-DM-A0XF | 1162 | 1 |
| TCGA-AD-6888 | 472 | 1 |
| TCGA-AA-3712 | 0 | 0 |
| TCGA-AA-3858 | 945 | 0 |
| TCGA-AA-3519 | 276 | 0 |
| TCGA-A6-6781 | 598 | 0 |
| TCGA-5M-AATE | 1200 | 0 |
| TCGA-AA-A03F | 0 | 1 |
| TCGA-AA-3842 | 1126 | 0 |
| TCGA-AA-A00O | 822 | 0 |
| TCGA-A6-A566 | 758 | 1 |
| TCGA-CM-4744 | 609 | 0 |
| TCGA-AA-3968 | 669 | 0 |
| TCGA-CK-4948 | 4502 | 0 |
| TCGA-DM-A1D6 | 570 | 1 |
| TCGA-AA-3561 | 424 | 0 |
| TCGA-CM-6676 | 337 | 0 |
| TCGA-CM-4752 | 396 | 0 |
| TCGA-A6-6138 | 685 | 0 |
| TCGA-AA-A01F | 974 | 0 |
| TCGA-AA-3980 | 242 | 0 |
| TCGA-AA-3553 | 730 | 0 |
| TCGA-AA-3554 | 546 | 0 |
| TCGA-G4-6297 | 2506 | 0 |
| TCGA-A6-5661 | 1020 | 0 |
| TCGA-CK-6751 | 518 | 0 |
| TCGA-CA-6719 | 435 | 0 |
| TCGA-AA-3972 | 1551 | 0 |
| TCGA-AA-3556 | 700 | 0 |
| TCGA-AA-3939 | 395 | 0 |
| TCGA-F4-6704 | 47 | 0 |
| TCGA-CM-6172 | 335 | 0 |
| TCGA-D5-6535 | 460 | 0 |
| TCGA-AA-3715 | 579 | 1 |
| TCGA-AA-A00L | 1157 | 0 |
| TCGA-CK-5913 | 1561 | 0 |
| TCGA-AA-3710 | 821 | 0 |
| TCGA-A6-2681 | 1387 | 0 |
| TCGA-AA-A01S | 31 | 0 |
| TCGA-D5-6538 | 521 | 0 |
| TCGA-AA-3815 | 1005 | 0 |
| TCGA-AD-6965 | 805 | 0 |
| TCGA-CK-6746 | 0 | 0 |
| TCGA-A6-4105 | 442 | 1 |
| TCGA-AZ-4323 | 43 | 1 |
| TCGA-G4-6317 | 1095 | 0 |
| TCGA-AA-3989 | 242 | 1 |
| TCGA-A6-2686 | 1126 | 1 |
| TCGA-CM-4750 | 244 | 0 |
| TCGA-AA-3693 | 0 | 0 |
| TCGA-CM-4747 | 761 | 0 |
| TCGA-AA-3496 | 31 | 0 |
| TCGA-A6-3808 | 1014 | 0 |
| TCGA-AA-3509 | 1915 | 0 |
| TCGA-AA-A024 | 1188 | 1 |
| TCGA-RU-A8FL | 1177 | 0 |
| TCGA-AY-4071 | 29 | 1 |
| TCGA-QL-A97D | 666 | 0 |
| TCGA-AZ-5407 | 2683 | 0 |
| TCGA-CA-5254 | 386 | 0 |
| TCGA-AA-3560 | 608 | 0 |
| TCGA-AA-A01T | 1005 | 0 |
| TCGA-A6-A567 | 1881 | 1 |
| TCGA-AA-3870 | 912 | 0 |
| TCGA-AA-A00Q | 1278 | 0 |
| TCGA-AA-3531 | 1035 | 0 |
| TCGA-AA-3851 | 1006 | 0 |
| TCGA-AA-3982 | 822 | 0 |
| TCGA-A6-5667 | 887 | 0 |
| TCGA-CM-4746 | 1126 | 0 |
| TCGA-NH-A6GA | 302 | 1 |
| TCGA-A6-2672 | 1419 | 0 |
| TCGA-AA-A01P | 1158 | 1 |
| TCGA-DM-A1HA | 2600 | 0 |
| TCGA-AA-3655 | 1856 | 0 |
| TCGA-AZ-6607 | 97 | 1 |
| TCGA-AA-3976 | 791 | 0 |
| TCGA-CM-4748 | 792 | 0 |
| TCGA-A6-5662 | 718 | 0 |
| TCGA-AA-3814 | 0 | 0 |
| TCGA-QG-A5Z1 | 256 | 1 |
| TCGA-G4-6307 | 1674 | 0 |
| TCGA-NH-A6GC | 389 | 0 |
| TCGA-AA-A02J | 153 | 1 |
| TCGA-AA-3662 | 184 | 0 |
| TCGA-F4-6808 | 1024 | 0 |
| TCGA-F4-6806 | 1260 | 0 |
| TCGA-AA-A01C | 457 | 0 |
| TCGA-CK-4952 | 475 | 0 |
| TCGA-CM-6167 | 456 | 0 |
| TCGA-AA-3544 | 426 | 0 |
| TCGA-AA-A02O | 28 | 0 |
| TCGA-AD-6895 | 763 | 0 |
| TCGA-G4-6298 | 0 | 1 |
| TCGA-AA-3514 | 31 | 0 |
| TCGA-D5-6533 | 775 | 0 |
| TCGA-AA-3811 | 306 | 1 |
| TCGA-AA-3861 | 914 | 0 |
| TCGA-AD-6899 | 176 | 1 |
| TCGA-G4-6294 | 858 | 1 |
| TCGA-D5-5541 | 1701 | 0 |
| TCGA-AZ-4616 | 156 | 1 |
| TCGA-AA-A01D | 334 | 1 |
| TCGA-D5-6928 | 354 | 0 |
| TCGA-4T-AA8H | 385 | 0 |
| TCGA-AA-A01I | 943 | 0 |
| TCGA-CA-5797 | 383 | 0 |
| TCGA-A6-6650 | 627 | 0 |
| TCGA-AA-3875 | 549 | 0 |
| TCGA-CM-6165 | 488 | 0 |
| TCGA-CK-5914 | 669 | 0 |
| TCGA-CM-5860 | 974 | 0 |
| TCGA-A6-6141 | 255 | 0 |
| TCGA-DM-A28H | 3561 | 0 |
| TCGA-DM-A1D8 | 383 | 1 |
| TCGA-AY-A71X | 588 | 0 |
| TCGA-AA-3812 | 1066 | 0 |
| TCGA-AZ-4315 | 1776 | 0 |
| TCGA-AY-6386 | 542 | 0 |
| TCGA-QG-A5YV | 1301 | 0 |
| TCGA-AA-3527 | 0 | 0 |
| TCGA-CK-6748 | 61 | 0 |
| TCGA-F4-6570 | 188 | 1 |
| TCGA-DM-A1DB | 1348 | 1 |
| TCGA-A6-2671 | 1331 | 1 |
| TCGA-AA-3952 | 61 | 1 |
| TCGA-G4-6311 | 1199 | 0 |
| TCGA-AA-3713 | 579 | 0 |
| TCGA-D5-6926 | 275 | 0 |
| TCGA-CM-6163 | 427 | 0 |
| TCGA-DM-A0XD | 743 | 1 |
| TCGA-CA-6715 | 383 | 0 |
| TCGA-AA-3688 | 578 | 0 |
| TCGA-AA-3526 | 580 | 0 |
| TCGA-A6-6137 | 824 | 0 |
| TCGA-CA-5256 | 379 | 0 |
| TCGA-AA-3856 | 30 | 0 |
| TCGA-CA-5796 | 377 | 0 |
| TCGA-AA-A01X | 791 | 0 |
| TCGA-AA-3529 | 0 | 1 |
| TCGA-A6-6652 | 751 | 0 |
| TCGA-CM-6677 | 337 | 0 |
| TCGA-D5-6530 | 621 | 0 |
| TCGA-DM-A282 | 4233 | 0 |
| TCGA-CM-6679 | 306 | 0 |
| TCGA-D5-6534 | 1316 | 0 |
| TCGA-AA-A00U | 518 | 0 |
| TCGA-A6-A565 | 494 | 1 |
| TCGA-A6-5660 | 888 | 0 |
| TCGA-QG-A5Z2 | 952 | 0 |
| TCGA-AD-6548 | 650 | 0 |
| TCGA-AA-A01K | 943 | 0 |
| TCGA-AA-3833 | 485 | 0 |
| TCGA-AA-3973 | 397 | 0 |
| TCGA-AY-6196 | 6 | 0 |
| TCGA-AA-3664 | 1643 | 0 |
| TCGA-DM-A1D7 | 405 | 1 |
| TCGA-AA-3525 | 1 | 0 |
| TCGA-G4-6588 | 796 | 0 |
| TCGA-CA-5255 | 376 | 0 |
| TCGA-AA-3869 | 822 | 1 |
| TCGA-AA-A00J | 549 | 0 |
| TCGA-AZ-6605 | 159 | 1 |
| TCGA-AA-A00W | 456 | 0 |
| TCGA-A6-6653 | 742 | 0 |
| TCGA-D5-6924 | 435 | 0 |
| TCGA-AY-A69D | 543 | 0 |
| TCGA-AA-3524 | 1096 | 0 |
| TCGA-AA-3831 | 547 | 0 |
| TCGA-DM-A0X9 | 3641 | 0 |
| TCGA-CM-6168 | 395 | 0 |
| TCGA-D5-6929 | 408 | 0 |
| TCGA-D5-5539 | 596 | 0 |
| TCGA-AD-A5EK | 500 | 0 |
| TCGA-AA-3819 | 761 | 0 |
| TCGA-AA-3530 | 580 | 0 |
| TCGA-D5-6531 | 540 | 0 |
| TCGA-F4-6461 | 338 | 1 |
| TCGA-AA-3877 | 943 | 0 |
| TCGA-AZ-4615 | 1002 | 0 |
| TCGA-AA-3850 | 0 | 1 |
| TCGA-CK-4950 | 2599 | 0 |
| TCGA-AA-3522 | 1127 | 0 |
| TCGA-NH-A50T | 553 | 0 |
| TCGA-F4-6854 | 16 | 0 |
| TCGA-AZ-5403 | 1910 | 1 |
| TCGA-CM-5344 | 670 | 0 |
| TCGA-A6-6654 | 726 | 0 |
| TCGA-D5-7000 | 312 | 0 |
| TCGA-D5-6536 | 543 | 0 |
| TCGA-AA-3994 | 822 | 0 |
| TCGA-AA-3673 | 1522 | 0 |
| TCGA-AZ-6599 | 206 | 1 |
| TCGA-AA-3506 | 1765 | 0 |
| TCGA-AD-5900 | 370 | 0 |
| TCGA-AA-3678 | 1430 | 0 |
| TCGA-AA-3696 | 153 | 1 |
| TCGA-EI-6514 | 496 | 0 |
| TCGA-F5-6465 | 1506 | 0 |
| TCGA-CI-6624 | 1466 | 0 |
| TCGA-AH-6897 | 804 | 0 |
| TCGA-AG-A00C | 183 | 0 |
| TCGA-AG-3882 | 608 | 0 |
| TCGA-DC-6683 | 762 | 0 |
| TCGA-AG-3894 | 426 | 0 |
| TCGA-AG-3883 | 31 | 0 |
| TCGA-AG-A016 | 276 | 0 |
| TCGA-EI-6510 | 556 | 0 |
| TCGA-CI-6622 | 1362 | 0 |
| TCGA-CI-6620 | 1009 | 0 |
| TCGA-AG-A015 | 1096 | 0 |
| TCGA-AG-A023 | 1581 | 1 |
| TCGA-AG-4021 | 121 | 1 |
| TCGA-G5-6233 | 556 | 1 |
| TCGA-G5-6641 | 804 | 0 |
| TCGA-EI-6885 | 415 | 0 |
| TCGA-DC-6156 | 943 | 0 |
| TCGA-AG-4005 | 427 | 0 |
| TCGA-EI-6882 | 262 | 0 |
| TCGA-AG-A008 | 424 | 0 |
| TCGA-AG-A026 | 59 | 1 |
| TCGA-AF-A56N | 360 | 0 |
| TCGA-AG-3602 | 0 | 0 |
| TCGA-AF-3400 | 1049 | 0 |
| TCGA-AG-3896 | 31 | 0 |
| TCGA-EI-6509 | 517 | 0 |
| TCGA-CI-6623 | 1443 | 0 |
| TCGA-AF-A56L | 2007 | 0 |
| TCGA-DC-6158 | 334 | 1 |
| TCGA-AF-5654 | 512 | 1 |
| TCGA-AG-3999 | 853 | 0 |
| TCGA-AG-3599 | 366 | 0 |
| TCGA-AG-3731 | 1126 | 0 |
| TCGA-DY-A1DG | 1566 | 1 |
| TCGA-AG-3594 | 61 | 1 |
| TCGA-F5-6813 | 598 | 1 |
| TCGA-AH-6644 | 838 | 0 |
| TCGA-AG-3898 | 1461 | 0 |
| TCGA-CI-6619 | 184 | 0 |
| TCGA-CL-5918 | 0 | 0 |
| TCGA-AG-A00H | 790 | 0 |
| TCGA-AG-3887 | 1124 | 0 |
| TCGA-AF-2692 | 412 | 0 |
| TCGA-AG-3598 | 1522 | 0 |
| TCGA-EF-5831 | 127 | 0 |
| TCGA-AG-3587 | 1400 | 0 |
| TCGA-AG-A02N | 1885 | 0 |
| TCGA-AG-A02G | 1185 | 1 |
| TCGA-AG-4015 | 0 | 0 |
| TCGA-AG-3725 | 0 | 0 |
| TCGA-EI-6508 | 636 | 0 |
| TCGA-AF-4110 | 912 | 0 |
| TCGA-AF-6136 | 741 | 0 |
| TCGA-AG-3732 | 1003 | 0 |
| TCGA-F5-6861 | 1160 | 0 |
| TCGA-EI-6507 | 607 | 0 |
| TCGA-EF-5830 | 106 | 0 |
| TCGA-AG-3609 | 608 | 0 |
| TCGA-EI-6883 | 350 | 0 |
| TCGA-AH-6547 | 76 | 1 |
| TCGA-DC-6160 | 1339 | 0 |
| TCGA-DC-6682 | 762 | 0 |
| TCGA-AF-6655 | 609 | 0 |
| TCGA-AG-A01L | 0 | 0 |
| TCGA-DY-A1DD | 1741 | 1 |
| TCGA-AG-3582 | 1096 | 1 |
| TCGA-AG-3881 | 579 | 0 |
| TCGA-AG-4008 | 518 | 0 |
| TCGA-AG-A00Y | 700 | 0 |
| TCGA-AF-2687 | 1427 | 0 |
| TCGA-AG-3893 | 1065 | 0 |
| TCGA-AG-3901 | 761 | 0 |
| TCGA-AG-3601 | 0 | 0 |
| TCGA-EI-6881 | 499 | 0 |
| TCGA-AG-A011 | 1126 | 0 |
| TCGA-CI-6621 | 419 | 0 |
| TCGA-AG-A02X | 1247 | 0 |
| TCGA-AG-3878 | 30 | 0 |
| TCGA-G5-6235 | 1696 | 0 |
| TCGA-AG-4007 | 31 | 0 |
| TCGA-AH-6544 | 1173 | 0 |
| TCGA-EI-7004 | 257 | 0 |
| TCGA-AH-6643 | 1314 | 1 |
| TCGA-AG-A01W | 0 | 0 |
| TCGA-AG-3593 | 1035 | 0 |
| TCGA-F5-6812 | 1110 | 0 |
| TCGA-DC-4749 | 762 | 0 |
| TCGA-DY-A1H8 | 992 | 1 |
| TCGA-F5-6864 | 379 | 0 |
| TCGA-DT-5265 | 384 | 0 |
| TCGA-AG-3580 | 244 | 0 |
| TCGA-G5-6572 | 1432 | 1 |
| TCGA-AG-4001 | 1096 | 0 |
| TCGA-F5-6814 | 1131 | 0 |
| TCGA-EI-6511 | 482 | 0 |
| TCGA-DC-6155 | 425 | 0 |
| TCGA-AG-3892 | 396 | 0 |
| TCGA-AG-3909 | 608 | 0 |
| TCGA-EI-6512 | 538 | 0 |
| TCGA-AG-3902 | 974 | 0 |
| TCGA-AF-3913 | 316 | 1 |
| TCGA-F5-6464 | 303 | 1 |
| TCGA-AG-3727 | 30 | 0 |
| TCGA-AG-A014 | 485 | 0 |
| TCGA-AF-6672 | 748 | 0 |
| TCGA-DC-6154 | 365 | 0 |
| TCGA-AG-3581 | 215 | 0 |
| TCGA-EI-6884 | 328 | 0 |
| TCGA-AG-A036 | 3562 | 0 |
| TCGA-DY-A0XA | 3846 | 0 |
| TCGA-AG-3591 | 1035 | 0 |
| TCGA-AF-2693 | 1155 | 0 |
| TCGA-AH-6549 | 532 | 0 |
| TCGA-F5-6863 | 361 | 1 |
| TCGA-AG-A01Y | 0 | 0 |
| TCGA-AG-3612 | 608 | 0 |
| TCGA-AF-2691 | 1309 | 0 |
| TCGA-AG-3583 | 610 | 1 |
| TCGA-AG-3726 | 243 | 0 |
| TCGA-AG-3575 | 365 | 0 |
| TCGA-AG-3890 | 518 | 0 |
| TCGA-DC-6681 | 790 | 0 |
| TCGA-AG-3742 | 30 | 0 |
| TCGA-F5-6702 | 869 | 1 |
| TCGA-AG-3605 | 30 | 0 |
| TCGA-AG-3578 | 974 | 0 |
| TCGA-AG-A032 | 1157 | 0 |
| TCGA-AG-A020 | 31 | 0 |
| TCGA-AH-6903 | 592 | 0 |
| TCGA-DC-6157 | 1581 | 0 |
| TCGA-DC-4745 | 639 | 0 |
| TCGA-AG-3592 | 1035 | 0 |
| TCGA-AG-3584 | 730 | 1 |
| TCGA-AG-3600 | 184 | 0 |
| TCGA-EI-6917 | 531 | 0 |
| TCGA-AG-4022 | 1400 | 0 |
| TCGA-DC-5869 | 943 | 0 |
| TCGA-AG-3611 | 424 | 0 |
| TCGA-DC-5337 | 792 | 0 |
| TCGA-EI-6506 | 625 | 0 |
| TCGA-F5-6571 | 1288 | 0 |
| TCGA-DY-A1DF | 734 | 1 |
| TCGA-EI-7002 | 364 | 0 |
| TCGA-AG-A01J | 31 | 0 |
| TCGA-F5-6811 | 979 | 0 |
| TCGA-AG-3728 | 912 | 0 |
| TCGA-AG-A01N | 943 | 0 |
| TCGA-AG-3574 | 91 | 1 |
| TCGA-BM-6198 | 646 | 0 |
| TCGA-AF-2689 | 1201 | 1 |
| TCGA-EI-6513 | 497 | 0 |
| TCGA-AG-A002 | 638 | 0 |
| TCGA-AG-3608 | 485 | 0 |
| TCGA-AG-A025 | 1520 | 0 |
| TCGA-DY-A1DE | 3932 | 0 |
| TCGA-AF-2690 | 524 | 1 |
| TCGA-AG-3885 | 546 | 0 |
| TCGA-AG-3586 | 31 | 0 |
| TCGA-AF-3911 | 1148 | 0 |
| TCGA-CL-5917 | 2376 | 0 |
| TCGA-DY-A1DC | 1258 | 1 |
| TCGA-AF-A56K | 2635 | 0 |
