# Supplementary material for: Identification of Hub Genes Related to Carcinogenesis and Prognosis in Colorectal Cancer Based on Integrated Bioinformatics
Source: Mediators Inflamm. 2020 Apr 9;2020:5934821. doi: 10.1155/2020/5934821 (PMC7171686; doi:10.1155/2020/5934821)
Supplement: Supplementary 5 — Table S5: the biological processes analyzed for overlapping DEGs. [file 5934821.f5.docx]

| description | count | ratio | pValue | adjustedPValue | regulated DEGs |
| --- | --- | --- | --- | --- | --- |
| chemotaxis | 5 | 0.128 | 9.03E-05 | 7.13E-03 | up |
| defense response | 8 | 0.205 | 2.23E-04 | 1.22E-02 | up |
| inflammatory response | 6 | 0.153 | 1.92E-04 | 1.22E-02 | up |
| locomotory behavior | 5 | 0.128 | 8.27E-04 | 3.92E-02 | up |
| response to external stimulus | 7 | 0.179 | 6.38E-04 | 3.23E-02 | up |
| response to wounding | 8 | 0.205 | 8.69E-05 | 7.13E-03 | up |
| regulation of cell adhesion | 5 | 0.128 | 4.66E-05 | 5.51E-03 | up |
| collagen catabolic process | 4 | 0.102 | 2.73E-07 | 1.94E-04 | up |
| neutrophil chemotaxis | 2 | 0.051 | 1.08E-03 | 4.79E-02 | up |
| multicellular organismal process | 23 | 0.589 | 2.21E-04 | 1.22E-02 | up |
| collagen metabolic process | 4 | 0.102 | 1.23E-06 | 2.91E-04 | up |
| locomotion | 7 | 0.179 | 1.63E-04 | 1.16E-02 | up |
| taxis | 5 | 0.128 | 9.03E-05 | 7.13E-03 | up |
| multicellular organismal metabolic process | 4 | 0.102 | 3.63E-06 | 5.15E-04 | up |
| multicellular organismal catabolic process | 4 | 0.102 | 7.92E-07 | 2.81E-04 | up |
| multicellular organismal macromolecule metabolic process | 4 | 0.102 | 1.83E-06 | 3.24E-04 | up |
| lipid metabolic process | 22 | 0.162 | 1.07E-05 | 4.65E-03 | down |
| chemotaxis | 8 | 0.059 | 2.00E-04 | 2.19E-02 | down |
| response to nutrient | 8 | 0.059 | 1.92E-04 | 2.19E-02 | down |
| digestion | 7 | 0.051 | 2.39E-05 | 7.80E-03 | down |
| response to external stimulus | 18 | 0.133 | 4.25E-06 | 2.77E-03 | down |
| response to hormone stimulus | 13 | 0.096 | 2.75E-04 | 2.56E-02 | down |
| response to extracellular stimulus | 10 | 0.074 | 2.02E-04 | 2.19E-02 | down |
| secondary metabolic process | 5 | 0.037 | 2.71E-04 | 2.56E-02 | down |
| response to nutrient levels | 10 | 0.074 | 1.02E-04 | 1.67E-02 | down |
| response to corticosteroid stimulus | 7 | 0.051 | 5.67E-05 | 1.06E-02 | down |
| response to chemical stimulus | 33 | 0.244 | 1.53E-06 | 1.99E-03 | down |
| taxis | 8 | 0.059 | 2.00E-04 | 2.19E-02 | down |
| response to steroid hormone stimulus | 10 | 0.074 | 5.40E-05 | 1.06E-02 | down |
| response to glucocorticoid stimulus | 7 | 0.051 | 3.62E-05 | 9.45E-03 | down |
