# Supplementary material for: Identification of Hub Genes Related to Carcinogenesis and Prognosis in Colorectal Cancer Based on Integrated Bioinformatics
Source: Mediators Inflamm. 2020 Apr 9;2020:5934821. doi: 10.1155/2020/5934821 (PMC7171686; doi:10.1155/2020/5934821)
Supplement: Supplementary 6 — Table S6: the molecular functions analyzed for overlapping DEGs. [file 5934821.f6.docx]

| description | count | ratio | pValue | adjustedPValue | regulated DEGs |
| --- | --- | --- | --- | --- | --- |
| G-protein-coupled receptor binding | 6 | 0.146 | 1.40E-06 | 4.96E-05 | up |
| endopeptidase activity | 5 | 0.121 | 2.87E-03 | 2.91E-02 | up |
| metalloendopeptidase activity | 3 | 0.073 | 2.56E-03 | 2.90E-02 | up |
| O-phospho-L-serine:2-oxoglutarate aminotransferase activity | 1 | 0.024 | 2.66E-03 | 2.90E-02 | up |
| receptor binding | 11 | 0.268 | 1.94E-05 | 5.50E-04 | up |
| cytokine activity | 7 | 0.17 | 7.71E-07 | 3.65E-05 | up |
| cytokine receptor binding | 5 | 0.121 | 1.27E-04 | 2.57E-03 | up |
| interleukin-8 receptor binding | 1 | 0.024 | 5.30E-03 | 4.43E-02 | up |
| structural molecule activity | 7 | 0.17 | 9.74E-04 | 1.54E-02 | up |
| calcium ion binding | 7 | 0.17 | 1.17E-03 | 1.66E-02 | up |
| chemokine activity | 5 | 0.121 | 1.45E-07 | 1.73E-05 | up |
| growth factor activity | 4 | 0.097 | 8.15E-04 | 1.45E-02 | up |
| apolipoprotein E binding | 1 | 0.024 | 5.30E-03 | 4.43E-02 | up |
| chemokine receptor binding | 5 | 0.121 | 2.43E-07 | 1.73E-05 | up |
| neuromedin U receptor binding | 1 | 0.024 | 2.66E-03 | 2.90E-02 | up |
| follistatin binding | 1 | 0.024 | 5.30E-03 | 4.43E-02 | up |
| extracellular matrix binding | 3 | 0.073 | 6.71E-05 | 1.59E-03 | up |
| alcohol dehydrogenase (NAD) activity | 5 | 0.035 | 1.71E-07 | 6.35E-05 | down |
| carbonate dehydratase activity | 9 | 0.063 | 1.13E-06 | 2.09E-04 | down |
| receptor binding | 8 | 0.056 | 5.15E-06 | 6.37E-04 | down |
| cytokine activity | 6 | 0.042 | 3.15E-05 | 2.61E-03 | down |
| hormone activity | 5 | 0.035 | 3.52E-05 | 2.61E-03 | down |
| chloride channel activity | 8 | 0.056 | 5.53E-05 | 2.87E-03 | down |
| steroid binding | 3 | 0.021 | 6.15E-05 | 2.87E-03 | down |
| chemokine activity | 7 | 0.049 | 6.18E-05 | 2.87E-03 | down |
| secondary active sulfate transmembrane transporter activity | 21 | 0.147 | 1.05E-04 | 4.32E-03 | down |
| selenium binding | 5 | 0.035 | 1.35E-04 | 5.01E-03 | down |
| anion transmembrane transporter activity | 2 | 0.014 | 2.51E-04 | 7.96E-03 | down |
| xenobiotic-transporting ATPase activity | 3 | 0.021 | 2.58E-04 | 7.96E-03 | down |
| cyclase regulator activity | 4 | 0.028 | 3.22E-04 | 9.18E-03 | down |
| cyclase activator activity | 8 | 0.056 | 4.76E-04 | 1.23E-02 | down |
| glucuronosyltransferase activity | 2 | 0.014 | 4.98E-04 | 1.23E-02 | down |
| inorganic anion transmembrane transporter activity | 4 | 0.028 | 9.01E-04 | 2.09E-02 | down |
| fatty acid transporter activity | 2 | 0.014 | 1.23E-03 | 2.68E-02 | down |
| steroid dehydrogenase activity | 4 | 0.028 | 1.32E-03 | 2.72E-02 | down |
| oxidoreductase activity, acting on CH-OH group of donors | 3 | 0.021 | 1.51E-03 | 2.89E-02 | down |
| oxidoreductase activity, acting on the CH-OH group of donors, NAD or NADP as acceptor | 2 | 0.014 | 1.71E-03 | 2.89E-02 | down |
| lyase activity | 2 | 0.014 | 1.71E-03 | 2.89E-02 | down |
| carbon-oxygen lyase activity | 2 | 0.014 | 1.71E-03 | 2.89E-02 | down |
| hydro-lyase activity | 2 | 0.014 | 2.27E-03 | 3.47E-02 | down |
| transmembrane transporter activity | 2 | 0.014 | 2.27E-03 | 3.47E-02 | down |
| guanylate cyclase regulator activity | 3 | 0.021 | 2.34E-03 | 3.47E-02 | down |
| guanylate cyclase activator activity | 3 | 0.021 | 2.58E-03 | 3.68E-02 | down |
| bile acid binding | 2 | 0.014 | 2.90E-03 | 3.72E-02 | down |
| steroid dehydrogenase activity, acting on the CH-OH group of donors, NAD or NADP as acceptor | 2 | 0.014 | 2.90E-03 | 3.72E-02 | down |
| chemokine receptor binding | 6 | 0.042 | 2.91E-03 | 3.72E-02 | down |
| xenobiotic transporter activity | 4 | 0.028 | 3.54E-03 | 4.18E-02 | down |
| cadmium ion binding | 17 | 0.119 | 3.58E-03 | 4.18E-02 | down |
| 3',5'-cyclic-GMP phosphodiesterase activity | 2 | 0.014 | 3.60E-03 | 4.18E-02 | down |
