# Supplementary material for: Identification of Hub Genes Related to Carcinogenesis and Prognosis in Colorectal Cancer Based on Integrated Bioinformatics
Source: Mediators Inflamm. 2020 Apr 9;2020:5934821. doi: 10.1155/2020/5934821 (PMC7171686; doi:10.1155/2020/5934821)
Supplement: Supplementary 7 — Table S7: the reactome pathway enriched for upregulated overlapping DEGs. [file 5934821.f7.docx]

| ReactomePathway | Ratio | Count | Pvalue | FDR |
| --- | --- | --- | --- | --- |
| Chemokine receptors bind chemokines | 0.0059 | 5 | 5.36E-07 | 4.13E-05 |
| Peptide ligand-binding receptors | 0.0233 | 6 | 3.09E-05 | 1.17E-03 |
| G alpha (i) signalling events | 0.0266 | 6 | 6.50E-05 | 1.62E-03 |
| Activation of Matrix Metalloproteinases | 0.0036 | 3 | 1.27E-04 | 2.41E-03 |
| Degradation of the extracellular matrix | 0.0109 | 4 | 2.02E-04 | 3.03E-03 |
| Collagen degradation | 0.0048 | 3 | 3.02E-04 | 3.62E-03 |
| Class A/1 (Rhodopsin-like receptors) | 0.0379 | 6 | 4.40E-04 | 4.83E-03 |
| GPCR ligand binding | 0.0489 | 6 | 1.66E-03 | 0.0149 |
| Basigin interactions | 0.0031 | 2 | 3.19E-03 | 0.0255 |
| Keratinization | 0.0134 | 3 | 5.50E-03 | 0.0385 |
| Interleukin-4 and 13 signaling | 0.0135 | 3 | 5.64E-03 | 0.0395 |
