# Supplementary material for: Identification of Hub Genes Related to Carcinogenesis and Prognosis in Colorectal Cancer Based on Integrated Bioinformatics
Source: Mediators Inflamm. 2020 Apr 9;2020:5934821. doi: 10.1155/2020/5934821 (PMC7171686; doi:10.1155/2020/5934821)
Supplement: Supplementary 8 — Table S8: the reactome pathway enriched for downregulated overlapping DEGs. [file 5934821.f8.docx]

| ReactomePathway | Ratio | Count | Pvalue | FDR |
| --- | --- | --- | --- | --- |
| Response to metal ions | 0.001 | 6 | 4.45E-10 | 4.31E-08 |
| Metallothioneins bind metals | 0.001 | 6 | 4.45E-10 | 4.31E-08 |
| Reversible hydration of carbon dioxide | 0.0004 | 2 | 5.05E-04 | 0.0326 |
| Miscellaneous digestion events | 0.0005 | 2 | 8.92E-04 | 0.0326 |
| Peptide ligand-binding receptors | 0.0233 | 8 | 9.83E-04 | 0.0326 |
| Synthesis, secretion, and deacylation of Ghrelin | 0.0022 | 3 | 1.02E-03 | 0.0326 |
| Bicarbonate transporters | 0.0006 | 2 | 1.38E-03 | 0.0332 |
| Transport and synthesis of PAPS | 0.0006 | 2 | 1.38E-03 | 0.0332 |
| Signaling by BMP | 0.0028 | 3 | 2.05E-03 | 0.0382 |
| Stimuli-sensing channels | 0.0103 | 5 | 2.11E-03 | 0.0382 |
| Abacavir transport and metabolism | 0.0009 | 2 | 2.67E-03 | 0.0382 |
| Erythrocytes take up oxygen and release carbon dioxide | 0.0009 | 2 | 2.67E-03 | 0.0382 |
| Transport of inorganic cations/anions and amino acids/oligopeptides | 0.0068 | 4 | 3.05E-03 | 0.0382 |
| Biological oxidations | 0.0165 | 6 | 3.23E-03 | 0.0382 |
| Multifunctional anion exchangers | 0.001 | 2 | 3.47E-03 | 0.0382 |
| Erythrocytes take up carbon dioxide and release oxygen | 0.001 | 2 | 3.47E-03 | 0.0382 |
| O2/CO2 exchange in erythrocytes | 0.001 | 2 | 3.47E-03 | 0.0382 |
