# Supplementary material for: Identification of Hub Genes Related to Carcinogenesis and Prognosis in Colorectal Cancer Based on Integrated Bioinformatics
Source: Mediators Inflamm. 2020 Apr 9;2020:5934821. doi: 10.1155/2020/5934821 (PMC7171686; doi:10.1155/2020/5934821)
Supplement: Supplementary 9 — Table S9: the protein-protein interactions among overlapping DEGs identified with combined score ≥ 0.4. [file 5934821.f9.docx]

| #node1 | node2 | combined_score |
| --- | --- | --- |
| CCL19 | CXCL13 | 0.993 |
| BMP2 | GREM2 | 0.992 |
| ACTG2 | MYH11 | 0.986 |
| CXCL1 | CXCL8 | 0.985 |
| CXCL11 | PPBP | 0.979 |
| SPP1 | MMP7 | 0.979 |
| CXCL12 | CCL19 | 0.976 |
| CXCL12 | CXCL13 | 0.976 |
| CXCL11 | CXCL3 | 0.976 |
| CXCL12 | CXCL11 | 0.975 |
| ADH1C | ADH1B | 0.973 |
| SPP1 | MMP3 | 0.972 |
| CXCL12 | CXCL8 | 0.972 |
| PYY | SST | 0.971 |
| CXCL11 | CXCL13 | 0.97 |
| CXCL1 | CXCL11 | 0.969 |
| CCL19 | CXCL11 | 0.969 |
| CXCL12 | CXCL1 | 0.969 |
| CXCL11 | CXCL8 | 0.968 |
| CXCL1 | CXCL3 | 0.966 |
| SGK1 | SCNN1B | 0.965 |
| CXCL1 | CXCL13 | 0.965 |
| CXCL8 | CXCL3 | 0.964 |
| CXCL12 | PPBP | 0.963 |
| HMGCS2 | FABP1 | 0.96 |
| CXCL1 | CCL19 | 0.957 |
| CCL19 | CXCL8 | 0.956 |
| CXCL3 | CXCL13 | 0.954 |
| MMP1 | MMP3 | 0.953 |
| CXCL12 | CXCL3 | 0.952 |
| CCL19 | CXCL3 | 0.947 |
| BMP2 | CHRDL1 | 0.946 |
| PPBP | CXCL13 | 0.941 |
| MMP1 | MMP7 | 0.941 |
| CCL19 | PPBP | 0.94 |
| PYY | INSL5 | 0.938 |
| MAOA | ADH1B | 0.938 |
| GCG | SST | 0.938 |
| KRT80 | KRT20 | 0.935 |
| CD177 | CEACAM1 | 0.933 |
| SEPP1 | CLEC3B | 0.933 |
| PYY | NMU | 0.932 |
| SST | NMU | 0.932 |
| CXCL8 | PPBP | 0.93 |
| KRT80 | KRT24 | 0.927 |
| COL11A1 | PCOLCE2 | 0.927 |
| GCG | VIP | 0.927 |
| CXCL8 | SST | 0.926 |
| KRT24 | KRT20 | 0.925 |
| CXCL1 | CHI3L1 | 0.925 |
| GCG | PYY | 0.925 |
| KRT6B | KRT23 | 0.923 |
| GCG | NMU | 0.923 |
| KRT24 | KRT6B | 0.923 |
| INSL5 | NMU | 0.922 |
| CFD | CLU | 0.922 |
| CXCL12 | SST | 0.921 |
| CLCA4 | CLCA1 | 0.92 |
| CLDN23 | CLDN8 | 0.919 |
| KRT80 | KRT23 | 0.919 |
| CLDN2 | CLDN23 | 0.917 |
| CLDN23 | CLDN1 | 0.917 |
| CHST5 | OGN | 0.917 |
| INSL5 | SST | 0.917 |
| CLU | PPBP | 0.916 |
| KRT6B | KRT20 | 0.916 |
| SPP1 | SCG2 | 0.914 |
| TCN1 | CHI3L1 | 0.914 |
| CLDN2 | CLDN1 | 0.914 |
| DHRS9 | RETSAT | 0.914 |
| MT1G | MT1H | 0.913 |
| ADH1C | RETSAT | 0.913 |
| PPBP | CXCL3 | 0.912 |
| GCG | EDN3 | 0.912 |
| CLDN2 | CLDN8 | 0.912 |
| CXCL8 | CXCL13 | 0.912 |
| MMP3 | MMP7 | 0.91 |
| CFD | PPBP | 0.91 |
| PYY | CXCL8 | 0.91 |
| CXCL1 | PPBP | 0.909 |
| CLDN8 | CLDN1 | 0.909 |
| SST | CXCL13 | 0.908 |
| CXCL12 | PYY | 0.908 |
| KRT24 | KRT23 | 0.908 |
| CXCL1 | SST | 0.908 |
| ACTG2 | CNN1 | 0.907 |
| KRT80 | KRT6B | 0.906 |
| KRT23 | KRT20 | 0.906 |
| CXCL1 | TCN1 | 0.905 |
| PLAC8 | PADI2 | 0.905 |
| PPBP | TCN1 | 0.905 |
| CXCL8 | NMU | 0.903 |
| ADH1B | RETSAT | 0.903 |
| CCL19 | SST | 0.903 |
| PPBP | SST | 0.902 |
| EDN3 | NMU | 0.902 |
| CXCL3 | SST | 0.902 |
| CXCL11 | SST | 0.902 |
| CXCL12 | NMU | 0.902 |
| PYY | CXCL11 | 0.901 |
| CXCL1 | PYY | 0.901 |
| PYY | CCL19 | 0.9 |
| PPBP | NMU | 0.9 |
| CHRDL1 | SCG2 | 0.9 |
| CNTN3 | CEACAM7 | 0.9 |
| CXCL8 | INSL5 | 0.9 |
| CXCL11 | NMU | 0.9 |
| CXCL11 | INSL5 | 0.9 |
| CXCL3 | NMU | 0.9 |
| INSL5 | CXCL13 | 0.9 |
| CXCL12 | INSL5 | 0.9 |
| CXCL1 | INSL5 | 0.9 |
| PYY | CXCL13 | 0.9 |
| SPP1 | CHRDL1 | 0.9 |
| INSL5 | PPBP | 0.9 |
| PYY | CXCL3 | 0.9 |
| CCL19 | NMU | 0.9 |
| CXCL1 | NMU | 0.9 |
| MEP1B | MEP1A | 0.9 |
| CCL19 | INSL5 | 0.9 |
| PYY | PPBP | 0.9 |
| CXCL13 | NMU | 0.9 |
| INSL5 | CXCL3 | 0.9 |
| MYH11 | CNN1 | 0.893 |
| SST | CHGA | 0.892 |
| VIP | SST | 0.892 |
| SLC4A4 | AHCYL2 | 0.89 |
| SPP1 | BMP2 | 0.88 |
| UGT1A1 | CES2 | 0.874 |
| CDHR2 | CDHR5 | 0.866 |
| SCG2 | CHGA | 0.86 |
| GUCA2A | TMIGD1 | 0.837 |
| SEPP1 | GPX3 | 0.837 |
| NR3C2 | HSD11B2 | 0.836 |
| MT1G | MT1X | 0.833 |
| MT1X | MT1H | 0.831 |
| MT1H | MT1E | 0.831 |
| MT1X | MT1E | 0.83 |
| GCG | PCK1 | 0.83 |
| SLC4A4 | CA4 | 0.824 |
| SEPP1 | LRP8 | 0.822 |
| DHRS11 | HSD17B2 | 0.809 |
| UGT2B17 | ADH1B | 0.804 |
| NR1H4 | GCG | 0.802 |
| SLC4A4 | CA2 | 0.8 |
| MT1X | MT1F | 0.786 |
| MT1G | MT1E | 0.784 |
| MT1G | MT1F | 0.782 |
| MT1F | MT1H | 0.769 |
| NR1H4 | FABP1 | 0.764 |
| MMP1 | CXCL8 | 0.751 |
| HSD11B2 | HPGD | 0.751 |
| UGT2B17 | HSD17B2 | 0.748 |
| SLCO1B3 | ABCG2 | 0.744 |
| GCG | SI | 0.74 |
| ASCL2 | LGR5 | 0.738 |
| SLCO2A1 | HPGD | 0.738 |
| GUCA2B | GUCA2A | 0.736 |
| GCG | CHGA | 0.731 |
| CES2 | UGT2A3 | 0.729 |
| HHLA2 | TMIGD1 | 0.728 |
| GUCA2B | TMIGD1 | 0.728 |
| MT1F | MT1E | 0.728 |
| ADH1C | MAOA | 0.727 |
| UGT1A1 | HSD17B2 | 0.726 |
| SI | CLCA1 | 0.726 |
| PLP1 | BCAS1 | 0.718 |
| ABCB1 | SLCO1B3 | 0.707 |
| CXCL8 | CHI3L1 | 0.705 |
| CXCL8 | MMP3 | 0.703 |
| ADH1C | UGT2A3 | 0.7 |
| MT1M | MT1E | 0.7 |
| SGK1 | NR3C2 | 0.698 |
| ADH1C | UGT2B17 | 0.693 |
| STMN2 | CHGA | 0.693 |
| UGT2A3 | HSD17B2 | 0.691 |
| PAPSS2 | SLC26A2 | 0.691 |
| UGT1A1 | ADH1B | 0.684 |
| SLC16A9 | SLC17A4 | 0.683 |
| ABCB1 | UGT1A1 | 0.678 |
| TMIGD1 | CLCA1 | 0.677 |
| UGT2B17 | CES2 | 0.676 |
| GUCA2A | CLCA1 | 0.676 |
| VIP | PYY | 0.672 |
| CA1 | SELENBP1 | 0.671 |
| KLK6 | SPINK5 | 0.67 |
| ZG16 | CLCA1 | 0.67 |
| CHGA | KRT20 | 0.669 |
| ADH1C | UGT1A1 | 0.666 |
| KLF4 | LGR5 | 0.665 |
| FCGBP | CLCA1 | 0.664 |
| ABCB1 | RUNDC3B | 0.663 |
| ADH1B | UGT2A3 | 0.661 |
| UGT1A1 | ABCG2 | 0.658 |
| ENTPD5 | PDE6A | 0.657 |
| ENTPD5 | PDE9A | 0.657 |
| GUCA2A | SLC26A3 | 0.652 |
| UGT1A1 | SLCO1B3 | 0.65 |
| COL11A1 | CILP | 0.65 |
| FCGBP | ZG16 | 0.649 |
| CXCL8 | MMP7 | 0.645 |
| COL11A1 | MMP3 | 0.64 |
| CXCL12 | SPP1 | 0.639 |
| CLU | LRP8 | 0.633 |
| PYY | CHGA | 0.631 |
| PCK1 | FABP1 | 0.631 |
| HMGCS2 | PCK1 | 0.627 |
| SLC4A4 | SLC26A3 | 0.624 |
| VIP | CHGA | 0.624 |
| TMIGD1 | MS4A12 | 0.616 |
| CCL23 | CCL19 | 0.613 |
| MAOA | BCHE | 0.613 |
| SLC16A9 | ABCG2 | 0.607 |
| GUCA2B | MS4A12 | 0.603 |
| IL6R | CXCL8 | 0.601 |
| SPP1 | CXCL8 | 0.599 |
| CXCL1 | MMP3 | 0.594 |
| MT1M | SLC30A10 | 0.593 |
| NR3C2 | SCNN1B | 0.592 |
| LGR5 | CHGA | 0.592 |
| NR3C2 | HSD17B2 | 0.589 |
| SLC30A10 | MT1H | 0.588 |
| CXCL1 | MMP1 | 0.587 |
| SLC16A9 | SLC30A10 | 0.587 |
| SLC30A10 | MT1F | 0.586 |
| MT1X | SLC30A10 | 0.584 |
| MT1G | SLC30A10 | 0.583 |
| FCGBP | MEP1B | 0.582 |
| SLC30A10 | MT1E | 0.582 |
| SLC4A4 | CA12 | 0.581 |
| FABP1 | UGT2A3 | 0.581 |
| CXCL8 | ABCG2 | 0.58 |
| TGFBI | MMP3 | 0.578 |
| GDPD3 | ZG16 | 0.578 |
| SLC4A4 | CA7 | 0.577 |
| GUCA2B | SLC26A3 | 0.575 |
| CXCL8 | CEACAM1 | 0.574 |
| NR1H4 | UGT1A1 | 0.573 |
| NR1H4 | SLCO1B3 | 0.573 |
| ZG16 | TMIGD1 | 0.573 |
| BMP2 | CILP | 0.57 |
| KLF4 | TDGF1 | 0.568 |
| SPP1 | ABCG2 | 0.567 |
| CLCA4 | MS4A12 | 0.566 |
| SPP1 | CLU | 0.565 |
| ZG16 | MS4A12 | 0.562 |
| BEST2 | BEST4 | 0.559 |
| GCG | REG3A | 0.559 |
| CCL23 | CXCL1 | 0.558 |
| BMP2 | OGN | 0.558 |
| CCL23 | CXCL13 | 0.557 |
| MT1M | MT1F | 0.556 |
| MMP1 | INHBA | 0.556 |
| CCL23 | CXCL11 | 0.555 |
| IL6R | IL1R2 | 0.553 |
| BEST2 | CLCA1 | 0.553 |
| MT1G | MT1M | 0.552 |
| SLC26A3 | TMIGD1 | 0.552 |
| CA1 | SLC4A4 | 0.546 |
| CLCA4 | GUCA2A | 0.545 |
| MT1M | MT1H | 0.543 |
| SLC26A3 | CLCA1 | 0.543 |
| VSNL1 | CHI3L1 | 0.535 |
| ANPEP | SI | 0.533 |
| BEST4 | TMIGD1 | 0.533 |
| KLF4 | ABCG2 | 0.531 |
| NR3C2 | HPGD | 0.527 |
| MMP3 | PPBP | 0.526 |
| GUCA2B | CLCA1 | 0.524 |
| CTHRC1 | MMP7 | 0.524 |
| GUCA2A | SI | 0.524 |
| TMIGD1 | SI | 0.523 |
| CXCL8 | DEFB1 | 0.522 |
| NR1H4 | PYY | 0.522 |
| IL1R2 | CXCL8 | 0.522 |
| SPP1 | MMP1 | 0.52 |
| SLC30A10 | SLC26A2 | 0.52 |
| NR1H4 | PCK1 | 0.52 |
| NR1H4 | UGT2A3 | 0.519 |
| CXCL12 | BMP2 | 0.518 |
| ZG16 | GUCA2B | 0.518 |
| DPT | OGN | 0.517 |
| CCL23 | CXCL8 | 0.515 |
| CLCA1 | MS4A12 | 0.514 |
| AQP8 | MS4A12 | 0.513 |
| SI | MEP1A | 0.512 |
| CLEC3B | CILP | 0.511 |
| FABP1 | MEP1A | 0.51 |
| SPP1 | CHI3L1 | 0.509 |
| CCL23 | CXCL3 | 0.507 |
| FCGBP | MAMDC2 | 0.507 |
| SLC30A10 | SLC26A3 | 0.505 |
| SCG2 | SST | 0.504 |
| CXCL1 | MMP7 | 0.502 |
| CXCL1 | SPP1 | 0.501 |
| SLC16A9 | SLC26A2 | 0.5 |
| LGR5 | KRT20 | 0.5 |
| SLC26A3 | MS4A12 | 0.499 |
| CKMT2 | LDHD | 0.499 |
| ABCB1 | ABCG2 | 0.498 |
| CXCL12 | MMP1 | 0.497 |
| MMP3 | CHI3L1 | 0.495 |
| SLC26A3 | AQP8 | 0.495 |
| GCG | SLC30A10 | 0.495 |
| CTHRC1 | PCOLCE2 | 0.494 |
| PCK1 | CHGA | 0.493 |
| CXCL12 | MMP3 | 0.492 |
| TMIGD1 | MEP1B | 0.492 |
| CXCL1 | IL1R2 | 0.489 |
| GHR | SST | 0.488 |
| CFD | ITLN1 | 0.488 |
| CLCA4 | TMIGD1 | 0.487 |
| LGR5 | ABCG2 | 0.486 |
| CLCA1 | MEP1A | 0.485 |
| SLC26A3 | CA4 | 0.484 |
| GUCA2B | CLCA4 | 0.483 |
| ETV4 | CXCL8 | 0.483 |
| GUCA2A | MEP1A | 0.481 |
| GPX3 | SLC26A2 | 0.48 |
| GCNT3 | CHST5 | 0.48 |
| BEST4 | MS4A12 | 0.478 |
| SLC16A9 | SLC26A3 | 0.478 |
| GCG | GUCA2A | 0.476 |
| CHRDL1 | GREM2 | 0.476 |
| SLC17A4 | SLC26A3 | 0.475 |
| BEST4 | CLCA1 | 0.475 |
| CCL23 | CXCL12 | 0.475 |
| SCNN1B | HSD11B2 | 0.475 |
| SI | KRT20 | 0.473 |
| MMP1 | CHI3L1 | 0.473 |
| NXPE4 | VSIG2 | 0.471 |
| FABP1 | KRT20 | 0.47 |
| CXCL1 | ANPEP | 0.469 |
| GHR | SPP1 | 0.469 |
| PCK1 | LDHD | 0.468 |
| CLCA4 | SI | 0.466 |
| SLC26A3 | KRT20 | 0.465 |
| CD177 | MS4A12 | 0.463 |
| MYH11 | KLF4 | 0.462 |
| CXCL1 | ABCG2 | 0.462 |
| CLCA1 | KRT20 | 0.459 |
| SCGN | CHGA | 0.459 |
| CCL23 | PPBP | 0.459 |
| KRT20 | MS4A12 | 0.456 |
| CES2 | ABCG2 | 0.455 |
| MMP1 | ETV4 | 0.455 |
| MYH11 | MFAP5 | 0.451 |
| ETV4 | MMP7 | 0.45 |
| ZG16 | GUCA2A | 0.45 |
| CEMIP | GUCA2B | 0.45 |
| CXCL1 | IL6R | 0.449 |
| CXCL8 | ANPEP | 0.447 |
| SLC30A10 | CHGA | 0.446 |
| MMP7 | TCN1 | 0.446 |
| CEMIP | MS4A12 | 0.445 |
| SLC26A3 | CA2 | 0.445 |
| NR1H4 | LGALS2 | 0.444 |
| PAPSS2 | FGL2 | 0.443 |
| KLF4 | CDKN2B | 0.443 |
| BMP2 | MMP1 | 0.441 |
| DPT | SLC30A10 | 0.441 |
| TMIGD1 | SECTM1 | 0.441 |
| NXPE4 | CHST5 | 0.44 |
| ZG16 | CLCA4 | 0.438 |
| CCDC68 | MAMDC2 | 0.437 |
| GCG | MEP1A | 0.436 |
| ZG16 | SLC26A3 | 0.436 |
| SLC26A3 | SI | 0.435 |
| BMP2 | KLF4 | 0.433 |
| OGN | CILP | 0.433 |
| REG3A | GUCA2B | 0.433 |
| FCGBP | CEACAM7 | 0.432 |
| CLCA4 | CEACAM7 | 0.432 |
| SCGN | SLC17A4 | 0.432 |
| NXPE4 | MS4A12 | 0.429 |
| CLCA4 | AQP8 | 0.429 |
| FCGBP | CLCA4 | 0.428 |
| CEMIP | CLCA4 | 0.428 |
| ENTPD5 | CTHRC1 | 0.427 |
| SCG2 | C2orf40 | 0.426 |
| BMP2 | MMP3 | 0.424 |
| MMP1 | CXCL3 | 0.422 |
| GUCA2B | KRT20 | 0.422 |
| SYNM | CLU | 0.42 |
| CLU | OGN | 0.42 |
| MMP1 | CXCL11 | 0.419 |
| SGK1 | HSD11B2 | 0.418 |
| GUCA2A | KRT20 | 0.417 |
| CLDN1 | CDH3 | 0.416 |
| TMIGD1 | MEP1A | 0.416 |
| NXPE4 | CLCA1 | 0.415 |
| DPT | MFAP5 | 0.415 |
| CXCL12 | IL6R | 0.415 |
| SLC17A4 | SLC30A10 | 0.414 |
| CA4 | ABCG2 | 0.414 |
| SRPX | HSD17B2 | 0.414 |
| HSD17B2 | NFE2L3 | 0.414 |
| PTPRH | GUCA2A | 0.414 |
| NR1H4 | HMGCS2 | 0.413 |
| PLP1 | STMN2 | 0.413 |
| TMIGD1 | KRT20 | 0.412 |
| CXCL12 | MMP7 | 0.41 |
| STMN2 | VSNL1 | 0.409 |
| SI | TCN1 | 0.408 |
| DPT | ANPEP | 0.407 |
| LGR5 | MMP7 | 0.406 |
| SLC26A3 | FABP1 | 0.405 |
| PSAT1 | SLC7A5 | 0.404 |
| GUCA2A | AQP8 | 0.404 |
| NXPE4 | CLCA4 | 0.404 |
| ITLN1 | CLCA1 | 0.404 |
| TMPRSS3 | HSD17B2 | 0.403 |
| HPGD | HSD17B2 | 0.4 |
| SLC17A4 | ABCG2 | 0.4 |
| VIP | SLC26A3 | 0.4 |
| SPP1 | DPT | 0.4 |
| MAMDC2 | CHRDL1 | 0.4 |
