# Supplementary material for: Identification of Hub Genes Related to Carcinogenesis and Prognosis in Colorectal Cancer Based on Integrated Bioinformatics
Source: Mediators Inflamm. 2020 Apr 9;2020:5934821. doi: 10.1155/2020/5934821 (PMC7171686; doi:10.1155/2020/5934821)
Supplement: Supplementary 10 — Table S10: three topological parameters calculated for module 1. [file 5934821.f10.docx]

| Gene | attribute | ClosenessCentrality | Degree | BetweennessCentrality |
| --- | --- | --- | --- | --- |
| CXCL3 | up | 1 | 11 | 0 |
| CXCL12 | down | 1 | 11 | 0 |
| CXCL13 | down | 1 | 11 | 0 |
| CCL19 | down | 1 | 11 | 0 |
| SST | down | 1 | 11 | 0 |
| PYY | down | 1 | 11 | 0 |
| PPBP | up | 1 | 11 | 0 |
| INSL5 | down | 1 | 11 | 0 |
| CXCL11 | up | 1 | 11 | 0 |
| NMU | up | 1 | 11 | 0 |
| CXCL8 | up | 1 | 11 | 0 |
| CXCL1 | up | 1 | 11 | 0 |
