# Supplementary material for: Identification of Hub Genes Related to Carcinogenesis and Prognosis in Colorectal Cancer Based on Integrated Bioinformatics
Source: Mediators Inflamm. 2020 Apr 9;2020:5934821. doi: 10.1155/2020/5934821 (PMC7171686; doi:10.1155/2020/5934821)
Supplement: Supplementary 11 — Table S11: three topological parameters calculated for module 2. [file 5934821.f11.docx]

| Gene | attribute | ClosenessCentrality | Degree | BetweennessCentrality |
| --- | --- | --- | --- | --- |
| SLC26A3 | down | 0.695652 | 9 | 0.532302 |
| SLC30A10 | down | 0.615385 | 7 | 0.501667 |
|  |  |  |  |  |
| CLCA1 | down | 0.551724 | 9 | 0.018214 |
| TMIGD1 | down | 0.551724 | 9 | 0.018214 |
| GUCA2A | down | 0.533333 | 8 | 0.015357 |
| GUCA2B | down | 0.533333 | 8 | 0.013492 |
| MS4A12 | down | 0.516129 | 7 | 0.012302 |
| ZG16 | down | 0.516129 | 7 | 0.010714 |
| CLCA4 | down | 0.421053 | 7 | 0.005913 |
| KRT20 | down | 0.516129 | 7 | 0.004246 |
| SI | down | 0.5 | 6 | 0.010913 |
| MT1H | down | 0.444444 | 6 | 0.001667 |
| MT1G | down | 0.444444 | 6 | 0.001667 |
| MT1E | down | 0.444444 | 6 | 0.001667 |
| MT1F | down | 0.444444 | 6 | 0.001667 |
| MT1X | down | 0.432432 | 5 | 0 |
| MT1M | down | 0.432432 | 5 | 0 |
|  |  | 0.505484 | 6.941176 | 0.067647 |
