# Supplementary material for: Identification of Hub Genes Related to Carcinogenesis and Prognosis in Colorectal Cancer Based on Integrated Bioinformatics
Source: Mediators Inflamm. 2020 Apr 9;2020:5934821. doi: 10.1155/2020/5934821 (PMC7171686; doi:10.1155/2020/5934821)
Supplement: Supplementary 12 — Table S12: the univariate Cox proportional hazards regression analysis for train group. [file 5934821.f12.docx]

| id | HR | HR.95L | HR.95H | pvalue |
| --- | --- | --- | --- | --- |
| SIX4 | 1.003203 | 1.001941 | 1.004466 | 6.33E-07 |
| SNAP25 | 1.001991 | 1.0012 | 1.002783 | 8.11E-07 |
| VGF | 1.000194 | 1.000113 | 1.000274 | 2.31E-06 |
| CREG2 | 1.002626 | 1.001516 | 1.003737 | 3.49E-06 |
| SPOCK3 | 1.006334 | 1.003631 | 1.009044 | 4.20E-06 |
| JPH3 | 1.003061 | 1.001747 | 1.004376 | 4.84E-06 |
| OTOP3 | 1.046254 | 1.026118 | 1.066785 | 5.11E-06 |
| CLVS2 | 1.009619 | 1.005404 | 1.013852 | 7.31E-06 |
| CELF4 | 1.006112 | 1.003418 | 1.008813 | 8.42E-06 |
| PIANP | 1.004767 | 1.002666 | 1.006873 | 8.51E-06 |
| CNTNAP4 | 1.025795 | 1.014165 | 1.037559 | 1.20E-05 |
| FCER2 | 1.001323 | 1.000729 | 1.001917 | 1.27E-05 |
| PAX5 | 1.000871 | 1.000476 | 1.001266 | 1.53E-05 |
| SFTA2 | 1.001236 | 1.000674 | 1.001798 | 1.61E-05 |
| PGPEP1L | 1.060516 | 1.031955 | 1.089868 | 2.46E-05 |
| SNCB | 1.013969 | 1.007341 | 1.020641 | 3.38E-05 |
| OFCC1 | 1.017051 | 1.008948 | 1.02522 | 3.44E-05 |
| LHX5 | 1.02362 | 1.012309 | 1.035058 | 3.83E-05 |
| LINC01158 | 1.046773 | 1.024162 | 1.069882 | 4.08E-05 |
| SCG3 | 1.000687 | 1.000356 | 1.001019 | 4.89E-05 |
| KLC3 | 1.003226 | 1.001667 | 1.004787 | 4.92E-05 |
| GABRG1 | 1.137697 | 1.068679 | 1.211172 | 5.34E-05 |
| POU4F1 | 1.005264 | 1.002698 | 1.007835 | 5.65E-05 |
| GABRD | 1.003641 | 1.001856 | 1.005429 | 6.28E-05 |
| TNNT1 | 1.000692 | 1.000352 | 1.001032 | 6.60E-05 |
| POU3F3 | 1.002932 | 1.001488 | 1.004379 | 6.86E-05 |
| ZNF676 | 1.033325 | 1.016706 | 1.050216 | 7.41E-05 |
| GRIN2A | 1.007565 | 1.003816 | 1.011328 | 7.43E-05 |
| RPH3A | 1.031859 | 1.015929 | 1.048039 | 7.79E-05 |
| DNAH17-AS1 | 1.018129 | 1.009062 | 1.027278 | 8.26E-05 |
| DUSP9 | 1.002058 | 1.001031 | 1.003087 | 8.52E-05 |
| GPR26 | 1.028761 | 1.014163 | 1.043568 | 0.000101 |
| SSTR2 | 1.001666 | 1.000823 | 1.00251 | 0.000107 |
| HPCAL4 | 1.01278 | 1.006272 | 1.019331 | 0.000113 |
| P2RX2 | 1.011137 | 1.005466 | 1.016839 | 0.000113 |
| LY6H | 1.008074 | 1.003964 | 1.0122 | 0.000114 |
| PLD5 | 1.030003 | 1.014543 | 1.045698 | 0.000128 |
| WBSCR28 | 1.02162 | 1.010442 | 1.032923 | 0.000139 |
| ACTL6B | 1.009061 | 1.004375 | 1.013769 | 0.000146 |
| TMEM151B | 1.008486 | 1.004096 | 1.012895 | 0.000147 |
| SIX2 | 1.001274 | 1.000616 | 1.001933 | 0.000149 |
| ERICH3 | 1.004953 | 1.002376 | 1.007535 | 0.000162 |
| MYH2 | 1.061345 | 1.029015 | 1.094692 | 0.000162 |
| GNG8 | 1.014573 | 1.006949 | 1.022256 | 0.00017 |
| DLL3 | 1.002697 | 1.001288 | 1.004107 | 0.000173 |
| SLITRK2 | 1.015785 | 1.00751 | 1.024128 | 0.000175 |
| IGSF11 | 1.008421 | 1.00401 | 1.012852 | 0.000177 |
| KCNC1 | 1.029231 | 1.013824 | 1.044872 | 0.000181 |
| PCSK2 | 1.001259 | 1.000597 | 1.001921 | 0.000192 |
| UNC80 | 1.007212 | 1.003405 | 1.011033 | 0.0002 |
| SLC7A14 | 1.002976 | 1.001406 | 1.004549 | 0.000201 |
| JPH4 | 1.003675 | 1.001733 | 1.005621 | 0.000205 |
| PROKR1 | 1.02239 | 1.010502 | 1.034417 | 0.000207 |
| KCNB2 | 1.015924 | 1.007468 | 1.02445 | 0.000211 |
| SLC17A7 | 1.011067 | 1.005195 | 1.016974 | 0.000213 |
| GABRG2 | 1.010441 | 1.004898 | 1.016015 | 0.000215 |
| ATP2B3 | 1.003293 | 1.001544 | 1.005045 | 0.000221 |
| SYT6 | 1.008039 | 1.003763 | 1.012333 | 0.000223 |
| RPRM | 1.001338 | 1.000627 | 1.002049 | 0.000225 |
| CDH10 | 1.08782 | 1.040208 | 1.137611 | 0.000228 |
| TMEM59L | 1.002332 | 1.001089 | 1.003577 | 0.000233 |
| POU3F4 | 1.053572 | 1.024657 | 1.083304 | 0.000237 |
| VGLL1 | 1.00449 | 1.00209 | 1.006895 | 0.000241 |
| ERBB4 | 1.02034 | 1.009418 | 1.031379 | 0.000245 |
| DPYSL5 | 1.001365 | 1.000635 | 1.002097 | 0.000249 |
| RND2 | 1.006023 | 1.002785 | 1.009272 | 0.000262 |
| LINC00461 | 1.041514 | 1.018977 | 1.06455 | 0.000268 |
| MCHR2 | 1.218594 | 1.095526 | 1.355487 | 0.000273 |
| METTL11B | 1.077589 | 1.034991 | 1.12194 | 0.000282 |
| ATCAY | 1.003601 | 1.00165 | 1.005556 | 0.000294 |
| AMH | 1.001066 | 1.000488 | 1.001644 | 0.000297 |
| TCL1A | 1.00151 | 1.000685 | 1.002336 | 0.000331 |
| SH3GL3 | 1.023799 | 1.010732 | 1.037035 | 0.000332 |
| DIRAS1 | 1.002614 | 1.001179 | 1.004051 | 0.000353 |
| MYT1L | 1.019051 | 1.008547 | 1.029665 | 0.000357 |
| PDZD4 | 1.002095 | 1.000943 | 1.003247 | 0.00036 |
| SLC8A2 | 1.002402 | 1.001076 | 1.003729 | 0.00038 |
| LRRC3B | 1.071197 | 1.031158 | 1.112791 | 0.000402 |
| FEV | 1.002544 | 1.001134 | 1.003956 | 0.000403 |
| NRXN1 | 1.002898 | 1.00129 | 1.004508 | 0.000409 |
| ASTN1 | 1.004648 | 1.002065 | 1.007238 | 0.000415 |
| INA | 1.002434 | 1.001081 | 1.003788 | 0.000418 |
| CHGA | 1.00006 | 1.000026 | 1.000093 | 0.000426 |
| HOTAIR | 1.004508 | 1.001997 | 1.007025 | 0.000428 |
| TCEAL5 | 1.013502 | 1.005925 | 1.021137 | 0.000461 |
| SLC39A12 | 1.083998 | 1.036055 | 1.134159 | 0.000475 |
| MAFA | 1.019659 | 1.008551 | 1.030889 | 0.000495 |
| CFC1 | 1.044545 | 1.019151 | 1.070572 | 0.000519 |
| CAMKV | 1.000997 | 1.000431 | 1.001563 | 0.000557 |
| PCDH11Y | 1.064712 | 1.027221 | 1.103572 | 0.000607 |
| CD19 | 1.001653 | 1.000701 | 1.002607 | 0.000667 |
| FIGF | 1.004263 | 1.0018 | 1.006733 | 0.000687 |
| SLC32A1 | 1.021473 | 1.008968 | 1.034133 | 0.000723 |
| ISM2 | 1.000272 | 1.000114 | 1.000431 | 0.000748 |
| DCAF12L2 | 1.054029 | 1.022203 | 1.086845 | 0.000769 |
| ATP8A2 | 1.006741 | 1.002795 | 1.010702 | 0.0008 |
| LINC01014 | 1.121598 | 1.048795 | 1.199455 | 0.000804 |
| RNU6-403P | 1.050268 | 1.020528 | 1.080875 | 0.000818 |
| CADM2 | 1.011806 | 1.004795 | 1.018866 | 0.000938 |
| COL19A1 | 1.025787 | 1.010409 | 1.041398 | 0.000954 |
| TUBB4A | 1.003726 | 1.001512 | 1.005946 | 0.000966 |
| DOCK3 | 1.003337 | 1.001351 | 1.005326 | 0.000982 |
