# Supplementary material for: Identification of Hub Genes Related to Carcinogenesis and Prognosis in Colorectal Cancer Based on Integrated Bioinformatics
Source: Mediators Inflamm. 2020 Apr 9;2020:5934821. doi: 10.1155/2020/5934821 (PMC7171686; doi:10.1155/2020/5934821)
Supplement: Supplementary 13 — Table S13: the LASSO penalized regression performed for train group. [file 5934821.f13.docx]

| id | futime | fustat | LHX5 | AMH | WBSCR28 | SFTA2 | SNAP25 | MYH2 | POU4F1 | SIX4 | ISM2 | KLC3 | CREG2 | OFCC1 | DNAH17-AS1 | METTL11B | PGPEP1L | PAX5 |
| --- | --- | --- | --- | --- | --- | --- | --- | --- | --- | --- | --- | --- | --- | --- | --- | --- | --- | --- |
| TCGA-A6-A567 | 5.153425 | 1 | 0 | 0 | 1 | 17 | 17 | 1 | 1 | 7 | 19 | 5 | 29 | 0 | 17 | 0 | 0 | 21 |
| TCGA-A6-6654 | 1.989041 | 0 | 0 | 135 | 22 | 2 | 57 | 4 | 21 | 97 | 13 | 32 | 20 | 0 | 10 | 4 | 1 | 286 |
| TCGA-DM-A0XF | 3.183562 | 1 | 2 | 17 | 48 | 425 | 4 | 0 | 37 | 50 | 5 | 55 | 13 | 16 | 31 | 2 | 1 | 2 |
| TCGA-AA-3815 | 2.753425 | 0 | 3 | 763 | 6 | 9 | 1 | 0 | 4 | 25 | 1 | 1 | 13 | 0 | 0 | 0 | 1 | 31 |
| TCGA-NH-A50T | 1.515068 | 0 | 1 | 18 | 17 | 44 | 4 | 0 | 30 | 18 | 48 | 4 | 9 | 1 | 14 | 1 | 1 | 1 |
| TCGA-CM-4744 | 1.668493 | 0 | 0 | 8 | 10 | 1305 | 9 | 0 | 0 | 16 | 6 | 14 | 69 | 2 | 6 | 0 | 0 | 28 |
| TCGA-AG-3612 | 1.665753 | 0 | 0 | 17 | 17 | 8 | 9 | 0 | 17 | 42 | 43 | 4 | 1 | 0 | 2 | 0 | 0 | 21 |
| TCGA-AZ-6607 | 0.265753 | 1 | 0 | 54 | 31 | 2146 | 75 | 2 | 11 | 390 | 3 | 351 | 466 | 15 | 6 | 34 | 0 | 25 |
| TCGA-EI-6513 | 1.361644 | 0 | 7 | 8 | 31 | 1568 | 33 | 1 | 36 | 20 | 407 | 58 | 58 | 63 | 16 | 3 | 1 | 12 |
| TCGA-AG-A026 | 0.161644 | 1 | 18 | 65 | 24 | 17 | 53 | 59 | 42 | 45 | 7 | 63 | 10 | 0 | 2 | 1 | 1 | 1 |
| TCGA-QG-A5YX | 2.747945 | 0 | 18 | 8 | 10 | 5 | 0 | 0 | 27 | 24 | 50 | 253 | 25 | 10 | 9 | 0 | 0 | 9 |
| TCGA-CM-5341 | 2.421918 | 0 | 0 | 9 | 5 | 22 | 4 | 0 | 2 | 20 | 110 | 16 | 10 | 1 | 0 | 0 | 0 | 8 |
| TCGA-AG-A025 | 4.164384 | 0 | 0 | 50 | 1 | 100 | 3 | 0 | 63 | 7 | 54 | 12 | 5 | 0 | 4 | 0 | 0 | 2 |
| TCGA-AG-3896 | 0.084932 | 0 | 1 | 297 | 6 | 30 | 0 | 0 | 9 | 8 | 398 | 28 | 2 | 0 | 0 | 0 | 0 | 12 |
| TCGA-A6-6137 | 2.257534 | 0 | 3 | 47 | 7 | 26 | 14 | 0 | 39 | 40 | 207 | 7 | 16 | 0 | 13 | 1 | 0 | 159 |
| TCGA-AA-3814 | 0 | 0 | 6 | 18 | 9 | 279 | 1 | 0 | 4 | 33 | 12 | 9 | 14 | 0 | 3 | 0 | 4 | 139 |
| TCGA-A6-5667 | 2.430137 | 0 | 2 | 2 | 44 | 27 | 79 | 11 | 5 | 15 | 166 | 41 | 5 | 0 | 8 | 3 | 4 | 10 |
| TCGA-AG-3594 | 0.167123 | 1 | 21 | 703 | 2 | 17 | 5 | 0 | 0 | 33 | 0 | 2 | 8 | 0 | 2 | 0 | 0 | 24 |
| TCGA-AA-A02W | 3.416438 | 0 | 44 | 23 | 8 | 351 | 9 | 0 | 8 | 22 | 862 | 21 | 18 | 0 | 27 | 0 | 1 | 17 |
| TCGA-DC-4749 | 2.087671 | 0 | 2 | 17 | 36 | 41 | 3 | 0 | 5 | 46 | 48 | 3 | 3 | 1 | 7 | 4 | 0 | 3 |
| TCGA-AY-6197 | 1.786301 | 0 | 0 | 33 | 12 | 39 | 4 | 0 | 134 | 71 | 15 | 96 | 57 | 2 | 9 | 0 | 0 | 25 |
| TCGA-EF-5831 | 0.347945 | 0 | 0 | 123 | 18 | 14 | 4 | 1 | 4 | 361 | 811 | 13 | 5 | 0 | 1 | 0 | 0 | 131 |
| TCGA-DC-6160 | 3.668493 | 0 | 0 | 35 | 11 | 359 | 1 | 0 | 5 | 9 | 2 | 19 | 15 | 3 | 13 | 2 | 0 | 51 |
| TCGA-DY-A1H8 | 2.717808 | 1 | 0 | 180 | 24 | 48 | 1 | 0 | 24 | 9 | 96 | 78 | 7 | 0 | 24 | 0 | 4 | 26 |
| TCGA-G5-6641 | 2.20274 | 0 | 0 | 391 | 13 | 108 | 4 | 0 | 3 | 43 | 27 | 7 | 18 | 8 | 2 | 2 | 1 | 16 |
| TCGA-AG-3878 | 0.082192 | 0 | 1 | 338 | 10 | 5 | 1 | 0 | 28 | 40 | 8 | 19 | 5 | 0 | 1 | 4 | 0 | 469 |
| TCGA-D5-6532 | 1.520548 | 0 | 2 | 77 | 9 | 39 | 9 | 0 | 17 | 15 | 3 | 7 | 4 | 3 | 19 | 1 | 0 | 6 |
| TCGA-EI-6917 | 1.454795 | 0 | 2 | 842 | 3 | 6 | 13 | 0 | 0 | 59 | 49 | 22 | 345 | 3 | 2 | 3 | 0 | 30 |
| TCGA-AG-3731 | 3.084932 | 0 | 0 | 371 | 3 | 31 | 64 | 3 | 19 | 117 | 154 | 30 | 7 | 8 | 35 | 6 | 3 | 1195 |
| TCGA-AZ-4308 | 9.106849 | 0 | 0 | 98 | 1 | 85 | 0 | 0 | 4 | 4 | 76 | 6 | 20 | 0 | 1 | 0 | 0 | 9 |
| TCGA-AF-3913 | 0.865753 | 1 | 2 | 0 | 16 | 20 | 0 | 0 | 2 | 28 | 212 | 11 | 6 | 1 | 8 | 1 | 0 | 1 |
| TCGA-AA-3522 | 3.087671 | 0 | 0 | 85 | 9 | 9 | 2 | 0 | 4 | 6 | 12 | 0 | 10 | 1 | 0 | 3 | 0 | 67 |
| TCGA-DY-A1DC | 3.446575 | 1 | 0 | 100 | 15 | 162 | 6 | 0 | 5 | 55 | 31 | 10 | 4 | 0 | 9 | 2 | 2 | 42 |
| TCGA-AA-3877 | 2.583562 | 0 | 0 | 582 | 14 | 27 | 7 | 1 | 1 | 11 | 1 | 16 | 5 | 1 | 4 | 0 | 2 | 8 |
| TCGA-D5-6540 | 1.345205 | 0 | 0 | 293 | 22 | 68 | 5 | 0 | 1 | 84 | 1 | 169 | 437 | 0 | 4 | 1 | 1 | 27 |
| TCGA-A6-2674 | 3.646575 | 0 | 14 | 35 | 4 | 64 | 23 | 1 | 79 | 91 | 1 | 23 | 30 | 0 | 3 | 1 | 2 | 114 |
| TCGA-AH-6897 | 2.20274 | 0 | 1 | 20 | 5 | 16 | 1 | 0 | 23 | 8 | 64 | 6 | 5 | 31 | 5 | 0 | 0 | 7 |
| TCGA-AA-3666 | 0.167123 | 1 | 2 | 33 | 2 | 206 | 1 | 0 | 5 | 11 | 8 | 4 | 140 | 1 | 0 | 0 | 0 | 7 |
| TCGA-AA-A01T | 2.753425 | 0 | 0 | 667 | 13 | 251 | 0 | 0 | 16 | 1 | 26 | 5 | 0 | 0 | 0 | 0 | 0 | 71 |
| TCGA-AA-3930 | 0.167123 | 1 | 2 | 24 | 7 | 24 | 3 | 0 | 27 | 5 | 1 | 20 | 59 | 3 | 0 | 0 | 0 | 10 |
| TCGA-CK-5915 | 0 | 0 | 0 | 62 | 11 | 21 | 1 | 0 | 0 | 11 | 45 | 14 | 10 | 0 | 2 | 0 | 1 | 1 |
| TCGA-F4-6570 | 0.515068 | 1 | 0 | 502 | 5 | 46 | 10 | 4 | 1 | 347 | 1 | 6 | 562 | 7 | 3 | 4 | 0 | 22 |
| TCGA-A6-2677 | 2.027397 | 1 | 2 | 15 | 4 | 2 | 25 | 24 | 95 | 93 | 67 | 11 | 12 | 15 | 53 | 4 | 4 | 31 |
| TCGA-AA-3867 | 2.00274 | 0 | 7 | 7 | 11 | 96 | 1 | 0 | 21 | 24 | 186 | 17 | 15 | 0 | 7 | 2 | 0 | 29 |
| TCGA-AA-A022 | 0 | 0 | 0 | 821 | 9 | 4 | 2 | 1 | 1 | 69 | 1 | 447 | 56 | 1 | 2 | 2 | 1 | 7 |
| TCGA-AA-3561 | 1.161644 | 0 | 3 | 8 | 7 | 32 | 0 | 0 | 4 | 15 | 1 | 3 | 1 | 0 | 0 | 0 | 0 | 0 |
| TCGA-DM-A28E | 9.994521 | 0 | 5 | 35 | 8 | 8 | 0 | 0 | 14 | 3 | 4 | 3 | 4 | 0 | 23 | 0 | 0 | 5 |
| TCGA-CM-5864 | 1.252055 | 0 | 4 | 119 | 6 | 28 | 5 | 0 | 43 | 9 | 61 | 20 | 57 | 0 | 4 | 0 | 0 | 14 |
| TCGA-D5-6922 | 0.843836 | 0 | 3 | 20 | 13 | 88 | 98 | 4 | 27 | 55 | 42 | 5 | 15 | 0 | 10 | 5 | 4 | 27 |
| TCGA-AG-3901 | 2.084932 | 0 | 31 | 20 | 0 | 20 | 19 | 2 | 3 | 86 | 25 | 18 | 8 | 7 | 1 | 4 | 0 | 14 |
| TCGA-AA-A00A | 3.169863 | 0 | 0 | 313 | 2 | 3 | 5 | 0 | 21 | 11 | 87 | 1 | 1 | 0 | 0 | 0 | 0 | 258 |
| TCGA-AY-A69D | 1.487671 | 0 | 1 | 245 | 15 | 4 | 37 | 0 | 110 | 50 | 7 | 3 | 3 | 20 | 4 | 1 | 0 | 236 |
| TCGA-NH-A5IV | 0 | 0 | 0 | 1 | 33 | 8 | 8 | 0 | 32 | 170 | 7 | 13 | 22 | 28 | 2 | 7 | 3 | 934 |
| TCGA-EI-6514 | 1.358904 | 0 | 22 | 3 | 22 | 35 | 32 | 9 | 2 | 30 | 4 | 12 | 5 | 1 | 8 | 0 | 0 | 2 |
| TCGA-A6-3808 | 2.778082 | 0 | 0 | 25 | 0 | 20 | 32 | 0 | 3 | 36 | 17 | 19 | 20 | 1 | 0 | 4 | 3 | 25 |
| TCGA-EI-6883 | 0.958904 | 0 | 4 | 23 | 49 | 12 | 12 | 1 | 5 | 39 | 1344 | 23 | 7 | 0 | 14 | 2 | 0 | 80 |
| TCGA-AA-A01Z | 3.084932 | 0 | 2 | 448 | 1 | 21 | 10 | 0 | 2 | 20 | 60 | 9 | 8 | 0 | 1 | 0 | 0 | 88 |
| TCGA-AG-3887 | 3.079452 | 0 | 1 | 20 | 3 | 7 | 12 | 0 | 4 | 37 | 11 | 19 | 0 | 1 | 9 | 1 | 0 | 6 |
| TCGA-A6-5659 | 2.536986 | 0 | 0 | 182 | 32 | 30 | 23 | 15 | 1 | 146 | 94 | 62 | 9 | 37 | 2 | 0 | 0 | 29 |
| TCGA-D5-5540 | 4.673973 | 0 | 2 | 47 | 13 | 42 | 8 | 0 | 5 | 23 | 41 | 2 | 0 | 0 | 4 | 3 | 0 | 0 |
| TCGA-CM-6172 | 0.917808 | 0 | 1 | 256 | 5 | 4 | 12 | 1 | 4 | 41 | 9 | 55 | 6 | 65 | 8 | 9 | 0 | 697 |
| TCGA-4N-A93T | 0.4 | 0 | 19 | 604 | 25 | 38 | 14 | 0 | 36 | 4 | 31 | 372 | 0 | 10 | 15 | 0 | 1 | 61 |
| TCGA-AZ-6599 | 0.564384 | 1 | 44 | 269 | 4 | 11 | 5 | 0 | 0 | 6 | 2 | 3 | 1 | 85 | 0 | 15 | 0 | 497 |
| TCGA-EI-6511 | 1.320548 | 0 | 3 | 79 | 125 | 64 | 8 | 1 | 43 | 148 | 47 | 3 | 21 | 1 | 3 | 1 | 0 | 80 |
| TCGA-AA-3489 | 0.586301 | 1 | 42 | 41 | 21 | 4 | 108 | 3 | 16 | 147 | 60 | 72 | 40 | 0 | 36 | 0 | 4 | 1956 |
| TCGA-AA-3972 | 4.249315 | 0 | 0 | 15 | 3 | 7 | 2 | 0 | 8 | 47 | 3 | 4 | 15 | 4 | 3 | 2 | 1 | 98 |
| TCGA-AY-4070 | 1.358904 | 1 | 0 | 31 | 19 | 713 | 6 | 0 | 119 | 140 | 117 | 150 | 89 | 22 | 52 | 1 | 8 | 35 |
| TCGA-A6-6782 | 1.690411 | 0 | 8 | 14 | 20 | 143 | 28 | 0 | 6 | 26 | 180 | 5 | 37 | 0 | 15 | 1 | 0 | 42 |
| TCGA-A6-A56B | 4.687671 | 1 | 13 | 260 | 3 | 0 | 7 | 0 | 6 | 29 | 20 | 126 | 5 | 15 | 2 | 0 | 0 | 0 |
| TCGA-G4-6304 | 4.468493 | 0 | 0 | 124 | 20 | 3 | 0 | 0 | 23 | 8 | 3 | 121 | 8 | 0 | 1 | 1 | 0 | 50 |
| TCGA-AA-A03J | 3.413699 | 0 | 0 | 63 | 5 | 52 | 2 | 0 | 8 | 7 | 14 | 27 | 5 | 0 | 4 | 2 | 0 | 2 |
| TCGA-DM-A1DB | 3.693151 | 1 | 33 | 863 | 29 | 187 | 9 | 0 | 30 | 12 | 96 | 72 | 27 | 0 | 68 | 1 | 0 | 86 |
| TCGA-G4-6294 | 2.350685 | 1 | 24 | 46 | 14 | 38 | 2 | 1 | 29 | 18 | 48 | 6 | 8 | 0 | 52 | 0 | 0 | 2 |
| TCGA-A6-6651 | 1.813699 | 0 | 6 | 3 | 18 | 28 | 393 | 19 | 8 | 76 | 41 | 41 | 41 | 13 | 14 | 4 | 4 | 564 |
| TCGA-AD-6895 | 2.090411 | 0 | 0 | 398 | 12 | 51 | 24 | 1 | 0 | 215 | 13 | 99 | 18 | 6 | 0 | 3 | 0 | 28 |
| TCGA-DM-A0XD | 2.035616 | 1 | 1 | 765 | 7 | 708 | 10 | 0 | 1 | 23 | 10 | 83 | 401 | 1 | 4 | 4 | 2 | 105 |
| TCGA-AG-3894 | 1.167123 | 0 | 1 | 26 | 0 | 118 | 1 | 0 | 9 | 43 | 26 | 11 | 4 | 0 | 0 | 0 | 0 | 4 |
| TCGA-D5-7000 | 0.854795 | 0 | 0 | 739 | 9 | 126 | 14 | 1 | 0 | 73 | 30 | 5 | 21 | 7 | 2 | 7 | 0 | 270 |
| TCGA-G4-6315 | 5.158904 | 0 | 1 | 9 | 9 | 7 | 2 | 0 | 10 | 19 | 14 | 25 | 9 | 0 | 17 | 1 | 1 | 105 |
| TCGA-AG-4022 | 3.835616 | 0 | 2 | 11 | 10 | 654 | 33 | 0 | 16 | 50 | 106 | 8 | 24 | 5 | 16 | 2 | 0 | 138 |
| TCGA-AA-3518 | 0.084932 | 0 | 0 | 480 | 10 | 3 | 0 | 0 | 0 | 71 | 0 | 18 | 5 | 5 | 0 | 0 | 0 | 14 |
| TCGA-NH-A8F7 | 1.487671 | 0 | 0 | 15 | 20 | 2 | 7 | 1 | 2 | 4 | 8 | 6 | 19 | 0 | 13 | 0 | 0 | 0 |
| TCGA-AA-3947 | 2.750685 | 0 | 0 | 3 | 4 | 9 | 0 | 0 | 0 | 10 | 0 | 1 | 1 | 1 | 0 | 1 | 0 | 0 |
| TCGA-A6-6780 | 1.676712 | 0 | 0 | 4 | 0 | 0 | 5 | 1 | 10 | 33 | 7 | 2 | 17 | 0 | 2 | 1 | 1 | 35 |
| TCGA-AA-A01C | 1.252055 | 0 | 1 | 24 | 13 | 36 | 1 | 0 | 3 | 4 | 155 | 22 | 2 | 1 | 1 | 1 | 0 | 2 |
| TCGA-AD-6963 | 2.284932 | 0 | 28 | 53 | 20 | 213 | 10 | 2 | 20 | 25 | 55 | 25 | 12 | 0 | 37 | 0 | 0 | 37 |
| TCGA-CI-6624 | 4.016438 | 0 | 0 | 65 | 10 | 37 | 19 | 1 | 21 | 37 | 798 | 5 | 4 | 30 | 2 | 0 | 0 | 168 |
| TCGA-AF-3400 | 2.873973 | 0 | 0 | 37 | 1 | 2 | 31 | 2 | 4 | 41 | 5 | 2 | 52 | 2 | 16 | 2 | 0 | 26 |
| TCGA-AA-3509 | 5.246575 | 0 | 4 | 24 | 5 | 206 | 3 | 0 | 59 | 17 | 16 | 2 | 41 | 3 | 1 | 0 | 0 | 10 |
| TCGA-A6-2671 | 3.646575 | 1 | 25 | 235 | 14 | 5 | 5 | 0 | 4 | 22 | 16 | 17 | 1 | 0 | 4 | 2 | 1 | 4 |
| TCGA-AG-A01W | 0 | 0 | 3 | 32 | 13 | 22 | 0 | 2 | 2 | 79 | 28 | 9 | 12 | 1 | 6 | 0 | 0 | 41 |
| TCGA-AG-3609 | 1.665753 | 0 | 0 | 38 | 15 | 5 | 2 | 0 | 6 | 27 | 5 | 28 | 11 | 0 | 5 | 0 | 0 | 17 |
| TCGA-G4-6317 | 3 | 0 | 0 | 21 | 15 | 132 | 14 | 0 | 15 | 4 | 511 | 2 | 7 | 1 | 3 | 1 | 0 | 1 |
| TCGA-AG-A036 | 9.758904 | 0 | 0 | 16 | 9 | 7 | 3 | 0 | 13 | 32 | 57 | 1 | 3 | 1 | 6 | 0 | 0 | 17 |
| TCGA-CM-4747 | 2.084932 | 0 | 25 | 454 | 6 | 13 | 2 | 0 | 1 | 9 | 0 | 14 | 2 | 1 | 0 | 0 | 0 | 42 |
| TCGA-AA-A010 | 2.915068 | 0 | 0 | 17 | 2 | 1 | 1 | 0 | 13 | 3 | 9 | 10 | 23 | 0 | 1 | 0 | 0 | 3 |
| TCGA-A6-5662 | 1.967123 | 0 | 19 | 305 | 10 | 38 | 30 | 1 | 4 | 323 | 799 | 131 | 2 | 6 | 67 | 4 | 0 | 3 |
| TCGA-AF-2693 | 3.164384 | 0 | 0 | 85 | 19 | 43 | 17 | 0 | 14 | 28 | 319 | 6 | 37 | 1 | 4 | 1 | 1 | 238 |
| TCGA-DM-A1D4 | 7.728767 | 1 | 0 | 1052 | 25 | 8 | 3 | 0 | 67 | 11 | 21 | 2 | 19 | 0 | 1 | 2 | 0 | 177 |
| TCGA-CA-5796 | 1.032877 | 0 | 5 | 19 | 1 | 2 | 2 | 0 | 20 | 10 | 10 | 3 | 15 | 2 | 0 | 0 | 0 | 13 |
| TCGA-AA-A00Q | 3.50137 | 0 | 20 | 143 | 5 | 24 | 7 | 1 | 0 | 3 | 38 | 5 | 7 | 6 | 1 | 1 | 0 | 227 |
| TCGA-AA-3544 | 1.167123 | 0 | 0 | 201 | 4 | 105 | 0 | 0 | 14 | 7 | 666 | 5 | 7 | 0 | 2 | 0 | 0 | 22 |
| TCGA-G4-6625 | 7.649315 | 0 | 27 | 118 | 5 | 2 | 28 | 1 | 28 | 40 | 12 | 38 | 8 | 3 | 6 | 1 | 2 | 424 |
| TCGA-AG-A02X | 3.416438 | 0 | 0 | 68 | 7 | 11 | 1 | 0 | 6 | 2 | 2 | 2 | 1 | 5 | 1 | 1 | 0 | 526 |
| TCGA-F5-6465 | 4.126027 | 0 | 2 | 42 | 8 | 213 | 29 | 5 | 28 | 59 | 40 | 31 | 15 | 0 | 7 | 5 | 0 | 82 |
| TCGA-G5-6572 | 3.923288 | 1 | 0 | 3 | 9 | 45 | 264 | 0 | 76 | 67 | 93 | 21 | 19 | 0 | 16 | 6 | 1 | 106 |
| TCGA-AG-3580 | 0.668493 | 0 | 0 | 16 | 1 | 17 | 2 | 0 | 7 | 12 | 2 | 1 | 3 | 0 | 4 | 0 | 0 | 4 |
| TCGA-AA-3812 | 2.920548 | 0 | 3 | 40 | 4 | 58 | 6 | 0 | 4 | 13 | 6 | 28 | 4 | 0 | 4 | 0 | 0 | 3 |
| TCGA-D5-6529 | 1.682192 | 0 | 0 | 62 | 9 | 295 | 20 | 1 | 22 | 53 | 17 | 141 | 34 | 6 | 8 | 11 | 0 | 82 |
| TCGA-CL-5918 | 0 | 0 | 45 | 21 | 21 | 130 | 3 | 0 | 4 | 10 | 10 | 14 | 3 | 6 | 23 | 0 | 0 | 4 |
| TCGA-A6-2681 | 3.8 | 0 | 0 | 48 | 3 | 32 | 23 | 0 | 19 | 12 | 41 | 11 | 10 | 0 | 7 | 3 | 1 | 29 |
| TCGA-AA-3543 | 0.082192 | 0 | 0 | 336 | 0 | 3 | 1 | 0 | 0 | 22 | 2 | 21 | 6 | 5 | 1 | 0 | 0 | 11 |
| TCGA-AG-3732 | 2.747945 | 0 | 9 | 23 | 1 | 22 | 15 | 2 | 11 | 6 | 26 | 0 | 6 | 0 | 13 | 0 | 1 | 1501 |
| TCGA-SS-A7HO | 5.010959 | 0 | 0 | 2 | 0 | 173 | 3 | 0 | 43 | 63 | 616 | 41 | 3 | 59 | 35 | 0 | 0 | 3 |
| TCGA-AA-3488 | 0.419178 | 1 | 11 | 9 | 18 | 168 | 2 | 0 | 17 | 12 | 62 | 2 | 4 | 0 | 5 | 0 | 1 | 0 |
| TCGA-AA-3525 | 0.00274 | 0 | 1 | 49 | 14 | 9 | 1 | 0 | 1 | 45 | 5 | 154 | 24 | 5 | 0 | 4 | 0 | 98 |
| TCGA-AA-3553 | 2 | 0 | 0 | 9 | 12 | 829 | 2 | 0 | 3 | 20 | 33 | 27 | 15 | 0 | 38 | 0 | 0 | 9 |
| TCGA-AY-A71X | 1.610959 | 0 | 2 | 471 | 11 | 18 | 2 | 0 | 10 | 39 | 20 | 19 | 187 | 63 | 1 | 0 | 2 | 236 |
| TCGA-CK-4951 | 5.846575 | 1 | 1 | 137 | 43 | 55 | 5 | 0 | 0 | 78 | 0 | 22 | 119 | 2 | 1 | 0 | 0 | 14 |
| TCGA-G4-6626 | 0.00274 | 1 | 8 | 34 | 26 | 113 | 1945 | 6 | 118 | 83 | 181 | 127 | 9 | 13 | 39 | 1 | 4 | 4484 |
| TCGA-DM-A1HA | 7.123288 | 0 | 0 | 30 | 7 | 13 | 9 | 0 | 5 | 76 | 3 | 362 | 47 | 19 | 1 | 0 | 0 | 3 |
| TCGA-D5-6928 | 0.969863 | 0 | 0 | 701 | 5 | 3 | 28 | 0 | 3 | 85 | 4 | 18 | 16 | 25 | 38 | 1 | 1 | 879 |
| TCGA-AF-2692 | 1.128767 | 0 | 8 | 42 | 5 | 31 | 9 | 0 | 4 | 12 | 7 | 15 | 38 | 0 | 5 | 0 | 0 | 10 |
| TCGA-G4-6307 | 4.586301 | 0 | 0 | 30 | 16 | 878 | 4 | 0 | 25 | 38 | 267 | 77 | 0 | 0 | 27 | 0 | 0 | 190 |
| TCGA-A6-4105 | 1.210959 | 1 | 2 | 650 | 35 | 8 | 14 | 0 | 2 | 189 | 13 | 87 | 14 | 2 | 3 | 2 | 0 | 107 |
| TCGA-CM-5349 | 2.506849 | 0 | 7 | 65 | 0 | 30 | 36 | 7 | 10 | 262 | 19 | 36 | 62 | 12 | 1 | 0 | 4 | 246 |
| TCGA-CA-5254 | 1.057534 | 0 | 0 | 520 | 1 | 65 | 0 | 0 | 16 | 583 | 1 | 63 | 873 | 1 | 5 | 4 | 0 | 533 |
| TCGA-A6-6138 | 1.876712 | 0 | 28 | 166 | 14 | 3 | 21 | 1 | 2 | 48 | 9 | 15 | 5 | 4 | 2 | 0 | 0 | 293 |
| TCGA-CM-6680 | 1.00274 | 0 | 30 | 152 | 21 | 12 | 14 | 1 | 42 | 93 | 66 | 86 | 38 | 113 | 3 | 7 | 1 | 133 |
| TCGA-NH-A6GA | 0.827397 | 1 | 0 | 126 | 118 | 1323 | 14 | 0 | 15 | 308 | 5 | 6 | 740 | 171 | 55 | 3 | 0 | 24 |
| TCGA-AG-4021 | 0.331507 | 1 | 1 | 685 | 29 | 1089 | 18 | 4 | 71 | 196 | 12001 | 81 | 34 | 28 | 71 | 12 | 0 | 167 |
| TCGA-AA-3713 | 1.586301 | 0 | 1 | 454 | 0 | 0 | 6 | 0 | 2 | 95 | 1 | 14 | 4 | 15 | 21 | 0 | 0 | 837 |
| TCGA-AG-3578 | 2.668493 | 0 | 4 | 254 | 1 | 50 | 2 | 0 | 58 | 42 | 4 | 13 | 100 | 0 | 2 | 4 | 1 | 123 |
| TCGA-F5-6813 | 1.638356 | 1 | 34 | 90 | 30 | 32 | 64 | 10 | 78 | 38 | 40 | 13 | 19 | 0 | 1 | 0 | 0 | 64 |
| TCGA-AY-6386 | 1.484932 | 0 | 0 | 72 | 26 | 26 | 8 | 0 | 0 | 10 | 7 | 63 | 22 | 11 | 1 | 2 | 0 | 19 |
| TCGA-AH-6544 | 3.213699 | 0 | 3 | 30 | 38 | 52 | 15 | 0 | 11 | 17 | 5 | 22 | 72 | 0 | 14 | 5 | 0 | 114 |
| TCGA-DY-A1DG | 4.290411 | 1 | 15 | 64 | 15 | 51 | 3 | 0 | 13 | 5 | 58 | 265 | 3 | 58 | 49 | 0 | 0 | 0 |
| TCGA-AF-6672 | 2.049315 | 0 | 2 | 75 | 4 | 74 | 7 | 0 | 0 | 97 | 451 | 18 | 351 | 4 | 12 | 8 | 0 | 151 |
| TCGA-CM-6676 | 0.923288 | 0 | 2 | 7 | 5 | 129 | 10 | 0 | 6 | 32 | 76 | 181 | 5 | 0 | 47 | 1 | 1 | 2 |
| TCGA-AA-3492 | 0.00274 | 1 | 0 | 136 | 1 | 104 | 5 | 3 | 0 | 7 | 0 | 7 | 12 | 0 | 0 | 0 | 0 | 29 |
| TCGA-EI-6510 | 1.523288 | 0 | 4 | 25 | 8 | 29 | 1 | 0 | 4 | 27 | 39 | 4 | 4 | 3 | 2 | 5 | 0 | 444 |
| TCGA-CK-6748 | 0.167123 | 0 | 27 | 10 | 11 | 83 | 49 | 1 | 7 | 204 | 227 | 48 | 13 | 0 | 9 | 9 | 1 | 2 |
| TCGA-AG-A02G | 3.246575 | 1 | 26 | 441 | 16 | 151 | 3 | 0 | 9 | 6 | 20 | 15 | 10 | 0 | 24 | 1 | 3 | 16 |
| TCGA-AA-A024 | 3.254795 | 1 | 27 | 100 | 1 | 14 | 0 | 0 | 3 | 10 | 28 | 2 | 69 | 0 | 0 | 3 | 0 | 0 |
| TCGA-AA-3977 | 2.084932 | 0 | 7 | 39 | 2 | 2 | 15 | 1 | 10 | 19 | 29 | 3 | 10 | 0 | 0 | 0 | 0 | 4 |
| TCGA-AG-A016 | 0.756164 | 0 | 11 | 1 | 1 | 31 | 0 | 0 | 31 | 2 | 34 | 6 | 2 | 0 | 4 | 0 | 2 | 0 |
| TCGA-AG-3582 | 3.00274 | 1 | 0 | 9 | 8 | 36 | 4 | 0 | 3 | 11 | 199 | 4 | 6 | 0 | 1 | 0 | 0 | 2 |
| TCGA-F4-6460 | 2.663014 | 1 | 4 | 40 | 27 | 428 | 148 | 9 | 26 | 47 | 1834 | 3 | 67 | 0 | 0 | 3 | 1 | 180 |
| TCGA-EI-6512 | 1.473973 | 0 | 0 | 24 | 14 | 28 | 4 | 2 | 109 | 77 | 27 | 11 | 69 | 1 | 7 | 0 | 0 | 39 |
| TCGA-AA-3941 | 2 | 0 | 0 | 191 | 5 | 442 | 3 | 0 | 0 | 126 | 29 | 10 | 247 | 4 | 0 | 1 | 0 | 301 |
| TCGA-DY-A1DF | 2.010959 | 1 | 3 | 97 | 27 | 143 | 7 | 6 | 56 | 16 | 1633 | 19 | 66 | 71 | 28 | 3 | 0 | 17 |
| TCGA-AZ-4682 | 1.863014 | 1 | 0 | 18 | 27 | 1160 | 6 | 0 | 13 | 26 | 2 | 9 | 9 | 2 | 4 | 3 | 2 | 0 |
| TCGA-A6-5661 | 2.794521 | 0 | 0 | 9 | 9 | 0 | 3 | 28 | 0 | 70 | 0 | 10 | 0 | 0 | 0 | 0 | 0 | 237 |
| TCGA-AA-3939 | 1.082192 | 0 | 17 | 26 | 0 | 84 | 1 | 2 | 18 | 18 | 8 | 6 | 3 | 7 | 0 | 1 | 0 | 12 |
| TCGA-DM-A28A | 2.205479 | 1 | 1 | 83 | 14 | 10 | 4 | 0 | 41 | 111 | 303 | 225 | 62 | 3 | 24 | 5 | 1 | 64 |
| TCGA-G4-6322 | 2.169863 | 0 | 0 | 404 | 12 | 7 | 7 | 0 | 0 | 318 | 17 | 46 | 8 | 0 | 2 | 0 | 1 | 617 |
| TCGA-AA-A02O | 0.076712 | 0 | 5 | 2412 | 10 | 33 | 2 | 0 | 0 | 22 | 17 | 28 | 22 | 9 | 0 | 0 | 0 | 53 |
| TCGA-AG-A011 | 3.084932 | 0 | 0 | 256 | 7 | 2 | 5 | 0 | 2 | 12 | 658 | 9 | 1 | 0 | 0 | 0 | 0 | 42 |
| TCGA-AZ-6600 | 1.008219 | 1 | 3 | 32 | 19 | 199 | 26 | 2 | 46 | 45 | 141 | 63 | 45 | 30 | 17 | 3 | 0 | 317 |
| TCGA-AG-A01L | 0 | 0 | 5 | 284 | 9 | 45 | 1 | 0 | 3 | 7 | 52 | 17 | 5 | 0 | 1 | 0 | 1 | 87 |
| TCGA-CA-5256 | 1.038356 | 0 | 4 | 0 | 10 | 65 | 2 | 0 | 8 | 13 | 79 | 2 | 0 | 0 | 2 | 2 | 0 | 5 |
| TCGA-DM-A1D0 | 10.88767 | 0 | 4 | 276 | 16 | 422 | 2 | 0 | 4 | 69 | 5 | 46 | 46 | 3 | 9 | 2 | 1 | 3 |
| TCGA-AA-A01F | 2.668493 | 0 | 10 | 11 | 16 | 21 | 0 | 0 | 6 | 8 | 0 | 11 | 121 | 1 | 10 | 0 | 0 | 0 |
| TCGA-AA-3852 | 0 | 1 | 0 | 242 | 1 | 2 | 1 | 0 | 2 | 17 | 8 | 29 | 14 | 11 | 3 | 1 | 0 | 639 |
| TCGA-CK-6746 | 0 | 0 | 0 | 161 | 33 | 4 | 1 | 0 | 1 | 6 | 0 | 517 | 67 | 3 | 8 | 0 | 0 | 414 |
| TCGA-AA-3980 | 0.663014 | 0 | 0 | 32 | 0 | 2 | 5 | 0 | 3 | 24 | 33 | 88 | 18 | 0 | 6 | 0 | 0 | 15 |
| TCGA-5M-AAT4 | 0.134247 | 1 | 0 | 278 | 27 | 63 | 7 | 0 | 11 | 60 | 19 | 184 | 66 | 0 | 58 | 2 | 71 | 235 |
| TCGA-AA-3976 | 2.167123 | 0 | 0 | 41 | 1 | 39 | 5 | 0 | 1 | 14 | 66 | 5 | 3 | 0 | 0 | 0 | 0 | 0 |
| TCGA-AG-3999 | 2.336986 | 0 | 23 | 43 | 7 | 42 | 5 | 0 | 14 | 4 | 104 | 37 | 47 | 1 | 2 | 1 | 0 | 8 |
| TCGA-CM-6162 | 1 | 0 | 1 | 6 | 1 | 12 | 83 | 3 | 0 | 130 | 23 | 4 | 98 | 1 | 8 | 1 | 2 | 102 |
| TCGA-DM-A282 | 11.59726 | 0 | 31 | 66 | 16 | 61 | 5 | 0 | 27 | 31 | 47 | 17 | 2 | 0 | 5 | 3 | 0 | 2 |
| TCGA-AA-3982 | 2.252055 | 0 | 2 | 10 | 1 | 8 | 5 | 4 | 2 | 26 | 35 | 15 | 10 | 2 | 3 | 0 | 2 | 19 |
| TCGA-G4-6320 | 2.20274 | 0 | 0 | 157 | 30 | 102 | 6 | 0 | 0 | 71 | 1 | 123 | 1 | 21 | 15 | 2 | 0 | 381 |
| TCGA-F5-6811 | 2.682192 | 0 | 21 | 2 | 7 | 187 | 10 | 2 | 35 | 143 | 24 | 24 | 15 | 1 | 6 | 3 | 0 | 18 |
| TCGA-G4-6310 | 5.30137 | 0 | 3 | 12 | 16 | 27 | 165 | 0 | 9 | 15 | 85 | 3 | 1 | 1 | 8 | 1 | 7 | 1 |
| TCGA-CM-4743 | 1.920548 | 0 | 0 | 132 | 9 | 213 | 18 | 0 | 1 | 44 | 1 | 26 | 142 | 2 | 0 | 7 | 0 | 6 |
| TCGA-AA-3848 | 0.838356 | 1 | 1 | 92 | 15 | 7 | 4 | 0 | 13 | 4 | 1298 | 164 | 4 | 0 | 1 | 0 | 0 | 9 |
| TCGA-AF-6655 | 1.668493 | 0 | 0 | 0 | 13 | 18 | 26 | 0 | 14 | 45 | 23 | 3 | 5 | 1 | 4 | 9 | 0 | 7 |
| TCGA-F4-6807 | 3.586301 | 0 | 10 | 10 | 9 | 25 | 113 | 1 | 0 | 68 | 16 | 7 | 46 | 1 | 1 | 1 | 1 | 166 |
| TCGA-A6-3809 | 2.728767 | 0 | 0 | 6 | 7 | 67 | 0 | 9 | 0 | 92 | 8 | 67 | 139 | 5 | 5 | 0 | 4 | 11 |
| TCGA-AG-3882 | 1.665753 | 0 | 0 | 56 | 15 | 24 | 109 | 0 | 1 | 2 | 2 | 12 | 3 | 0 | 7 | 0 | 0 | 138 |
| TCGA-AA-3715 | 1.586301 | 1 | 0 | 197 | 15 | 7 | 2 | 1 | 0 | 158 | 1 | 253 | 216 | 1 | 10 | 12 | 0 | 98 |
| TCGA-QG-A5YV | 3.564384 | 0 | 0 | 8 | 34 | 29 | 1 | 0 | 16 | 17 | 8 | 12 | 5 | 0 | 4 | 2 | 6 | 42 |
| TCGA-DC-5337 | 2.169863 | 0 | 0 | 0 | 21 | 38 | 10 | 0 | 10 | 14 | 9 | 3 | 0 | 0 | 21 | 3 | 0 | 6 |
| TCGA-DY-A1DE | 10.7726 | 0 | 23 | 41 | 4 | 20 | 52 | 6 | 36 | 62 | 49 | 78 | 14 | 3 | 10 | 0 | 0 | 7 |
| TCGA-AG-A01J | 0.084932 | 0 | 1 | 15 | 4 | 21 | 1 | 0 | 3 | 3 | 17 | 20 | 2 | 0 | 3 | 0 | 0 | 6 |
| TCGA-AA-3994 | 2.252055 | 0 | 0 | 192 | 11 | 67 | 17 | 0 | 1 | 25 | 7 | 14 | 16 | 8 | 2 | 5 | 0 | 358 |
| TCGA-AA-A00K | 1.50411 | 0 | 2 | 68 | 2 | 14 | 3 | 2 | 26 | 6 | 67 | 62 | 8 | 0 | 3 | 0 | 1 | 4 |
| TCGA-AG-A032 | 3.169863 | 0 | 12 | 26 | 18 | 3 | 13 | 3 | 10 | 14 | 57 | 6 | 3 | 2 | 4 | 0 | 1 | 17 |
| TCGA-AA-3866 | 1.419178 | 0 | 0 | 8 | 6 | 7 | 16 | 0 | 1 | 25 | 7 | 1 | 8 | 0 | 2 | 0 | 0 | 13 |
| TCGA-F4-6463 | 2.978082 | 0 | 0 | 41 | 2 | 12 | 81 | 4 | 65 | 104 | 224 | 22 | 51 | 0 | 2 | 9 | 0 | 24 |
| TCGA-AG-3727 | 0.082192 | 0 | 0 | 84 | 0 | 52 | 0 | 0 | 9 | 1 | 15 | 3 | 2 | 0 | 0 | 0 | 1 | 3 |
| TCGA-AA-3833 | 1.328767 | 0 | 2 | 21 | 9 | 35 | 18 | 1 | 0 | 57 | 0 | 150 | 69 | 0 | 0 | 1 | 0 | 197 |
| TCGA-D5-5541 | 4.660274 | 0 | 2 | 7 | 17 | 29 | 15 | 0 | 39 | 47 | 24 | 93 | 25 | 7 | 16 | 2 | 0 | 6 |
| TCGA-AA-3531 | 2.835616 | 0 | 4 | 23 | 4 | 8 | 1 | 0 | 13 | 3 | 51 | 5 | 0 | 0 | 0 | 0 | 0 | 0 |
| TCGA-AA-A02K | 1.167123 | 1 | 0 | 1458 | 51 | 986 | 3 | 2 | 16 | 17 | 120 | 81 | 11 | 117 | 140 | 1 | 0 | 4 |
| TCGA-CM-4750 | 0.668493 | 0 | 0 | 1 | 36 | 90 | 1 | 1 | 1 | 5 | 1 | 16 | 6 | 0 | 6 | 0 | 0 | 10 |
| TCGA-DC-6156 | 2.583562 | 0 | 0 | 65 | 18 | 525 | 828 | 1 | 1 | 32 | 44 | 10 | 162 | 2 | 15 | 10 | 0 | 1127 |
| TCGA-AG-3598 | 4.169863 | 0 | 1 | 15 | 2 | 4 | 1 | 0 | 13 | 7 | 8 | 13 | 0 | 0 | 1 | 0 | 0 | 46 |
| TCGA-F5-6464 | 0.830137 | 1 | 2 | 14 | 14 | 216 | 243 | 25 | 91 | 92 | 2 | 1 | 1219 | 0 | 6 | 11 | 1 | 42 |
| TCGA-A6-A566 | 2.076712 | 1 | 1 | 120 | 2 | 20 | 54 | 0 | 1 | 77 | 6 | 4 | 61 | 7 | 7 | 31 | 0 | 8 |
| TCGA-AG-3601 | 0 | 0 | 16 | 5 | 6 | 30 | 8 | 0 | 16 | 7 | 23 | 16 | 12 | 1 | 2 | 3 | 0 | 6 |
| TCGA-AG-3890 | 1.419178 | 0 | 2 | 86 | 16 | 33 | 4 | 0 | 18 | 10 | 0 | 60 | 9 | 0 | 13 | 0 | 1 | 42 |
| TCGA-D5-5538 | 4.550685 | 1 | 9 | 222 | 5 | 360 | 6 | 0 | 58 | 34 | 5 | 28 | 28 | 22 | 5 | 4 | 1 | 95 |
| TCGA-AF-3911 | 3.145205 | 0 | 14 | 50 | 22 | 1368 | 59 | 5 | 34 | 39 | 394 | 0 | 51 | 2 | 9 | 5 | 3 | 11 |
| TCGA-F4-6704 | 0.128767 | 0 | 9 | 158 | 12 | 12 | 101 | 15 | 8 | 627 | 16 | 13 | 22 | 58 | 0 | 7 | 4 | 20 |
| TCGA-AA-A00N | 0.334247 | 1 | 19 | 51 | 12 | 3 | 14 | 0 | 7 | 42 | 12 | 18 | 24 | 7 | 2 | 3 | 0 | 18 |
| TCGA-AA-3845 | 0 | 1 | 0 | 216 | 8 | 1 | 4 | 1 | 0 | 2088 | 0 | 144 | 37 | 0 | 1 | 2 | 0 | 30 |
| TCGA-D5-6530 | 1.70137 | 0 | 1 | 36 | 13 | 19 | 3 | 0 | 7 | 12 | 3 | 44 | 6 | 8 | 2 | 0 | 0 | 73 |
| TCGA-G5-6235 | 4.646575 | 0 | 2 | 9 | 6 | 41 | 4 | 0 | 14 | 12 | 1 | 13 | 4 | 11 | 34 | 0 | 0 | 113 |
| TCGA-AG-3591 | 2.835616 | 0 | 0 | 140 | 9 | 99 | 6 | 0 | 1 | 44 | 19 | 20 | 43 | 0 | 5 | 15 | 2 | 16 |
| TCGA-AA-A01K | 2.583562 | 0 | 0 | 105 | 2 | 13 | 5 | 2 | 8 | 28 | 16 | 8 | 54 | 0 | 10 | 4 | 0 | 4 |
| TCGA-EI-6506 | 1.712329 | 0 | 3 | 102 | 10 | 1 | 12 | 3 | 25 | 15 | 4 | 16 | 10 | 0 | 23 | 1 | 0 | 39 |
| TCGA-AY-4071 | 0.079452 | 1 | 7 | 55 | 4 | 12 | 13 | 0 | 4 | 27 | 3 | 1 | 2 | 1 | 3 | 0 | 0 | 28 |
| TCGA-AA-3660 | 6.506849 | 0 | 0 | 20 | 6 | 201 | 3 | 0 | 1 | 29 | 24 | 121 | 11 | 3 | 2 | 5 | 7 | 32 |
| TCGA-CM-4746 | 3.084932 | 0 | 2 | 1 | 12 | 5 | 4 | 0 | 0 | 7 | 22 | 8 | 7 | 2 | 0 | 0 | 0 | 2 |
| TCGA-G4-6295 | 0.69589 | 0 | 3 | 13 | 3 | 42 | 7 | 0 | 28 | 12 | 39 | 20 | 4 | 0 | 1 | 1 | 2 | 236 |
| TCGA-AA-3502 | 2.917808 | 0 | 14 | 85 | 2 | 7 | 0 | 0 | 2 | 6 | 7 | 13 | 13 | 0 | 0 | 0 | 0 | 169 |
| TCGA-AA-3685 | 3.087671 | 0 | 18 | 9 | 40 | 103 | 5 | 0 | 6 | 20 | 1 | 17 | 6 | 0 | 6 | 3 | 0 | 21 |
| TCGA-AG-A002 | 1.747945 | 0 | 28 | 164 | 2 | 3 | 40 | 0 | 12 | 230 | 2 | 99 | 253 | 0 | 12 | 1 | 2 | 0 |
| TCGA-CM-5863 | 1.252055 | 0 | 3 | 14 | 7 | 148 | 6 | 0 | 10 | 59 | 10 | 10 | 201 | 0 | 1 | 2 | 0 | 18 |
| TCGA-AA-3527 | 0 | 0 | 0 | 99 | 0 | 81 | 1 | 0 | 0 | 9 | 19 | 22 | 4 | 0 | 3 | 0 | 0 | 21 |
| TCGA-CM-6165 | 1.336986 | 0 | 1 | 10 | 5 | 4 | 14 | 6 | 16 | 58 | 25 | 2 | 9 | 0 | 1 | 0 | 0 | 41 |
| TCGA-CM-5861 | 1.252055 | 0 | 0 | 72 | 5 | 43 | 4 | 0 | 0 | 280 | 1 | 134 | 43 | 12 | 5 | 4 | 0 | 26 |
| TCGA-AF-A56K | 7.219178 | 0 | 9 | 11 | 14 | 32 | 36 | 0 | 21 | 51 | 42 | 26 | 13 | 2 | 18 | 2 | 4 | 21 |
| TCGA-CI-6623 | 3.953425 | 0 | 0 | 3 | 22 | 117 | 10 | 0 | 6 | 57 | 27 | 4 | 10 | 0 | 5 | 0 | 0 | 4 |
| TCGA-CM-4752 | 1.084932 | 0 | 3 | 16 | 9 | 35 | 8 | 0 | 13 | 12 | 27 | 26 | 3 | 2 | 4 | 1 | 0 | 11 |
| TCGA-A6-6652 | 2.057534 | 0 | 15 | 23 | 29 | 105 | 7 | 0 | 2 | 11 | 0 | 18 | 19 | 10 | 28 | 1 | 1 | 6 |
| TCGA-AA-3526 | 1.589041 | 0 | 16 | 8 | 31 | 41 | 7 | 0 | 14 | 59 | 14 | 188 | 14 | 4 | 9 | 5 | 0 | 11 |
| TCGA-A6-2682 | 1.161644 | 1 | 7 | 122 | 2 | 67 | 0 | 0 | 8 | 19 | 25 | 34 | 8 | 2 | 7 | 0 | 0 | 0 |
| TCGA-A6-3807 | 2.887671 | 0 | 7 | 1 | 1 | 14 | 7 | 0 | 2 | 9 | 28 | 13 | 0 | 1 | 15 | 1 | 3 | 7 |
| TCGA-AD-6548 | 1.780822 | 0 | 0 | 406 | 9 | 170 | 29 | 0 | 20 | 44 | 38 | 37 | 79 | 32 | 13 | 3 | 0 | 74 |
| TCGA-AF-6136 | 2.030137 | 0 | 5 | 13 | 10 | 33 | 19 | 0 | 16 | 53 | 14 | 5 | 145 | 0 | 0 | 2 | 0 | 67 |
| TCGA-AA-3663 | 0.580822 | 0 | 0 | 89 | 11 | 44 | 5 | 0 | 0 | 130 | 4 | 16 | 63 | 7 | 9 | 1 | 0 | 4 |
| TCGA-CA-6717 | 1.063014 | 0 | 38 | 10 | 3 | 16 | 78 | 3 | 47 | 261 | 65 | 7 | 66 | 6 | 5 | 2 | 1 | 567 |
| TCGA-DC-6682 | 2.087671 | 0 | 0 | 128 | 15 | 4 | 13 | 0 | 131 | 195 | 14 | 4 | 27 | 0 | 28 | 1 | 0 | 50 |
| TCGA-G4-6588 | 2.180822 | 0 | 0 | 156 | 30 | 14 | 6 | 0 | 0 | 168 | 2 | 30 | 20 | 3 | 5 | 6 | 0 | 36 |
| TCGA-D5-6932 | 0.947945 | 0 | 6 | 40 | 13 | 28 | 6 | 0 | 43 | 48 | 184 | 39 | 9 | 7 | 38 | 1 | 0 | 8 |
| TCGA-AZ-4614 | 0.471233 | 1 | 0 | 320 | 38 | 27 | 1 | 0 | 1 | 265 | 5 | 11 | 131 | 0 | 8 | 0 | 3 | 7 |
| TCGA-QG-A5YW | 2.454795 | 0 | 10 | 348 | 15 | 23 | 7 | 6 | 6 | 63 | 58 | 493 | 10 | 5 | 121 | 3 | 1 | 72 |
| TCGA-D5-6927 | 0.786301 | 0 | 0 | 6 | 13 | 20 | 19 | 2 | 5 | 39 | 2 | 19 | 44 | 1 | 7 | 3 | 0 | 13 |
| TCGA-A6-2672 | 3.887671 | 0 | 2 | 178 | 5 | 8 | 11 | 0 | 0 | 11 | 1 | 4 | 12 | 0 | 1 | 0 | 1 | 15 |
| TCGA-AA-3560 | 1.665753 | 0 | 0 | 3 | 2 | 52 | 13 | 0 | 10 | 29 | 63 | 4 | 1 | 1 | 1 | 3 | 0 | 8 |
| TCGA-AA-3530 | 1.589041 | 0 | 2 | 8 | 3 | 9 | 2 | 0 | 4 | 17 | 5 | 8 | 7 | 0 | 2 | 0 | 0 | 49 |
| TCGA-AY-A8YK | 1.569863 | 0 | 8 | 79 | 20 | 63 | 7 | 0 | 8 | 18 | 81 | 7 | 3 | 0 | 46 | 0 | 0 | 45 |
| TCGA-CK-4947 | 1.463014 | 0 | 0 | 10 | 1 | 177 | 7 | 0 | 3 | 32 | 5 | 3 | 5 | 0 | 3 | 1 | 0 | 136 |
| TCGA-AZ-4323 | 0.117808 | 1 | 179 | 602 | 14 | 32 | 2248 | 13 | 18 | 305 | 103 | 692 | 134 | 20 | 60 | 16 | 5 | 4326 |
| TCGA-F5-6861 | 3.178082 | 0 | 16 | 21 | 5 | 15 | 10 | 2 | 121 | 22 | 115 | 10 | 40 | 0 | 4 | 0 | 1 | 12 |
| TCGA-DC-6681 | 2.164384 | 0 | 6 | 16 | 9 | 9 | 86 | 9 | 38 | 32 | 77 | 69 | 5 | 4 | 18 | 2 | 0 | 16 |
| TCGA-AG-3608 | 1.328767 | 0 | 0 | 48 | 29 | 6 | 3 | 0 | 3 | 25 | 29 | 19 | 3 | 0 | 0 | 3 | 0 | 15 |
| TCGA-DM-A285 | 0.490411 | 1 | 3 | 39 | 13 | 341 | 14 | 1 | 637 | 329 | 335 | 112 | 501 | 29 | 34 | 4 | 2 | 32 |
| TCGA-AA-3854 | 3.00274 | 0 | 0 | 48 | 5 | 4 | 2 | 0 | 54 | 17 | 1 | 13 | 136 | 0 | 1 | 1 | 0 | 162 |
| TCGA-3L-AA1B | 1.30137 | 0 | 38 | 2 | 6 | 80 | 39 | 3 | 36 | 57 | 76 | 90 | 11 | 1 | 2 | 6 | 0 | 133 |
| TCGA-DM-A1D8 | 1.049315 | 1 | 5 | 2418 | 33 | 25 | 7 | 0 | 111 | 56 | 3 | 56 | 6 | 1 | 6 | 4 | 3 | 4 |
| TCGA-D5-6926 | 0.753425 | 0 | 3 | 14 | 17 | 45 | 15 | 1 | 31 | 46 | 491 | 4 | 9 | 0 | 2 | 9 | 0 | 11 |
| TCGA-AA-3710 | 2.249315 | 0 | 0 | 99 | 5 | 99 | 2 | 0 | 2 | 40 | 1 | 4 | 87 | 0 | 2 | 0 | 0 | 12 |
| TCGA-F4-6856 | 2.942466 | 0 | 5 | 12 | 7 | 3 | 6 | 0 | 45 | 7 | 9 | 36 | 51 | 7 | 1 | 0 | 0 | 34 |
| TCGA-AA-3831 | 1.49863 | 0 | 2 | 34 | 7 | 28 | 2 | 1 | 12 | 14 | 45 | 42 | 2 | 3 | 1 | 0 | 0 | 9 |
| TCGA-AG-3600 | 0.50411 | 0 | 2 | 304 | 9 | 39 | 2 | 0 | 2 | 3 | 9 | 8 | 179 | 5 | 1 | 0 | 0 | 760 |
| TCGA-G4-6299 | 6.213699 | 0 | 0 | 895 | 28 | 21 | 3 | 3 | 1 | 321 | 0 | 87 | 46 | 6 | 33 | 14 | 0 | 45 |
| TCGA-CM-4751 | 2.252055 | 0 | 0 | 90 | 5 | 231 | 12 | 1 | 2 | 40 | 2 | 19 | 212 | 6 | 1 | 0 | 0 | 248 |
| TCGA-4T-AA8H | 1.054795 | 0 | 0 | 531 | 6 | 30 | 1 | 0 | 20 | 10 | 30 | 51 | 3 | 14 | 4 | 1 | 0 | 6 |
| TCGA-AG-A020 | 0.084932 | 0 | 4 | 14 | 14 | 8 | 2 | 0 | 4 | 69 | 13 | 1 | 8 | 2 | 5 | 0 | 0 | 20 |
| TCGA-CA-6715 | 1.049315 | 0 | 7 | 3 | 23 | 27 | 20 | 0 | 109 | 29 | 41 | 8 | 75 | 5 | 11 | 1 | 1 | 1 |
| TCGA-D5-6535 | 1.260274 | 0 | 6 | 39 | 10 | 45 | 2 | 0 | 9 | 66 | 7 | 19 | 50 | 3 | 1 | 1 | 0 | 158 |
| TCGA-DM-A280 | 0.646575 | 1 | 26 | 5 | 8 | 2145 | 3 | 0 | 8 | 26 | 22 | 3 | 104 | 1 | 3 | 0 | 0 | 0 |
| TCGA-F5-6814 | 3.09863 | 0 | 6 | 436 | 3 | 11 | 37 | 13 | 210 | 61 | 74 | 144 | 24 | 1 | 1 | 1 | 1 | 8 |
| TCGA-CK-4952 | 1.30137 | 0 | 0 | 686 | 6 | 48 | 7 | 0 | 1 | 17 | 1 | 7 | 38 | 29 | 5 | 15 | 0 | 657 |
| TCGA-AG-3728 | 2.49863 | 0 | 0 | 10 | 32 | 99 | 11 | 1 | 8 | 8 | 57 | 116 | 6 | 1 | 10 | 4 | 1 | 94 |
| TCGA-D5-6534 | 3.605479 | 0 | 1 | 200 | 3 | 244 | 58 | 5 | 1 | 143 | 17 | 10 | 144 | 0 | 3 | 4 | 2 | 206 |
| TCGA-AA-3675 | 3.920548 | 0 | 25 | 23 | 19 | 3 | 5 | 0 | 0 | 30 | 7 | 21 | 14 | 0 | 4 | 1 | 1 | 2 |
| TCGA-EF-5830 | 0.290411 | 0 | 0 | 27 | 1 | 735 | 1 | 0 | 10 | 180 | 2474 | 38 | 42 | 85 | 6 | 2 | 0 | 10 |
| TCGA-F4-6703 | 3.989041 | 0 | 0 | 12 | 2 | 105 | 50 | 0 | 4 | 220 | 0 | 114 | 120 | 2 | 10 | 3 | 1 | 61 |
| TCGA-AG-3586 | 0.084932 | 0 | 0 | 19 | 1 | 640 | 3 | 0 | 66 | 13 | 30 | 8 | 20 | 0 | 11 | 0 | 0 | 5 |
| TCGA-NH-A6GC | 1.065753 | 0 | 34 | 13 | 2 | 11 | 5 | 1 | 6 | 15 | 104 | 22 | 26 | 8 | 2 | 3 | 0 | 8 |
| TCGA-AA-3552 | 1.084932 | 1 | 9 | 16 | 12 | 246 | 2 | 0 | 4 | 11 | 162 | 33 | 8 | 0 | 1 | 0 | 12 | 36 |
| TCGA-AA-3538 | 2.167123 | 0 | 2 | 16 | 10 | 302 | 2 | 0 | 10 | 29 | 31 | 5 | 11 | 0 | 9 | 0 | 0 | 14 |
| TCGA-AA-3851 | 2.756164 | 0 | 7 | 273 | 11 | 70 | 3 | 0 | 0 | 125 | 40 | 4 | 57 | 0 | 0 | 4 | 3 | 16 |
| TCGA-CM-6679 | 0.838356 | 0 | 0 | 5 | 50 | 20 | 47 | 1 | 17 | 43 | 32 | 4 | 14 | 0 | 7 | 6 | 1 | 82 |
| TCGA-G4-6628 | 6.641096 | 0 | 0 | 78 | 43 | 11 | 32 | 0 | 0 | 9 | 0 | 82 | 14 | 0 | 13 | 0 | 0 | 75 |
| TCGA-AG-4015 | 0 | 0 | 2 | 25 | 19 | 7 | 0 | 0 | 7 | 8 | 80 | 10 | 5 | 9 | 6 | 0 | 0 | 11 |
| TCGA-CM-4748 | 2.169863 | 0 | 7 | 12 | 3 | 7 | 3 | 1 | 2 | 5 | 16 | 11 | 6 | 0 | 4 | 0 | 0 | 14 |
| TCGA-CA-6716 | 1.016438 | 0 | 0 | 3 | 13 | 16 | 61 | 0 | 0 | 148 | 16 | 20 | 14 | 0 | 1 | 0 | 0 | 444 |
| TCGA-G4-6306 | 3.723288 | 0 | 9 | 13 | 17 | 359 | 1 | 0 | 10 | 4 | 100 | 4 | 3 | 6 | 5 | 0 | 0 | 21 |
| TCGA-AA-3979 | 2 | 0 | 2 | 5 | 0 | 23 | 1 | 0 | 4 | 11 | 12 | 4 | 8 | 1 | 2 | 2 | 0 | 26 |
| TCGA-F5-6571 | 3.528767 | 0 | 4 | 60 | 9 | 10 | 18 | 0 | 23 | 80 | 147 | 4 | 49 | 0 | 14 | 3 | 1 | 25 |
| TCGA-AA-3692 | 3 | 1 | 5 | 8 | 3 | 28 | 3 | 0 | 10 | 23 | 128 | 6 | 10 | 34 | 4 | 1 | 0 | 16 |
| TCGA-DC-6683 | 2.087671 | 0 | 3 | 16 | 7 | 229 | 30 | 4 | 47 | 51 | 675 | 1 | 10 | 0 | 5 | 2 | 0 | 64 |
| TCGA-AY-A54L | 1.438356 | 0 | 3 | 209 | 17 | 131 | 9 | 1 | 41 | 40 | 1235 | 77 | 49 | 1 | 2 | 2 | 0 | 46 |
| TCGA-DM-A28H | 9.756164 | 0 | 0 | 57 | 5 | 89 | 1 | 0 | 0 | 28 | 235 | 14 | 3 | 1 | 10 | 0 | 1 | 50 |
| TCGA-AA-3664 | 4.50137 | 0 | 0 | 304 | 1 | 13 | 2 | 0 | 0 | 76 | 9 | 16 | 134 | 3 | 2 | 0 | 0 | 35 |
| TCGA-A6-5656 | 2.742466 | 0 | 2 | 14 | 8 | 33 | 11 | 5 | 6 | 42 | 18 | 22 | 10 | 49 | 16 | 2 | 2 | 201 |
| TCGA-G4-6311 | 3.284932 | 0 | 0 | 13 | 13 | 268 | 12 | 0 | 10 | 43 | 7 | 8 | 46 | 2 | 13 | 13 | 0 | 562 |
| TCGA-AA-A00R | 0.082192 | 0 | 1 | 763 | 41 | 17 | 1 | 0 | 2 | 27 | 2 | 61 | 1 | 0 | 4 | 0 | 0 | 35 |
| TCGA-QG-A5Z2 | 2.608219 | 0 | 1 | 63 | 19 | 7 | 3 | 0 | 8 | 80 | 5 | 59 | 6 | 16 | 1 | 5 | 1 | 2224 |
| TCGA-F4-6461 | 0.926027 | 1 | 0 | 276 | 7 | 0 | 18 | 0 | 7 | 155 | 4 | 118 | 70 | 4 | 0 | 2 | 1 | 1290 |
| TCGA-AA-A00D | 1.583562 | 0 | 0 | 139 | 4 | 83 | 4 | 0 | 0 | 66 | 472 | 29 | 29 | 21 | 10 | 0 | 0 | 190 |
| TCGA-AY-6196 | 0.016438 | 0 | 1 | 4 | 4 | 20 | 36 | 22 | 3 | 61 | 1 | 13 | 305 | 47 | 3 | 23 | 1 | 687 |
| TCGA-A6-4107 | 2.70411 | 0 | 0 | 389 | 20 | 122 | 1 | 0 | 0 | 14 | 51 | 19 | 28 | 7 | 1 | 1 | 0 | 22 |
| TCGA-DC-6155 | 1.164384 | 0 | 0 | 18 | 32 | 165 | 3 | 0 | 4 | 3 | 11 | 2 | 3 | 0 | 10 | 0 | 0 | 32 |
| TCGA-AG-3881 | 1.586301 | 0 | 0 | 88 | 7 | 10 | 41 | 0 | 19 | 7 | 23 | 31 | 21 | 0 | 7 | 1 | 0 | 19 |
| TCGA-AG-A023 | 4.331507 | 1 | 6 | 205 | 3 | 108 | 8 | 0 | 20 | 14 | 9 | 13 | 9 | 1 | 26 | 1 | 0 | 89 |
| TCGA-AA-3870 | 2.49863 | 0 | 0 | 224 | 17 | 1089 | 9 | 0 | 8 | 13 | 17 | 182 | 260 | 1 | 0 | 0 | 0 | 86 |
| TCGA-AZ-6608 | 0.161644 | 1 | 0 | 47 | 31 | 136 | 0 | 0 | 39 | 4 | 164 | 45 | 13 | 1 | 5 | 2 | 1 | 2 |
| TCGA-AG-3726 | 0.665753 | 0 | 0 | 83 | 2 | 14 | 0 | 0 | 4 | 5 | 19 | 19 | 2 | 0 | 8 | 0 | 0 | 43 |
| TCGA-AH-6903 | 1.621918 | 0 | 12 | 17 | 13 | 35 | 10 | 0 | 45 | 38 | 81 | 237 | 98 | 19 | 8 | 0 | 1 | 29 |
| TCGA-NH-A50U | 0.915068 | 1 | 14 | 151 | 42 | 663 | 5 | 0 | 196 | 80 | 258 | 901 | 14 | 74 | 3 | 4 | 0 | 1 |
| TCGA-QL-A97D | 1.824658 | 0 | 16 | 17 | 12 | 15 | 3 | 1 | 7 | 17 | 3 | 2 | 5 | 10 | 2 | 0 | 1 | 104 |
| TCGA-A6-6653 | 2.032877 | 0 | 0 | 178 | 9 | 14 | 2 | 0 | 0 | 36 | 2 | 32 | 9 | 0 | 6 | 2 | 0 | 7 |
