# Supplementary material for: Identification of Hub Genes Related to Carcinogenesis and Prognosis in Colorectal Cancer Based on Integrated Bioinformatics
Source: Mediators Inflamm. 2020 Apr 9;2020:5934821. doi: 10.1155/2020/5934821 (PMC7171686; doi:10.1155/2020/5934821)
Supplement: Supplementary 14 — Table S14: differential expression of PGPEP1L in human. [file 5934821.f14.docx]

| Gene | Species | Experiment accession | Comparison | log_2 fold change | Adjusted p-value |
| --- | --- | --- | --- | --- | --- |
| ENSG00000183571 | homo sapiens | E-GEOD-68086 | 'breast carcinoma' vs 'normal' | -4.4 | 3.74E-15 |
| ENSG00000183571 | homo sapiens | E-GEOD-68086 | 'colorectal carcinoma' vs 'normal' | -3.8 | 2.60E-12 |
| ENSG00000183571 | homo sapiens | E-GEOD-68086 | 'pancreatic adenocarcinoma' vs 'normal' | -3.8 | 4.14E-10 |
| ENSG00000183571 | homo sapiens | E-GEOD-59612 | 'contrast-enhancing core; glioma' vs 'normal' | -3.7 | 4.30E-28 |
| ENSG00000183571 | homo sapiens | E-GEOD-68086 | 'hepatobiliary carcinoma' vs 'normal' | -3 | 5.46E-05 |
| ENSG00000183571 | homo sapiens | E-MTAB-6013 | 'induced pluripotent stem cell' vs 'cardiac muscle cell' | 3 | 0.025615 |
| ENSG00000183571 | homo sapiens | E-MTAB-7340 | 'induced pluripotent stem cell' vs 'fibroblast of dermis' in 'normal' | 2.9 | 0.003502 |
| ENSG00000183571 | homo sapiens | E-GEOD-68086 | 'non-small cell lung carcinoma' vs 'normal' | -2.7 | 5.74E-08 |
| ENSG00000183571 | homo sapiens | E-GEOD-57116 | '3 h DRB 8 min 4sU' vs 'untreated' | 2.7 | 1.96E-05 |
| ENSG00000183571 | homo sapiens | E-GEOD-57116 | '3 h DRB 0 min 4sU' vs 'untreated' | 2.7 | 2.94E-05 |
| ENSG00000183571 | homo sapiens | E-MTAB-4054 | 'Barrett鈥檚 esophagus; low-grade dysplasia' vs 'normal' | -2.3 | 0.009413 |
| ENSG00000183571 | homo sapiens | E-GEOD-68086 | 'glioblastoma' vs 'normal' | -1.9 | 0.000402 |
| ENSG00000183571 | homo sapiens | E-GEOD-103501 | 'Growth Medium' vs 'none' in 'systemic-onset juvenile idiopathic arthritis' | -1.8 | 0.04142 |
| ENSG00000183571 | homo sapiens | E-GEOD-59612 | 'non-enhancing margin; glioma' vs 'normal' | -1.6 | 1.44E-08 |
| ENSG00000183571 | homo sapiens | E-GEOD-60585 | 'siRNAs against Integrator 11' vs 'control' in 'whole-cell RNA' | 1.5 | 0.000833 |
| ENSG00000183571 | homo sapiens | E-GEOD-59781 | 'PAX2 siRNA' vs 'control' | -1.2 | 0.011339 |
